# Whole transcriptome profiling reveals major cell types in the cellular immune response against acute and chronic active Epstein-Barr virus infection

**DOI:** 10.1038/s41598-017-18195-z

**Published:** 2017-12-19

**Authors:** Huaqing Zhong, Xinran Hu, Andrew B. Janowski, Gregory A. Storch, Liyun Su, Lingfeng Cao, Jinsheng Yu, Jin Xu

**Affiliations:** 1 Department of Clinical Laboratory, Children’s Hospital of Fudan University, Minhang District, Shanghai, 201102 China; 2 Departments of Pediatrics, Washington University School of Medicine, Saint Louis, Missouri 63110 United States; 3 Departments of Genetics, Washington University School of Medicine, Saint Louis, Missouri 63110 United States

## Abstract

Epstein–Barr virus (EBV) is a common human pathogen that infects over 95% of the population worldwide. In the present study, the whole transcriptome microarray data were generated from peripheral blood mononuclear cells from Chinese children with acute infectious mononucleosis (AIM) and chronic active EBV infection (CAEBV) that were also compared with a publicly available microarray dataset from a study of American college students with AIM. Our study characterized for the first time a broad spectrum of molecular signatures in AIM and CAEBV. The key findings from the transcriptome profiling were validated with qPCR and flow cytometry assays. The most important finding in our study is the discovery of predominant γδ TCR expression and γδ T cell expansion in AIM. This finding, in combination with the striking up-regulation of *CD3*, *CD8 and CD94*, suggests that CD8+ T cells and CD94+ NK cells may play a major role in AIM. Moreover, the unique up-regulation of *CD64A*/*B* and its significant correlation with the monocyte marker *CD14* was observed in CAEBV and that implies an important role of monocytes in CAEBV. In conclusion, our study reveals major cell types (particularly γδ T cells) in the host cellular immune response against AIM and CAEBV.

## Introduction

Epstein–Barr virus (EBV), also known as human herpesvirus-4, is one of the most important human pathogens worldwide. Primary EBV infection frequently occurs in the early life of an individual and by age 35–40, approximately 95% of the worldwide population has been infected^1,2^. In most cases EBV infection is asymptomatic because of a highly effective host immune response, but some individuals develop acute infectious mononucleosis (AIM) or chronic active EBV infection (CAEBV), while others develop EBV-associated lymphoid or epithelial malignancies^1–4^. Typically, AIM is a self-limiting disease with characteristic immunopathology including transient proliferation of EBV-infected B cells in the oropharynx and a robust immune response with CD8+ T cells in blood^3^. AIM may last a few weeks with the majority of AIM cases evolving into a lifelong latent phase of infection that occurs without notable clinical consequences^1,4^. In rare cases, primary EBV infection in an immunocompetent host can result in persistent or recurrent AIM-like symptoms including fever, lymphadenopathy, hepatosplenomegaly and liver dysfunction. A high EBV-DNA load can be detected in the peripheral blood in these cases. These cases are often classified as CAEBV^2,5^. Most cases with CAEBV have been reported from Japan with clonal expansion of EBV-infected T cells or natural killer (NK) cells^6–8^. CAEBV results in high morbidity and mortality with 5-year survival rates as low as 35%^6,9,10^. Currently, there is a lack of effective therapies for EBV infection although hematopoietic stem cell transplants have been successful for the treatment of CAEBV in several reports^2,5,9,10^. Further molecular studies on the immunopathology of EBV infection could help stimulate insights into development of new treatment options for improved outcomes in patients with CAEBV.

Gene expression profiling is a common approach for studies on molecular signatures for the immunopathology of EBV infection^11–14^. Microarray studies have been previously reported for Japanese children with CAEBV or American college students with AIM. In Zhang *et al*.^11^, Japanese children with EBV infections were studied and they identified a molecular signature from six SNK/T cell lines that were isolated from three patients with nasal NK/T-cell lymphoma and from the peripheral blood of three patients with CAEBV. Ito *et al*. analyzed gene expression profiles of peripheral blood mononuclear cells (PBMC) from four Japanese CAEBV patients and identified a set of 20 dysregulated genes^12^. Using PBMC from eight American college students with AIM, Dunmire *et al*. found that primary EBV infection induced an expression profile distinct from other viruses but similar to hemophagocytic syndromes^13^. In addition, Greenough *et al*. analyzed gene expression of sorted CD8+ T cells from 10 American college students with AIM. They reported a 28-gene molecular signature that correlated with CD8+ T cell expansion^14^. While these microarray profiling studies identified important molecular signatures and key genes associated with EBV infection, they have utilized sorted cells, cell lines, or microarray platforms with limited coverage of transcriptome, or they have been limited to specific populations with either AIM or CAEBV but not both in a single study.

In the present study, we performed a high-density microarray analysis at the whole transcriptome level with PBMC from both Chinese children with AIM and CAEBV. Our whole transcriptome study has the potential to provide a broader spectrum of molecular signatures than previous studies, to reveal distinct roles for different immune cells, and to identify unique key genes highly correlated with major immune cells by carrying out a direct comparison between AIM and CAEBV that is lacking in most of previous studies. In addition, we evaluated the similarities and differences in expression of major immune molecules between the present study and a publicly available gene expression dataset from a study of American college students with AIM^13^. This cross-dataset evaluation will help highlight differences in immunopathology of AIM between American college students and young Chinese children. Our study will expand our understanding of the immunopathology of EBV infection and will also serve as the basis for further exploration towards the development of diagnostic and therapeutic strategies for the EBV infection.

## Results

### Demographics and EBV infection status of study subjects

A total of 110 subjects were recruited in this study for microarray analysis and for qPCR and flow cytometry assays (Table S1 in Supplementary information). Of those, 17 were in the primary cohort for whole transcriptome microarray profiling, including 6 with AIM (4 male/2 female, median age 2 years), 5 with CAEBV (3 male/2 female, median age 9 years) and 6 healthy controls (3 male/3 female, median age 3 years). The remaining 93 subjects made up the secondary cohort. Of those, 12 AIM, 4 CAEBV and 14 healthy control subjects were used in the qPCR assays, and 28 AIM and 35 healthy control subjects were used in the flow cytometry assays. All 46 AIM patients from the primary and secondary cohorts recovered fully without sequelae. Among 9 CAEBV patients, 6 experienced 2-3 recurrent episodes of AIM-like symptoms, 1 developed hemophagocytic syndrome, and 2 died after 9-10 months.

As shown in Table 1, plasma EBV DNA was detectable in 19 of the 46 cases with AIM and all 9 patients with CAEBV, with measurements ranging from 1,020 to 48,300 (median 3,280) for AIM and from 3,440 to 1,460,000 (median 53,200) copies/ml of blood for CAEBV. This corresponded to a ~16-fold higher median EBV DNA load in plasma from patients with CAEBV than that from patients with AIM. Leukocyte EBV DNA was also detected in 40 patients with AIM, ranging from 1,190 to 3,760,000 (median 40,950) copies/ml of blood. Additionally, all 46 patients with AIM were positive for EBV viral capsid antigen (VCA)-IgM, and 38 were also positive for EBV VCA-IgG. All 9 patients with CAEBV were positive for EBV VCA-IgG and negative for EBV VCA-IgM. The serological data also indicated that 87% (48/55) of healthy control subjects were previously exposed to EBV with a positive EBV VCA-IgG. Furthermore, the predominant cell type of EBV-infected cells was determined for 5 CAEBV patients, of which 1 was CD8+ T cells (Subject ID: 15VC2295), 1 was CD56+ NK cells (Subject ID: 15VC3454), and the remaining 3 were CD4+ T cells.

**Table 1:** EBV Laboratory Results for the Study Subjects.

EBV Test	Acute EBV (N = 46)	Chronic Active EBV (N = 9)	Healthy Control (N = 55)
EBV VCA-IgM (# positive)	46	0	0
Primary cohort	6	0	0
Secondary cohort	40	0	0
EBV VCA-IgG (# positive)	38	9	48
Primary cohort	6	5	6
Secondary cohort	32	4	42
EBV ENA-lgG (# positive)	0	8	47
Primary cohort	0	5	4
Secondary cohort	0	3	43
EBV DNA load in plasma [#; range; median (copies/ml blood)]	19; 1020~48300; 3280	9; 3440~1460000; 53200	ND
Primary cohort	3; 3680~13400; 10300	5; 3440~1460000; 36000	ND
Secondary cohort	16; 1020~48300; 3350	4; 5840~202000; 29765	ND
EBV DNA load in WBC [#; range; median (copies/ml blood)]	40; 1190~3760000; 40950	ND	ND
Primary cohort	4; 2380~513000; 55290	ND	ND
Secondary cohort	36; 1190~3760000; 40950	ND	ND

Note: ND –not determined.

### Differential expression profiles of host whole transcriptome

**Figure 1:**
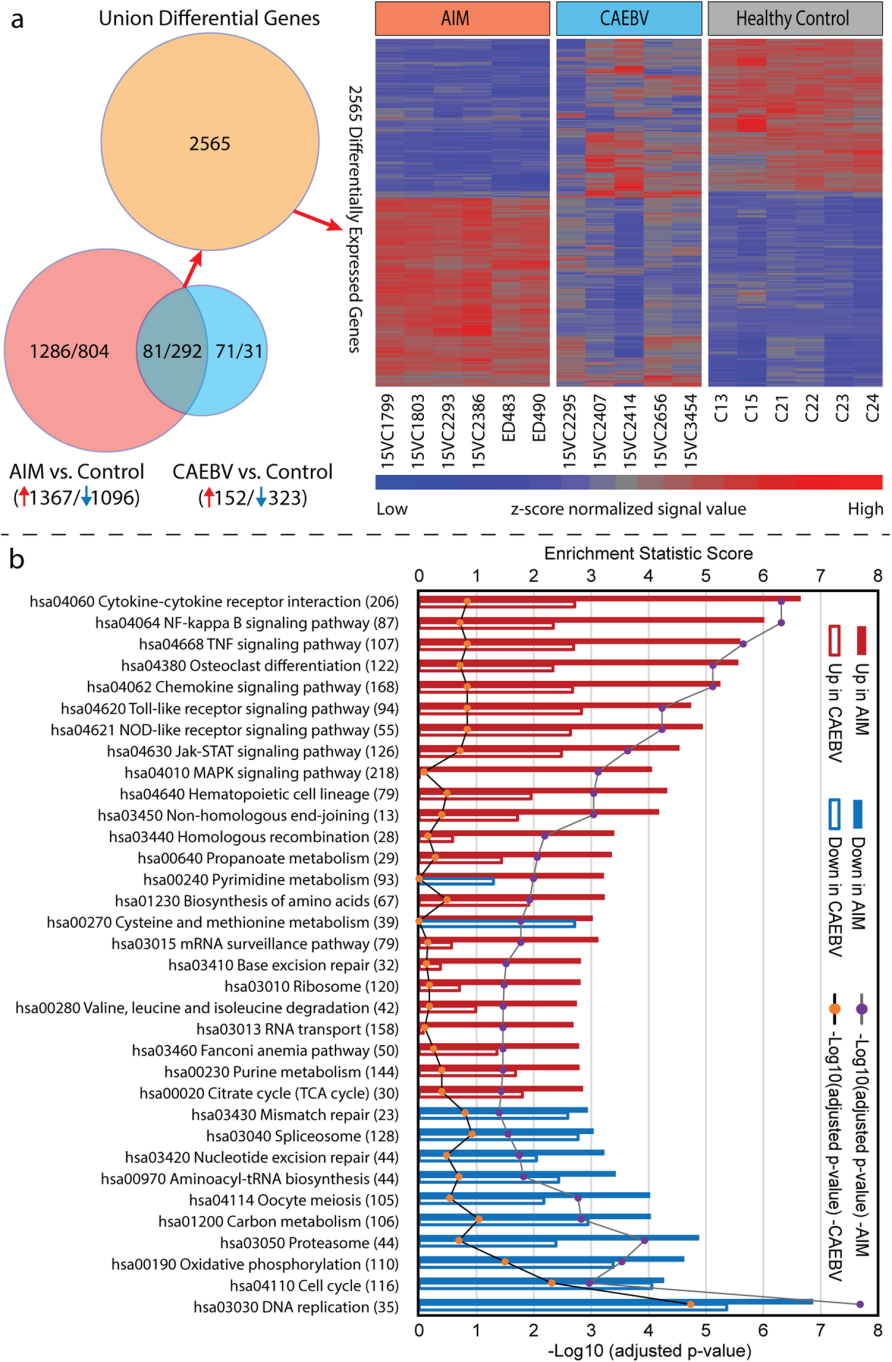
Overview of the whole transcriptome regulation in our primary cohort. (**a**) Differentially expressed union 2565 genes’ profiles for 6 acute infectious mononucleosis (AIM), 5 chronic active EBV infection (CAEBV) and 6 healthy control subjects. The Venn diagram depicts how many genes were differentially expressed in each comparison and how many genes overlapped between comparisons. Intensity values were normalized by quantile across samples and by z-score across genes. In the heatmap, each row represents a gene and each column represents a sample, with the 17 samples labeled at the bottom. (**b**) Significantly enriched KEGG pathways. Each pathway is labeled by KEGG pathway ID and name. The number of genes in a pathway is given in parenthesis. Solid bars are for AIM, and open bars are for CAEBV. Up-regulation is marked with red and down with blue. The p-value reflects the level of confidence for enrichment significance; and the enrichment score is an equivalent to the t value in a Student t-test and can be a gauge for the degree of pathway dysregulation.

The whole transcriptome expression profiles were defined for the 17 subjects in the primary cohort, with blood samples taken 2–7 (median 3) days after disease onset for 6 AIM cases, and 2–14 (median 3) months after disease onset for 5 CAEBV cases. In AIM, 2463 transcripts were significantly differentially expressed when compared to healthy controls, of which 1096 (44%) were down-regulated and 1367 (56%) were up-regulated (Table S2 in Supplementary information). In CAEBV, 475 transcripts were significantly altered when compared to healthy controls, with 323 (68%) transcripts down-regulated and 152 (32%) transcripts up-regulated (Table S3 in Supplementary information). The expression profiles of the 2565 transcripts that were differentially expressed in both or either of AIM and CAEBV groups demonstrated distinct transcriptional signatures for AIM and CAEBV (Fig. 1a).

### Pathway enrichment of host whole transcriptome

To attain an overview of host biological pathway regulations, we employed the generally applicable gene set and pathway enrichment (GAGE) analysis tool^15^ to map our whole transcriptome microarray data obtained from the primary cohort onto the signaling and metabolism pathways in the Kyoto Encyclopedia of Genes and Genomes (KEGG) database. Of the 202 pathways, 24 were up-regulated and 10 were down-regulated in AIM, whereas none was up-regulated and 3 were down-regulated in CAEBV. As shown in Fig. 1b, the up-regulated pathways were involved in cell cycle, DNA replication and repair, and energy production, and the down-regulated pathways were mostly related to immune signal transduction, such as cytokine-cytokine receptor interaction, NF-kappa B signaling pathway, TNF signaling pathway, and Toll-like receptor signaling pathway.

### Differential expression of host immune mediators

To characterize the molecular signature of the host immune system, we enriched the microarray data for gene expression changes in host immune mediators, including interleukins, interferons, chemokines and complement components. First, we compared the expression of these mediators between AIM and CAEBV in our primary cohort, and then evaluated the differences and similarities between subjects with AIM from our primary cohort and the Minnesota cohort^13^. A total of 303 transcripts for the immune mediators were found in our HTA2.0 microarray data (Table S4 in Supplementary information), of which 75 (24.7%) changed their expression levels significantly in AIM and 42 (13.9%) in CAEBV. Thirty-five genes were common between the 75 in AIM and the 42 in CAEBV. While many of these immune mediators (62/75, 82%) were down-regulated in AIM, 3 mediators (*CCL5*, *CCR5*, and *IL32*) were notably up-regulated in AIM. These 3 mediators have been reported to be able to attract T cells to infection site and to activate T and NK cells^16,17^.

**Figure 2:**
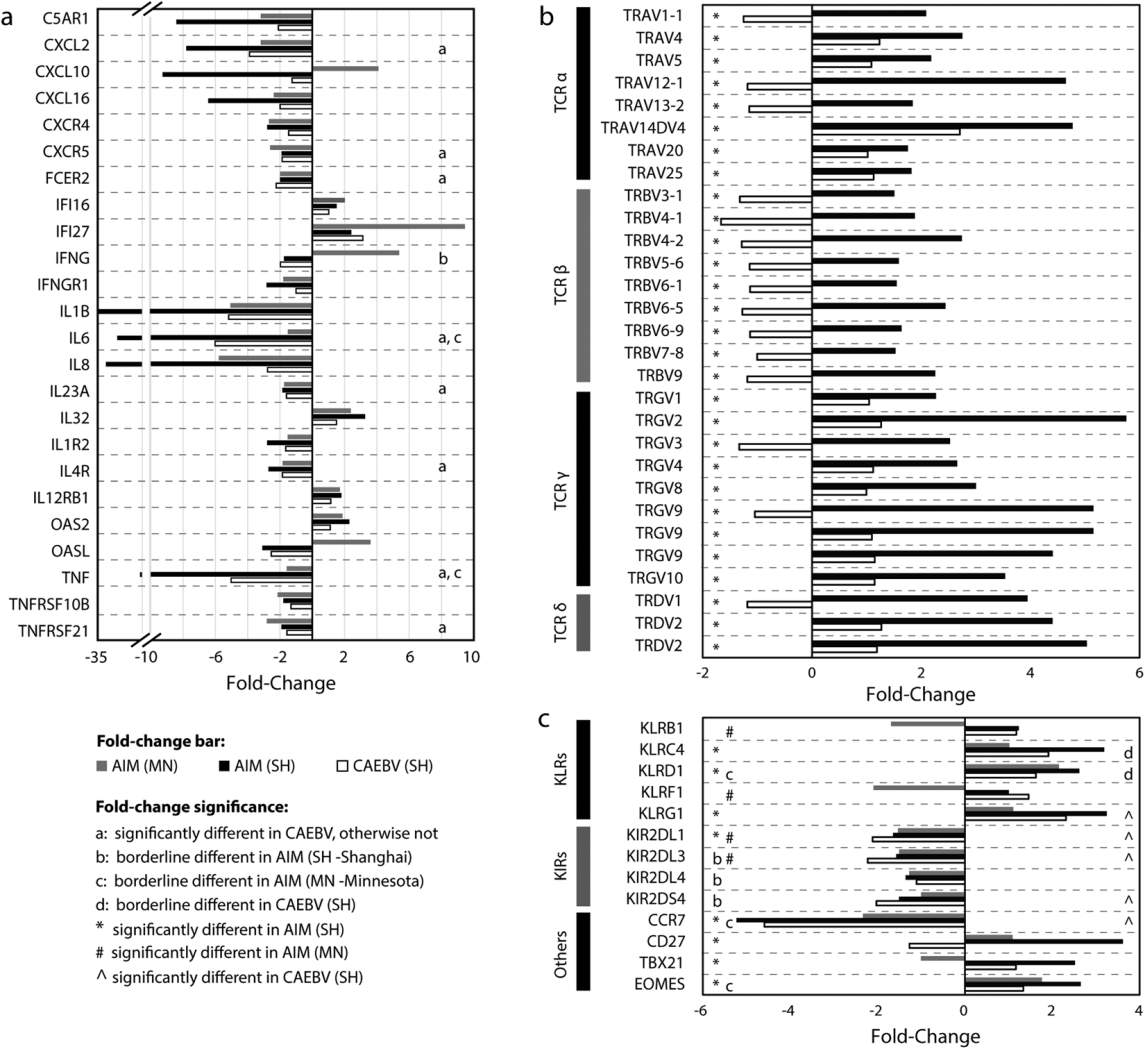
Bar charts of fold-change data. (**a**) 24 major immune mediators, of which 21 were significantly dysregulated in patients with acute EBV infection (AIM) from both our primary cohort (Shanghai) and the Minnesota cohort. Additional 3 important immune mediators (*IFNG*, *IL6*, and *TNF*) were also included in the chart although their expression levels were just borderline different in either of the two cohorts. The “significantly different” means adjusted p < 0.05; and the “borderline different” indicates an adjusted p-value of 0.05 to 0.20. The panel b shows the fold-change data for 26 T cell receptors (TCRs), and the panel c for 5 killer cell lectin-like receptors (KLRs), 4 killer cell immunoglobulin-like receptors (KIRs), and 2 other receptors and 2 transcription factors that are important for differentiation of lymphocytes.

In the Minnesota microarray dataset^13^, 294 immune mediators were found and 30 of them (10.2%) were differentially expressed, including 19 (19/30, 63%) down-regulated and 11 up-regulated (Table S4 in Supplementary information). To show the similarities and differences in transcription regulation of important immune mediators between AIM subjects from our primary cohort and the Minnesota cohort, we plotted the fold-change data for 24 genes in Fig. 2a, including 21 significantly dysregulated genes that were defined in both cohorts and 3 additional genes (*IFNG*, *IL6* and *TNF*) that were borderline dysregulated in either of the two cohorts. Except for a few genes, the majority of these immune mediator genes were concordant in the transcription regulation between cases with AIM from our primary cohort and the Minnesota cohort as shown in Fig. 2a.

### Differential expression of cytotoxicity cell receptors

The cellular immune response is an important part of the host response to EBV infection. Thus, we analyzed the expression of cell markers specific for cytotoxic T cell (including αβ T, γδ T, and NK-like T) and NK cell receptors. On the HTA2.0 chip, there are 84 transcripts for T cell receptors (TCRs), of which 62 are for functional TCRs. Because of the high complexity of the TCR genome, we determined whether each transcript sequence is explicitly representative of a single TCR gene. In the UCSC Genome Browser, HTA2.0 sequences for 3 of the 62 transcripts had multiple hits to human genome, and 2 additional transcripts (HTA2.0 transcript IDs: TC07001294.hg.1 and TC14000090.hg.1) were found to cover *TRGV9* and *TRDV2*, respectively. In 167 exon sequences (called “PSR”) for these 2 transcripts, 5 perfectly matched exon sequences for *TRGV9* and *TRDV2*. Finally, 57 transcripts and 5 PSRs for 59 functional TCR genes were examined with the HTA2.0 microarray data for our primary cohort. It was found that 26 of 59 TCR transcripts were significantly up-regulated (Fig. 2b) in AIM compared to healthy controls, with an average fold-change of 2.30 for 17 TCR α/β variable segments and 3.99 for 9 TCR γ/δ variable segments. In contrast, expression levels for none of the 59 functional TCR transcripts were altered significantly in CAEBV when compared to healthy controls. Comparative evaluation of the TCR expression in AIM subjects between our primary cohort and the Minnesota cohort was impossible due to the lack of TCR probes in the Minnesota dataset.

We also analyzed two additional sets of cytotoxicity-mediating cell surface receptors, including killer cell lectin-like receptors (KLRs) and killer cell immunoglobulin-like receptors (KIRs). In the microarray data from our primary cohort, 3 KLRs (*KLRC4/K1*, *KLRD1*, *KLRG1*) were up-regulated significantly in AIM (adjusted p < 0.05 for all) and significantly or moderately in CAEBV (adjusted p = 0.007 for *KLRG1*, 0.07 for *KLRC4*, and 0.19 for *KLRD1*, Fig. 2c). Of the KIRs, 3 (*KIR2DL1*, *KIR2DL3*, *KIR2DS4*) were down-regulated significantly in CAEBV (adjusted p < 0.05) and moderately in AIM (adjusted p < 0.09) (Fig. 2c). Furthermore, *CD27*, a receptor required for generation and long-term maintenance of T cell immunity, was significantly up-regulated in AIM but not in CAEBV. *CCR7*, a homing molecule that directs lymphocytes toward central lymphoid tissues such as lymph nodes, was significantly down-regulated by ~5 fold in both AIM and CAEBV. Two transcription factors, *TBX21* and *EOMES* known to be necessary for the differentiation of effector CD8+ T cells were significantly up-regulated by ~2.6 fold in AIM but not in CAEBV (Fig. 2c).

In the Minnesota dataset, the expression of many KLRs and KIRs was similar to our data except for *KLRB1* and *KLRF1* that were down-regulated in the Minnesota dataset and exhibited no significant change in our data, as shown in Fig. 2c.

### Distinct expression patterns of blood immune cells

**Figure 3:**
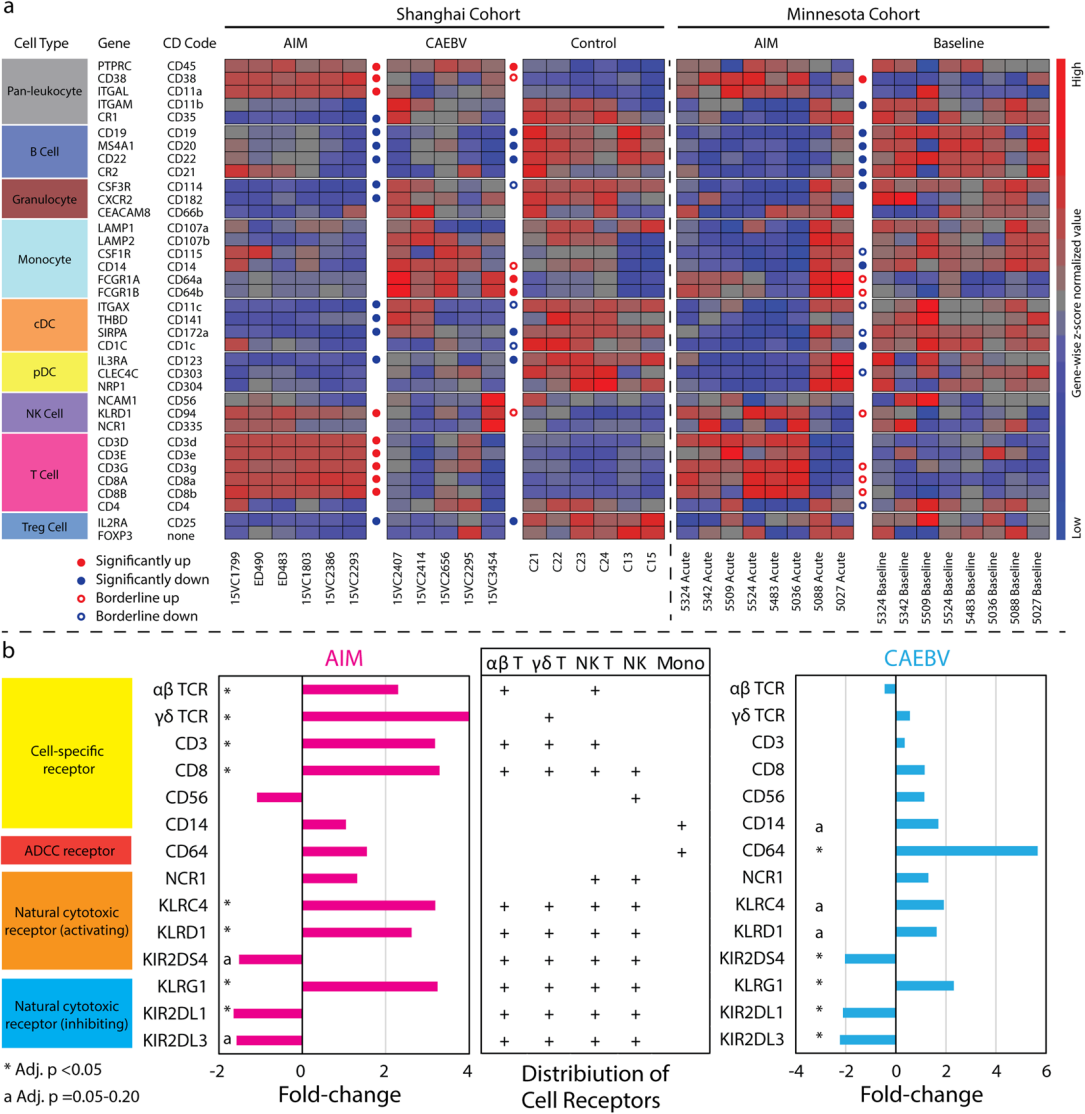
Expression patterns of immune cell markers and cytotoxicity cell receptors. (**a**) The expression of the 36 major immune cell markers was profiled with transcriptome data from our primary cohort (Shanghai) and the Minnesota cohort. “Significantly up or down” signifies that fold-change and adjusted p-value for the gene met the interpretation cutoffs set in the Method section, i.e. absolute fold-change >= 1.5 & adjusted p < 0.05. “Borderline up or down” indicates that these genes met the fold-change cutoff but the adjusted p-values for these genes were in the range of 0.05 to 0.20. Sample IDs are labelled at the bottom of heat maps. cDC: conventional myeloid dendritic cell, pDC: plasmacytoid dendritic cell, and Treg: T regulatory cell. (**b**) The expression regulation of major cytotoxicity cell receptors was analyzed with transcriptome data from our primary cohort. Mean fold-change and adjusted p values were used in the bar chart for TCRs, *CD3*, *CD8*, and *CD64* expression. ADCC: antibody-dependent cell-mediated cytotoxicity.

To characterize the contribution of different immune cells in the immunopathology of EBV infection, we examined the expression levels of 36 cell markers for peripheral blood immune cells in our primary cohort and the independent Minnesota cohort^13^ (Fig. 3a). These 36 cell markers were selected according to the field consensus from major research groups, such as the Biolegend Inc. and BD Biosciences. In the comparison between AIM and CAEBV in our microarray dataset, many similarities were seen, as shown in Fig. 3a, such as down-regulation of B cell, cDC, pDC, and granulocyte markers, up-regulation of NK cell markers such as *CD94* (but not *CD56*), and striking up-regulation of CD8+ cytotoxicity cell markers. The most significant difference was that the up-regulation of the monocyte markers *CD64A*/*B* was seen only in CAEBV and not in AIM.

The comparative evaluation between patients with AIM from our primary cohort and the Minnesota cohort demonstrated the same similarities as found in the AIM versus CAEBV comparison within our primary cohort as described above. The differences were also seen; for example, marginal up-regulation of *CD64A*/*B* and significant down-regulation of *CD21* (EBV receptor on B cells) were only observed in cases with AIM from the Minnesota cohort, and down-regulation of Treg cell marker *CD25* was solely seen in patients with AIM from our primary cohort.

Intriguingly, an activating pattern of cell-specific and cytotoxicity cell receptor expressions was observed in our primary cohort (Fig. 3b), with the predominant activation of cytotoxic T cells (particularly γδ T cells) in AIM and CD64+ monocytes in CAEBV. Moreover, the balance between activating and inhibiting KLRs and KIRs was towards activation for cytotoxicity cells, i.e. both CD8+ T and NK cells in AIM and NK cells in CAEBV.

### Correlation of immune cell markers with EBV DNA load

**Figure 4:**
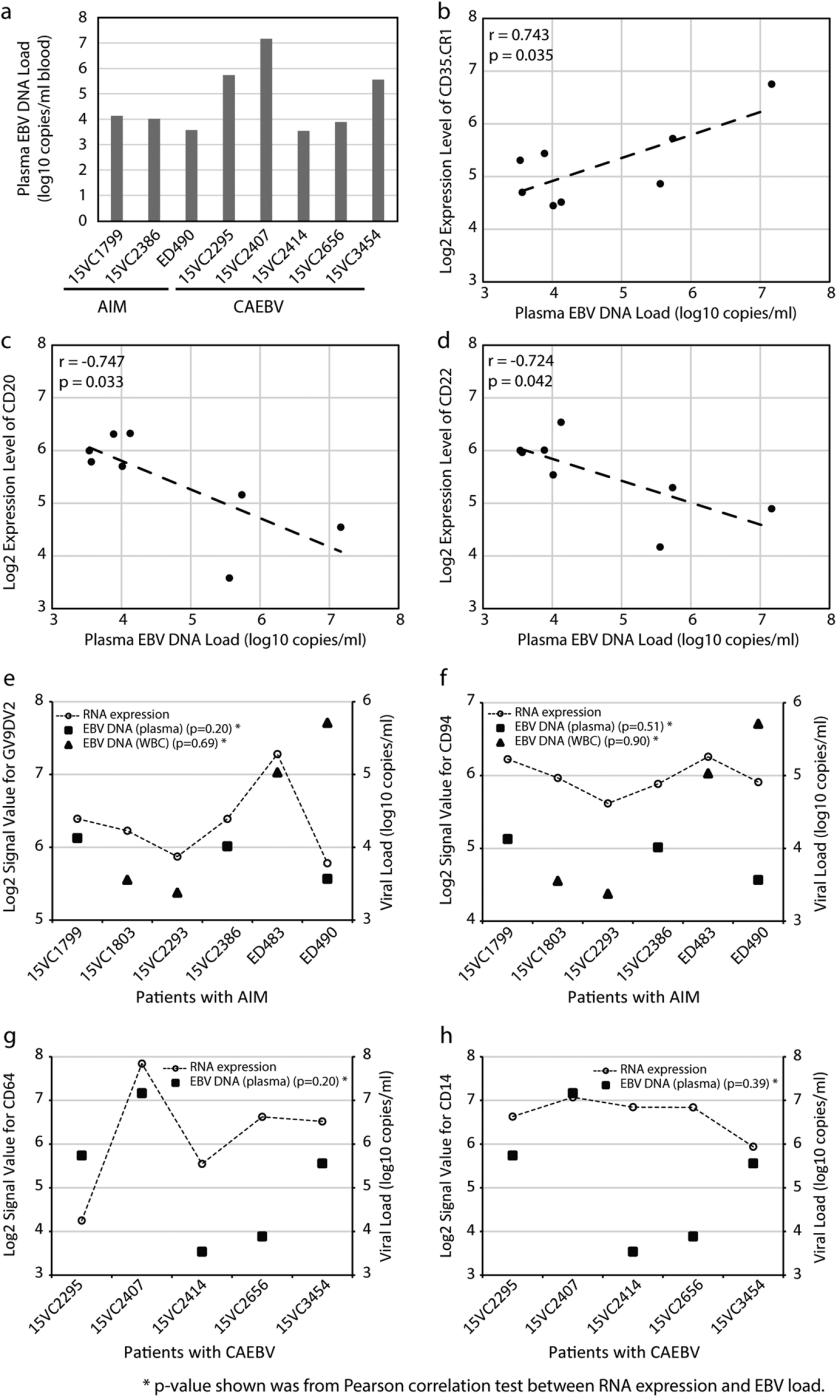
Correlation between plasma EBV DNA load and blood immune cell markers. In 8 EBV patients, including 3 AIM and 5 CAEBV cases (Note: 3 of 6 cases with AIM had undetectable level of plasma EBV DNA load), (**a**) shows the data for plasma EBV DNA load, (**b**) shows a positive correlation with *CD35*, (**c**) shows a negative correlation with *CD20*, and (**d**) shows a negative correlation with *CD22*. In all 6 AIM cases profiled by the microarrays, the RNA expression of GV9DV2 TCR (panel e) and *CD94* (panel f) was not significantly correlated with EBV loads, neither of *CD64* (panel g) and *CD14* (panel h) in all 5 CAEBV cases.

It was observed that the plasma EBV DNA load detectable in 8 cases with EBV infection (3 AIM and 5 CAEBV) was negatively correlated with the expression level of B cell markers *CD20* (r = −0.747, p = 0.033) and *CD22* (r = −0.724, p = 0.042), and positively correlated with a leukocyte marker *CD35* (r = 0.743, p = 0.035) (Fig. 4a–d). However, the EBV DNA load in plasma and white blood cells did not correlate with the expression level of any T cell, NK cell, granulocyte, monocyte, or other cell markers, including Vgamma9Vdelta2 TCR (*GV9DV2*), *CD94*, *CD64*, and *CD14* (all p > 0.05, Fig. 4e–h).

### Hyperactive and hypoactive genes in acute EBV infection

**Figure 5:**
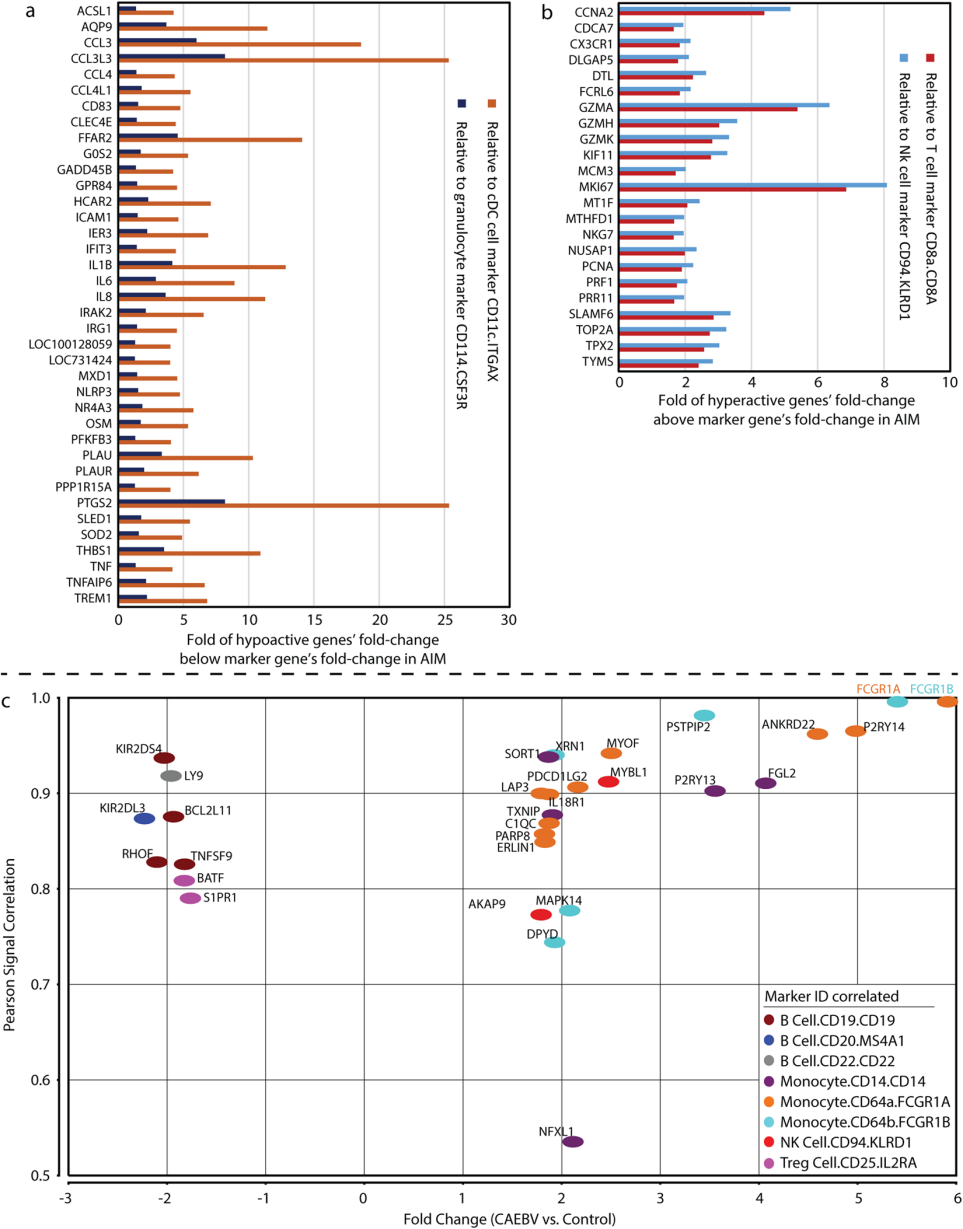
Key genes defined through whole transcriptome profiling analysis. (**a**) Hypoactive and (**b**) hyperactive genes identified in patients with acute EBV infection (AIM) in our primary cohort. The expression levels of the 61 genes were highly correlated with those of blood immune cell markers at Pearson correlation coefficient >= 0.8 and their fold-changes were at least 1.5 fold below (panel A: hypoactive) or above (panel B: hyperactive) the fold-changes of a cell marker gene. The model cell markers (*CD11c*, *CD114*, *CD8a*, and *CD94*) were selected based upon the level of their overall correlation with individual genes. (**c**) Unique dysregulated genes (n = 102) in chronic active EBV infection (CAEBV). These genes were differentially expressed in CAEBV but not in AIM. There were 30 protein coding genes with absolute fold-change values greater than 1.75 at adjusted p < 0.05. Most of these up-regulated genes (15/22) were highly correlated with monocyte markers *CD64A/B* (*FCGR1A/B*) and *CD14*. Most of down-regulated genes (6/8) were correlated with EBV targeting B cell markers *CD19/20/22*.

Within the differentially expressed genes, we wanted to identify hyperactive and hypoactive genes whose expression levels exceeded simple changes in numbers of peripheral blood cell types. This was achieved through the signal correlation between expression levels of cell marker genes and all remaining genes on the chip. We set a cutoff of 0.8 for Pearson correlation coefficient to define highly correlated genes with the 36 markers for peripheral blood cell types We further selected the highly correlated genes with fold-changes that were at least 1.5 fold above or below the fold-change of cell marker genes. Applying this analysis to the AIM subjects in the primary cohort, we identified 38 down-regulated genes that we defined as hypoactive (Fig. 5a) and 23 up-regulated genes that we defined as hyperactive (Fig. 5b). The model cell markers were selected based upon the level of their overall correlation with individual genes and were displayed in Fig. 5a and b. The hyperactive genes were most significantly correlated with CD8+ T cell and CD94+ NK cell markers, while the hypoactive genes were significantly correlated with B cell, Treg, cDC, pDC and granulocyte markers (Figure S1 in Supplementary information).

### Unique dysregulated genes in chronic active EBV infection

With both acute and chronic active EBV infection cases included in the study, we were able to identify 102 genes dysregulated only in CAEBV through gene-list overlapping (Fig. 1a). We then selected 30 of the 102 genes that were protein coding with a fold-change greater than 1.75 for more efficient interpretation (Fig. 5c). Notably, *FCGR1A* and *FCGR1B* demonstrated the greatest up-regulation (>5 fold), and they were best correlated with the monocyte marker *CD14* (Pearson r = 0.68 and 0.73, respectively). Eleven of the 22 up-regulated genes were correlated with *FCGR1A/B*, whereas 4 (*FGL2*, *P2RY13*, *SORT1*, and *TXNIP*) were correlated with *CD14*. The other up-regulated gene, *MYBL1*, was best correlated with the NK cell marker *CD94* (*KLRD1*). The remaining 4 up-regulated genes were not importantly correlated with any cell markers (Pearson r < 0.8). On the other hand, expression levels for 6 of the 8 down-regulated genes (*BCL2L11*, *KIR2DL3*, *KIR2DS4*, *LY9*, *RHOF* and *TNFSF9*) were highly correlated with B cell markers (*CD19/20/22*), and 2 (*BATF* and *S1PR1*) were correlated with Treg cell marker *CD25*.

### Signal correlation of GV9DV2, CD94 and CD64 with certain cytokines

In order to define distinct cytokine signature associated with AIM and CAEBV, we analyzed the microarray signal correlation of 16 important cytokines selected according to the field consensus^18^ (10 lymphokines: *CSF2*, *IL2*, *IL3*, *IL4*, *IL5*, *IL6*, *IFNG*, *LTA*, *LTB*, *MIF* and 6 monokines: *CSF1*, *IL1A*, *IL1B*, *IFNA1*, *IFNB1*, *TNF*) with 3 key immune markers (*GV9DV2*, *CD94*, and *CD64*) identified in the present study. In the 6 AIM cases, the most significantly correlated cytokines with *GV9DV2* were three monokines (*CSF1*, *IL1B*, and *TNF*) (Pearson correlation r = 0.75–0.95 and p = 0.003–0.088), while one lymphokine (*LTB*) was moderately correlated with *CD94* (r = 0.74, p = 0.091). In the 5 CAEBV cases, two lymphokines (*IFNG* and *IL2*) were borderline correlated with *CD64* (r = 0.70–0.73, p = 0.16–0.18).

### Fold-change confirmation of immune response genes by qPCR

**Figure 6:**
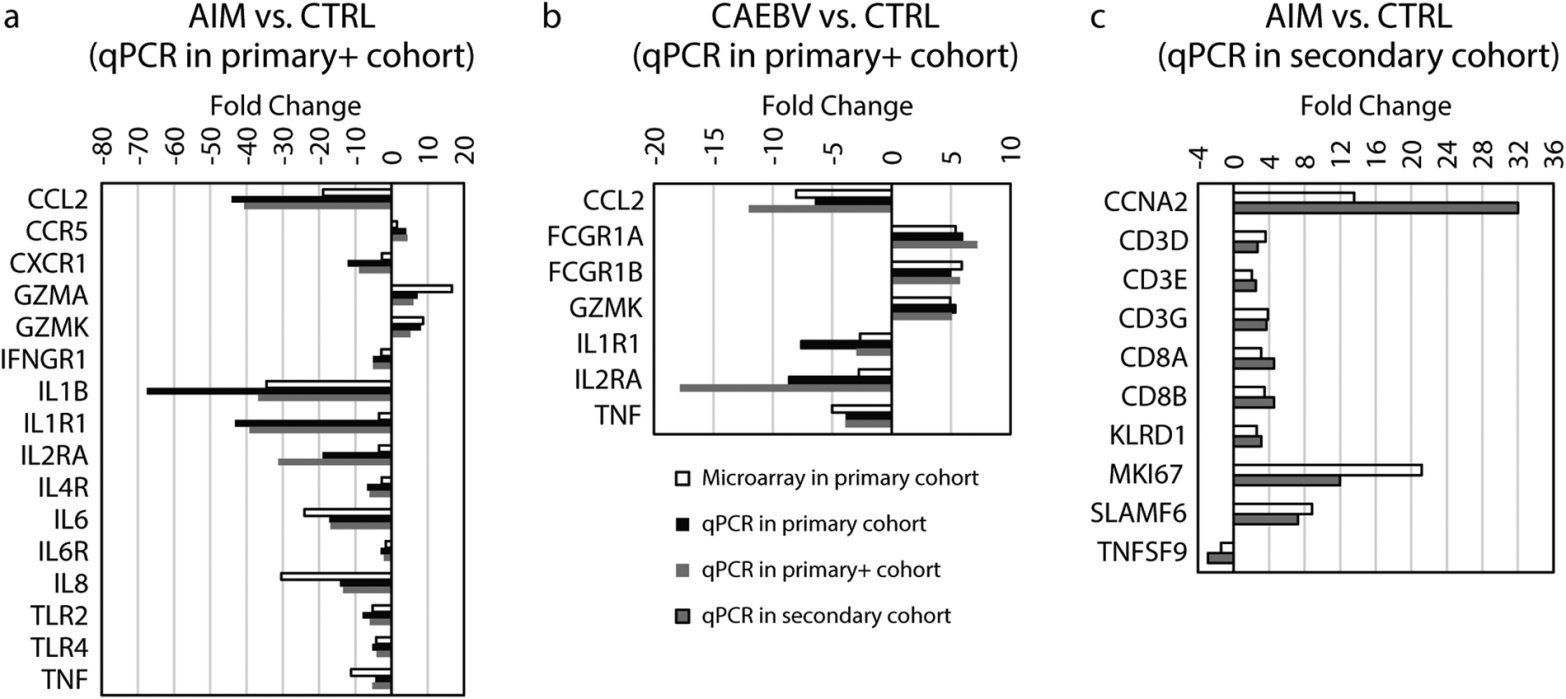
Data from the validation qPCR assays. (**a**–**c**) Bar charts present fold-change data from microarray and qPCR analyses for acute infectious mononucleosis (AIM) and chronic active EBV infection (CAEBV). The panels a & b: qPCR assays were performed in 27 cases and analyzed separately for the primary cohort (17 cases) and for the “primary+” cohort (27 cases: 17 from the primary and 10 from the secondary cohorts); and the panel c: qPCR assays were then performed in 26 cases solely from the secondary cohort.

To confirm the fold-change difference found by microarrays, a total of 28 immune mediator genes and a reference gene (actin-beta) were assayed by qPCR. The 28 genes were differentially expressed and defined by our microarray data in either or both of subjects with AIM and CAEBV from the primary cohort. These immune genes represented the important alterations found by our microarrays. Using RNA samples from the primary cohort, we first tested 16 genes for AIM and 7 genes for CAEBV, and concordant fold-changes were observed (Fig. 6a and b). These same sets of genes were then examined in a larger cohort (the “primary+” cohort), including 17 cases from the primary cohort and 10 additional cases from the secondary cohort. This further confirmed the fold-changes found by the microarrays. We then performed qPCR assays for 10 additional genes solely in the secondary cohort, including 12 cases with AIM and 14 healthy controls (We were unable to recruit additional cases with CAEBV into the secondary cohort during the time period for qPCR assays). This confirmed the fold-changes in an independent cohort (Fig. 6c). The qPCR assays confirmed the significant up- or down-regulation of these genes found by microarrays, indicating the reliable measurements that were used in the microarray transcriptome profiling analysis in our study.

### Expansion of granzyme-B+ cytotoxic cells, γδ TCR-specific T cells, and CD94+ killer cells

**Figure 7:**
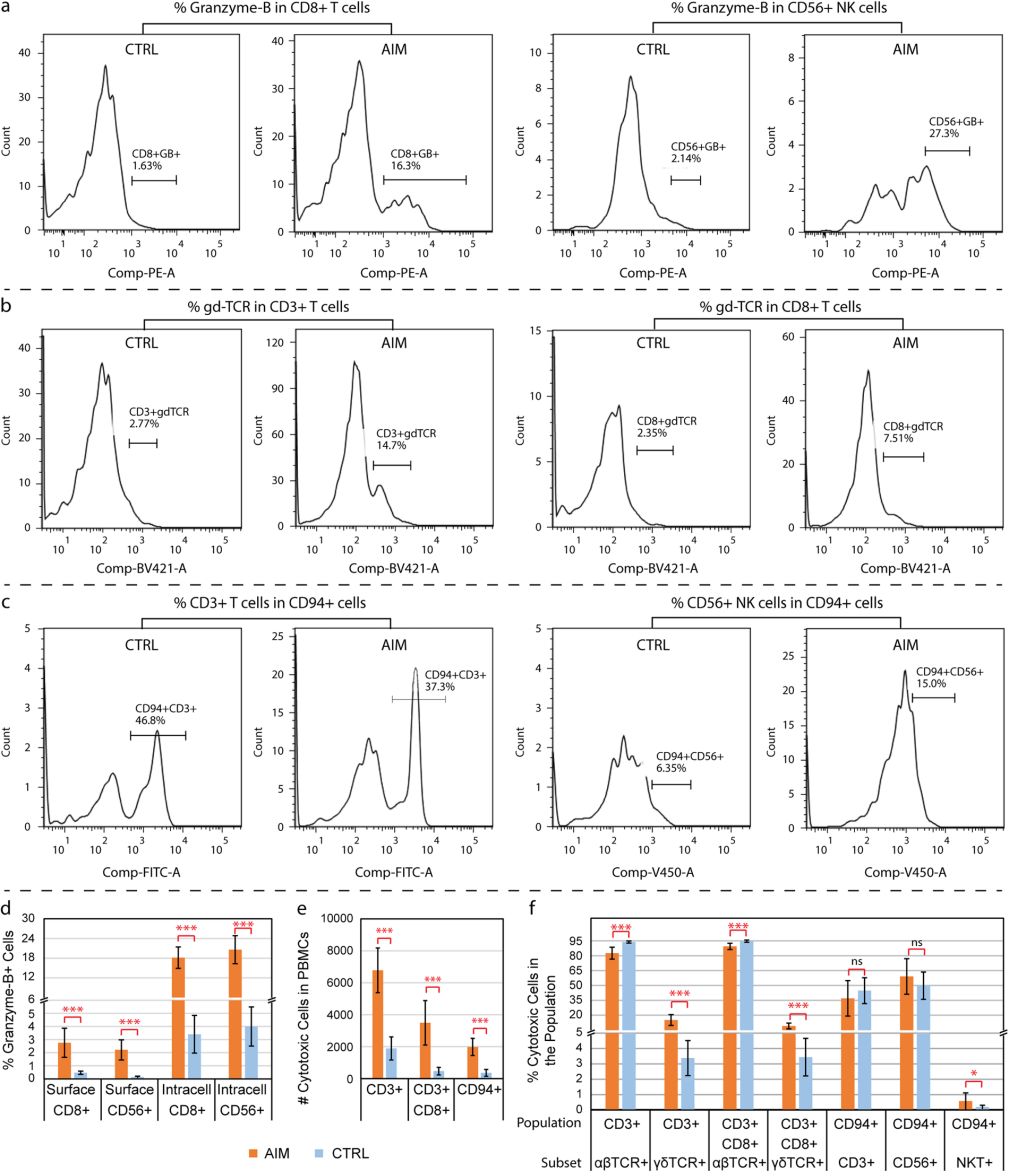
Data from the validation flow cytometry assays. Panels (a–c) show representative flow cytometry blots from experiments for frequencies of Granzyme-B, γδ TCR (gdTCR), and CD94-expressing cytotoxic cells. Panels (d–f) present the results summary from the flow cytometry analyses with statistics. Each bar represents the mean and standard deviation of the data for each cell population. (**d**) Percent of intracellular or surface granzyme-B + cells in the pools of CD3+ CD8+ and CD56+ cell populations from 8–10 subjects with acute infectious mononucleosis (AIM) and 10–15 healthy controls (CTRL) in the secondary cohort. (**e**) Number of cytotoxic cells in 10,000 PBMCs from 10 patients with AIM and 10 healthy controls. (**f**) Percent of cytotoxic cells in a specific cell population from the same cohort of 10 AIM and 10 healthy control subjects. Mann-Whitney U test was performed and the significant difference was marked with *(p < 0.05), ***(p < 0.001) and ns for no significance (p > 0.2) between AIM vs. CTRL.

The flow cytometry assays were performed to analyze the expansion of specific cytotoxic cell populations. The representative flow cytometry blots were shown in Fig. 7a–c. As a specific marker for cytotoxic lymphocytes, granzyme-B was used to determine the percentage of cytotoxic CD8+ T and CD56+ NK cells in the pool of PBMCs. The surface granzyme-B-positive cells were dramatically expanded by 8.4- and 15.2-fold in the populations of CD8+ T and CD56+ NK cells from the AIM subjects (Fig. 7d, all p < 0.001), respectively when compared to those from healthy control subjects. The intracellular granzyme-B-positive cells were also significantly increased by ~5 folds in these cell populations from AIM subjects (Fig. 7d, all p < 0.001). We were unable to recruit additional cases with CAEBV during the time period for the flow cytometry assays that requires freshly isolated blood cells.

The frequencies of αβ and γδ TCR-specific T cells and KLRD1/CD94+ killer cells in patients with AIM were analyzed using sets of specific antibodies with the flow cytometry assays. When compared to the age-matched healthy controls (median: 3 vs. 4 years old in AIM and controls), the number of positive cells for CD3, CD3+ CD8, and CD94 were all significantly increased in 10 patients with AIM (p < 0.001, Fig. 7e). The percent of αβ TCR-specific T cell population was relatively shrunken by 11% (82.5% vs. 93.8%, p < 0.001) in the CD3+ T cells and 5% (89.4% vs. 94.6%, p < 0.001) in the CD3+ CD8+ T cells from the 10 patients with AIM (Fig. 7f); whereas the percent of the γδ TCR-specific T cell population was significantly expanded by 4.5-fold (15.1% vs. 3.4%, p < 0.001) in the CD3+ T cells and 2.7-fold (9.1% vs. 3.4%, p < 0.001) in the CD3+ CD8+ T cells from the 10 patients with AIM (Fig. 7f). In addition, the ratio between the percentage of αβ and γδ TCR-specific T cells in CD3+ PBMCs was 5.0-fold down in the patients with AIM (mean 6:1, range 2.4:1 to 8.7:1) when compared to the healthy controls (mean 30:1, range 15:1 to 40:1) (p < 0.001).

Among KLRD1/CD94-expressing killer cells, CD3+ T cells, CD56+ NK cells and NKT cells accounted for 37.0%, 59.1% and 0.6%, respectively, in the 10 patients with AIM. No statistical difference was found in percent of both CD3+ and CD56+ cells from CD94+ killer cells between the AIM and healthy control subjects (p > 0.2, Fig. 7f). Although the patients with AIM had a higher percentage of NKT cells (0.6% vs. 0.2%, p = 0.035) than the healthy controls, the absolute number of NKT cells was much less than CD3+ T cells and CD56+ NK cells in the pool of CD94-expressing killer cells.

## Discussion

The present study has characterized for the first time a broad spectrum of molecular signatures in PBMC from patients with AIM and CAEBV in a single analysis. It covers the major part of the host immune response including important immune mediators, cytotoxicity cell receptors, and distinct expressions of major peripheral blood immune cell markers. The most important finding in our study is the discovery of predominant γδ TCR expression and γδ T cell expansion in AIM. This is achieved through the probe sequence mapping of multiple TCR transcripts uniquely covered by the HTA2.0 microarrays and flow cytometry analysis. To our knowledge, this is the first TCR gene expression analysis in a whole transcriptome profiling study of EBV infection.

A notable advantage of the platform HTA2.0 in the present study is the comprehensive coverage of the human transcriptome. Particularly, the HTA2.0 microarray detects a large number of diverse T cell surface receptors (TCRs). This unique capability facilitated the discovery of a higher magnitude of up-regulation for γδ TCRs than that for αβ TCRs (3.99 vs. 2.30) in AIM in our study. A recent case report implicates the ability of γδ T cells to control EBV^19^. An earlier study also underlines the role of γδ T cells in AIM^20^. Two recent studies further highlighted the important role of γδ T cells in control of EBV infection and reactivation^21,22^. Djaoud *et al*.^21^ identified two alternate patterns of host innate immune response to EBV infection: NK cells alone and NK cells together with γδ T cells. In their study, Xiang *et al*.^22^ demonstrated that pamidronate treatment inhibited the development of Epstein-Barr virus-induced lymphoproliferative disease in humanized mice through selective activation and expansion of Vγ9Vδ2-T cells. γδ T cells are usually much less common than αβ T cells in blood, and are in highest abundance in the gut mucosa^23^.

Our microarray data demonstrated a likely predominant expansion of γδ T cells, including both Vδ1 and Vδ2 subsets, and this was confirmed in the flow cytometry experiments. The Vδ1 sunset might be induced and trafficked from the primary site of EBV infection (the nasopharyngeal mucosa), while the Vδ2 T cells expand within the peripheral blood^24^. These findings suggest that CD8+ cytotoxic T cells (predominantly γδ T cells) may play a major role in AIM. However, the expansion of these cells could also mediate the immune pathologies of the respective syndromes in patients, and be activated by classical T cell responses, failing to control the virus. Our flow cytometry assays demonstrated the significant expansion of granzyme-B-positive CD8+ T cells and γδTCR-specific T cells in AIM, supporting that these cells are the significant responder cells in the host cellular immune response against EBV infection. Our γδTCR RNA expression and γδ T cell population data did not exhibit the two alternate cellular response patterns defined in the Djaoud *et al*. study^21^ in the AIM cases from our primary and secondary cohorts. This may be due to the small number of cases included in our study.

NK cells are often considered the primary effectors of host innate immunity against EBV^25^. Our study showed no significant change in the expression level of the NK cell-specific marker, *CD56*, in either AIM or CAEBV subjects. It is worth noting that one case with NK cell-type CAEBV (Subject ID: 15VC3454) showed a stronger expression of *CD56* than those with non-NK cell-type CAEBV (Fig. 3a). Human NK cells can be divided into two subsets based on their cell-surface density of CD56, CD56bright and CD56dim^26^. The CD56dim NK cell subset is more naturally cytotoxic. Our results found up-regulation of certain NK cell-specific natural cytotoxicity receptors such as *CD94* (*KLRD1*) and *CD314* (*KLRC4*) (Figs 2c and 3a). The activating expression pattern of these killer cell receptors (including KLRs and KIRs, Fig. 3b) suggests that NK cells, likely the CD56dim NK cells, were activated in both AIM and CAEBV. It should be noted that *CD94* is expressed primarily by NK cells, but also by a subset of CD3+/CD8+ αβ T cells, γδ T cells and NK T cells^27^. Our flow cytometry studies found that both CD3+ T cells and CD56+ NK cells were major subpopulations in the CD94-expressing killer cell pool while the NKT cells were a minor subpopulation (Fig. 7f). The up-regulation of *CD94* is indicative of the involvement of both T cells and NK cells in the host innate immune response to EBV infection (Figs 3b, 7e and 7f).

The comparative evaluation with another patient population is another unique part of our study. It leads to the observation of many similarities and several differences in expression of major immune molecules in AIM between young Chinese children and American college students^13^. The major similarities are the significant expansion of CD8+ T cells and the dramatic down-regulation of B cell and granulocyte markers (Fig. 3a), which is consistent with current understanding of the immunopathology for AIM^1,3,13,14^. The major differences are discordant expression of some interferon pathway genes, such as *CXCL10*, *IFNG*, *OASL* (Fig. 2a), and some killer cell lectin-like receptors, such as *KLRB1* and *KLRF1* (Fig. 2c), which may represent distinct immunopathology for AIM in different populations and age groups. Our 6 AIM cases are much younger than 8 AIM cases from the Minnesota cohort (median 2 versus 18 years old)^3^. The differences observed for those interferon genes may depict distinct interferon response patterns in AIM between young Chinese children and American college students.

Basically, our study showed the down-regulation of multiple humoral immune mediators in AIM and CAEBV (Figs 2a and 5a). Most of these mediators were highly correlated with B cell and granulocyte markers (Figure S1 in Supplementary information). It is worth noting that the down-regulation of humoral immune mediators and B cell markers could also be the result of a change in the cellular composition in PBMC specimens from healthy controls and patients with EBV infection. Our results also demonstrate an extensive up-regulation of the cytotoxicity cell-specific markers, such as *CD3* and *CD8*, and their effectors (Figs 2a, 3a and 5b). This implicates the expansion of cytotoxicity T cells in AIM and an enhanced cytotoxic activity in host response to AIM^3^. Our results also showed elevated expression levels of *IL32* ^16^ and *IL12RB1* ^28^, both expressed mainly on activated T and NK cells. These results support the fact that the cellular immune is essential in the control of EBV infection as CD8+ cytotoxic T and NK cells control proliferating B cells that are largely infected by EBV^1,4^. Moreover, we examined the signal correlation of 10 lymphokines and 6 monokines with *GV9DV2*, *CD94*, and *CD64*. The results showed that 3 monokines (*CSF1*, *IL1B*, *TNF*) and 1 lymphokine *LTB* were correlated in some degree with the signal of *GV9DV2*/*CD94* in AIM, and 2 lymphokines (*IFNG*, *IL2*) with *CD64* in CAEBV. It was noted that 3 of the 6 cytokine (*CSF1*, *IL1B*, *TNF*) were down-regulated and the other 3 (*IFNG*, *IL2* and *LTB*) had no significant changes in their RNA expression level in AIM and/or CAEBV when compared to healthy controls. In fact, many of these cytokines could be secreted from both lymphocytes and monocytes. Based on these findings, a definite lymphokine and monokine signature could not be established for AIM and CAEBV in our study.

In addition, our study identified a number of hyperactive and hypoactive genes through signal correlation analysis. While the hypoactive genes are largely associated with the significant reduction in B cell and granulocyte numbers in AIM, many of the hyperactive genes are major immune response receptors, effectors, and regulators for cytotoxicity cells, such as *CX3CR1*, *FCRL6*, *GMZA/H/K*, *NKG7*, *PRF1*, and *SLAMF6* (Fig. 5b). Of those genes, *FCRL6* encodes Fc receptor-like 6, a cell surface glycoprotein with tyrosine-based regulatory potential^29–31^, and its expression is restricted to mature lymphocytes with cytotoxic capability. Studies have shown that *FCRL6* is a useful marker for distinguishing perforin-expressing cytotoxic lymphocytes, such as CD56dim NK cells, Vδ1+ and Vδ2+ γδ T cells, effector memory CD8+ T cells, and rare cytotoxic CD4+ T cells^29^. Future functional studies can be focused on these hyperactive genes to help understand the immunopathology of EBV infection.

Importantly, the present study identified 30 unique dysregulated genes in CAEBV. Five of the 30 genes (*ANKRD22*, *FCGR1A*, *GBP5*, *LAP3*, and *P2RY14*) are among a set of 10 up-regulated genes identified by Ito *et al*. in their cases with CAEBV^12^. In addition, several phagocytosis-associated genes such as *C1QC*, *FGL2* ^32^ and *PSTPIP2* ^33^ as well as monocyte markers *FCGR1A* and *FCGR1B* (*CD64A/B*) are among the group of CAEBV-unique dysregulated genes. In light of *FCGR1A/B* being high-affinity receptors for the Fc portion of an antibody^34^, our transcriptome profiling results depict a relatively hyperactive phagocytosis and monocyte-mediated antibody-dependent cellular cytotoxicity (ADCC) in CAEBV. *CD64* is expressed on monocytes, macrophages, dendritic cells, and activated granulocytes. Our results showed that the expression of many CAEBV-unique genes was highly correlated with the level of *CD64*, but not with that of cytotoxic T cell (*CD3*, *CD8*) and NK cell (*CD56*, *CD94*) markers. Furthermore, the expression of *FCGR1A/B* was best correlated with the monocyte marker *CD14*. These findings suggest that monocytes may play an important role in the cellular immune response to CAEBV through hyperactive phagocytosis and ADCC.

Lastly, our study found significant correlations between plasma EBV DNA load and the expression levels of B-cell markers *CD20*, *CD22*, and leukocyte *CD35* (Fig. 4b–d) in AIM and CAEBV. In fact, prior studies have demonstrated that the EBV viral load in the blood can be used to monitor episodes of EBV infection or reactivation^35,36^. In addition, the EBV DNA load is associated with the disease severity and number of CD8+ cells^3^. In our study, the negative correlation of plasma EBV DNA load with B cell markers *CD20*/*CD22* may indicate the lytic effect of the virus on B cells commonly in AIM and CAEBV. The positive correlation of plasma EBV DNA load with leukocyte marker *CD35* can reflect active immune response against EBV through *CD35*-expressed dendritic cells, monocyte, and T/NK cells. As one of the interactive mechanisms between EBV and leukocytes, *CD35* binds immune complexes coated with C3b or C4b and mediates their transport to and removal by the fixed phagocyte systems of the spleen and liver.

While our study reveals a number of significant immune response alterations in patients with AIM and CAEBV, some issues associated with EBV infection still remain to be elucidated in future studies. First, the biological significance of some of the hyperactive genes in AIM as well as the uniquely dysregulated genes in CAEBV requires further investigation. Second, transcriptional differences between AIM patients with and without subsequent chronic courses would help resolve how and why CAEBV develops in some AIM cases but not in other cases. Additionally, studies on additional CAEBV cases with predominant involvement of each of the different subtypes of EBV-infected cells (B, T, and NK cells) would allow identification of subtype-specific molecular signatures.

In summary, our study has characterized for the first time a broad spectrum of molecular signatures in PBMC from patients with AIM and CAEBV. It covers the major part of the host immune response including important immune mediators, cytotoxicity cell receptors, and distinct expressions of major peripheral blood immune cell markers. The key findings from the transcriptome profiling were validated with qPCR and flow cytometry assays. The most important finding in our study is the discovery of predominant γδ TCR expression and γδ T cell expansion in AIM. This finding, in combination with the striking up-regulation of *CD3, CD8 and CD94*, suggests that CD8+ T cells and CD94+ NK cells may play a major role in AIM. Moreover, our transcriptome data found the unique up-regulation of *CD64A/B* and its significant correlation with the monocyte marker *CD14* in CAEBV and that implies an important role of monocytes in CAEBV. In conclusion, our study reveals major cell types (particularly γδ T cells) in the host cellular immune response against acute and chronic active Epstein-Barr virus infection. The characterized molecular signatures and major cell types will help direct future functional studies on the pathogenesis of EBV infection and stimulate insights into development of new treatment strategies for patients with CAEBV.

## Materials and Methods

### Study subjects

EBV patients and healthy controls who visited the Children’s Hospital of Fudan University in Shanghai, China during 2014–2017 were invited to participate in this study. A total of 17 subjects were recruited in the primary cohort for microarray analysis, including 6 healthy control subjects, 6 acute infectious mononucleosis (AIM), and 5 chronic active EBV infection (CAEBV) patients. Ninety-three additional subjects were recruited as a secondary cohort for quantitative PCR analysis and flow cytometry assays, including 49 heathy subjects, 40 patients with AIM and 4 cases with CAEBV. The study protocol was approved by the Ethics Committee for Clinical Investigation of the Children’s Hospital of Fudan University. The parents of all child subjects provided written informed consent on their behalf. The standard regulations for the use of human specimens were followed.

The healthy controls were defined as those who presented for routine health examination without apparent symptoms of any illness. AIM met the following criteria for the diagnosis: (1) fever with tonsil swelling, lymphadenopathy, splenomegaly, hepatomegaly, and/or liver enzyme abnormalities; (2) mononucleosis: an abnormal increase of white blood cells that have a single nucleus (mononuclear cells) in the blood; and (3) laboratory-confirmed primary AIM. A positive viral capsid antigen (VCA)-IgM was required to meet the definition of AIM. The VCA-IgG can be either positive or negative but the anti-extractable nuclear antigen (ENA) antibody must be negative to diagnose primary AIM. CAEBV met the following criteria for the diagnosis, in accordance with proposed guidelines^37^: (1) Persistent or recurrent AIM-like symptoms; (2) Unusual pattern of anti-EBV antibodies with raised VCA-IgG and ENA-IgG, or quantities of EBV in the peripheral blood greater than 500 copies/ml blood; and (3) Chronic illness without previous immunologic abnormalities or any other recent infection. All EBV patients received general supportive treatments, and had not been treated with any immunosuppressive agents or antivirals.

### EBV serology, DNA load and infected cells

Blood samples were drawn from all subjects in the study and the EBV serology profile was measured using the commercial EBV test kit from the EUROIMMUN Corporation (Lubeck, Germany). The EBV DNA load was measured using a PCR-based commercial kit from the DAAN Gene Co. Ltd (Guangzhou, China). The standard protocols from the manufacturers were followed for these assays. For EBV-infected cells, mononuclear cells were separated from peripheral blood through the Ficoll process, and then were fractionated into CD19+, CD4+, CD8+, or CD56+ cells using specific MicroBeads reagents from Miltenyi Biotech (San Diego, CA, USA) following the manufacturer’s instructions. After the specific cell separation, the EBV loads were determined in each of the fractionated cell types by quantitative PCR. Patients were defined as CD4, CD8, or NK cell type of EBV infection when CD4+ cells, CD8+ cells, or CD56+ cells had the greatest EBV DNA load.

### RNA samples

Blood samples were subjected to the Ficoll process to isolate peripheral mononuclear cells (PBMC) from the whole blood specimens within 2 hours after draw. Isolated PBMC samples were stored in Trizol solution in a −70 °C freezer until the RNA extraction was carried out using the RNeasy Mini Kit from Qiagen AB (Cat. # 74104, Sollentuna, German) following the manufacturer’s standard protocol including a DNase treatment. The quantities and quality of the RNA samples were assessed using a Thermo Nanodrop ND-2000 UV-Vis Spectrophotometer (Wilmington, DE, USA) and conventional agarose gel electrophoresis. All RNA samples had both 18S and 28S bands and were judged to be free of DNA and protein contamination based OD260:280 of 1.8 to 2.0.

### Microarray processing

A total of 17 RNA samples in the primary cohort were analyzed in the microarray study. The Affymetrix Human Transcriptome Array 2.0 (HTA2.0) was utilized for the whole transcriptome profiling analysis. This array can measure expression levels of 67,528 transcripts simultaneously, representing a comprehensive profiling tool for host gene expression. The microarray processing was completed at the Shanghai Gminix Biotech Inc. in Shanghai, China. The whole process followed Affymetrix recommended protocols using the Ambion Whole-Transcript (WT) RNA Amplification system and the GeneChip WT Terminal Labeling kit. In brief, the assays started with 100 ng of total RNA for cDNA synthesis and amplification and then proceeded to fragmentation and labeling reactions. The biotin-labeled cRNAs (5 µg) were hybridized onto the HTA2.0 arrays. After washing and staining, the arrays were scanned using a GeneChip 3000 7 G Scanner. All arrays passed Affymetrix recommended QC metrics (pos_vs_neg_auc > 0.8).

### Data analysis

The signal intensity data of the HTA2.0 arrays were processed through Affymetrix Expression Console (v1.4.1.46) with standard configuration for expression arrays, including robust multi-array average (RMA) background correction, median polish probe-level signal summarization, and quantile normalization. To corroborate the findings from our microarray data, we downloaded a sole and independent dataset from the public database at the National Center for Biotechnology Information (GEO accession: GSE45918)^13^. This dataset was generated with the Illumina Human Ref-8 v3.0 expression beadchips and used the same type of PBMC samples as used in our study. The signal data from the download were subjected to quantile normalization and then to a paired differential analysis across 8 subjects in the study. The differential analysis in both datasets were performed using the R package ‘limma’^38^, which is an R tool using linear model data fitting and empirical Bayesian statistics to determine the differential expression between groups and between pre- and post-disease pairs. A multiple test correction was applied using the Benjamini and Hochberg False Discovery Rate (FDR) method on a subset of data for known genes with Entrez Gene IDs and whose signals had a detection p-value < 0.05 for >50% of probe sets in at least 1 of the samples. This FDR-based adjusted p-value at 0.05 was considered to represent a significant difference. Due to potential fold-change compression issues with this type of HTA2.0 array^39^, we chose 1.5-fold as the cutoff for downstream interpretation. The transcriptome expression data from the present study have been deposited into the NCBI public database (GEO number: GSE85599).

### Pathway analysis

The whole transcriptome expression data obtained from the primary cohort were fed into the R package “GAGE” (generally applicable gene set and pathway enrichment analysis)^15^ for KEGG pathway enrichment analysis (Kyoto Encyclopedia of Genes and Genomes). The package used log2 fold-change (log2FC) values of all mapped transcripts in the databases and determined if a particular pathway was significantly up- or down-regulated from the whole transcriptome background. The FDR at 0.05 was set for significance of the enrichments.

### Quantitative PCR assay (qPCR)

500 ng aliquots of RNA samples from each subject were first reverse-transcribed to cDNA using the PrimeScript™ RT reagent Kit with genomic DNA Eraser (Perfect Real Time) from the Takara Bio Inc. (Kusatsu, Shiga, Japan). Then 50 ng of cDNAs was added in the real-time qPCR reaction. Primers for qPCR assay were purchased from the Sangon Biotech Company (Shanghai, China) and the sequences are available in Table S5 in Supplementary information. In the qPCR reaction, 10 µM each of forward and reverse primers were added, along with the SYBR® Premix Ex Taq™ (Tli RNaseH Plus) master mix (Takara Bio Inc., Japan). The assays were carried out in triplicate on a Roche LightCycler 480II real-time PCR instrument following the manufacturer’s protocols. All assays had a single amplification peak on the melting temperature curve with greater than 80% of PCR efficiency and less than 15% of coefficient of variation in triplicate reactions. Relative expression level was calculated using comparative Ct method as described previously^40^. The fold-change difference between EBV infection and control subjects were determined by the Mann-Whitney test, with a Bootstrap permutation-adjusted p-value < 0.05 for significance.

### Flow cytometry assay

Antibodies for cell markers were all from BD Biosceinces (San Jose in California, USA), except one for NKT cell marker (TCR V alpha 24J alpha18) that was from eBiosciences (a division of Thermo Fisher Scientific, Waltham in Massachusetts, USA). The antibodies involved in the flow cytometry studies were as following (clone-dye): anti-CD3 (UCHT1-FITC), anti-CD8 (RPA-T8-APC), anti-αβ-TCR (T10B9.1A-31-PE), anti-γδ-TCR (B1-BV421), anti-CD94 (HP-3D9-APC), anti-CD56 (B19-V450), anti-TCR V alpha 24J alpha18 (6B11-PE) (a specific marker for NKT cells), and anti-granzyme B (GB11-PE).

For T and NK cell activity study, freshly isolated PBMCs from study subjects were stimulated with phytohaemagglutinin (PHA) (15 µg/ml) after culture overnight at 10^6^ cells/ml in RPMI 1640 (Thermo Fisher Scientific/Gibco) containing 10% FBS (Gibco) and 600 IU/ml IL-2 (Peprotech, Rocky Hill in New Jersey, USA). In assays for cell surface markers, the PBMCs after 4 h stimulation with PHA were stained with the antibodies for CD3, CD8, CD56, and granzyme B. In assays for intracellular content of granzyme B, the PBMCs after 2 h stimulation with PHA were treated with BD GolgiStop™ protein transport inhibitor for additional 2 h, and then were stained with the antibodies for CD3, CD8, and CD56 for 20 min at 4 °C. After washing with PBS, the PBMCs were treated using the BD cell membrane poring reagent in Fixation/Permeabilization solution kit for 20 min at 4 °C, followed by granzyme B antibody staining for 20 min at 4 °C. Finally, the PBMCs were analyzed with FlowJo software (v7.6) for fluorescence of specific markers on the flow cytometer FACSCanto II from the BD Company (Franklin Lakes in New Jersey, USA).

For T and NK cell population analysis, an aliquot of freshly isolated PBMCs at 10^6^ cells/ml from study subjects were stained with the antibodies for CD3 and CD8 in combination with antibodies for αβ-TCR and γδ-TCR to determine the frequencies of αβ and γδ TCR-specific T cells. The other aliquot of the cells were stained with the antibodies for CD94 in conjunction with antibodies for CD3, CD56, and TCR V alpha 24J alpha18 to determine the abundance of CD94+ killer cells (T, NK, NKT cells). Cells were incubated for 30 min and followed by washing, and then analyzed with FlowJo software (v7.6) on the flow cytometer FACSCanto II (BD).

## Electronic supplementary material

Supplementary Information 
