# Supplementary material for: Whole transcriptome profiling reveals major cell types in the cellular immune response against acute and chronic active Epstein-Barr virus infection
Source: Sci Rep. 2017 Dec 19;7:17775. doi: 10.1038/s41598-017-18195-z (PMC5736708; doi:10.1038/s41598-017-18195-z)
Supplement: Supplementary file 1 — Supplementary Information [file 41598_2017_18195_MOESM1_ESM.pdf]

Supplementary Information

Whole transcriptome profiling reveals major cell types in the cellular immune response against acute and chronic active Epstein-Barr virus infection

Huaqing Zhong[1], Xinran Hu[2], Andrew B. Janowski[2], Gregory A. Storch[2], Liyun Su[1], Lingfeng Cao[1], Jinsheng Yu[3], and Jin Xu[1]

Department of Clinical Laboratory[1], Children's Hospital of Fudan University, Minhang District, Shanghai 201102, China; Departments of Pediatrics[2] and Genetics[3], Washington University School of Medicine, Saint Louis, Missouri 63110, United States.

Supplementary information includes the following:

1. Supplementary Figure S1: Fold-change and correlation data for hyperactive and hypoactive genes.
2. Supplementary Table S1: Clinical data and EBV lab results for 110 study subjects.
3. Supplementary Table S2: Differentially expressed genes between AIM vs. Healthy controls.
4. Supplementary Table S3: Differentially expressed genes between CAEBV vs. Healthy controls.
5. Supplementary Table S4: Fold-change data for 303 immune mediators.
6. Supplementary Table S5: Primers used in qPCR assays.

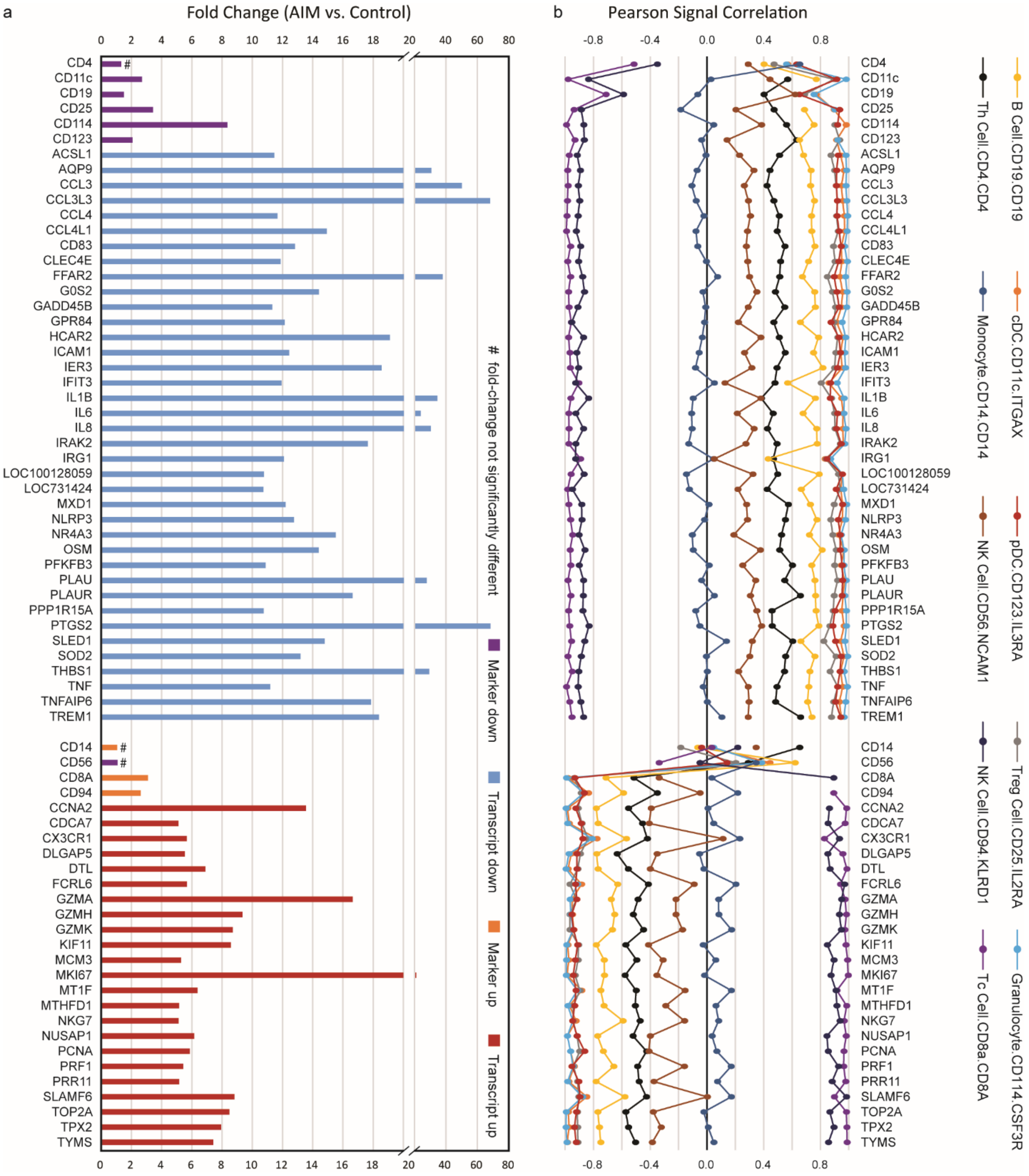

Supplementary Figure S1. Fold-change (a) and Pearson correlation data (b) for 10 cell markers and 61 hypoactive and hyperactive genes identified in subjects with acute EBV infection (AIM) in the primary cohort. Note: 23 up-regulated hyperactive genes were highly correlated positively with cytotoxic T cell (Tc) marker *CD8A* and NK cell marker *CD94* (*KLRD1*), and 38 down-regulated hypoactive genes were highly correlated positively with B cell, conventional dendritic cell (cDC), Treg and granulocyte markers.

Supplementary Table S1 -Clinical data and EBV lab results

Patient ID	Gender	Age	Group	Cohort	HTA2.0 microarray	qPCR assay	Flow cytometry assay	EBV DNA load in plasma (copies/ml blood)	EBV DNA load in white blood cells (copies/ml blood)	EBV VCA-IgM	EBV VCA-IgG	EBV-NA	Time at sampling from disease onset	Symtoms	Days hospitolized (total)	Days to recovery (outcome)
15VC1799	M	27 m	AIM	Primary	Yes	Yes		1.34E+04	n/a	(+)	(+)	(-)	5 d	fever, tonsil swelling, cervical lymphadenopathy	3	14
15VC1803	F	18 m	AIM	Primary	Yes	Yes		undetectable	3.58E+03	(+)	(+)	(-)	3 d	fever, cough, tonsil swelling, cervical lymphadenopathy	3	15
15VC2293	M	4 y	AIM	Primary	Yes	Yes		undetectable	2.38E+03	(+)	(+)	(-)	2 d	fever, cough, tonsil swelling, cervical lymphadenopathy, liver enlargement	3	15
15VC2386	F	3 y	AIM	Primary	Yes	Yes		1.03E+04	n/a	(+)	(+)	(-)	2 d	fever, sore throat, tonsil swelling, cervical lymphadenopathy, liver enlargement	8	20
ED483	M	24 m	AIM	Primary	Yes	Yes		undetectable	1.07E+05	(+)	(+)	(-)	2 d	fever, tonsil swelling, enlargement of liver & spleen	3	15
ED490	M	12 m	AIM	Primary	Yes	Yes		3.68E+03	5.13E+05	(+)	(+)	(-)	7 d	fever, sore throat, tonsil swelling, cervical lymphadenopathy, enlargement of liver & spleen	5	20
15VC2295	F	3 y	CAEBV	Primary	Yes	Yes		5.46E+05	n/a	(-)	(+)	(+)	2 m	fever, jaundice, tonsil swelling, cervical lymphadenopathy, enlargement of liver & spleen, ALT & AST elevated	45	hemophagocytic syndrome
15VC2407	M	9 y	CAEBV	Primary	Yes	Yes		1.46E+07	n/a	(-)	(+)	(+)	3 m	fever, cervical lymphadenopathy, enlargement of liver & spleen, ALT & AST elevated	17	disease continued, with recurrence of 2 times
15VC2414	M	15 y	CAEBV	Primary	Yes	Yes		3.44E+03	n/a	(-)	(+)	(+)	12 m	fever, tonsil swelling, splenectomy	79	disease continued, with recurrence of 2 times
15VC2656	F	2 y	CAEBV	Primary	Yes	Yes		7.70E+03	n/a	(-)	(+)	(+)	3 m	fever, enlargement of liver & spleen, ALT & AST elevated	13	disease continued, with recurrence of 2 times
15VC3454	M	12 y	CAEBV	Primary	Yes	Yes		3.60E+05	n/a	(-)	(+)	(+)	14 m	fever, skin impetigo, cervical lymphadenopathy, liver enlargement	27	disease continued, with recurrence of 3 times
C13	M	1 y	CTRL	Primary	Yes	Yes		undetectable	n/a	(-)	(+)	(-)				
C15	F	3 y	CTRL	Primary	Yes	Yes		undetectable	n/a	(-)	(+)	(+)				
C21	F	1 y	CTRL	Primary	Yes	Yes		undetectable	n/a	(-)	(+)	(-)				
C22	M	3 y	CTRL	Primary	Yes	Yes		undetectable	n/a	(-)	(+)	(+)				
C23	M	3 y	CTRL	Primary	Yes	Yes		undetectable	n/a	(-)	(+)	(+)				
C24	F	4 y	CTRL	Primary	Yes	Yes		undetectable	n/a	(-)	(+)	(+)				
16VC0100	F	4 y	AIM	Secondary		Yes		1.80E+03	1.55E+06	(+)	(+)	(-)	6 d	fever, palpebral edema, sore throat, skin rash, tonsil swelling, cervical lymphadenopathy	outpatient	23
16VC9468	F	9 y	AIM	Secondary		Yes		6.17E+03	n/a	(+)	(+)	(-)	7 d	fever, sore throat, tonsil swelling, cervical lymphadenopathy, ALT & AST elevated	4	27
16VC9539	F	3 y	AIM	Secondary		Yes		undetectable	2.01E+06	(+)	(+)	(-)	3 d	fever, palpebral edema, tonsil swelling, cervical lymphadenopathy	outpatient	29
16VC9546	M	1 y	AIM	Secondary		Yes		3.98E+03	n/a	(+)	(+)	(-)	4 d	fever, sore throat, skin rash, palpebral edema, tonsil swelling, cervical lymphadenopathy, enlargement of liver & spleen, ALT & AST elevated	outpatient	24
16VC9677	F	2 y	AIM	Secondary		Yes		undetectable	1.46E+05	(+)	(-)	(-)	4 d	fever, sore throat, tonsil swelling, cervical lymphadenopathy	outpatient	22
16VC9724	F	1 y	AIM	Secondary		Yes		undetectable	1.63E+04	(+)	(+)	(-)	3 d	fever, sore throat, skin rash, tonsil swelling, cervical lymphadenopathy,	outpatient	19
16VC9731	M	2 y	AIM	Secondary		Yes		2.88E+03	3.76E+06	(+)	(+)	(-)	4 d	fever, sore throat, cough, tonsil swelling	outpatient	19
16VC9784	M	4 y	AIM	Secondary		Yes		undetectable	5.51E+04	(+)	(+)	(-)	4 d	fever, palpebral edema, tonsil swelling, cervical lymphadenopathy, enlargement of liver & spleen, ALT & AST elevated	3	23
16VC9799	M	12 y	AIM	Secondary		Yes		undetectable	2.80E+04	(+)	(+)	(-)	10 d	fever, cough, sore throat, tonsil swelling, cervical lymphadenopathy	outpatient	30
16VC9842	M	3 y	AIM	Secondary		Yes		1.02E+03	2.30E+05	(+)	(+)	(-)	3 d	fever, palpebral edema, sore throat, tonsil swelling, cervical lymphadenopathy	3	13
ED482 *	F	5 y	AIM	Secondary		Yes		undetectable	1.07E+04	(+)	(+)	(-)	10 d	fever, cough, tonsil swelling, cervical lymphadenopathy, enlargement of liver & spleen	9	23
ED504 *	F	12 y	AIM	Secondary		Yes		undetectable	2.17E+04	(+)	(+)	(-)	3 d	fever, palpebral edema, tonsil swelling, cervical lymphadenopathy, ALT & AST elevated	5	19
IM-1(17ES171)	M	3 y	AIM	Secondary			cell surface	1.26E+03	3.53E+04	(+)	(+)	(-)	7 d	fever, tonsil swelling, cervical lymphadenopathy, ALT & AST elevated	outpatient	20
IM-10(17ES204)	F	1 y	AIM	Secondary			cell surface	undetectable	1.87E+03	(+)	(+)	(-)	3 d	fever, tonsil swelling, cervical lymphadenopathy, ALT & AST elevated	5	26
IM-11(17ES2247)	M	9 y	AIM	Secondary			intracellular	undetectable	5.91E+03	(+)	(+)	(-)	4 d	fever, cough, tonsil swelling, cervical lymphadenopathy, ALT & AST elevated	outpatient	23
IM-12(17ES2279)	M	3 y	AIM	Secondary			intracellular	1.57E+03	3.01E+04	(+)	(+)	(-)	4 d	fever, palpebral edema, tonsil swelling, cervical lymphadenopathy,	outpatient	25
IM-13(17ES2281)	M	7 y	AIM	Secondary			intracellular	7.24E+03	3.40E+05	(+)	(+)	(-)	2 d	fever, palpebral edema, tonsil swelling, sore throat, cervical lymphadenopathy,	3	18
IM-14(17ES3780)	M	2 y	AIM	Secondary			intracellular	2.39E+03	4.50E+05	(+)	(+)	(-)	3 d	fever, palpebral edema, tonsil swelling, cervical lymphadenopathy, palpebral edema	3	17
IM-15(17ES3929)	F	3 y	AIM	Secondary			intracellular	6.06E+03	1.60E+06	(+)	(+)	(-)	2 d	fever, sore throat, cough, tonsil swelling, cervical lymphadenopathy, ALT & AST elevated	outpatient	23
IM-16(17ES3979)	M	2 y	AIM	Secondary			intracellular	undetectable	1.30E+05	(+)	(+)	(-)	4 d	fever, sore throat, tonsil swelling, cervical lymphadenopathy	outpatient	24
IM-17(17ES3998)	M	2 y	AIM	Secondary			intracellular	undetectable	3.64E+04	(+)	(+)	(-)	3 d	fever, sore throat, tonsil swelling, cervical lymphadenopathy	3	27
IM-18(17ES4051)	M	3 y	AIM	Secondary			intracellular	undetectable	9.29E+03	(+)	(+)	(-)	4 d	fever, palpebral edema, cough, tonsil swelling, cervical lymphadenopathy, enlargement of liver & spleen, ALT & AST elevated	4	22
IM-2(17ES182)	M	2 y	AIM	Secondary			cell surface	undetectable	undetectable	(+)	(-)	(-)	2 d	fever, skin rash, cough, cervical lymphadenopathy	3	10
IM-3(17ES183)	M	7 y	AIM	Secondary			cell surface	undetectable	3.39E+03	(+)	(+)	(-)	2 d	fever, palpebral edema, tonsil swelling, cervical lymphadenopathy, ALT & AST elevated	3	22
IM-4(17ES310)	M	10 y	AIM	Secondary			cell surface	undetectable	3.20E+04	(+)	(+)	(-)	3 d	fever, palpebral edema, tonsil swelling, cervical lymphadenopathy	7	18
IM-5(17ES322)	M	4 y	AIM	Secondary			cell surface	undetectable	1.20E+03	(+)	(+)	(-)	5 d	fever, cough, tonsil swelling	6	34
IM-6(17ES509)	M	2 y	AIM	Secondary			cell surface	undetectable	4.21E+05	(+)	(-)	(-)	6 d	fever, cough, tonsil swelling	outpatient	24
IM-7(17ES618)	M	7 y	AIM	Secondary			cell surface	undetectable	1.19E+03	(+)	(-)	(-)	3 d	fever, tonsil swelling, cervical lymphadenopathy, ALT & AST elevated	3	16
IM-8(17ES619)	M	4 y	AIM	Secondary			cell surface	undetectable	2.01E+03	(+)	(+)	(-)	4 d	fever, sore throat, cough	3	26
IM-9(17ES1904)	M	1 y	AIM	Secondary			cell surface	1.54E+04	9.62E+05	(+)	(-)	(-)	3 d	fever, tonsil swelling, cervical lymphadenopathy	3	14
IM41	F	5Y	AIM	Secondary			population	undetectable	2.40E+03	(+)	(-)	(-)	3d	fever, cough, tonsil swelling	outpatient	10
IM42	F	2Y	AIM	Secondary			population	undetectable	1.65E+05	(+)	(+)	(-)	4 d	fever, tonsil swelling, cervical lymphadenopathy , palpebral edema	outpatient	15
IM43	M	2Y	AIM	Secondary			population	4.70E+03	2.00E+05	(+)	(+)	(-)	5d	fever, tonsil swelling, cervical lymphadenopathy , palpebral edema	3	18

Patient ID	Gender	Age	Group	Cohort	HTA2.0 microarray	qPCR assay	Flow cytometry assay	EBV DNA load in plasma (copies/ml blood)	EBV DNA load in white blood cells (copies/ml blood)	EBV VCA-IgM	EBV VCA-IgG	EBV-NA	Time at sampling from disease onset	Symtoms	Days hospitolized (total)	Days to recovery (outcome)
IM44	F	3Y	AIM	Secondary			population	2.34E+03	3.91E+05	(+)	(+)	(-)	5d	fever, cough, tonsil swelling, skin rash, cervical lymphadenopathy，palpebral edema,enlargement of liver，ALT & AST elevated	3	26
IM45	M	2Y	AIM	Secondary			population	1.63E+03	6.63E+05	(+)	(+)	(-)	11d	fever, cough, tonsil swelling, cervical lymphadenopathy，palpebral edema,enlargement of liver & spleen，ALT & AST elevated	3	29
IM46	F	5Y	AIM	Secondary			population	undetectable	1.78E+04	(+)	(+)	(-)	9d	fever, cough, tonsil swelling, cervical lymphadenopathy，palpebral edema,enlargement of liver & spleen，ALT & AST elevated	4	27
IM47	M	4Y	AIM	Secondary			population	undetectable	1.89E+05	(+)	(+)	(-)	6d	fever, cough, tonsil swelling, cervical lymphadenopathy，palpebral edema,enlargement of liver & spleen，ALT & AST elevated	6	35
IM48	M	2Y	AIM	Secondary			population	4.83E+04	n/a	(+)	(-)	(-)	2d	fever, tonsil swelling, cervical lymphadenopathy，palpebral edema,enlargement of liver，ALT & AST elevated	outpatient	18
IM49	M	2Y	AIM	Secondary			population	undetectable	3.68E+04	(+)	(+)	(-)	5d	fever, tonsil swelling, cervical lymphadenopathy，palpebral edema	outpatient	10
IM50	M	3Y	AIM	Secondary			population	3.82E+03	4.48E+05	(+)	(-)	(-)	9d	fever, tonsil swelling, cervical lymphadenopathy，palpebral edema,enlargement of liver，ALT & AST elevated	5	26
ED570 *	F	10 y	CAEBV	Secondary		Yes		6.33E+03	n/a	(-)	(+)	(+)	8 m	fever, cough, cervical lymphadenopathy, enlargement of liver & spleen, ALT & AST elevated	63	dead
ED577 *	M	5 y	CAEBV	Secondary		Yes		5.32E+04	n/a	(-)	(+)	(+)	9 m	fever, convulsion, skin impetigo, cervical lymphadenopathy, enlargement of liver & spleen, ALT & AST elevated	34	dead
ED578 *	F	3 y	CAEBV	Secondary		Yes		5.84E+03	n/a	(-)	(+)	(+)	2.5 y	fever, cough, vasculitis, cervical lymphadenopathy, enlargement of liver & spleen, ALT & AST elevated	55	disease continued, with recurrence of 3 times
ED580 *	F	3 y	CAEBV	Secondary		Yes		2.02E+05	n/a	(-)	(+)	(-)	1.5 y	fever, cough, cervical lymphadenopathy, enlargement of liver & spleen, ALT & AST elevated	36	disease continued, with recurrence of 3 times
12c9 *	M	10 y	CTRL	Secondary		Yes		undetectable	n/a	(-)	(-)	(+)				
C11 *	F	1 y	CTRL	Secondary		Yes		undetectable	n/a	(-)	(-)	(-)				
C12 *	F	2 y	CTRL	Secondary		Yes		undetectable	n/a	(-)	(-)	(-)				
C14 *	F	3 y	CTRL	Secondary		Yes		undetectable	n/a	(-)	(-)	(-)				
C51	M	3 y	CTRL	Secondary		Yes		undetectable	n/a	(-)	(+)	(+)				
C52	M	6 y	CTRL	Secondary		Yes		undetectable	n/a	(-)	(+)	(+)				
C53	F	4 y	CTRL	Secondary		Yes		undetectable	n/a	(-)	(+)	(+)				
C54	F	4 y	CTRL	Secondary		Yes		undetectable	n/a	(-)	(+)	(+)				
C55	M	6 y	CTRL	Secondary		Yes		undetectable	n/a	(-)	(+)	(+)				
C56	M	3 y	CTRL	Secondary		Yes		undetectable	n/a	(-)	(+)	(+)				
C57	F	4 y	CTRL	Secondary		Yes		undetectable	n/a	(-)	(+)	(+)				
C58	M	7 y	CTRL	Secondary		Yes		undetectable	n/a	(-)	(+)	(+)				
C59	M	3 y	CTRL	Secondary		Yes		undetectable	n/a	(-)	(+)	(+)				
C60	M	11 y	CTRL	Secondary		Yes		undetectable	n/a	(-)	(+)	(+)				
H1	M	4 y	CTRL	Secondary			surface activity	undetectable	n/a	(-)	(+)	(+)				
H2	M	9 y	CTRL	Secondary			surface activity	undetectable	n/a	(-)	(+)	(+)				
H3	F	4 y	CTRL	Secondary			surface activity	undetectable	n/a	(-)	(+)	(+)				
H4	M	4 y	CTRL	Secondary			surface activity	undetectable	n/a	(-)	(+)	(+)				
H5	F	7 y	CTRL	Secondary			surface activity	undetectable	n/a	(-)	(+)	(+)				
H6	M	6 y	CTRL	Secondary			surface activity	undetectable	n/a	(-)	(+)	(+)				
H7	M	8 y	CTRL	Secondary			surface activity	undetectable	n/a	(-)	(+)	(+)				
H8	F	4 y	CTRL	Secondary			surface activity	undetectable	n/a	(-)	(+)	(+)				
H9	M	5 y	CTRL	Secondary			surface activity	undetectable	n/a	(-)	(+)	(+)				
H10	M	5 y	CTRL	Secondary			surface activity	undetectable	n/a	(-)	(+)	(+)				
H11	M	3 y	CTRL	Secondary			surface activity	undetectable	n/a	(-)	(+)	(+)				
H12	F	1 y	CTRL	Secondary			surface activity	undetectable	n/a	(-)	(+)	(+)				
H13	F	5 y	CTRL	Secondary			surface activity	undetectable	n/a	(-)	(+)	(+)				
H14	M	4 y	CTRL	Secondary			surface activity	undetectable	n/a	(-)	(+)	(+)				
H15	M	9 y	CTRL	Secondary			surface activity	undetectable	n/a	(-)	(+)	(+)				
H16	M	5 y	CTRL	Secondary			intracell activity	undetectable	n/a	(-)	(+)	(+)				
H17	F	4 y	CTRL	Secondary			intracell activity	undetectable	n/a	(-)	(+)	(+)				
H18	F	6 y	CTRL	Secondary			intracell activity	undetectable	n/a	(-)	(+)	(+)				
H19	F	4 y	CTRL	Secondary			intracell activity	undetectable	n/a	(-)	(+)	(+)				
H20	F	4 y	CTRL	Secondary			intracell activity	undetectable	n/a	(-)	(+)	(+)				
H21	F	5 y	CTRL	Secondary			intracell activity	undetectable	n/a	(-)	(+)	(+)				
H22	M	6 y	CTRL	Secondary			intracell activity	undetectable	n/a	(-)	(+)	(+)				
H23	M	3 y	CTRL	Secondary			intracell activity	undetectable	n/a	(-)	(+)	(+)				
H24	M	8 y	CTRL	Secondary			intracell activity	undetectable	n/a	(-)	(+)	(+)				
H25	F	5 y	CTRL	Secondary			intracell activity	undetectable	n/a	(-)	(+)	(+)				
H41	M	8Y	CTRL	Secondary			population	undetectable	n/a	(-)	(+)	(+)				
H42	M	3Y	CTRL	Secondary			population	undetectable	n/a	(-)	(+)	(+)				
H43	M	2Y	CTRL	Secondary			population	undetectable	n/a	(-)	(-)	(-)				
H44	M	10Y	CTRL	Secondary			population	undetectable	n/a	(-)	(+)	(+)				
H45	F	1Y	CTRL	Secondary			population	undetectable	n/a	(-)	(-)	(-)				
H46	F	5Y	CTRL	Secondary			population	undetectable	n/a	(-)	(+)	(+)				
H47	M	3Y	CTRL	Secondary			population	undetectable	n/a	(-)	(+)	(+)				
H48	F	1Y	CTRL	Secondary			population	undetectable	n/a	(-)	(-)	(-)				
H49	F	8Y	CTRL	Secondary			population	undetectable	n/a	(-)	(+)	(+)				
H50	F	6Y	CTRL	Secondary			population	undetectable	n/a	(-)	(+)	(+)				

Note: * also in the "primary+" cohort; AIM -acute infectious mononucleosis; CAEBV -chronic active EBV infection; CTRL -healthy control; n/a -did not test; undetectable -less than 1,000 copies/ml blood in PCR lab test.

Supplementary Table S2 -Differential genes AIM vs. CTRL

transcript cluster id	Gene Symbol	Entrez Gene ID	Description	FoldChange (AIM/control)	P.Value (AIM/control)	adj.P.Val (AIM/control)
TC10001756.hg.1	MKI67	4288	antigen identified by monoclonal antibody Ki-67	21.188458	6.01E-10	1.41553E-07
TC05000231.hg.1	GZMA	3001	granzyme A (granzyme 1, cytotoxic T-lymphocyte-associated serine esterase 3)	16.644227	6.01E-08	2.93162E-06
TC04001516.hg.1	CCNA2	890	cyclin A2	13.557397	5.91E-12	1.49591E-08
TC06001353.hg.1	HIST1H3F	8968	histone cluster 1, H3f	9.646229	2.37E-11	2.77365E-08
TC14000981.hg.1	GZMH	2999	granzyme H (cathepsin G-like 2, protein h-CCPX)	9.358658	8.31E-08	3.69284E-06
TC01003403.hg.1	SLAMF6	114836	SLAM family member 6	8.820681	7.04E-06	0.000100362
TC05000227.hg.1	GZMK	3003	granzyme K (granzyme 3; tryptase II)	8.705819	4.56E-05	0.000429543
TC06001427.hg.1	HIST1H3F	8968	histone cluster 1, H3f	8.614766	4.13E-09	4.99537E-07
TC10000659.hg.1	KIF11	3832	kinesin family member 11	8.573475	3.24633E-10	1.05495E-07
TC17001462.hg.1	TOP2A	7153	topoisomerase (DNA) II alpha 170kDa	8.482522	5.84136E-10	1.41553E-07
TC20000198.hg.1	TPX2	22974	TPX2, microtubule-associated, homolog (Xenopus laevis)	7.940327	2.16538E-14	6.5188E-10
TC06001343.hg.1	HIST1H2AB	8335	histone cluster 1, H2ab	7.49441	8.13833E-11	5.68442E-08
TC18000006.hg.1	TYMS	7298	thymidylate synthetase	7.426067	1.33179E-11	1.90959E-08
TC01001767.hg.1	DTL	51514	denticleless E3 ubiquitin protein ligase homolog (Drosophila)	6.897324	1.10462E-11	1.78611E-08
TC06001348.hg.1	HIST1H4D	8360	histone cluster 1, H4d	6.891852	1.04455E-10	6.18368E-08
TC06000265.hg.1	HIST1H2BM	8342	histone cluster 1, H2bm	6.702573	3.36413E-08	1.91788E-06
TC16000473.hg.1	MT1F	4494	metallothionein 1F	6.37933	0.00199369	0.009178335
TC06001342.hg.1	HIST1H3B	8358	histone cluster 1, H3b	6.250594	2.30951E-10	8.65508E-08
TC15000306.hg.1	NUSAP1	51203	nucleolar and spindle associated protein 1	6.150882	2.72627E-10	9.53515E-08
TC20000592.hg.1	PCNA	5111	proliferating cell nuclear antigen	5.864061	7.56217E-08	3.4311E-06
TC01001356.hg.1	FCRL6	343413	Fc receptor-like 6	5.682418	1.76915E-06	3.46399E-05
TC03001304.hg.1	CX3CR1	1524	chemokine (C-X3-C motif) receptor 1	5.664957	1.99115E-05	0.000223728
TC06001429.hg.1	HIST1H3J	8356	histone cluster 1, H3j	5.648985	2.13306E-10	8.38535E-08
TC14001155.hg.1	DLGAP5	9787	discs, large (Drosophila) homolog-associated protein 5	5.529951	1.85935E-10	8.38535E-08
TC06000173.hg.1	HIST1H2AE	3012	histone cluster 1, H2ae	5.43581	2.65434E-10	9.53515E-08
TC10001366.hg.1	PRF1	5551	perforin 1 (pore forming protein)	5.416991	6.78532E-07	1.66363E-05
TC06001799.hg.1	MCM3	4172	minichromosome maintenance complex component 3	5.291469	1.11401E-11	1.78611E-08
TC17000718.hg.1	PRR11	55771	proline rich 11	5.163019	8.87549E-12	1.75568E-08
TC14000388.hg.1	MTHFD1	4522	methylenetetrahydrofolate dehydrogenase (NADP+ dependent) 1, methenyltetrahydrofo	5.155253	1.37197E-09	2.49778E-07
TC19001776.hg.1	NKG7	4818	natural killer cell group 7 sequence	5.11404	1.81433E-06	3.52669E-05
TC02001035.hg.1	CDCA7	83879	cell division cycle associated 7	5.108407	4.96883E-11	4.36035E-08
TC16000258.hg.1	PLK1	5347	polo-like kinase 1	4.996582	1.36269E-12	9.67172E-09
TC12001614.hg.1	PRIM1	5557	primase, DNA, polypeptide 1 (49kDa)	4.87546	5.0631E-10	1.38958E-07
TC06000178.hg.1	HIST1H2BI	8346	histone cluster 1, H2bi	4.842443	3.14802E-09	4.25335E-07
TC12000082.hg.1	NCAPD2	9918	non-SMC condensin I complex, subunit D2	4.821426	7.92491E-12	1.69617E-08
TC17000768.hg.1	DCAF7	10238	DDB1 and CUL4 associated factor 7	4.628961	3.11349E-09	4.24315E-07
TC01001347.hg.1	PYHIN1	149628	pyrin and HIN domain family, member 1	4.575719	2.51126E-08	1.57051E-06
TC12001106.hg.1	FOXM1	2305	forkhead box M1	4.556623	1.96587E-10	8.38535E-08
TC22001427.hg.1	APOBEC3D	140564	apolipoprotein B mRNA editing enzyme, catalytic polypeptide-like 3D	4.517334	1.72319E-08	1.2193E-06
TC02002376.hg.1	MCM6	4175	minichromosome maintenance complex component 6	4.496285	3.63323E-08	2.05812E-06
TC06000171.hg.1	HIST1H2BF	8343	histone cluster 1, H2bf	4.485664	5.27765E-11	4.36035E-08
TC15000337.hg.1	WDR76	79968	WD repeat domain 76	4.467414	2.56092E-12	1.042E-08
TC04000145.hg.1	CD38	952	CD38 molecule	4.462163	2.0584E-07	7.17944E-06
TC15001841.hg.1	IDH2	3418	isocitrate dehydrogenase 2 (NADP+), mitochondrial	4.458052	2.09073E-11	2.61127E-08
TC11002307.hg.1	USP28	57646	ubiquitin specific peptidase 28	4.410921	1.42358E-08	1.08124E-06
TC01001791.hg.1	CENPF	1063	centromere protein F, 350/400kDa	4.356365	3.28405E-10	1.06469E-07
TC02000057.hg.1	RRM2	6241	ribonucleotide reductase M2	4.345308	8.48221E-09	7.85664E-07
TC15000391.hg.1	SLC27A2	11001	solute carrier family 27 (fatty acid transporter), member 2	4.311809	3.10035E-07	9.53622E-06
TC06000270.hg.1	HIST1H2BO	8348	histone cluster 1, H2bo	4.243893	1.26732E-10	6.91701E-08
TC20000555.hg.1	SIRPG	55423	signal-regulatory protein gamma	4.221079	3.61215E-07	1.06369E-05
TC01002500.hg.1	CLSPN	63967	claspin	4.203497	1.38491E-09	2.51523E-07
TC01003194.hg.1	HIST2H2AB	317772	histone cluster 2, H2ab	4.179914	1.94114E-11	2.55107E-08
TC09000526.hg.1	SMC2	10592	structural maintenance of chromosomes 2	4.172566	7.7942E-09	7.54036E-07
TC11001724.hg.1	CKAP5	9793	cytoskeleton associated protein 5	4.129971	1.29376E-09	2.40406E-07
TC08000346.hg.1	MCM4	4173	minichromosome maintenance complex component 4	4.128249	2.12721E-10	8.38535E-08
TC18000506.hg.1	ACAA2	10449	acetyl-CoA acyltransferase 2	4.119088	2.51594E-09	3.7632E-07
TC22000260.hg.1	MCM5	4174	minichromosome maintenance complex component 5	4.106715	4.09422E-11	4.29252E-08
TC01003662.hg.1	ASPM	259266	asp (abnormal spindle) homolog, microcephaly associated (Drosophila)	4.069985	1.51683E-09	2.73441E-07
TC07001004.hg.1	GIMAP4	55303	GTPase, IMAP family member 4	4.015487	8.29475E-07	1.94086E-05
TC06001387.hg.1	HIST1H2BK	85236	histone cluster 1, H2bk	3.919741	9.82896E-08	4.22075E-06
TC02000582.hg.1	NCAPH	23397	non-SMC condensin I complex, subunit H	3.915175	3.94118E-10	1.20001E-07
TC11000572.hg.1	RARRES3	5920	retinoic acid receptor responder (tazarotene induced) 3	3.912765	2.14282E-10	8.38535E-08
TC17001745.hg.1	SKA2	348235	spindle and kinetochore associated complex subunit 2	3.912358	1.05E-10	6.18368E-08
TC04002941.hg.1	FGFBP2	83888	fibroblast growth factor binding protein 2	3.873851	2.24E-05	0.000245975
TC11001061.hg.1	CD3G	917	CD3g molecule, gamma (CD3-TCR complex)	3.86354	3.82E-04	0.002401401
TC05001947.hg.1	ANXA6	309	annexin A6	3.762009	1.15E-05	0.000146135
TC11000090.hg.1	RRM1	6240	ribonucleotide reductase M1	3.726135	2.59E-09	3.79989E-07
TC12000504.hg.1	ESYT1	23344	extended synaptotagmin-like protein 1	3.720436	1.31487E-06	2.74025E-05
TC06000166.hg.1	HIST1H4C	8364	histone cluster 1, H4c	3.695796	1.04363E-08	8.85781E-07
TC04000155.hg.1	NCAPG	64151	non-SMC condensin I complex, subunit G	3.650266	5.60593E-08	2.81213E-06

transcript cluster id	Gene Symbol	Entrez Gene ID	Description	FoldChange (AIM/control)	P.Value (AIM/control)	adj.P.Val (AIM/control)
TC11002347.hg.1	CD3D	915	CD3d molecule, delta (CD3-TCR complex)	3.621887	0.000275509	0.00183775
TC06000176.hg.1	HIST1H2BH	8345	histone cluster 1, H2bh	3.621314	5.6779E-10	1.41553E-07
TC12000080.hg.1	CD27	939	CD27 molecule	3.6186	5.87935E-05	0.000529248
TC12001633.hg.1	CDK4	1019	cyclin-dependent kinase 4	3.614773	2.55367E-09	3.79989E-07
TC06001424.hg.1	HIST2H4B	554313	histone cluster 2, H4b	3.609853	2.68723E-10	9.53515E-08
TC15000846.hg.1	FANCI	55215	Fanconi anemia, complementation group I	3.598935	5.13249E-10	1.39356E-07
TC20000063.hg.1	MCM8	84515	minichromosome maintenance complex component 8	3.579517	2.88318E-10	1.00884E-07
TC15000638.hg.1	KIF23	9493	kinesin family member 23	3.533428	1.06271E-09	2.09754E-07
TC01002981.hg.1	DENND2D	79961	DENN/MADD domain containing 2D	3.521496	1.72735E-07	6.36264E-06
TC18000009.hg.1	NDC80	10403	NDC80 kinetochore complex component	3.510318	4.23445E-08	2.32479E-06
TC06001428.hg.1	HIST1H4L	8368	histone cluster 1, H4l	3.506269	1.09239E-10	6.29E-08
TC02002060.hg.1	CD8B	926	CD8b molecule	3.485317	1.58541E-08	1.15945E-06
TC17000016.hg.1	RPA1	6117	replication protein A1, 70kDa	3.456983	4.78713E-09	5.46811E-07
TC02002204.hg.1	BUB1	699	BUB1 mitotic checkpoint serine/threonine kinase	3.39531	4.82071E-11	4.36035E-08
TC16000493.hg.1	GPR56	9289	G protein-coupled receptor 56	3.393016	1.98005E-07	7.00737E-06
TC06001684.hg.1	CCDC167	154467	coiled-coil domain containing 167	3.389854	8.38406E-09	7.84797E-07
TC16000468.hg.1	MT1E	4493	metallothionein 1E	3.385389	0.01358694	0.04260257
TC15001449.hg.1	LEO1	123169	Leo1, Paf1/RNA polymerase II complex component, homolog (S. cerevisiae)	3.377526	8.91012E-08	3.96212E-06
TC14000968.hg.1	PSME2	5721	proteasome (prosome, macropain) activator subunit 2 (PA28 beta)	3.370965	5.13436E-08	2.65996E-06
TC06001386.hg.1	HIST1H2BJ	8970	histone cluster 1, H2bj	3.339071	2.00986E-07	7.08266E-06
TC07001294.hg.1	TARP	445347	TCR gamma alternate reading frame protein	3.321957	9.58103E-07	2.15849E-05
TC02002504.hg.1	STK39	27347	serine threonine kinase 39	3.30859	2.75836E-07	8.8285E-06
TC03000873.hg.1	SMC4	10051	structural maintenance of chromosomes 4	3.287004	4.63426E-08	2.47386E-06
TC06000347.hg.1	TUBB	203068	tubulin, beta class I	3.281761	3.64454E-10	1.14126E-07
TC16001154.hg.1	CNOT1	23019	CCR4-NOT transcription complex, subunit 1	3.276711	1.44235E-07	5.56378E-06
TC11001365.hg.1	GVINP1	387751	GTPase, very large interferon inducible pseudogene 1	3.276688	0.00092977	0.005000388
TC16000099.hg.1	IL32	9235	interleukin 32	3.274913	1.04778E-07	4.4186E-06
TC01000426.hg.1	LCK	3932	lymphocyte-specific protein tyrosine kinase	3.266551	1.21263E-09	2.34402E-07
TC11002009.hg.1	CPT1A	1374	carnitine palmitoyltransferase 1A (liver)	3.266117	1.91997E-07	6.89442E-06
TC0X001064.hg.1	SMC1A	8243	structural maintenance of chromosomes 1A	3.257093	1.35621E-05	0.000166416
TC12000138.hg.1	KLRG1	10219	killer cell lectin-like receptor subfamily G, member 1	3.244224	9.14461E-07	2.09155E-05
TC04001741.hg.1	HMGB2	3148	high mobility group box 2	3.241392	2.14224E-08	1.41815E-06
TC08001191.hg.1	PRKDC	5591	protein kinase, DNA-activated, catalytic polypeptide	3.229554	5.11304E-06	7.81442E-05
TC12001566.hg.1	CBX5	23468	chromobox homolog 5	3.227402	2.00653E-10	8.38535E-08
TC06000223.hg.1	HIST1H2AG	8969	histone cluster 1, H2ag	3.223277	6.33446E-10	1.47976E-07
TC10001702.hg.1	PRDX3	10935	peroxiredoxin 3	3.216853	5.20475E-08	2.67694E-06
TC19001159.hg.1	DNMT1	1786	DNA (cytosine-5-)-methyltransferase 1	3.198946	7.54557E-10	1.65572E-07
TC07001654.hg.1	MCM7	4176	minichromosome maintenance complex component 7	3.190745	1.62775E-07	6.08835E-06
TC12001222.hg.1	KLRC4	8302	killer cell lectin-like receptor subfamily C, member 4	3.189216	2.36986E-05	0.000257211
TC15000449.hg.1	CCNB2	9133	cyclin B2	3.166312	9.0436E-09	8.14718E-07
TC01001175.hg.1	HIST2H3A	333932	histone cluster 2, H3a	3.165405	1.99493E-08	1.35559E-06
TC11001870.hg.1	MTA2	9219	metastasis associated 1 family, member 2	3.160607	2.50708E-11	2.92766E-08
TC22001426.hg.1	APOBEC3C	27350	apolipoprotein B mRNA editing enzyme, catalytic polypeptide-like 3C	3.157928	4.437E-09	5.26348E-07
TC09000357.hg.1	CEP78	84131	centrosomal protein 78kDa	3.134239	2.24703E-07	7.66471E-06
TC03001903.hg.1	GPR171	29909	G protein-coupled receptor 171	3.132331	8.48766E-05	0.000706894
TC10000721.hg.1	SCD	6319	stearoyl-CoA desaturase (delta-9-desaturase)	3.129506	1.12785E-11	1.78611E-08
TC17001378.hg.1	CCL5	6352	chemokine (C-C motif) ligand 5	3.118005	5.36111E-08	2.74168E-06
TC01003189.hg.1	HIST2H3A	333932	histone cluster 2, H3a	3.103946	2.29753E-08	1.49009E-06
TC14000982.hg.1	GZMB	3002	granzyme B (granzyme 2, cytotoxic T-lymphocyte-associated serine esterase 1)	3.103426	1.59428E-05	0.000189014
TC06002103.hg.1	SAMD3	154075	sterile alpha motif domain containing 3	3.097967	4.32833E-08	2.3496E-06
TC10000813.hg.1	ACSL5	51703	acyl-CoA synthetase long-chain family member 5	3.097902	4.17947E-07	1.18931E-05
TC11001788.hg.1	SSRP1	6749	structure specific recognition protein 1	3.093789	1.28942E-08	1.02726E-06
TC16001158.hg.1	GOT2	2806	glutamic-oxaloacetic transaminase 2, mitochondrial	3.088251	9.51277E-09	8.39153E-07
TC02002059.hg.1	CD8A	925	CD8a molecule	3.087516	1.75027E-08	1.23997E-06
TC01001688.hg.1	ATP2B4	493	ATPase, Ca++ transporting, plasma membrane 4	3.079683	8.5327E-05	0.000708834
TC01002864.hg.1	TGFBR3	7049	transforming growth factor, beta receptor III	3.075558	1.00449E-05	0.000131614
TC06004063.hg.1	HIST1H2AI	8329	histone cluster 1, H2ai	3.068191	1.55122E-09	2.80936E-07
TC01000427.hg.1	HDAC1	3065	histone deacetylase 1	3.058699	2.41616E-09	3.71801E-07
TC01003801.hg.1	INTS7	25896	integrator complex subunit 7	3.05693	2.10179E-10	8.38535E-08
TC01000575.hg.1	NASP	4678	nuclear autoantigenic sperm protein (histone-binding)	3.041715	2.80287E-07	8.9165E-06
TC15002807.hg.1	KIAA0101	9768	KIAA0101	3.039	4.85037E-07	1.31538E-05
TC12001184.hg.1	M6PR	4074	mannose-6-phosphate receptor (cation dependent)	3.034404	3.31846E-08	1.91788E-06
TC14000568.hg.1	CPSF2	53981	cleavage and polyadenylation specific factor 2, 100kDa	3.032729	4.13032E-09	5.07262E-07
TC08000213.hg.1	ESCO2	157570	establishment of sister chromatid cohesion N-acetyltransferase 2	3.002016	1.9652E-10	8.38535E-08
TC12000467.hg.1	NCKAP1L	3071	NCK-associated protein 1-like	2.969678	1.73313E-09	2.97728E-07
TC11002175.hg.1	CTSC	1075	cathepsin C	2.967799	2.48193E-10	9.43525E-08
TC20000164.hg.1	CST7	8530	cystatin F (leukocystatin)	2.961418	3.94563E-06	6.39928E-05
TC05000202.hg.1	NNT	23530	nicotinamide nucleotide transhydrogenase	2.961165	7.43209E-08	3.42639E-06
TC18000461.hg.1	TPGS2	25941	tubulin polyglutamylase complex subunit 2	2.940432	1.48888E-08	1.10658E-06
TC17001520.hg.1	ACLY	47	ATP citrate lyase	2.939213	1.96957E-10	8.38535E-08
TC12001296.hg.1	RECQL	5965	RecQ protein-like (DNA helicase Q1-like)	2.930368	3.85138E-08	2.15417E-06
TC10000098.hg.1	MCM10	55388	minichromosome maintenance complex component 10	2.918944	5.11298E-11	4.36035E-08

transcript cluster id	Gene Symbol	Entrez Gene ID	Description	FoldChange (AIM/control)	P.Value (AIM/control)	adj.P.Val (AIM/control)
TC19002698.hg.1	ECH1	1891	enoyl CoA hydratase 1, peroxisomal	2.915281	2.83287E-07	8.9846E-06
TC0X000120.hg.1	POLA1	5422	polymerase (DNA directed), alpha 1, catalytic subunit	2.913759	1.82535E-07	6.65905E-06
TC15000606.hg.1	PTPLAD1	51495	protein tyrosine phosphatase-like A domain containing 1	2.909585	5.60171E-05	0.000509296
TC11001812.hg.1	LPXN	9404	leupaxin	2.887338	1.08354E-06	2.38164E-05
TC05001481.hg.1	ENC1	8507	ectodermal-neural cortex 1 (with BTB domain)	2.877146	0.000297362	0.001960052
TC12001392.hg.1	ABCD2	225	ATP-binding cassette, sub-family D (ALD), member 2	2.875837	3.88386E-05	0.000379301
TC11002473.hg.1	NCAPD3	23310	non-SMC condensin II complex, subunit D3	2.870662	5.57997E-10	1.41553E-07
TC06000225.hg.1	HIST1H2AH	85235	histone cluster 1, H2ah	2.867122	1.93398E-10	8.38535E-08
TC06000168.hg.1	HIST1H1E	3008	histone cluster 1, H1e	2.855741	1.82389E-06	3.55753E-05
TC19000672.hg.1	SAE1	10055	SUMO1 activating enzyme subunit 1	2.850543	1.05635E-08	8.93452E-07
TC12001470.hg.1	TUBA1B	10376	tubulin, alpha 1b	2.842516	7.80269E-08	3.55344E-06
TC11003499.hg.1	COX8A	1351	cytochrome c oxidase subunit VIIIA (ubiquitous)	2.834128	6.42281E-06	9.44313E-05
TC06000163.hg.1	HIST1H4A	8359	histone cluster 1, H4a	2.820355	3.71755E-08	2.10955E-06
TC19001501.hg.1	MAP4K1	11184	mitogen-activated protein kinase kinase kinase kinase 1	2.817164	3.6892E-07	1.07744E-05
TC01006300.hg.1	STIL	6491	SCL/TAL1 interrupting locus	2.808189	1.38959E-08	1.07713E-06
TC16002068.hg.1	SPN	6693	sialophorin	2.80612	5.32115E-08	2.73807E-06
TC15000614.hg.1	ZWILCH	55055	zwilch kinetochore protein	2.789648	1.09663E-08	9.21914E-07
TC12000163.hg.1	LOC101060038	101060038	uncharacterized LOC101060038	2.778173	8.4316E-06	0.000115904
TC20000320.hg.1	MYBL2	4605	v-myb myeloblastosis viral oncogene homolog (avian)-like 2	2.76868	7.78758E-07	1.86374E-05
TC16001029.hg.1	SEPT1	1731	septin 1	2.758225	9.48508E-06	0.000126165
TC06002091.hg.1	THEMIS	387357	thymocyte selection associated	2.754579	1.69121E-06	3.36941E-05
TC01003833.hg.1	EPRS	2058	glutamyl-prolyl-tRNA synthetase	2.738078	3.25486E-06	5.5154E-05
TC01000846.hg.1	CDC7	8317	cell division cycle 7	2.73479	1.45029E-08	1.09209E-06
TC17000495.hg.1	CDC6	990	cell division cycle 6	2.72862	3.00775E-07	9.40277E-06
TC11002074.hg.1	UCP2	7351	uncoupling protein 2 (mitochondrial, proton carrier)	2.720654	8.00044E-06	0.000111536
TC19000229.hg.1	RNASEH2A	10535	ribonuclease H2, subunit A	2.709206	1.593E-10	7.81319E-08
TC17000834.hg.1	SLC9A3R1	9368	solute carrier family 9, subfamily A (NHE3, cation proton antiporter 3), member 3 regulat	2.702916	6.93794E-10	1.58351E-07
TC06000164.hg.1	HIST1H3C	8352	histone cluster 1, H3c	2.701112	3.86631E-10	1.20001E-07
TC20000040.hg.1	CDC25B	994	cell division cycle 25B	2.696314	6.77511E-07	1.67216E-05
TC01003796.hg.1	SLC30A1	7779	solute carrier family 30 (zinc transporter), member 1	2.69551	0.01333209	0.04199368
TC16001133.hg.1	NUDT21	11051	nudix (nucleoside diphosphate linked moiety X)-type motif 21	2.693061	4.65417E-08	2.49155E-06
TC16000349.hg.1	ITGAL	3683	integrin, alpha L (antigen CD11A (p180), lymphocyte function-associated antigen 1; alpha	2.68525	3.13572E-06	5.34981E-05
TC06001430.hg.1	HIST1H2AM	8336	histone cluster 1, H2am	2.684342	1.90809E-12	9.67172E-09
TC04001436.hg.1	CENPE	1062	centromere protein E, 312kDa	2.682373	2.02924E-06	3.85568E-05
TC11001173.hg.1	DCPS	28960	decapping enzyme, scavenger	2.678642	1.16104E-08	9.61616E-07
TC02001761.hg.1	SOS1	6654	son of sevenless homolog 1 (Drosophila)	2.674987	3.45384E-05	0.000346707
TC03001832.hg.1	COPB2	9276	coatomer protein complex, subunit beta 2 (beta prime)	2.674202	4.94304E-11	4.36035E-08
TC08001584.hg.1	ATAD2	29028	ATPase family, AAA domain containing 2	2.671926	1.6273E-08	1.18936E-06
TC20000821.hg.1	SAMHD1	25939	SAM domain and HD domain 1	2.666456	0.002508489	0.01102781
TC10000664.hg.1	CEP55	55165	centrosomal protein 55kDa	2.665855	1.41023E-08	1.08669E-06
TC17000531.hg.1	TUBG1	7283	tubulin, gamma 1	2.660566	3.57288E-09	4.75086E-07
TC03001257.hg.1	EOMES	8320	eomesodermin	2.652412	9.34943E-06	0.000125224
TC16001234.hg.1	AARS	16	alanyl-tRNA synthetase	2.641395	4.57704E-09	5.35579E-07
TC13000491.hg.1	PARP4	143	poly (ADP-ribose) polymerase family, member 4	2.636636	4.14384E-08	2.30783E-06
TC12000160.hg.1	KLRD1	3824	killer cell lectin-like receptor subfamily D, member 1	2.618959	0.000201777	0.001437122
TC12000496.hg.1	CDK2	1017	cyclin-dependent kinase 2	2.613135	9.65312E-10	2.01189E-07
TC12001489.hg.1	RACGAP1	29127	Rac GTPase activating protein 1	2.60896	4.07966E-11	4.29252E-08
TC16000579.hg.1	SF3B3	23450	splicing factor 3b, subunit 3, 130kDa	2.606592	8.97904E-08	4.00695E-06
TC17000839.hg.1	ICT1	3396	immature colon carcinoma transcript 1	2.603171	5.8398E-07	1.50146E-05
TC15001652.hg.1	COX5A	9377	cytochrome c oxidase subunit Va	2.594281	4.18297E-10	1.25861E-07
TC11000790.hg.1	POLD3	10714	polymerase (DNA-directed), delta 3, accessory subunit	2.593182	8.97002E-10	1.91485E-07
TC0X000609.hg.1	SH2D1A	4068	SH2 domain containing 1A	2.582058	1.43414E-08	1.09056E-06
TC12000818.hg.1	C12orf75	387882	chromosome 12 open reading frame 75	2.580144	7.96695E-07	1.89884E-05
TC11001981.hg.1	PPP1CA	5499	protein phosphatase 1, catalytic subunit, alpha isozyme	2.57601	2.84544E-07	9.03981E-06
TC05000639.hg.1	RAD50	10111	RAD50 homolog (S. cerevisiae)	2.575043	2.67261E-06	4.75584E-05
TC03000715.hg.1	DNAJC13	23317	DnaJ (Hsp40) homolog, subfamily C, member 13	2.575008	9.16483E-06	0.000123487
TC18000180.hg.1	ME2	4200	malic enzyme 2, NAD(+)-dependent, mitochondrial	2.572465	2.91966E-07	9.21204E-06
TC07000235.hg.1	ANLN	54443	anillin, actin binding protein	2.556125	7.97113E-09	7.72316E-07
TC07001499.hg.1	BAZ1B	9031	bromodomain adjacent to zinc finger domain, 1B	2.553162	3.67881E-09	4.86008E-07
TC05001024.hg.1	LOC728554	728554	THO complex 3 pseudogene	2.553144	1.05407E-06	2.33766E-05
TC09001547.hg.1	STOM	2040	stomatin	2.550258	9.43002E-08	4.13476E-06
TC09000358.hg.1	PSAT1	29968	phosphoserine aminotransferase 1	2.543753	2.03676E-10	8.38535E-08
TC20000822.hg.1	RBL1	5933	retinoblastoma-like 1 (p107)	2.542243	4.24751E-08	2.341E-06
TC17000402.hg.1	TAF15	8148	TAF15 RNA polymerase II, TATA box binding protein (TBP)-associated factor, 68kDa	2.533617	2.32261E-08	1.5061E-06
TC06000172.hg.1	HIST1H4E	8367	histone cluster 1, H4e	2.52895	8.52686E-09	7.98755E-07
TC12000925.hg.1	RFC5	5985	replication factor C (activator 1) 5, 36.5kDa	2.524869	2.0471E-08	1.38876E-06
TC03000803.hg.1	HPS3	84343	Hermansky-Pudlak syndrome 3	2.524489	5.60454E-06	8.4759E-05
TC08001248.hg.1	TOX	9760	thymocyte selection-associated high mobility group box	2.522895	0.001875587	0.008773683
TC03000851.hg.1	GMPS	8833	guanine monphosphate synthetase	2.520986	5.87078E-09	6.26963E-07
TC17000613.hg.1	TBX21	30009	T-box 21	2.519353	1.04697E-06	2.32679E-05
TC03000242.hg.1	KIF15	56992	kinesin family member 15	2.507266	2.09226E-09	3.44582E-07
TC06001421.hg.1	HIST1H2BL	8340	histone cluster 1, H2bl	2.499304	1.49171E-10	7.67883E-08

transcript cluster id	Gene Symbol	Entrez Gene ID	Description	FoldChange (AIM/control)	P.Value (AIM/control)	adj.P.Val (AIM/control)
TC01000563.hg.1	KIF2C	11004	kinesin family member 2C	2.488627	5.70812E-10	1.41553E-07
TC16001239.hg.1	COG4	25839	component of oligomeric golgi complex 4	2.488164	7.32471E-10	1.65572E-07
TC15000328.hg.1	TUBGCP4	27229	tubulin, gamma complex associated protein 4	2.488018	5.13518E-08	2.67694E-06
TC03001882.hg.1	HLTF	6596	helicase-like transcription factor	2.486885	2.00309E-09	3.32523E-07
TC12001572.hg.1	GTSF1	121355	gametocyte specific factor 1	2.486633	1.37146E-05	0.000168416
TC12001605.hg.1	TIMELESS	8914	timeless circadian clock	2.484407	9.11423E-09	8.23945E-07
TC12000761.hg.1	TMPO	7112	thymopoietin	2.480874	6.42713E-06	9.47071E-05
TC02001895.hg.1	CCT4	10575	chaperonin containing TCP1, subunit 4 (delta)	2.480693	5.77237E-07	1.49133E-05
TC06001980.hg.1	PREP	5550	prolyl endopeptidase	2.474419	3.24984E-07	9.88115E-06
TC04000331.hg.1	PAICS	10606	phosphoribosylaminoimidazole carboxylase, phosphoribosylaminoimidazole succinocarbo	2.471779	2.6683E-07	8.66066E-06
TC14002306.hg.1	UBR7	55148	ubiquitin protein ligase E3 component n-recognin 7 (putative)	2.466084	3.78315E-08	2.15017E-06
TC09001051.hg.1	STOML2	30968	stomatin (EPB72)-like 2	2.46401	3.69312E-08	2.10955E-06
TC20000384.hg.1	CSE1L	1434	CSE1 chromosome segregation 1-like (yeast)	2.45952	2.40475E-07	8.11281E-06
TC11003488.hg.1	HNRNPUL2	221092	heterogeneous nuclear ribonucleoprotein U-like 2	2.456808	1.15263E-08	9.61616E-07
TC12000974.hg.1	KNTC1	9735	kinetochore associated 1	2.454008	1.35587E-07	5.35669E-06
TC0X001113.hg.1	PDZD11	51248	PDZ domain containing 11	2.448843	9.166E-12	1.78611E-08
TC11002362.hg.1	H2AFX	3014	H2A histone family, member X	2.447841	2.30346E-08	1.50146E-06
TC09000463.hg.1	HIATL1	84641	hippocampus abundant transcript-like 1	2.446546	4.17194E-06	6.71394E-05
TC12000569.hg.1	XPOT	11260	exportin, tRNA	2.44545	2.18441E-06	4.08162E-05
TC03001390.hg.1	SLC25A20	788	solute carrier family 25 (carnitine/acylcarnitine translocase), member 20	2.439158	9.19422E-09	8.26548E-07
TC14000466.hg.1	EIF2B2	8892	eukaryotic translation initiation factor 2B, subunit 2 beta, 39kDa	2.439048	1.75596E-08	1.25372E-06
TC06001675.hg.1	STK38	11329	serine/threonine kinase 38	2.436612	0.000461171	0.002812484
TC01001516.hg.1	DARS2	55157	aspartyl-tRNA synthetase 2, mitochondrial	2.43518	6.843E-10	1.58351E-07
TC03001283.hg.1	TRANK1	9881	tetratricopeptide repeat and ankyrin repeat containing 1	2.433594	4.24292E-05	0.000407814
TC06001426.hg.1	HIST1H1B	3009	histone cluster 1, H1b	2.432832	6.46505E-07	1.62065E-05
TC11003431.hg.1	DPP3	10072	dipeptidyl-peptidase 3	2.431464	2.39507E-09	3.71801E-07
TC10000468.hg.1	SEC24C	9632	SEC24 family, member C (S. cerevisiae)	2.42963	7.22922E-12	1.69617E-08
TC14000916.hg.1	SUPT16H	11198	suppressor of Ty 16 homolog (S. cerevisiae)	2.428362	2.07312E-06	3.92754E-05
TC12003244.hg.1	PA2G4	5036	proliferation-associated 2G4, 38kDa	2.42703	1.24482E-06	2.65637E-05
TC11000585.hg.1	STIP1	10963	stress-induced-phosphoprotein 1	2.426009	6.72009E-08	3.24916E-06
TC16000851.hg.1	CARHSP1	23589	calcium regulated heat stable protein 1, 24kDa	2.424701	6.28936E-11	5.11859E-08
TC11000622.hg.1	POLA2	23649	polymerase (DNA directed), alpha 2, accessory subunit	2.424409	1.53774E-09	2.82152E-07
TC01003876.hg.1	PARP1	142	poly (ADP-ribose) polymerase 1	2.424143	3.18699E-08	1.88734E-06
TC14000491.hg.1	AHSA1	10598	AHA1, activator of heat shock 90kDa protein ATPase homolog 1 (yeast)	2.423161	1.18437E-08	9.84773E-07
TC06001354.hg.1	HIST1H3G	8355	histone cluster 1, H3g	2.422021	2.71044E-08	1.69339E-06
TC14000584.hg.1	IFI27	3429	interferon, alpha-inducible protein 27	2.42045	0.01483453	0.04573167
TC01000433.hg.1	RBBP4	5928	retinoblastoma binding protein 4	2.415302	7.31915E-08	3.41521E-06
TC17000466.hg.1	PSMB3	5691	proteasome (prosome, macropain) subunit, beta type, 3	2.402789	1.40038E-06	2.90451E-05
TC07003353.hg.1	PSMA2	5683	proteasome (prosome, macropain) subunit, alpha type, 2	2.38377	2.55049E-07	8.4371E-06
TC01001174.hg.1	HIST2H2AA4	723790	histone cluster 2, H2aa4	2.383015	5.14779E-09	5.80124E-07
TC03001188.hg.1	NUP210	23225	nucleoporin 210kDa	2.364964	3.18906E-05	0.000326252
TC17001637.hg.1	SKAP1	8631	src kinase associated phosphoprotein 1	2.364008	7.49161E-05	0.000641388
TC11000187.hg.1	CTR9	9646	Ctr9, Paf1/RNA polymerase II complex component, homolog (S. cerevisiae)	2.361994	2.28997E-08	1.50102E-06
TC06000184.hg.1	BTN3A2	11118	butyrophilin, subfamily 3, member A2	2.361879	0.00015915	0.001186482
TC02001271.hg.1	XRCC5	7520	X-ray repair complementing defective repair in Chinese hamster cells 5 (double-strand-br	2.360842	1.62472E-08	1.19371E-06
TC01000972.hg.1	CHI3L2	1117	chitinase 3-like 2	2.36079	2.86097E-10	1.02749E-07
TC12003257.hg.1	COX6A1	1337	cytochrome c oxidase subunit VIa polypeptide 1	2.358291	2.77016E-07	8.9165E-06
TC17000057.hg.1	RNF167	26001	ring finger protein 167	2.357866	2.23238E-07	7.68574E-06
TC06001350.hg.1	HIST1H2BG	8339	histone cluster 1, H2bg	2.353175	9.40538E-07	2.14491E-05
TC11000006.hg.1	PSMD13	5719	proteasome (prosome, macropain) 26S subunit, non-ATPase, 13	2.35274	2.84825E-09	4.10895E-07
TC0X000050.hg.1	PRPS2	5634	phosphoribosyl pyrophosphate synthetase 2	2.348463	2.35234E-07	7.99658E-06
TC11000563.hg.1	POLR2G	5436	polymerase (RNA) II (DNA directed) polypeptide G	2.346272	1.65894E-08	1.21003E-06
TC15000223.hg.1	ARHGAP11A	9824	Rho GTPase activating protein 11A	2.342017	2.12545E-08	1.42932E-06
TC01002658.hg.1	ORC1	4998	origin recognition complex, subunit 1	2.341928	8.78039E-09	8.12997E-07
TC14001154.hg.1	WDHD1	11169	WD repeat and HMG-box DNA binding protein 1	2.3403	5.56586E-09	6.14551E-07
TC21000154.hg.1	CHAF1B	8208	chromatin assembly factor 1, subunit B (p60)	2.3392	3.08196E-08	1.85322E-06
TC20000942.hg.1	NFATC2	4773	nuclear factor of activated T-cells, cytoplasmic, calcineurin-dependent 2	2.337881	1.60283E-05	0.000190366
TC01003405.hg.1	CD84	8832	CD84 molecule	2.331419	3.79334E-08	2.15417E-06
TC05000368.hg.1	IQGAP2	10788	IQ motif containing GTPase activating protein 2	2.325155	0.000336741	0.002172185
TC15000290.hg.1	CASC5	57082	cancer susceptibility candidate 5	2.322073	5.43263E-09	6.0578E-07
TC10001014.hg.1	PRKCQ	5588	protein kinase C, theta	2.321883	9.31987E-06	0.000125224
TC19001555.hg.1	EXOSC5	56915	exosome component 5	2.320429	6.11187E-06	9.07808E-05
TC01000545.hg.1	CDC20	991	cell division cycle 20	2.313761	6.38626E-14	1.14235E-09
TC11001851.hg.1	DDB1	1642	damage-specific DNA binding protein 1, 127kDa	2.306673	1.00353E-07	4.3309E-06
TC20000784.hg.1	PIGU	128869	phosphatidylinositol glycan anchor biosynthesis, class U	2.305477	3.76368E-09	4.94506E-07
TC16000342.hg.1	CORO1A	11151	coronin, actin binding protein, 1A	2.303679	1.1419E-08	9.61616E-07
TC10000802.hg.1	SMC3	9126	structural maintenance of chromosomes 3	2.30311	2.59891E-06	4.66738E-05
TC10001101.hg.1	PIP4K2A	5305	phosphatidylinositol-5-phosphate 4-kinase, type II, alpha	2.296335	1.58882E-05	0.000189294
TC16002104.hg.1	BOLA2	552900	bolA homolog 2 (E. coli)	2.295744	7.21195E-08	3.3976E-06
TC12000886.hg.1	OAS2	4939	2'-5'-oligoadenylate synthetase 2, 69/71kDa	2.294474	0.002426181	0.01075426
TC15001667.hg.1	PTPN9	5780	protein tyrosine phosphatase, non-receptor type 9	2.282616	5.70704E-08	2.88636E-06
TC10000900.hg.1	BUB3	9184	BUB3 mitotic checkpoint protein	2.276283	9.21929E-08	4.09556E-06

transcript cluster id	Gene Symbol	Entrez Gene ID	Description	FoldChange (AIM/control)	P.Value (AIM/control)	adj.P.Val (AIM/control)
TC13000119.hg.1	RFC3	5983	replication factor C (activator 1) 3, 38kDa	2.274716	1.61408E-06	3.25867E-05
TC11000133.hg.1	ILK	3611	integrin-linked kinase	2.274325	5.16488E-09	5.80399E-07
TC0X001180.hg.1	ITM2A	9452	integral membrane protein 2A	2.272311	7.57596E-05	0.000647762
TC01001814.hg.1	IARS2	55699	isoleucyl-tRNA synthetase 2, mitochondrial	2.270665	1.0728E-07	4.54301E-06
TC01000342.hg.1	CD52	1043	CD52 molecule	2.266985	8.80409E-05	0.000728734
TC08001620.hg.1	LINC00861	100130231	long intergenic non-protein coding RNA 861	2.262409	0.000141581	0.001080046
TC09001331.hg.1	IARS	3376	isoleucyl-tRNA synthetase	2.260426	3.58184E-07	1.06586E-05
TC01003887.hg.1	LBR	3930	lamin B receptor	2.25723	1.2814E-08	1.03769E-06
TC02001065.hg.1	AGPS	8540	alkylglycerone phosphate synthase	2.25478	6.87216E-07	1.69761E-05
TC12000343.hg.1	TMEM106C	79022	transmembrane protein 106C	2.250372	4.04323E-09	5.07262E-07
TC03000563.hg.1	CD96	10225	CD96 molecule	2.249686	0.001074663	0.005630493
TC02000245.hg.1	GALM	130589	galactose mutarotase (aldose 1-epimerase)	2.249454	3.6157E-05	0.00035994
TC18000178.hg.1	SKA1	220134	spindle and kinetochore associated complex subunit 1	2.248831	1.68119E-05	0.000197706
TC06000593.hg.1	RRP36	88745	ribosomal RNA processing 36 homolog (S. cerevisiae)	2.246618	5.75465E-09	6.26286E-07
TC01000707.hg.1	USP1	7398	ubiquitin specific peptidase 1	2.244973	1.59259E-08	1.18462E-06
TC22000319.hg.1	GRAP2	9402	GRB2-related adaptor protein 2	2.243755	8.11925E-08	3.71204E-06
TC04000031.hg.1	SCARNA22	677770	small Cajal body-specific RNA 22	2.243568	6.0431E-06	9.00454E-05
TC07002083.hg.1	NCAPG2	54892	non-SMC condensin II complex, subunit G2	2.242208	4.4252E-07	1.25177E-05
TC04000213.hg.1	PGM2	55276	phosphoglucomutase 2	2.240098	1.13053E-07	4.73965E-06
TC06000684.hg.1	BAG2	9532	BCL2-associated athanogene 2	2.239459	8.55842E-09	8.03376E-07
TC01003113.hg.1	CD160	11126	CD160 molecule	2.237299	1.31334E-05	0.000162979
TC0X001522.hg.1	HCFC1	3054	host cell factor C1 (VP16-accessory protein)	2.233237	7.68951E-08	3.55344E-06
TC06000268.hg.1	HIST1H2BN	8341	histone cluster 1, H2bn	2.232814	2.40821E-08	1.56134E-06
TC0X001530.hg.1	FLNA	2316	filamin A, alpha	2.232719	9.93352E-09	8.73695E-07
TC16001318.hg.1	GINS2	51659	GINS complex subunit 2 (Psf2 homolog)	2.230739	9.2749E-13	9.67172E-09
TC12001458.hg.1	DDX23	9416	DEAD (Asp-Glu-Ala-Asp) box polypeptide 23	2.227955	4.64172E-10	1.3648E-07
TC15002760.hg.1	ARHGAP11B	89839	Rho GTPase activating protein 11B	2.226738	1.81009E-08	1.28479E-06
TC16000977.hg.1	NSMCE1	197370	non-SMC element 1 homolog (S. cerevisiae)	2.220039	1.51988E-05	0.000183347
TC02000440.hg.1	CCT7	10574	chaperonin containing TCP1, subunit 7 (eta)	2.219411	6.69486E-08	3.25328E-06
TC07000932.hg.1	CASP2	835	caspase 2, apoptosis-related cysteine peptidase	2.216234	4.75208E-09	5.53337E-07
TC06000358.hg.1	TCF19	6941	transcription factor 19	2.215187	3.03081E-07	9.53021E-06
TC15001685.hg.1	ETFA	2108	electron-transfer-flavoprotein, alpha polypeptide	2.215064	2.82443E-09	4.10895E-07
TC01002340.hg.1	HNRNPR	10236	heterogeneous nuclear ribonucleoprotein R	2.213816	1.25359E-06	2.67428E-05
TC17001098.hg.1	TRAPPC1	58485	trafficking protein particle complex 1	2.213478	1.04766E-06	2.33766E-05
TC05001307.hg.1	OXCT1	5019	3-oxoacid CoA transferase 1	2.207492	9.69348E-07	2.20088E-05
TC08001616.hg.1	KIAA0196	9897	KIAA0196	2.206735	4.3561E-10	1.31302E-07
TC02001083.hg.1	ITGA4	3676	integrin, alpha 4 (antigen CD49D, alpha 4 subunit of VLA-4 receptor)	2.202291	9.62E-05	0.000786174
TC01002611.hg.1	PRDX1	5052	peroxiredoxin 1	2.200109	3.87218E-07	1.12948E-05
TC07000889.hg.1	AGK	55750	acylglycerol kinase	2.199497	4.2082E-08	2.34122E-06
TC14000371.hg.1	PRKCH	5583	protein kinase C, eta	2.197284	1.69322E-07	6.36264E-06
TC22001478.hg.1	SAMM50	25813	sorting and assembly machinery component 50 homolog (S. cerevisiae)	2.193199	6.42901E-09	6.70912E-07
TC04000989.hg.1	LYAR	55646	Ly1 antibody reactive	2.189996	7.22108E-10	1.65572E-07
TC20000816.hg.1	NDRG3	57446	NDRG family member 3	2.188319	1.92208E-05	0.000219258
TC14000931.hg.1	DAD1	1603	defender against cell death 1	2.183229	1.91319E-07	6.93873E-06
TC19000226.hg.1	ASNA1	439	arsA arsenite transporter, ATP-binding, homolog 1 (bacterial)	2.176347	1.03029E-08	8.93452E-07
TC16000492.hg.1	GPR114	221188	G protein-coupled receptor 114	2.176158	1.50123E-07	5.80635E-06
TC06000592.hg.1	KLHDC3	116138	kelch domain containing 3	2.175391	7.81539E-09	7.72316E-07
TC16000050.hg.1	NDUFB10	4716	NADH dehydrogenase (ubiquinone) 1 beta subcomplex, 10, 22kDa	2.174653	7.74E-10	1.74122E-07
TC02000642.hg.1	MRPS9	64965	mitochondrial ribosomal protein S9	2.174542	2.53142E-10	9.53515E-08
TC09000415.hg.1	CKS2	1164	CDC28 protein kinase regulatory subunit 2	2.173276	1.48113E-07	5.76993E-06
TC12001330.hg.1	ASUN	55726	asunder spermatogenesis regulator	2.173153	2.9775E-09	4.24315E-07
TC14000159.hg.1	PSME1	5720	proteasome (prosome, macropain) activator subunit 1 (PA28 alpha)	2.170048	3.18802E-07	9.8224E-06
TC12003287.hg.1	VPS33A	65082	vacuolar protein sorting 33 homolog A (S. cerevisiae)	2.170038	5.23721E-12	1.49591E-08
TC17001869.hg.1	ATP5H	10476	ATP synthase, H+ transporting, mitochondrial Fo complex, subunit d	2.169581	1.86078E-06	3.64563E-05
TC11001746.hg.1	MTCH2	23788	mitochondrial carrier 2	2.169333	2.92975E-07	9.31023E-06
TC06001344.hg.1	HIST1H2BB	3018	histone cluster 1, H2bb	2.169213	1.43876E-07	5.64101E-06
TC02002052.hg.1	IMMT	10989	inner membrane protein, mitochondrial	2.168301	1.03899E-09	2.12817E-07
TC08001110.hg.1	GSR	2936	glutathione reductase	2.16757	8.42341E-09	7.99275E-07
TC02002217.hg.1	CKAP2L	150468	cytoskeleton associated protein 2-like	2.16586	2.2707E-06	4.22564E-05
TC06000188.hg.1	BTN3A3	10384	butyrophilin, subfamily 3, member A3	2.162895	2.60995E-05	0.000278985
TC16001269.hg.1	RFWD3	55159	ring finger and WD repeat domain 3	2.16227	4.24243E-07	1.21238E-05
TC06001970.hg.1	ASCC3	10973	activating signal cointegrator 1 complex subunit 3	2.159339	7.20757E-05	0.00062301
TC20000469.hg.1	ZNF831	128611	zinc finger protein 831	2.15714	6.91136E-06	0.000100053
TC16001267.hg.1	GLG1	2734	golgi glycoprotein 1	2.156719	0.000118247	0.000932658
TC01003414.hg.1	ARHGAP30	257106	Rho GTPase activating protein 30	2.156677	2.26259E-07	7.79297E-06
TC19000097.hg.1	UHRF1	29128	ubiquitin-like with PHD and ring finger domains 1	2.155553	4.88202E-11	4.36035E-08
TC17001036.hg.1	PFN1	5216	profilin 1	2.151642	7.23731E-08	3.41521E-06
TC04001686.hg.1	PPID	5481	peptidylprolyl isomerase D	2.149307	2.14751E-08	1.44553E-06
TC10002952.hg.1	ARHGAP19	84986	Rho GTPase activating protein 19	2.145479	1.09759E-06	2.43137E-05
TC17001372.hg.1	SLFN12L	100506736	schlafen family member 12-like	2.145452	2.06884E-06	3.9381E-05
TC12001403.hg.1	ZCRB1	85437	zinc finger CCHC-type and RNA binding motif 1	2.144392	4.40073E-08	2.41566E-06
TC12000062.hg.1	RAD51AP1	10635	RAD51 associated protein 1	2.138607	7.18736E-05	0.000622022

transcript cluster id	Gene Symbol	Entrez Gene ID	Description	FoldChange (AIM/control)	P.Value (AIM/control)	adj.P.Val (AIM/control)
TC19000567.hg.1	SNRPA	6626	small nuclear ribonucleoprotein polypeptide A	2.134668	3.76748E-07	1.10722E-05
TC19001016.hg.1	ABHD17A	81926	abhydrolase domain containing 17A	2.132102	3.76612E-08	2.15417E-06
TC09001218.hg.1	C9orf41	138199	chromosome 9 open reading frame 41	2.132001	1.69288E-08	1.2369E-06
TC15000731.hg.1	PSMA4	5685	proteasome (prosome, macropain) subunit, alpha type, 4	2.130967	3.48831E-07	1.05728E-05
TC16001083.hg.1	SHCBP1	79801	SHC SH2-domain binding protein 1	2.126865	1.18071E-05	0.000149856
TC05001275.hg.1	NUP155	9631	nucleoporin 155kDa	2.126327	2.13117E-06	4.02154E-05
TC20000779.hg.1	EIF2S2	8894	eukaryotic translation initiation factor 2, subunit 2 beta, 38kDa	2.126199	1.51242E-08	1.1469E-06
TC04000645.hg.1	PLK4	10733	polo-like kinase 4	2.126133	1.08221E-05	0.000140474
TC15001465.hg.1	RAB27A	5873	RAB27A, member RAS oncogene family	2.125718	2.77517E-08	1.72547E-06
TC11003469.hg.1	STT3A	3703	STT3A, subunit of the oligosaccharyltransferase complex (catalytic)	2.125585	1.02796E-05	0.00013475
TC03001751.hg.1	RUVBL1	8607	RuvB-like 1 (E. coli)	2.12448	1.25068E-08	1.02726E-06
TC12002084.hg.1	MPHOSPH9	10198	M-phase phosphoprotein 9	2.124441	1.83652E-07	6.77494E-06
TC12001353.hg.1	IPO8	10526	importin 8	2.121846	3.21087E-06	5.50292E-05
TC12002032.hg.1	CIT	11113	citron (rho-interacting, serine/threonine kinase 21)	2.121197	5.06677E-10	1.41553E-07
TC05001617.hg.1	ERAP1	51752	endoplasmic reticulum aminopeptidase 1	2.120638	0.000208454	0.001477743
TC04000421.hg.1	USO1	8615	USO1 vesicle transport factor	2.117544	2.45037E-05	0.000265847
TC12001492.hg.1	LIMA1	51474	LIM domain and actin binding 1	2.117118	3.60437E-08	2.10299E-06
TC09000035.hg.1	JAK2	3717	Janus kinase 2	2.115795	0.001731022	0.008256072
TC11000324.hg.1	CAPRIN1	4076	cell cycle associated protein 1	2.115324	1.18912E-08	9.92906E-07
TC0X001416.hg.1	ARHGEF6	9459	Rac/Cdc42 guanine nucleotide exchange factor (GEF) 6	2.113335	0.001504394	0.007377903
TC02001736.hg.1	STRN	6801	striatin, calmodulin binding protein	2.111908	7.78162E-08	3.60083E-06
TC11001793.hg.1	SLC43A1	8501	solute carrier family 43, member 1	2.110832	2.64859E-08	1.68246E-06
TC22001467.hg.1	APOBEC3F	200316	apolipoprotein B mRNA editing enzyme, catalytic polypeptide-like 3F	2.108994	1.62443E-08	1.20324E-06
TC16000634.hg.1	CENPN	55839	centromere protein N	2.107418	2.97384E-06	5.18026E-05
TC14000608.hg.1	VRK1	7443	vaccinia related kinase 1	2.10645	3.3424E-09	4.60403E-07
TC17001769.hg.1	BRIP1	83990	BRCA1 interacting protein C-terminal helicase 1	2.106223	7.06888E-11	5.68442E-08
TC09001042.hg.1	SIGMAR1	10280	sigma non-opioid intracellular receptor 1	2.103296	1.40881E-07	5.56378E-06
TC10000673.hg.1	HELLS	3070	helicase, lymphoid-specific	2.10254	2.87348E-06	5.06656E-05
TC19002626.hg.1	BABAM1	29086	BRISC and BRCA1 A complex member 1	2.102088	5.60694E-08	2.88309E-06
TC15001264.hg.1	ZNF106	64397	zinc finger protein 106	2.100952	1.49949E-05	0.000181581
TC02001663.hg.1	ASXL2	55252	additional sex combs like 2 (Drosophila)	2.100695	0.000212125	0.001495557
TC12000612.hg.1	YEATS4	8089	YEATS domain containing 4	2.100234	2.83799E-05	0.000298663
TC19001256.hg.1	RASAL3	64926	RAS protein activator like 3	2.099133	2.12666E-06	4.02154E-05
TC02002478.hg.1	DPP4	1803	dipeptidyl-peptidase 4	2.09605	0.0041299	0.01642864
TC01002289.hg.1	RCC2	55920	regulator of chromosome condensation 2	2.095537	1.93821E-09	3.32126E-07
TC19000527.hg.1	PSMD8	5714	proteasome (prosome, macropain) 26S subunit, non-ATPase, 8	2.09545	2.46114E-07	8.29394E-06
TC17000355.hg.1	ATAD5	79915	ATPase family, AAA domain containing 5	2.094308	1.27757E-07	5.19274E-06
TC18000547.hg.1	LMAN1	3998	lectin, mannose-binding, 1	2.093652	0.000544882	0.003237155
TC12001544.hg.1	ITGB7	3695	integrin, beta 7	2.093033	0.000114116	0.000905648
TC07000843.hg.1	NUP205	23165	nucleoporin 205kDa	2.090474	1.04124E-07	4.47447E-06
TC01003365.hg.1	FCRL3	115352	Fc receptor-like 3	2.090215	0.01039625	0.03438025
TC07000276.hg.1	OGDH	4967	oxoglutarate (alpha-ketoglutarate) dehydrogenase (lipoamide)	2.089841	5.19324E-06	8.0066E-05
TC16000569.hg.1	CYB5B	80777	cytochrome b5 type B (outer mitochondrial membrane)	2.089749	9.4655E-09	8.51318E-07
TC10001423.hg.1	AP3M1	26985	adaptor-related protein complex 3, mu 1 subunit	2.085984	9.29367E-09	8.42208E-07
TC01003085.hg.1	FAM72D	728833	family with sequence similarity 72, member D	2.085912	0.000252005	0.001719113
TC17001586.hg.1	EFTUD2	9343	elongation factor Tu GTP binding domain containing 2	2.085389	4.39699E-06	7.06622E-05
TC06000186.hg.1	BTN3A1	11119	butyrophilin, subfamily 3, member A1	2.085168	3.50265E-05	0.000351486
TC14001085.hg.1	MIS18BP1	55320	MIS18 binding protein 1	2.083865	1.03815E-07	4.47447E-06
TC04002921.hg.1	SDAD1	55153	SDA1 domain containing 1	2.083374	0.000402001	0.002521694
TC01000346.hg.1	HMGN2	3151	high mobility group nucleosomal binding domain 2	2.081521	2.72943E-05	0.000289773
TC05001538.hg.1	DHFR	1719	dihydrofolate reductase	2.079418	4.60787E-09	5.47137E-07
TC17000399.hg.1	AP2B1	163	adaptor-related protein complex 2, beta 1 subunit	2.07589	5.05814E-06	7.81978E-05
TC08000196.hg.1	CDCA2	157313	cell division cycle associated 2	2.075595	3.99802E-07	1.16248E-05
TC0X000783.hg.1	BRCC3	79184	BRCA1/BRCA2-containing complex, subunit 3	2.075554	1.38595E-08	1.08864E-06
TC02001685.hg.1	PPM1G	5496	protein phosphatase, Mg2+/Mn2+ dependent, 1G	2.07293	7.5355E-09	7.60257E-07
TC19000232.hg.1	CALR	811	calreticulin	2.072914	3.30904E-05	0.000337475
TC06004064.hg.1	HIST1H3H	8357	histone cluster 1, H3h	2.072468	8.47329E-05	0.000708738
TC17001056.hg.1	C1QBP	708	complement component 1, q subcomponent binding protein	2.070908	0.000218307	0.001529992
TC21000412.hg.1	GART	2618	phosphoribosylglycinamide formyltransferase, phosphoribosylglycinamide synthetase, ph	2.067838	1.66107E-09	2.97728E-07
TC11001060.hg.1	CD3E	916	CD3e molecule, epsilon (CD3-TCR complex)	2.067203	3.22829E-05	0.000330727
TC03001796.hg.1	TOPBP1	11073	topoisomerase (DNA) II binding protein 1	2.065613	3.12239E-07	9.77467E-06
TC12000095.hg.1	USP5	8078	ubiquitin specific peptidase 5 (isopeptidase T)	2.064511	8.46616E-09	8.03376E-07
TC05001870.hg.1	DIAPH1	1729	diaphanous-related formin 1	2.064244	5.35738E-07	1.42958E-05
TC04001419.hg.1	H2AFZ	3015	H2A histone family, member Z	2.063917	1.96672E-06	3.79859E-05
TC14000316.hg.1	CDKN3	1033	cyclin-dependent kinase inhibitor 3	2.059575	9.77139E-08	4.27818E-06
TC10001042.hg.1	NUDT5	11164	nudix (nucleoside diphosphate linked moiety X)-type motif 5	2.059254	1.21073E-09	2.40406E-07
TC09002909.hg.1	NUP188	23511	nucleoporin 188kDa	2.056867	1.17803E-05	0.000149821
TC06004137.hg.1	ARHGAP18	93663	Rho GTPase activating protein 18	2.056441	2.96353E-08	1.81729E-06
TC21000201.hg.1	UBASH3A	53347	ubiquitin associated and SH3 domain containing A	2.055503	0.000166704	0.001235334
TC19001286.hg.1	LSM4	25804	LSM4 homolog, U6 small nuclear RNA associated (S. cerevisiae)	2.052352	8.30783E-09	7.98755E-07
TC18000480.hg.1	ATP5A1	498	ATP synthase, H+ transporting, mitochondrial F1 complex, alpha subunit 1, cardiac muscle	2.049587	2.19043E-07	7.66471E-06
TC07000246.hg.1	LOC100506776	100506776	uncharacterized LOC100506776	2.049047	7.13242E-06	0.000102816

transcript cluster id	Gene Symbol	Entrez Gene ID	Description	FoldChange (AIM/control)	P.Value (AIM/control)	adj.P.Val (AIM/control)
TC11002390.hg.1	HSPA8	3312	heat shock 70kDa protein 8	2.04798	1.41776E-07	5.60046E-06
TC05000604.hg.1	LMNB1	4001	lamin B1	2.047583	0.00039219	0.002471667
TC05000072.hg.1	CCT5	22948	chaperonin containing TCP1, subunit 5 (epsilon)	2.046327	3.4949E-07	1.05904E-05
TC10001673.hg.1	DCLRE1A	9937	DNA cross-link repair 1A	2.04378	1.78915E-05	0.000208059
TC06002240.hg.1	FBXO5	26271	F-box protein 5	2.039038	3.23227E-06	5.53578E-05
TC02001386.hg.1	PSMD1	5707	proteasome (prosome, macropain) 26S subunit, non-ATPase, 1	2.037333	1.20215E-06	2.61295E-05
TC02000594.hg.1	ZAP70	7535	zeta-chain (TCR) associated protein kinase 70kDa	2.037297	1.06557E-06	2.38127E-05
TC20000868.hg.1	ADA	100	adenosine deaminase	2.036267	1.40727E-06	2.93377E-05
TC14000294.hg.1	TMX1	81542	thioredoxin-related transmembrane protein 1	2.03515	6.80198E-06	9.91443E-05
TC02000047.hg.1	CPSF3	51692	cleavage and polyadenylation specific factor 3, 73kDa	2.034193	6.23907E-10	1.53277E-07
TC08000327.hg.1	VDAC3	7419	voltage-dependent anion channel 3	2.034123	8.81546E-09	8.20169E-07
TC01003337.hg.1	CCT3	7203	chaperonin containing TCP1, subunit 3 (gamma)	2.033754	2.00931E-05	0.000227649
TC11000541.hg.1	FEN1	2237	flap structure-specific endonuclease 1	2.033629	4.53869E-06	7.22687E-05
TC11000303.hg.1	ELP4	26610	elongator acetyltransferase complex subunit 4	2.032622	0.000147831	0.001120061
TC11001873.hg.1	GANAB	23193	glucosidase, alpha; neutral AB	2.031704	3.96755E-06	6.49576E-05
TC06000761.hg.1	TTK	7272	TTK protein kinase	2.031079	1.26202E-06	2.69266E-05
TC17001109.hg.1	AURKB	9212	aurora kinase B	2.030017	5.67553E-09	6.26286E-07
TC11000521.hg.1	TMEM109	79073	transmembrane protein 109	2.028102	5.81497E-07	1.50713E-05
TC02001475.hg.1	PPP1R7	5510	protein phosphatase 1, regulatory subunit 7	2.025725	4.80529E-09	5.62805E-07
TC08001438.hg.1	CCNE2	9134	cyclin E2	2.021797	4.04829E-05	0.000395315
TC03000297.hg.1	APEH	327	acylaminoacyl-peptide hydrolase	2.018793	1.26916E-07	5.18862E-06
TC05000298.hg.1	CCNB1	891	cyclin B1	2.016192	1.7147E-09	3.04836E-07
TC17000577.hg.1	NMT1	4836	N-myristoyltransferase 1	2.014139	2.18879E-09	3.61155E-07
TC03001247.hg.1	TOP2B	7155	topoisomerase (DNA) II beta 180kDa	2.013606	1.03016E-05	0.000135148
TC12001153.hg.1	PHB2	11331	prohibitin 2	2.012764	2.92869E-05	0.000305741
TC06000266.hg.1	HIST1H4J	8363	histone cluster 1, H4j	2.012076	2.82012E-08	1.75768E-06
TC12001938.hg.1	SELPLG	6404	selectin P ligand	2.010208	8.41999E-06	0.000116717
TC01004025.hg.1	FH	2271	fumarate hydratase	2.009779	1.12876E-07	4.76789E-06
TC06002078.hg.1	HDDC2	51020	HD domain containing 2	2.006656	5.65587E-08	2.8955E-06
TC02000599.hg.1	INPP4A	3631	inositol polyphosphate-4-phosphatase, type I, 107kDa	2.005507	8.07896E-06	0.000113214
TC06001302.hg.1	DEK	7913	DEK oncogene	2.005059	8.74678E-07	2.05372E-05
TC06004098.hg.1	PHACTR2	9749	phosphatase and actin regulator 2	2.004992	5.60971E-08	2.88636E-06
TC14001324.hg.1	C14orf1	11161	chromosome 14 open reading frame 1	2.002217	3.82939E-06	6.3364E-05
TC01002414.hg.1	RPA2	6118	replication protein A2, 32kDa	2.000911	8.42642E-08	3.88977E-06
TC17001724.hg.1	SRSF1	6426	serine/arginine-rich splicing factor 1	1.999263	1.93423E-07	7.0159E-06
TC06001345.hg.1	HIST1H1C	3006	histone cluster 1, H1c	1.997924	1.92906E-07	7.00828E-06
TC09000695.hg.1	SPTAN1	6709	spectrin, alpha, non-erythrocytic 1	1.997829	3.38647E-05	0.000343723
TC16000837.hg.1	GLYR1	84656	glyoxylate reductase 1 homolog (Arabidopsis)	1.997802	6.81114E-07	1.69761E-05
TC11001723.hg.1	ARHGAP1	392	Rho GTPase activating protein 1	1.997596	4.70497E-08	2.55838E-06
TC01002955.hg.1	PSMA5	5686	proteasome (prosome, macropain) subunit, alpha type, 5	1.991138	5.51169E-09	6.21268E-07
TC13000426.hg.1	TFDP1	7027	transcription factor Dp-1	1.988711	4.99766E-11	4.45245E-08
TC09001048.hg.1	VCP	7415	valosin containing protein	1.988701	3.3552E-10	1.14126E-07
TC05001463.hg.1	MRPS27	23107	mitochondrial ribosomal protein S27	1.988667	1.07756E-07	4.60845E-06
TC18000191.hg.1	WDR7	23335	WD repeat domain 7	1.987971	2.72244E-05	0.000289717
TC17000640.hg.1	ABI3	51225	ABI family, member 3	1.985334	1.75692E-06	3.50633E-05
TC17000617.hg.1	PNPO	55163	pyridoxamine 5'-phosphate oxidase	1.984577	3.10597E-09	4.42055E-07
TC01003679.hg.1	KIF14	9928	kinesin family member 14	1.983805	6.4686E-08	3.19848E-06
TC06002142.hg.1	MTFR2	113115	mitochondrial fission regulator 2	1.982586	3.08243E-08	1.88114E-06
TC19000112.hg.1	CLPP	8192	ClpP caseinolytic peptidase, ATP-dependent, proteolytic subunit homolog (E. coli)	1.981139	3.91823E-07	1.14892E-05
TC17001408.hg.1	SYNRG	11276	synergin, gamma	1.980272	2.34082E-05	0.000257033
TC11000565.hg.1	TMEM179B	374395	transmembrane protein 179B	1.980192	1.16348E-05	0.000148694
TC12002047.hg.1	COQ5	84274	coenzyme Q5 homolog, methyltransferase (S. cerevisiae)	1.980123	8.52759E-09	8.12342E-07
TC02002886.hg.1	HJURP	55355	Holliday junction recognition protein	1.979041	7.0102E-09	7.25633E-07
TC03001632.hg.1	KIAA1524	57650	KIAA1524	1.977901	1.85379E-06	3.64927E-05
TC01001430.hg.1	NUF2	83540	NUF2, NDC80 kinetochore complex component, homolog (S. cerevisiae)	1.976439	5.98574E-08	3.03092E-06
TC01003753.hg.1	RAB7L1	8934	RAB7, member RAS oncogene family-like 1	1.974609	2.84436E-06	5.0502E-05
TC17000918.hg.1	RNF213	57674	ring finger protein 213	1.971798	0.000592343	0.003469898
TC01003200.hg.1	ANP32E	81611	acidic (leucine-rich) nuclear phosphoprotein 32 family, member E	1.970696	2.52515E-05	0.000272697
TC01002246.hg.1	AGMAT	79814	agmatine ureohydrolase (agmatinase)	1.966184	4.46537E-07	1.26252E-05
TC01001976.hg.1	EXO1	9156	exonuclease 1	1.966093	2.06562E-06	3.94917E-05
TC06000169.hg.1	HIST1H2BD	3017	histone cluster 1, H2bd	1.964481	1.69039E-07	6.38425E-06
TC02001605.hg.1	SMC6	79677	structural maintenance of chromosomes 6	1.962372	3.2686E-06	5.59136E-05
TC14000346.hg.1	PSMA3	5684	proteasome (prosome, macropain) subunit, alpha type, 3	1.961812	4.79262E-06	7.55618E-05
TC06001349.hg.1	HIST1H3D	8351	histone cluster 1, H3d	1.96019	9.18588E-09	8.42208E-07
TC03000664.hg.1	MCM2	4171	minichromosome maintenance complex component 2	1.959402	1.50889E-09	2.85891E-07
TC17000548.hg.1	NBR1	4077	neighbor of BRCA1 gene 1	1.958924	4.37251E-09	5.35579E-07
TC15001650.hg.1	SCAMP2	10066	secretory carrier membrane protein 2	1.958723	7.11014E-07	1.7597E-05
TC12001827.hg.1	NDUFA12	55967	NADH dehydrogenase (ubiquinone) 1 alpha subcomplex, 12	1.958153	2.31268E-06	4.30393E-05
TC02000146.hg.1	RAB10	10890	RAB10, member RAS oncogene family	1.957434	8.53117E-07	2.01943E-05
TC02000203.hg.1	LCLAT1	253558	lysocardiolipin acyltransferase 1	1.956952	1.65799E-07	6.32717E-06
TC0X000574.hg.1	DOCK11	139818	dedicator of cytokinesis 11	1.956428	0.0014671	0.007247455
TC11001423.hg.1	CSNK2A1P	283106	casein kinase 2, alpha 1 polypeptide pseudogene	1.955483	7.97931E-05	0.000677094

transcript cluster id	Gene Symbol	Entrez Gene ID	Description	FoldChange (AIM/control)	P.Value (AIM/control)	adj.P.Val (AIM/control)
TC12001298.hg.1	LDHB	3945	lactate dehydrogenase B	1.955171	0.00015208	0.001146839
TC04001134.hg.1	PDS5A	23244	PDS5, regulator of cohesion maintenance, homolog A (S. cerevisiae)	1.953252	0.000237658	0.001640839
TC17000052.hg.1	PSMB6	5694	proteasome (prosome, macropain) subunit, beta type, 6	1.952747	7.6631E-07	1.86792E-05
TC03002088.hg.1	RFC4	5984	replication factor C (activator 1) 4, 37kDa	1.952005	5.53646E-09	6.23482E-07
TC06002238.hg.1	SYNE1	23345	spectrin repeat containing, nuclear envelope 1	1.951705	0.002308589	0.01034074
TC01001385.hg.1	SDHC	6391	succinate dehydrogenase complex, subunit C, integral membrane protein, 15kDa	1.950761	1.27111E-07	5.19386E-06
TC19000489.hg.1	HCST	10870	hematopoietic cell signal transducer	1.946582	1.37822E-05	0.000170014
TC09000925.hg.1	PSIP1	11168	PC4 and SFRS1 interacting protein 1	1.946416	3.00834E-05	0.000312538
TC11000345.hg.1	PRR5L	79899	proline rich 5 like	1.94451	3.66039E-06	6.12552E-05
TC16001104.hg.1	BRD7	29117	bromodomain containing 7	1.943174	0.000651374	0.003746593
TC05000844.hg.1	G3BP1	10146	GTPase activating protein (SH3 domain) binding protein 1	1.942783	1.50131E-06	3.10598E-05
TC14001346.hg.1	VIPAS39	63894	VPS33B interacting protein, apical-basolateral polarity regulator, spe-39 homolog	1.941866	1.26244E-06	2.70145E-05
TC04001718.hg.1	DDX60	55601	DEAD (Asp-Glu-Ala-Asp) box polypeptide 60	1.940437	7.18456E-05	0.00062301
TC03000689.hg.1	COPG1	22820	coatomer protein complex, subunit gamma 1	1.939765	1.79169E-10	8.38535E-08
TC14000386.hg.1	SYNE2	23224	spectrin repeat containing, nuclear envelope 2	1.939119	0.002471839	0.01092396
TC16000194.hg.1	NOMO3	408050	NODAL modulator 3	1.938071	9.51238E-07	2.18415E-05
TC16000912.hg.1	NOMO2	283820	NODAL modulator 2	1.935883	7.95276E-07	1.92149E-05
TC19000532.hg.1	ACTN4	81	actinin, alpha 4	1.934617	7.28893E-06	0.000104813
TC12001609.hg.1	ATP5B	506	ATP synthase, H+ transporting, mitochondrial F1 complex, beta polypeptide	1.934537	1.34933E-06	2.84952E-05
TC0X000636.hg.1	HPRT1	3251	hypoxanthine phosphoribosyltransferase 1	1.933652	2.52637E-06	4.60213E-05
TC15000802.hg.1	EFTUD1P1	648809	elongation factor Tu GTP binding domain containing 1 pseudogene 1	1.933456	2.15453E-07	7.63043E-06
TC17001914.hg.1	TK1	7083	thymidine kinase 1, soluble	1.93275	4.20032E-09	5.26348E-07
TC09000207.hg.1	MELK	9833	maternal embryonic leucine zipper kinase	1.931821	2.09426E-07	7.47511E-06
TC03001535.hg.1	EOGT	285203	EGF domain-specific O-linked N-acetylglucosamine (GlcNAc) transferase	1.931346	4.64938E-06	7.3919E-05
TC01002345.hg.1	E2F2	1870	E2F transcription factor 2	1.931217	5.52744E-08	2.88251E-06
TC03000609.hg.1	TIMMDC1	51300	translocase of inner mitochondrial membrane domain containing 1	1.930982	1.85578E-07	6.88688E-06
TC11001517.hg.1	KIF18A	81930	kinesin family member 18A	1.926869	1.05964E-07	4.5612E-06
TC01002598.hg.1	RNU5D-1	26830	RNA, U5D small nuclear 1	1.925669	0.003931224	0.01579644
TC13000190.hg.1	RB1	5925	retinoblastoma 1	1.925391	0.000158164	0.0011854
TC01000956.hg.1	AHCYL1	10768	adenosylhomocysteinase-like 1	1.92472	4.08739E-08	2.33194E-06
TC06001341.hg.1	HIST1H4B	8366	histone cluster 1, H4b	1.924606	1.57271E-05	0.000189014
TC02001560.hg.1	YWHAQ	10971	tyrosine 3-monooxygenase/tryptophan 5-monooxygenase activation protein, theta polyp	1.924357	1.10074E-08	9.59059E-07
TC10000067.hg.1	ATP5C1	509	ATP synthase, H+ transporting, mitochondrial F1 complex, gamma polypeptide 1	1.922075	9.33077E-08	4.17801E-06
TC01000493.hg.1	MACF1	23499	microtubule-actin crosslinking factor 1	1.922027	0.000197203	0.001419392
TC04000030.hg.1	WHSC1	7468	Wolf-Hirschhorn syndrome candidate 1	1.921072	2.05291E-10	8.65508E-08
TC19001589.hg.1	XRCC1	7515	X-ray repair complementing defective repair in Chinese hamster cells 1	1.920264	6.71265E-08	3.31088E-06
TC01000504.hg.1	CAP1	10487	CAP, adenylate cyclase-associated protein 1 (yeast)	1.92016	3.56695E-05	0.000357478
TC02002813.hg.1	FARSB	10056	phenylalanyl-tRNA synthetase, beta subunit	1.919198	5.97115E-05	0.000540479
TC17002860.hg.1	KPNA2	3838	karyopherin alpha 2 (RAG cohort 1, importin alpha 1)	1.918996	0.003561715	0.01463079
TC05001827.hg.1	CDC23	8697	cell division cycle 23	1.915275	9.76199E-08	4.3085E-06
TC01003006.hg.1	PTPN22	26191	protein tyrosine phosphatase, non-receptor type 22 (lymphoid)	1.914976	3.26402E-06	5.59136E-05
TC08000216.hg.1	ELP3	55140	elongator acetyltransferase complex subunit 3	1.914965	1.78535E-07	6.69802E-06
TC16000899.hg.1	FOPNL	123811	FGFR1OP N-terminal like	1.913453	1.00352E-05	0.000133009
TC17000535.hg.1	VPS25	84313	vacuolar protein sorting 25 homolog (S. cerevisiae)	1.908794	2.71933E-07	8.9165E-06
TC01002694.hg.1	DHCR24	1718	24-dehydrocholesterol reductase	1.908649	1.6169E-09	2.97728E-07
TC10001551.hg.1	ALDH18A1	5832	aldehyde dehydrogenase 18 family, member A1	1.908162	1.2303E-08	1.02726E-06
TC04001225.hg.1	NOA1	84273	nitric oxide associated 1	1.907714	5.08032E-06	7.89129E-05
TC16000759.hg.1	MRPS34	65993	mitochondrial ribosomal protein S34	1.90752	1.18226E-07	4.95868E-06
TC09000519.hg.1	RNF20	56254	ring finger protein 20, E3 ubiquitin protein ligase	1.907005	7.54437E-09	7.68128E-07
TC16000180.hg.1	NOMO1	23420	NODAL modulator 1	1.905067	1.30307E-06	2.76986E-05
TC03001862.hg.1	SLC9A9	285195	solute carrier family 9, subfamily A (NHE9, cation proton antiporter 9), member 9	1.903782	0.000130101	0.001013382
TC10001309.hg.1	ZWINT	11130	ZW10 interacting kinetochore protein	1.9036	7.10671E-09	7.3611E-07
TC17001627.hg.1	MRPL10	124995	mitochondrial ribosomal protein L10	1.902531	1.74516E-06	3.4993E-05
TC01000476.hg.1	CDCA8	55143	cell division cycle associated 8	1.899586	1.18784E-09	2.40406E-07
TC15000850.hg.1	TICRR	90381	TOPBP1-interacting checkpoint and replication regulator	1.898147	3.13634E-06	5.42263E-05
TC01001915.hg.1	GNPAT	8443	glyceronephosphate O-acyltransferase	1.897741	2.30032E-06	0.000042963
TC01003751.hg.1	NUCKS1	64710	nuclear casein kinase and cyclin-dependent kinase substrate 1	1.894397	6.76487E-06	9.91304E-05
TC0X000376.hg.1	KIF4A	24137	kinesin family member 4A	1.894136	4.31548E-08	2.41566E-06
TC0X001121.hg.1	IL2RG	3561	interleukin 2 receptor, gamma	1.89233	0.000210598	0.001491834
TC05001900.hg.1	LARS	51520	leucyl-tRNA synthetase	1.891337	0.002349551	0.01050013
TC01002796.hg.1	PIGK	10026	phosphatidylinositol glycan anchor biosynthesis, class K	1.891252	0.000962764	0.00515527
TC0X000428.hg.1	GPR174	84636	G protein-coupled receptor 174	1.890182	4.70528E-06	7.4717E-05
TC16000966.hg.1	NDUFAB1	4706	NADH dehydrogenase (ubiquinone) 1, alpha/beta subcomplex, 1, 8kDa	1.889749	2.00545E-05	0.000227737
TC12000018.hg.1	ADIPOR2	79602	adiponectin receptor 2	1.888678	1.8619E-09	3.29773E-07
TC0X000635.hg.1	PHF6	84295	PHD finger protein 6	1.883718	0.000170354	0.001260229
TC01000549.hg.1	KDM4A	9682	lysine (K)-specific demethylase 4A	1.880805	1.45491E-11	2.36556E-08
TC14002328.hg.1	RDH11	51109	retinol dehydrogenase 11 (all-trans/9-cis/11-cis)	1.880366	4.13221E-07	1.20219E-05
TC12001547.hg.1	AAAS	8086	achalasia, adrenocortical insufficiency, alacrimia	1.878861	1.16077E-06	2.55749E-05
TC01002916.hg.1	SASS6	163786	spindle assembly 6 homolog (C. elegans)	1.878635	3.51119E-07	1.06512E-05
TC10001707.hg.1	MCMBP	79892	minichromosome maintenance complex binding protein	1.877783	8.01395E-07	1.93494E-05
TC09001028.hg.1	DCAF12	25853	DDB1 and CUL4 associated factor 12	1.877655	0.003793948	0.01537789
TC16000327.hg.1	KIF22	3835	kinesin family member 22	1.876666	8.00621E-07	1.93494E-05

transcript cluster id	Gene Symbol	Entrez Gene ID	Description	FoldChange (AIM/control)	P.Value (AIM/control)	adj.P.Val (AIM/control)
TC01000347.hg.1	RPS6KA1	6195	ribosomal protein S6 kinase, 90kDa, polypeptide 1	1.875032	4.56555E-07	1.28084E-05
TC15001858.hg.1	PRC1	9055	protein regulator of cytokinesis 1	1.873745	0.000149391	0.001131085
TC11001844.hg.1	PRPF19	27339	pre-mRNA processing factor 19	1.872713	1.7398E-05	0.000204406
TC01001664.hg.1	IPO9	55705	importin 9	1.872648	5.24799E-08	2.77764E-06
TC12001756.hg.1	E2F7	144455	E2F transcription factor 7	1.872609	3.36877E-05	0.000343614
TC22000046.hg.1	CDC45	8318	cell division cycle 45	1.87209	1.40584E-08	1.10304E-06
TC05003400.hg.1	DDX46	9879	DEAD (Asp-Glu-Ala-Asp) box polypeptide 46	1.870977	8.0958E-06	0.000113761
TC01002676.hg.1	NDC1	55706	NDC1 transmembrane nucleoporin	1.870666	7.72198E-09	7.7686E-07
TC11001214.hg.1	VPS26B	112936	vacuolar protein sorting 26 homolog B (S. pombe)	1.870568	1.36649E-08	1.08864E-06
TC11002439.hg.1	ETS1	2113	v-ets erythroblastosis virus E26 oncogene homolog 1 (avian)	1.866963	2.56893E-05	0.000277106
TC07000969.hg.1	CUL1	8454	cullin 1	1.86623	1.04895E-05	0.000137758
TC17001302.hg.1	KIAA0100	9703	KIAA0100	1.865572	3.12454E-09	4.48101E-07
TC01002186.hg.1	CLSTN1	22883	calsyntenin 1	1.865557	4.69012E-05	0.000444483
TC0X000886.hg.1	RBBP7	5931	retinoblastoma binding protein 7	1.864004	7.76533E-06	0.000110383
TC01001751.hg.1	TRAF3IP3	80342	TRAF3 interacting protein 3	1.862058	3.53875E-05	0.000356369
TC11001478.hg.1	E2F8	79733	E2F transcription factor 8	1.861637	1.89272E-07	6.96592E-06
TC08000160.hg.1	XPO7	23039	exportin 7	1.861553	2.09777E-09	3.56194E-07
TC20000829.hg.1	TTI1	9675	TELO2 interacting protein 1	1.861441	5.6487E-07	1.49133E-05
TC11002361.hg.1	HYOU1	10525	hypoxia up-regulated 1	1.860545	0.000223939	0.001563462
TC02000791.hg.1	UGGT1	56886	UDP-glucose glycoprotein glucosyltransferase 1	1.857639	3.44881E-05	0.000348563
TC19001280.hg.1	JAK3	3718	Janus kinase 3	1.857152	4.20406E-06	6.84666E-05
TC14000922.hg.1	METTL3	56339	methyltransferase like 3	1.857081	1.09192E-05	0.000142075
TC01003190.hg.1	HIST2H2AA4	723790	histone cluster 2, H2aa4	1.857038	1.96499E-07	7.16611E-06
TC15001644.hg.1	UBL7	84993	ubiquitin-like 7 (bone marrow stromal cell-derived)	1.854879	1.53657E-06	3.17603E-05
TC01001639.hg.1	NEK7	140609	NIMA-related kinase 7	1.854742	8.37162E-07	2.00086E-05
TC17001094.hg.1	TP53	7157	tumor protein p53	1.854714	1.69878E-07	6.45957E-06
TC17000444.hg.1	AATF	26574	apoptosis antagonizing transcription factor	1.854594	4.86562E-08	2.64994E-06
TC17001171.hg.1	NCOR1	9611	nuclear receptor corepressor 1	1.853609	5.0708E-07	1.38711E-05
TC22000393.hg.1	TTC38	55020	tetratricopeptide repeat domain 38	1.85336	2.11943E-08	1.45601E-06
TC04000559.hg.1	OSTC	58505	oligosaccharyltransferase complex subunit (non-catalytic)	1.853071	6.05322E-05	0.000545855
TC05001678.hg.1	REEP5	7905	receptor accessory protein 5	1.85244	8.93041E-08	4.07819E-06
TC14001441.hg.1	TC2N	123036	tandem C2 domains, nuclear	1.849352	0.002261806	0.01018886
TC06001133.hg.1	TMEM181	57583	transmembrane protein 181	1.848192	6.15394E-07	1.58603E-05
TC0X000388.hg.1	OGT	8473	O-linked N-acetylglucosamine (GlcNAc) transferase	1.847776	0.006616979	0.02410426
TC19000669.hg.1	ARHGAP35	2909	Rho GTPase activating protein 35	1.846664	1.68647E-06	3.42289E-05
TC11000979.hg.1	ACAT1	38	acetyl-CoA acetyltransferase 1	1.846344	2.02879E-07	7.35809E-06
TC19001864.hg.1	PPP6R1	22870	protein phosphatase 6, regulatory subunit 1	1.845673	1.19514E-08	1.01627E-06
TC14001348.hg.1	SPTLC2	9517	serine palmitoyltransferase, long chain base subunit 2	1.845621	8.54957E-07	2.03189E-05
TC10001002.hg.1	GDI2	2665	GDP dissociation inhibitor 2	1.845464	5.29689E-05	0.000492944
TC04000556.hg.1	HADH	3033	hydroxyacyl-CoA dehydrogenase	1.843574	4.91762E-08	2.66968E-06
TC05000161.hg.1	SKP2	6502	S-phase kinase-associated protein 2, E3 ubiquitin protein ligase	1.843488	4.26333E-09	5.3365E-07
TC06002215.hg.1	NUP43	348995	nucleoporin 43kDa	1.840675	1.44602E-05	0.000177372
TC02000603.hg.1	EIF5B	9669	eukaryotic translation initiation factor 5B	1.839393	0.000347736	0.002241749
TC11002199.hg.1	MRE11A	4361	MRE11 meiotic recombination 11 homolog A (S. cerevisiae)	1.838881	9.47015E-08	4.2416E-06
TC19000442.hg.1	GPI	2821	glucose-6-phosphate isomerase	1.838877	1.62268E-06	3.31695E-05
TC18000248.hg.1	CNDP2	55748	CNDP dipeptidase 2 (metallopeptidase M20 family)	1.838115	2.84131E-08	1.78833E-06
TC09001403.hg.1	TRIM14	9830	tripartite motif containing 14	1.836754	7.67468E-07	1.87855E-05
TC02000295.hg.1	MSH2	4436	mutS homolog 2, colon cancer, nonpolyposis type 1 (E. coli)	1.835341	1.42775E-06	2.99735E-05
TC14000940.hg.1	PRMT5	10419	protein arginine methyltransferase 5	1.835244	2.14928E-07	7.66062E-06
TC12002062.hg.1	ANAPC5	51433	anaphase promoting complex subunit 5	1.83497	0.000199516	0.001434325
TC17000396.hg.1	SLFN5	162394	schlafen family member 5	1.834822	0.005897011	0.02200056
TC19002630.hg.1	PSENEN	55851	presenilin enhancer 2 homolog (C. elegans)	1.834432	0.003038358	0.01286624
TC16000884.hg.1	PARN	5073	poly(A)-specific ribonuclease	1.834142	2.29427E-07	7.99658E-06
TC10000373.hg.1	CDK1	983	cyclin-dependent kinase 1	1.832809	6.11104E-06	0.000091847
TC02001627.hg.1	C2orf43	60526	chromosome 2 open reading frame 43	1.831651	3.12572E-08	1.91609E-06
TC03000417.hg.1	ARL6IP5	10550	ADP-ribosylation-like factor 6 interacting protein 5	1.830609	1.12292E-06	2.50152E-05
TC21000353.hg.1	CCT8	10694	chaperonin containing TCP1, subunit 8 (theta)	1.82986	1.23717E-06	2.67736E-05
TC15001736.hg.1	EFTUD1	79631	elongation factor Tu GTP binding domain containing 1	1.828516	4.19508E-08	2.37451E-06
TC12000237.hg.1	CMAS	55907	cytidine monophosphate N-acetylneuraminic acid synthetase	1.828402	4.72659E-09	5.65721E-07
TC20001761.hg.1	CPNE1	8904	copine I	1.828387	3.87806E-06	6.41818E-05
TC0X000586.hg.1	SLC25A5	292	solute carrier family 25 (mitochondrial carrier; adenine nucleotide translocator), member	1.82837	2.07527E-06	3.98863E-05
TC12000949.hg.1	MLEC	9761	malectin	1.826553	2.36761E-07	8.17777E-06
TC19001210.hg.1	PRDX2	7001	peroxiredoxin 2	1.826441	4.70687E-05	0.000446197
TC01003692.hg.1	CSRP1	1465	cysteine and glycine-rich protein 1	1.826413	3.03001E-06	5.28071E-05
TC01000808.hg.1	PRKACB	5567	protein kinase, cAMP-dependent, catalytic, beta	1.826295	2.80506E-05	0.000297667
TC01003868.hg.1	AIDA	64853	axin interactor, dorsalization associated	1.826095	1.08617E-05	0.000141763
TC15000364.hg.1	SQRDL	58472	sulfide quinone reductase-like (yeast)	1.824743	0.000689833	0.003939487
TC07002034.hg.1	ABCF2	10061	ATP-binding cassette, sub-family F (GCN20), member 2	1.824149	6.94022E-06	0.000101275
TC02002211.hg.1	ANAPC1	64682	anaphase promoting complex subunit 1	1.823449	0.000024908	0.000270621
TC06002022.hg.1	FYN	2534	FYN oncogene related to SRC, FGR, YES	1.822748	6.23032E-05	0.00055828
TC17001455.hg.1	IKZF3	22806	IKAROS family zinc finger 3 (Aiolos)	1.821455	0.000371704	0.002367594
TC06001679.hg.1	PPIL1	51645	peptidylprolyl isomerase (cyclophilin)-like 1	1.817382	1.55062E-06	3.20592E-05

transcript cluster id	Gene Symbol	Entrez Gene ID	Description	FoldChange (AIM/control)	P.Value (AIM/control)	adj.P.Val (AIM/control)
TC19000320.hg.1	AP1M1	8907	adaptor-related protein complex 1, mu 1 subunit	1.81684	2.48617E-07	8.44161E-06
TC10000752.hg.1	GBF1	8729	golgi brefeldin A resistant guanine nucleotide exchange factor 1	1.816603	3.46837E-08	2.08377E-06
TC10001584.hg.1	COX15	1355	cytochrome c oxidase assembly homolog 15 (yeast)	1.816106	1.77152E-08	1.29613E-06
TC17000048.hg.1	MED11	400569	mediator complex subunit 11	1.814479	8.50033E-05	0.000713295
TC17000756.hg.1	METTL2A	339175	methyltransferase like 2A	1.814409	1.19388E-06	2.61629E-05
TC11001947.hg.1	FIBP	9158	fibroblast growth factor (acidic) intracellular binding protein	1.8138	5.29624E-07	1.43221E-05
TC20000840.hg.1	SNORA71C	677839	small nucleolar RNA, H/ACA box 71C	1.8132	6.39086E-06	9.54022E-05
TC06001732.hg.1	TRERF1	55809	transcriptional regulating factor 1	1.813043	2.7691E-06	4.98361E-05
TC12000091.hg.1	LAG3	3902	lymphocyte-activation gene 3	1.812314	1.36925E-08	1.09209E-06
TC02000970.hg.1	PSMD14	10213	proteasome (prosome, macropain) 26S subunit, non-ATPase, 14	1.81186	1.2722E-06	2.74025E-05
TC12001507.hg.1	BIN2	51411	bridging integrator 2	1.810053	0.000674482	0.003866656
TC16001031.hg.1	DCTPP1	79077	dCTP pyrophosphatase 1	1.809607	0.00023273	0.001615948
TC14000572.hg.1	GOLGA5	9950	golgin A5	1.808573	1.13547E-06	2.52366E-05
TC12000096.hg.1	TPI1	7167	triosephosphate isomerase 1	1.808518	1.18991E-05	2.61596E-05
TC0X001543.hg.1	GAB3	139716	GRB2-associated binding protein 3	1.808462	2.49112E-05	0.000270683
TC06001908.hg.1	IBTK	25998	inhibitor of Bruton agammaglobulinemia tyrosine kinase	1.807996	4.9926E-05	0.000469211
TC17001895.hg.1	RNF157	114804	ring finger protein 157	1.807432	6.58286E-05	0.000582946
TC12002153.hg.1	POLE	5426	polymerase (DNA directed), epsilon, catalytic subunit	1.806829	4.80787E-09	5.74898E-07
TC16000463.hg.1	OGFOD1	55239	2-oxoglutarate and iron-dependent oxygenase domain containing 1	1.805565	3.05663E-07	9.74992E-06
TC18000537.hg.1	NARS	4677	asparaginyl-tRNA synthetase	1.805552	6.53865E-05	0.000580034
TC18000492.hg.1	HDHD2	84064	haloacid dehalogenase-like hydrolase domain containing 2	1.805316	5.53932E-05	0.000509662
TC11002474.hg.1	THYN1	29087	thymocyte nuclear protein 1	1.803424	7.7141E-09	7.79345E-07
TC12001887.hg.1	NUP37	79023	nucleoporin 37kDa	1.803199	7.58854E-10	1.7906E-07
TC15001337.hg.1	CEP152	22995	centrosomal protein 152kDa	1.802341	4.68365E-06	7.4717E-05
TC10002964.hg.1	MRPS16	51021	mitochondrial ribosomal protein S16	1.802268	5.29971E-07	1.43221E-05
TC17001952.hg.1	ACTG1	71	actin, gamma 1	1.801989	1.24452E-05	0.000157811
TC17001875.hg.1	MIF4GD	57409	MIF4G domain containing	1.80116	2.08885E-07	7.51044E-06
TC15000837.hg.1	MRPS11	64963	mitochondrial ribosomal protein S11	1.800798	2.98228E-12	1.19302E-08
TC19001281.hg.1	IL12RB1	3594	interleukin 12 receptor, beta 1	1.800773	3.24428E-08	1.97457E-06
TC09000700.hg.1	LOC100506100	100506100	uncharacterized LOC100506100	1.800289	1.588E-09	2.97728E-07
TC05000891.hg.1	TTC1	7265	tetratricopeptide repeat domain 1	1.799964	1.29813E-07	5.33043E-06
TC10001201.hg.1	HNRNPF	3185	heterogeneous nuclear ribonucleoprotein F	1.799307	4.04685E-07	1.19265E-05
TC16000528.hg.1	CBFB	865	core-binding factor, beta subunit	1.797332	4.63483E-05	0.00044127
TC03001150.hg.1	TADA3	10474	transcriptional adaptor 3	1.796983	7.57662E-09	7.75332E-07
TC05003396.hg.1	KIF2A	3796	kinesin heavy chain member 2A	1.796055	0.000104326	0.000844801
TC05000148.hg.1	TARS	6897	threonyl-tRNA synthetase	1.793192	1.03527E-06	2.35302E-05
TC04001507.hg.1	MAD2L1	4085	MAD2 mitotic arrest deficient-like 1 (yeast)	1.793161	1.25837E-10	7.47619E-08
TC12000602.hg.1	NUP107	57122	nucleoporin 107kDa	1.793115	4.45378E-07	1.26711E-05
TC06004082.hg.1	PSMB9	5698	proteasome (prosome, macropain) subunit, beta type, 9	1.792639	9.89942E-07	2.26901E-05
TC10000696.hg.1	ZDHHC16	84287	zinc finger, DHHC-type containing 16	1.792521	6.28719E-10	1.58351E-07
TC11000693.hg.1	AIP	9049	aryl hydrocarbon receptor interacting protein	1.79226	1.20642E-06	2.64284E-05
TC02000199.hg.1	LBH	81606	limb bud and heart development	1.788981	3.44186E-05	0.000348563
TC01001378.hg.1	UFC1	51506	ubiquitin-fold modifier conjugating enzyme 1	1.787151	2.96174E-06	5.21465E-05
TC16001027.hg.1	CD2BP2	10421	CD2 (cytoplasmic tail) binding protein 2	1.786792	1.34947E-08	1.08864E-06
TC03000999.hg.1	PSMD2	5708	proteasome (prosome, macropain) 26S subunit, non-ATPase, 2	1.786435	1.08541E-07	4.702E-06
TC01006326.hg.1	HIST2H2AC	8338	histone cluster 2, H2ac	1.785909	5.4141E-09	6.23482E-07
TC16000239.hg.1	UQCRC2	7385	ubiquinol-cytochrome c reductase core protein II	1.784974	1.13058E-06	2.52221E-05
TC03000930.hg.1	ECT2	1894	epithelial cell transforming sequence 2 oncogene	1.784908	2.48259E-06	4.57492E-05
TC12003205.hg.1	NDUFA9	4704	NADH dehydrogenase (ubiquinone) 1 alpha subcomplex, 9, 39kDa	1.784863	8.7732E-05	0.000731038
TC02000378.hg.1	ACTR2	10097	ARP2 actin-related protein 2 homolog (yeast)	1.784164	5.48491E-05	0.00050592
TC12001766.hg.1	PPP1R12A	4659	protein phosphatase 1, regulatory subunit 12A	1.784071	2.29284E-06	4.30462E-05
TC06000591.hg.1	PPP2R5D	5528	protein phosphatase 2, regulatory subunit B', delta	1.780892	1.15067E-09	2.40406E-07
TC01000424.hg.1	EIF3I	8668	eukaryotic translation initiation factor 3, subunit I	1.780184	2.14809E-07	7.66471E-06
TC10000469.hg.1	FUT11	170384	fucosyltransferase 11 (alpha (1,3) fucosyltransferase)	1.779886	1.22192E-05	0.000155949
TC11000395.hg.1	DGKZ	8525	diacylglycerol kinase, zeta	1.779321	1.16245E-10	7.15239E-08
TC02000139.hg.1	CENPO	79172	centromere protein O	1.777016	1.76988E-08	1.29934E-06
TC05000907.hg.1	HMMR	3161	hyaluronan-mediated motility receptor (RHAMM)	1.776856	1.18993E-06	2.61629E-05
TC05000334.hg.1	MCCC2	64087	methylcrotonoyl-CoA carboxylase 2 (beta)	1.776735	1.38608E-10	7.80118E-08
TC15001474.hg.1	RFX7	64864	regulatory factor X, 7	1.776538	2.02384E-06	3.92419E-05
TC17000804.hg.1	PRKAR1A	5573	protein kinase, cAMP-dependent, regulatory, type I, alpha	1.776265	0.000331313	0.002154589
TC04001805.hg.1	IRF2	3660	interferon regulatory factor 2	1.776086	8.01034E-05	0.000682497
TC17001812.hg.1	HELZ	9931	helicase with zinc finger	1.775744	5.81571E-06	8.855E-05
TC10001097.hg.1	DNAJC1	64215	DnaJ (Hsp40) homolog, subfamily C, member 1	1.77478	9.9302E-06	0.000132661
TC19001021.hg.1	AP3D1	8943	adaptor-related protein complex 3, delta 1 subunit	1.774101	4.68391E-08	2.59581E-06
TC07001995.hg.1	PDIA4	9601	protein disulfide isomerase family A, member 4	1.774095	0.000116738	0.000928219
TC12000239.hg.1	ETNK1	55500	ethanolamine kinase 1	1.771578	1.25654E-05	0.000159289
TC17001125.hg.1	STX8	9482	syntaxin 8	1.770733	0.001067191	0.005616999
TC08001280.hg.1	ARMC1	55156	armadillo repeat containing 1	1.76895	4.0273E-08	2.33598E-06
TC01002759.hg.1	SERBP1	26135	SERPINE1 mRNA binding protein 1	1.768517	0.000622007	0.003619493
TC19000479.hg.1	COX6B1	1340	cytochrome c oxidase subunit VIb polypeptide 1 (ubiquitous)	1.768325	4.63927E-07	1.30841E-05
TC03000051.hg.1	FANCD2	2177	Fanconi anemia, complementation group D2	1.767769	7.1407E-06	0.000103694
TC19000444.hg.1	UBA2	10054	ubiquitin-like modifier activating enzyme 2	1.767265	2.72582E-05	0.000291836

transcript cluster id	Gene Symbol	Entrez Gene ID	Description	FoldChange (AIM/control)	P.Value (AIM/control)	adj.P.Val (AIM/control)
TC06001044.hg.1	VTA1	51534	Vps20-associated 1 homolog (S. cerevisiae)	1.766626	2.31159E-06	4.33449E-05
TC15001549.hg.1	PPIB	5479	peptidylprolyl isomerase B (cyclophilin B)	1.76654	5.60795E-06	8.61581E-05
TC12000325.hg.1	IRAK4	51135	interleukin-1 receptor-associated kinase 4	1.766224	1.86678E-05	0.000216268
TC10000772.hg.1	TAF5	6877	TAF5 RNA polymerase II, TATA box binding protein (TBP)-associated factor, 100kDa	1.766216	1.95841E-08	1.3948E-06
TC05001050.hg.1	CANX	821	calnexin	1.765959	4.02523E-05	0.000395416
TC01001640.hg.1	PTPRC	5788	protein tyrosine phosphatase, receptor type, C	1.76251	8.97266E-06	0.000123265
TC19000416.hg.1	CCNE1	898	cyclin E1	1.76228	9.8076E-06	0.000131488
TC12000525.hg.1	SHMT2	6472	serine hydroxymethyltransferase 2 (mitochondrial)	1.761452	9.32814E-08	4.23292E-06
TC04000336.hg.1	POLR2B	5431	polymerase (RNA) II (DNA directed) polypeptide B, 140kDa	1.758628	2.35384E-08	1.57586E-06
TC19000088.hg.1	CHAF1A	10036	chromatin assembly factor 1, subunit A (p150)	1.75824	6.08891E-08	3.12252E-06
TC08000278.hg.1	PROSC	11212	proline synthetase co-transcribed homolog (bacterial)	1.757866	1.83816E-07	6.91304E-06
TC01001263.hg.1	INTS3	65123	integrator complex subunit 3	1.757448	0.0001971	0.001422738
TC17001786.hg.1	FTSJ3	117246	FtsJ homolog 3 (E. coli)	1.756966	2.27281E-07	7.99658E-06
TC09000693.hg.1	GLE1	2733	GLE1 RNA export mediator homolog (yeast)	1.755343	2.72813E-07	9.01115E-06
TC16000300.hg.1	SBK1	388228	SH3-binding domain kinase 1	1.755313	4.70713E-06	7.51438E-05
TC01000977.hg.1	ATP5F1	515	ATP synthase, H+ transporting, mitochondrial Fo complex, subunit B1	1.755228	5.45355E-07	1.46816E-05
TC19000414.hg.1	POP4	10775	processing of precursor 4, ribonuclease P/MRP subunit (S. cerevisiae)	1.754733	3.27363E-05	0.0003372
TC15000611.hg.1	DIS3L	115752	DIS3 mitotic control homolog (S. cerevisiae)-like	1.753325	4.49213E-07	1.28084E-05
TC18000565.hg.1	TMX3	54495	thioredoxin-related transmembrane protein 3	1.751019	9.93095E-06	0.000132727
TC02001665.hg.1	HADHA	3030	hydroxyacyl-CoA dehydrogenase/3-ketoacyl-CoA thiolase/enoyl-CoA hydratase (trifunctio	1.750736	6.78555E-06	9.98402E-05
TC20000055.hg.1	PCNA-AS1	100302739	PCNA antisense RNA 1	1.750113	0.000205908	0.001469434
TC20000271.hg.1	RPN2	6185	ribophorin II	1.749547	0.000799151	0.004440362
TC17000089.hg.1	ACAP1	9744	ArfGAP with coiled-coil, ankyrin repeat and PH domains 1	1.749462	7.17621E-05	0.00062535
TC19001215.hg.1	FARSA	2193	phenylalanyl-tRNA synthetase, alpha subunit	1.749329	4.33667E-07	1.25758E-05
TC0X000994.hg.1	NDUFB11	54539	NADH dehydrogenase (ubiquinone) 1 beta subcomplex, 11, 17.3kDa	1.748573	0.000107355	0.000867313
TC12002018.hg.1	TAOK3	51347	TAO kinase 3	1.748369	0.000294011	0.00195646
TC10000870.hg.1	SEC23IP	11196	SEC23 interacting protein	1.747358	1.03321E-07	4.54301E-06
TC07000338.hg.1	CCT6A	908	chaperonin containing TCP1, subunit 6A (zeta 1)	1.746817	2.08357E-06	4.02093E-05
TC06000149.hg.1	GMNN	51053	geminin, DNA replication inhibitor	1.746361	0.000143201	0.001098234
TC02000173.hg.1	SNX17	9784	sorting nexin 17	1.745945	2.11603E-08	1.47381E-06
TC12001331.hg.1	TM7SF3	51768	transmembrane 7 superfamily member 3	1.744895	2.00778E-06	3.90651E-05
TC19000015.hg.1	PTBP1	5725	polypyrimidine tract binding protein 1	1.74398	4.28317E-05	0.000415704
TC01001357.hg.1	SLAMF8	56833	SLAM family member 8	1.742945	2.51131E-05	0.000273239
TC14000141.hg.1	C14orf119	55017	chromosome 14 open reading frame 119	1.742212	3.06399E-05	0.000319296
TC0X000253.hg.1	EBP	10682	emopamil binding protein (sterol isomerase)	1.742125	2.0012E-09	3.53427E-07
TC14000605.hg.1	PAPOLA	10914	poly(A) polymerase alpha	1.741689	0.000123635	0.000974895
TC20000467.hg.1	NELFCD	51497	negative elongation factor complex member C/D	1.740922	2.40254E-05	0.000264254
TC16001284.hg.1	KARS	3735	lysyl-tRNA synthetase	1.740299	1.28206E-05	0.000161689
TC12003226.hg.1	FKBP11	51303	FK506 binding protein 11, 19 kDa	1.737808	0.001221986	0.006257569
TC11000412.hg.1	PTPRJ	5795	protein tyrosine phosphatase, receptor type, J	1.737364	1.03138E-05	0.000136509
TC14000326.hg.1	KTN1	3895	kinectin 1 (kinesin receptor)	1.73728	4.39029E-05	0.000424427
TC14001206.hg.1	PPP2R5E	5529	protein phosphatase 2, regulatory subunit B', epsilon isoform	1.736485	1.44022E-05	0.000177463
TC17002886.hg.1	TMEM256	254863	transmembrane protein 256	1.735919	0.000258028	0.001761228
TC07002027.hg.1	CDK5	1020	cyclin-dependent kinase 5	1.733773	3.75844E-07	1.12948E-05
TC17000188.hg.1	TRPV2	51393	transient receptor potential cation channel, subfamily V, member 2	1.73307	4.99029E-07	1.38224E-05
TC16000979.hg.1	GTF3C1	2975	general transcription factor IIIC, polypeptide 1, alpha 220kDa	1.731565	8.0377E-07	1.95603E-05
TC17001328.hg.1	BLMH	642	bleomycin hydrolase	1.730315	1.42872E-06	3.01855E-05
TC06004121.hg.1	ATF6B	1388	activating transcription factor 6 beta	1.729224	3.71446E-05	0.000370751
TC06000147.hg.1	ACOT13	55856	acyl-CoA thioesterase 13	1.729048	4.05536E-06	6.68883E-05
TC20000789.hg.1	GSS	2937	glutathione synthetase	1.726396	4.79328E-10	1.41553E-07
TC17001537.hg.1	FAM134C	162427	family with sequence similarity 134, member C	1.726125	6.62072E-06	9.8171E-05
TC09000495.hg.1	ANP32B	10541	acidic (leucine-rich) nuclear phosphoprotein 32 family, member B	1.725931	1.25758E-05	0.000159742
TC0X001173.hg.1	ATRX	546	alpha thalassemia/mental retardation syndrome X-linked	1.725556	0.003327878	0.01387101
TC06000421.hg.1	KIFC1	3833	kinesin family member C1	1.723853	1.48684E-08	1.17191E-06
TC01001838.hg.1	CAPN2	824	calpain 2, (m/II) large subunit	1.723586	4.13501E-05	0.000404233
TC11002133.hg.1	ALG8	79053	ALG8, alpha-1,3-glucosyltransferase	1.723242	2.71405E-06	4.9444E-05
TC17000849.hg.1	MRPS7	51081	mitochondrial ribosomal protein S7	1.723144	0.000272704	0.001839002
TC02001795.hg.1	LRPPRC	10128	leucine-rich pentatricopeptide repeat containing	1.721304	0.000391492	0.002478729
TC02001260.hg.1	ATIC	471	5-aminoimidazole-4-carboxamide ribonucleotide formyltransferase/IMP cyclohydrolase	1.721238	3.04453E-07	9.77468E-06
TC05001392.hg.1	DEPDC1B	55789	DEP domain containing 1B	1.721226	4.6306E-10	1.41553E-07
TC17001591.hg.1	DCAKD	79877	dephospho-CoA kinase domain containing	1.721099	3.15858E-06	5.51002E-05
TC10000797.hg.1	ADD3	120	adducin 3 (gamma)	1.717925	0.000508366	0.003066816
TC19000259.hg.1	MIR639	693224	microRNA 639	1.717266	0.000161471	0.00121065
TC19002704.hg.1	ATP5SL	55101	ATP5S-like	1.717258	2.25513E-05	0.000251256
TC06001425.hg.1	HIST1H2AK	8330	histone cluster 1, H2ak	1.71719	1.92178E-06	3.79369E-05
TC03000549.hg.1	TRAT1	50852	T cell receptor associated transmembrane adaptor 1	1.716815	0.002974786	0.01264384
TC06001355.hg.1	HIST1H4H	8365	histone cluster 1, H4h	1.71678	0.00338692	0.0140659
TC17000772.hg.1	PSMC5	5705	proteasome (prosome, macropain) 26S subunit, ATPase, 5	1.715911	8.3138E-07	2.00799E-05
TC12001597.hg.1	SMARCC2	6601	SWI/SNF related, matrix associated, actin dependent regulator of chromatin, subfamily c,	1.714762	1.67021E-05	0.000199198
TC06001586.hg.1	DAXX	1616	death-domain associated protein	1.713889	1.41368E-07	5.72933E-06
TC02002033.hg.1	SUCLG1	8802	succinate-CoA ligase, alpha subunit	1.713514	8.88576E-08	4.11651E-06
TC12000462.hg.1	COPZ1	22818	coatomer protein complex, subunit zeta 1	1.713087	7.51492E-09	7.7686E-07

transcript cluster id	Gene Symbol	Entrez Gene ID	Description	FoldChange (AIM/control)	P.Value (AIM/control)	adj.P.Val (AIM/control)
TC08000528.hg.1	LRRCC1	85444	leucine rich repeat and coiled-coil centrosomal protein 1	1.71301	1.91824E-05	0.000221647
TC12000946.hg.1	DYNLL1	8655	dynein, light chain, LC8-type 1	1.712816	1.53512E-06	3.20543E-05
TC01000641.hg.1	PRPF38A	84950	PRP38 pre-mRNA processing factor 38 (yeast) domain containing A	1.712299	1.10515E-06	2.49004E-05
TC16000439.hg.1	ADCY7	113	adenylate cyclase 7	1.712026	7.8247E-05	0.000669542
TC11000482.hg.1	FAM111B	374393	family with sequence similarity 111, member B	1.711742	0.002926238	0.01247827
TC03001448.hg.1	TLR9	54106	toll-like receptor 9	1.71168	8.68672E-08	4.06403E-06
TC10000476.hg.1	VCL	7414	vinculin	1.710467	0.002752633	0.01190198
TC02000089.hg.1	DDX1	1653	DEAD (Asp-Glu-Ala-Asp) box helicase 1	1.709819	5.58952E-05	0.000514662
TC01003354.hg.1	SH2D2A	9047	SH2 domain containing 2A	1.709787	1.5952E-05	0.000192494
TC12000010.hg.1	WNK1	65125	WNK lysine deficient protein kinase 1	1.709169	2.29398E-05	0.00025501
TC12001959.hg.1	GIT2	9815	G protein-coupled receptor kinase interacting ArfGAP 2	1.708123	0.000103255	0.000839979
TC10001615.hg.1	C10orf76	79591	chromosome 10 open reading frame 76	1.707856	1.75541E-06	3.54675E-05
TC12003214.hg.1	CNPY2	10330	canopy 2 homolog (zebrafish)	1.707633	3.12658E-05	0.000325239
TC05001223.hg.1	DROSHA	29102	drosha, ribonuclease type III	1.707495	1.94077E-07	7.17944E-06
TC09000149.hg.1	ANXA2P2	304	annexin A2 pseudogene 2	1.70717	0.001594889	0.007750741
TC01002604.hg.1	HECTD3	79654	HECT domain containing E3 ubiquitin protein ligase 3	1.70661	2.66694E-07	8.9165E-06
TC05001295.hg.1	FYB	2533	FYN binding protein	1.706306	0.000459169	0.002820414
TC10000933.hg.1	GLRX3	10539	glutaredoxin 3	1.7054	8.43582E-06	0.00011797
TC02002697.hg.1	NDUFS1	4719	NADH dehydrogenase (ubiquinone) Fe-S protein 1, 75kDa (NADH-coenzyme Q reductase)	1.70399	1.54657E-09	2.97728E-07
TC16000072.hg.1	CCNF	899	cyclin F	1.703033	2.67173E-10	1.05004E-07
TC14000773.hg.1	PPP2R5C	5527	protein phosphatase 2, regulatory subunit B', gamma	1.702917	4.69612E-06	7.52149E-05
TC14002343.hg.1	NEDD8	4738	neural precursor cell expressed, developmentally down-regulated 8	1.701584	6.53747E-08	3.31088E-06
TC22001468.hg.1	APOBEC3G	60489	apolipoprotein B mRNA editing enzyme, catalytic polypeptide-like 3G	1.700981	7.83962E-07	1.92671E-05
TC22001422.hg.1	DRG1	4733	developmentally regulated GTP binding protein 1	1.700827	5.52446E-07	1.48633E-05
TC0X000228.hg.1	UBA1	7317	ubiquitin-like modifier activating enzyme 1	1.700753	0.000393372	0.00248898
TC12001139.hg.1	MRPL51	51258	mitochondrial ribosomal protein L51	1.697279	2.15986E-06	4.14005E-05
TC04001808.hg.1	MLF1IP	79682	MLF1 interacting protein	1.696085	0.001954209	0.009089498
TC11001738.hg.1	PSMC3	5702	proteasome (prosome, macropain) 26S subunit, ATPase, 3	1.695922	1.42003E-08	1.14087E-06
TC14000630.hg.1	EVL	51466	Enah/Vasp-like	1.695509	0.000169935	0.001262627
TC12000507.hg.1	NABP2	79035	nucleic acid binding protein 2	1.695268	3.26187E-09	4.75086E-07
TC13000157.hg.1	DNAJC15	29103	DnaJ (Hsp40) homolog, subfamily C, member 15	1.695084	1.65376E-06	3.40157E-05
TC03001418.hg.1	UBA7	7318	ubiquitin-like modifier activating enzyme 7	1.694796	8.34709E-06	0.000117122
TC17001457.hg.1	MED24	9862	mediator complex subunit 24	1.694275	3.06003E-08	1.91609E-06
TC17001664.hg.1	PHB	5245	prohibitin	1.693221	9.30398E-09	8.73695E-07
TC03001624.hg.1	CBLB	868	Cbl proto-oncogene, E3 ubiquitin protein ligase B	1.692828	6.02374E-06	9.14257E-05
TC01002788.hg.1	CRYZ	1429	crystallin, zeta (quinone reductase)	1.69209	2.25166E-07	7.99658E-06
TC19001275.hg.1	BST2	684	bone marrow stromal cell antigen 2	1.691485	0.001063306	0.00560766
TC01001723.hg.1	IKBKE	9641	inhibitor of kappa light polypeptide gene enhancer in B-cells, kinase epsilon	1.690561	2.33025E-07	8.17777E-06
TC06004056.hg.1	GOPC	57120	golgi-associated PDZ and coiled-coil motif containing	1.69026	0.000201734	0.001447951
TC17000725.hg.1	CLTC	1213	clathrin, heavy chain (Hc)	1.689762	9.24962E-07	2.17226E-05
TC06000195.hg.1	HMGN4	10473	high mobility group nucleosomal binding domain 4	1.689516	3.26465E-06	5.65647E-05
TC09000099.hg.1	FOCAD	54914	focadhesin	1.689135	5.25722E-06	8.21274E-05
TC13001720.hg.1	BIVM	54841	basic, immunoglobulin-like variable motif containing	1.68839	1.22278E-07	5.1816E-06
TC07001250.hg.1	GGCT	79017	gamma-glutamylcyclotransferase	1.687452	1.28438E-07	5.34926E-06
TC09000492.hg.1	NCBP1	4686	nuclear cap binding protein subunit 1, 80kDa	1.68731	5.65738E-05	0.000520108
TC19001232.hg.1	ASF1B	55723	anti-silencing function 1B histone chaperone	1.687029	3.06051E-08	1.91609E-06
TC15000588.hg.1	SNX1	6642	sorting nexin 1	1.686606	7.31105E-08	3.55344E-06
TC01002679.hg.1	HSPB11	51668	heat shock protein family B (small), member 11	1.68655	1.21378E-08	1.0464E-06
TC09000893.hg.1	ERMP1	79956	endoplasmic reticulum metallopeptidase 1	1.68609	0.000473503	0.002894169
TC11002268.hg.1	KDELC2	143888	KDEL (Lys-Asp-Glu-Leu) containing 2	1.685562	2.38928E-06	4.46022E-05
TC19001166.hg.1	KEAP1	9817	kelch-like ECH-associated protein 1	1.685461	2.12657E-07	7.66471E-06
TC02002827.hg.1	DOCK10	55619	dedicator of cytokinesis 10	1.683764	0.004040059	0.01617961
TC04000430.hg.1	SEPT11	55752	septin 11	1.68371	3.28258E-06	0.000056696
TC15001557.hg.1	MTFMT	123263	mitochondrial methionyl-tRNA formyltransferase	1.683008	4.0913E-08	2.37451E-06
TC07000497.hg.1	RSBN1L	222194	round spermatid basic protein 1-like	1.682763	9.81993E-05	0.000806771
TC02005009.hg.1	SP140	11262	SP140 nuclear body protein	1.682652	0.000295433	0.001965986
TC16002086.hg.1	PSMB10	5699	proteasome (prosome, macropain) subunit, beta type, 10	1.682638	1.21292E-06	2.67253E-05
TC06001207.hg.1	EXOC2	55770	exocyst complex component 2	1.682403	0.000187261	0.001367169
TC16000427.hg.1	PHKB	5257	phosphorylase kinase, beta	1.6819	0.00548246	0.02074496
TC15000412.hg.1	TMOD3	29766	tropomodulin 3 (ubiquitous)	1.681061	1.14266E-05	0.000148262
TC17000362.hg.1	NF1	4763	neurofibromin 1	1.681	1.2392E-05	0.000158097
TC10001369.hg.1	PCBD1	5092	pterin-4 alpha-carbinolamine dehydratase/dimerization cofactor of hepatocyte nuclear fa	1.680488	5.83545E-08	3.04783E-06
TC19001730.hg.1	VRK3	51231	vaccinia related kinase 3	1.679609	8.63967E-07	2.07423E-05
TC01002985.hg.1	WDR77	79084	WD repeat domain 77	1.679552	5.74356E-07	1.5203E-05
TC16000686.hg.1	CDT1	81620	chromatin licensing and DNA replication factor 1	1.679335	7.96588E-07	1.95254E-05
TC0X001067.hg.1	HUWE1	10075	HECT, UBA and WWE domain containing 1, E3 ubiquitin protein ligase	1.678984	3.39677E-05	0.000346814
TC12000028.hg.1	FKBP4	2288	FK506 binding protein 4, 59kDa	1.678685	5.41194E-05	0.000501991
TC11000546.hg.1	INCENP	3619	inner centromere protein antigens 135/155kDa	1.678229	7.16867E-09	7.65983E-07
TC02002733.hg.1	LANCL1	10314	LanC lantibiotic synthetase component C-like 1 (bacterial)	1.677663	6.36662E-07	1.65198E-05
TC01001893.hg.1	HIST3H2BB	128312	histone cluster 3, H2bb	1.676935	0.000117964	0.000939122
TC07001880.hg.1	SLC35B4	84912	solute carrier family 35, member B4	1.676677	5.3976E-08	2.88636E-06
TC11002305.hg.1	ZW10	9183	zw10 kinetochore protein	1.676214	3.7177E-10	1.26663E-07

transcript cluster id	Gene Symbol	Entrez Gene ID	Description	FoldChange (AIM/control)	P.Value (AIM/control)	adj.P.Val (AIM/control)
TC04000319.hg.1	SRD5A3	79644	steroid 5 alpha-reductase 3	1.676092	2.63383E-07	8.90541E-06
TC02000500.hg.1	TMSB10	9168	thymosin beta 10	1.675947	4.46465E-06	7.23797E-05
TC09001664.hg.1	FAM78A	286336	family with sequence similarity 78, member A	1.6752	7.40687E-06	0.000107396
TC02005034.hg.1	MRPL53	116540	mitochondrial ribosomal protein L53	1.674349	3.34858E-05	0.000343723
TC15000685.hg.1	CSK	1445	c-src tyrosine kinase	1.674325	6.65989E-08	3.34488E-06
TC04000891.hg.1	TRAPPC11	60684	trafficking protein particle complex 11	1.674107	6.56491E-07	1.68552E-05
TC0X001358.hg.1	AIFM1	9131	apoptosis-inducing factor, mitochondrion-associated, 1	1.674086	2.00372E-08	1.43382E-06
TC01003636.hg.1	TPR	7175	translocated promoter region, nuclear basket protein	1.673736	0.000257402	0.001761004
TC09000781.hg.1	WDR5	11091	WD repeat domain 5	1.673734	0.000135992	0.00105563
TC05000697.hg.1	KIF20A	10112	kinesin family member 20A	1.6732	1.0072E-10	6.87196E-08
TC10001410.hg.1	PPP3CB	5532	protein phosphatase 3, catalytic subunit, beta isozyme	1.672522	1.00208E-05	0.000134062
TC01000101.hg.1	PARK7	11315	parkinson protein 7	1.672317	1.10156E-07	4.79566E-06
TC12000084.hg.1	GAPDH	2597	glyceraldehyde-3-phosphate dehydrogenase	1.670297	2.33482E-06	4.39811E-05
TC22001487.hg.1	THOC5	8563	THO complex 5	1.670261	2.27426E-07	8.06716E-06
TC13000468.hg.1	SKA3	221150	spindle and kinetochore associated complex subunit 3	1.670097	6.76324E-08	3.37462E-06
TC16001084.hg.1	VPS35	55737	vacuolar protein sorting 35 homolog (S. cerevisiae)	1.669065	5.76061E-05	0.000529083
TC11001945.hg.1	CFL1	1072	cofilin 1 (non-muscle)	1.668093	1.06477E-07	4.702E-06
TC06004150.hg.1	FKBP5	2289	FK506 binding protein 5	1.667889	0.0031589	0.01329271
TC13000367.hg.1	TPP2	7174	tripeptidyl peptidase II	1.66781	0.000146822	0.001121889
TC14000777.hg.1	DYNC1H1	1778	dynein, cytoplasmic 1, heavy chain 1	1.667791	4.73002E-06	7.58296E-05
TC01003623.hg.1	EDEM3	80267	ER degradation enhancer, mannosidase alpha-like 3	1.667538	2.81564E-05	0.000300238
TC04000935.hg.1	FRG1	2483	FSHD region gene 1	1.666589	1.72206E-06	3.5116E-05
TC07003288.hg.1	ARPC1B	10095	actin related protein 2/3 complex, subunit 1B, 41kDa	1.666497	6.96748E-05	0.000612755
TC18001006.hg.1	PSMG2	56984	proteasome (prosome, macropain) assembly chaperone 2	1.666337	1.54107E-06	3.22276E-05
TC10001580.hg.1	GOT1	2805	glutamic-oxaloacetic transaminase 1, soluble	1.665426	5.03031E-07	1.399E-05
TC05000908.hg.1	MAT2B	27430	methionine adenosyltransferase II, beta	1.665217	2.56367E-08	1.70875E-06
TC02000158.hg.1	TMEM214	54867	transmembrane protein 214	1.664148	2.58865E-09	4.10895E-07
TC03001384.hg.1	UQCRC1	7384	ubiquinol-cytochrome c reductase core protein I	1.663777	1.68826E-07	6.55813E-06
TC09001455.hg.1	IKBKAP	8518	inhibitor of kappa light polypeptide gene enhancer in B-cells, kinase complex-associated p	1.663169	2.92524E-05	0.000308524
TC12001277.hg.1	ERP27	121506	endoplasmic reticulum protein 27	1.662332	0.009006703	0.03079333
TC22000610.hg.1	TPST2	8459	tyrosylprotein sulfotransferase 2	1.66227	4.19597E-07	1.23832E-05
TC04001405.hg.1	ADH5	128	alcohol dehydrogenase 5 (class III), chi polypeptide	1.662036	5.95156E-07	1.56631E-05
TC01000302.hg.1	LYPLA2	11313	lysophospholipase II	1.661662	5.04545E-09	6.10658E-07
TC11001987.hg.1	CDK2AP2	10263	cyclin-dependent kinase 2 associated protein 2	1.660309	8.92536E-07	2.12461E-05
TC07002021.hg.1	GIMAP6	474344	GTPase, IMAP family member 6	1.65996	0.000480597	0.00293343
TC01002686.hg.1	SSBP3	23648	single stranded DNA binding protein 3	1.659026	6.53629E-07	1.68359E-05
TC03001690.hg.1	LRRC58	116064	leucine rich repeat containing 58	1.658388	0.000164976	0.001234325
TC06001751.hg.1	XPO5	57510	exportin 5	1.657469	1.45023E-06	3.07685E-05
TC04000968.hg.1	SLBP	7884	stem-loop binding protein	1.657339	3.81312E-06	6.39928E-05
TC11000624.hg.1	DPF2	5977	D4, zinc and double PHD fingers family 2	1.656852	2.61611E-08	1.72547E-06
TC07003320.hg.1	GLCCI1	113263	glucocorticoid induced transcript 1	1.656669	5.07989E-07	1.41274E-05
TC22000202.hg.1	UQCR10	29796	ubiquinol-cytochrome c reductase, complex III subunit X	1.655591	1.0632E-05	0.000140596
TC22000725.hg.1	RAC2	5880	ras-related C3 botulinum toxin substrate 2 (rho family, small GTP binding protein Rac2)	1.655568	5.99108E-05	0.000545019
TC11000360.hg.1	API5	8539	apoptosis inhibitor 5	1.654968	0.000106858	0.000867074
TC01003702.hg.1	UBE2T	29089	ubiquitin-conjugating enzyme E2T (putative)	1.654895	2.07315E-07	7.59018E-06
TC13000518.hg.1	SLC46A3	283537	solute carrier family 46, member 3	1.653332	4.01292E-05	0.000396161
TC04001450.hg.1	TBCK	93627	TBC1 domain containing kinase	1.6532	0.000608225	0.00356442
TC15001505.hg.1	ANXA2	302	annexin A2	1.65313	0.001792854	0.008513003
TC12001599.hg.1	ANKRD52	283373	ankyrin repeat domain 52	1.652423	3.04292E-07	9.8224E-06
TC16000915.hg.1	ARL6IP1	23204	ADP-ribosylation factor-like 6 interacting protein 1	1.651887	8.43163E-06	0.000118351
TC03001487.hg.1	FAM208A	23272	family with sequence similarity 208, member A	1.651507	0.000628314	0.003655137
TC06000175.hg.1	HIST1H4F	8361	histone cluster 1, H4f	1.651206	9.18385E-06	0.000125939
TC03001227.hg.1	SGOL1	151648	shugoshin-like 1 (S. pombe)	1.651202	1.77333E-05	0.000209174
TC20000303.hg.1	PLCG1	5335	phospholipase C, gamma 1	1.650521	0.000100545	0.000823083
TC16000822.hg.1	TRAP1	10131	TNF receptor-associated protein 1	1.650376	1.08608E-05	0.000142903
TC07001861.hg.1	TMEM209	84928	transmembrane protein 209	1.650142	1.26116E-05	0.000160447
TC03003339.hg.1	CLDND1	56650	claudin domain containing 1	1.6499	5.45618E-06	8.49227E-05
TC03001723.hg.1	SNX4	8723	sorting nexin 4	1.64901	2.54817E-06	4.7147E-05
TC19000996.hg.1	POLR2E	5434	polymerase (RNA) II (DNA directed) polypeptide E, 25kDa	1.648968	1.10761E-05	0.000145036
TC09000607.hg.1	CNTRL	11064	centriolin	1.648926	0.000260188	0.001776488
TC13000539.hg.1	HSPH1	10808	heat shock 105kDa/110kDa protein 1	1.648909	0.000160433	0.001207439
TC06000685.hg.1	PRIM2	5558	primase, DNA, polypeptide 2 (58kDa)	1.648547	2.98381E-07	9.74155E-06
TC09001059.hg.1	TLN1	7094	talin 1	1.647467	0.000155094	0.001175081
TC11001835.hg.1	MRPL16	54948	mitochondrial ribosomal protein L16	1.647306	7.00198E-07	1.77449E-05
TC10000181.hg.1	APBB1IP	54518	amyloid beta (A4) precursor protein-binding, family B, member 1 interacting protein	1.646701	7.01628E-05	0.000616917
TC11003470.hg.1	CHEK1	1111	checkpoint kinase 1	1.646269	5.16135E-06	8.10877E-05
TC05000649.hg.1	HSPA4	3308	heat shock 70kDa protein 4	1.645995	2.146E-07	7.75225E-06
TC17000332.hg.1	ERAL1	26284	Era-like 12S mitochondrial rRNA chaperone 1	1.645875	1.08876E-07	4.78189E-06
TC12001002.hg.1	BRI3BP	140707	BRI3 binding protein	1.645592	2.34295E-06	4.41178E-05
TC12001138.hg.1	VAMP1	6843	vesicle-associated membrane protein 1 (synaptobrevin 1)	1.645571	0.000365337	0.002343862
TC11002334.hg.1	PCSK7	9159	proprotein convertase subtilisin/kexin type 7	1.644862	6.87943E-07	1.75368E-05
TC03001757.hg.1	RPN1	6184	ribophorin I	1.644742	1.07772E-06	2.45626E-05

transcript cluster id	Gene Symbol	Entrez Gene ID	Description	FoldChange (AIM/control)	P.Value (AIM/control)	adj.P.Val (AIM/control)
TC01000663.hg.1	MRPL37	51253	mitochondrial ribosomal protein L37	1.644737	0.000214858	0.001524181
TC0X000911.hg.1	RPS6KA3	6197	ribosomal protein S6 kinase, 90kDa, polypeptide 3	1.644609	8.11367E-05	0.000692431
TC02002745.hg.1	BARD1	580	BRCA1 associated RING domain 1	1.64457	2.47498E-07	8.524E-06
TC15000292.hg.1	RAD51	5888	RAD51 homolog (S. cerevisiae)	1.644076	2.27028E-08	1.57051E-06
TC17000104.hg.1	MPDU1	9526	mannose-P-dolichol utilization defect 1	1.642935	5.50068E-07	1.49056E-05
TC17001295.hg.1	POLDIP2	26073	polymerase (DNA-directed), delta interacting protein 2	1.642769	4.28495E-07	1.25758E-05
TC02001119.hg.1	MFSD6	54842	major facilitator superfamily domain containing 6	1.642274	1.74398E-05	0.000206757
TC19000720.hg.1	RUVBL2	10856	RuvB-like 2 (E. coli)	1.642223	6.62896E-08	3.34622E-06
TC01001588.hg.1	TSEN15	116461	tRNA splicing endonuclease 15 homolog (S. cerevisiae)	1.641639	2.74795E-06	5.00801E-05
TC01001101.hg.1	GPR89B	51463	G protein-coupled receptor 89C -RefSeq NM_001097616 -Entrez_id 728932 (This record v	1.641606	1.69184E-05	0.000201829
TC02001755.hg.1	HNRNPLL	92906	heterogeneous nuclear ribonucleoprotein L-like	1.64157	0.00170944	0.008202761
TC10000726.hg.1	FAM178A	55719	family with sequence similarity 178, member A	1.641051	1.7183E-06	3.51214E-05
TC06000937.hg.1	ASF1A	25842	anti-silencing function 1A histone chaperone	1.640892	1.05805E-05	0.000140343
TC11000249.hg.1	ZDHHC13	54503	zinc finger, DHHC-type containing 13	1.640856	4.74401E-07	1.34536E-05
TC12000334.hg.1	PCED1B	91523	PC-esterase domain containing 1B	1.640638	0.01207513	0.0388395
TC02001592.hg.1	NBAS	51594	neuroblastoma amplified sequence	1.639541	8.69389E-05	0.000729747
TC05001408.hg.1	CENPK	64105	centromere protein K	1.639378	0.000132726	0.001036254
TC21000111.hg.1	SOD1	6647	superoxide dismutase 1, soluble	1.638467	0.001400085	0.007008936
TC04001376.hg.1	PIGY	84992	phosphatidylinositol glycan anchor biosynthesis, class Y	1.638104	0.000179175	0.001320855
TC0X000589.hg.1	NDUFA1	4694	NADH dehydrogenase (ubiquinone) 1 alpha subcomplex, 1, 7.5kDa	1.637264	2.47087E-06	4.60213E-05
TC06000144.hg.1	MRS2	57380	MRS2 magnesium transporter	1.63664	8.72385E-07	2.09679E-05
TC15001249.hg.1	NDUFAF1	51103	NADH dehydrogenase (ubiquinone) complex I, assembly factor 1	1.636411	2.22418E-06	4.25354E-05
TC09002916.hg.1	FKBP15	23307	FK506 binding protein 15, 133kDa	1.636409	8.56719E-05	0.000721556
TC07001852.hg.1	TNPO3	23534	transportin 3	1.636398	1.64402E-09	3.14273E-07
TC16000533.hg.1	E2F4	1874	E2F transcription factor 4, p107/p130-binding	1.636284	5.98328E-05	0.000545019
TC01002175.hg.1	ENO1	2023	enolase 1, (alpha)	1.636105	0.000268639	0.001823002
TC06001293.hg.1	ATXN1	6310	ataxin 1	1.635993	6.97405E-05	0.000613885
TC17001770.hg.1	INTS2	57508	integrator complex subunit 2	1.635628	3.48425E-06	5.98079E-05
TC14001492.hg.1	C14orf64	388011	chromosome 14 open reading frame 64	1.635183	0.009104434	0.03104634
TC12001435.hg.1	RPAP3	79657	RNA polymerase II associated protein 3	1.634907	1.96187E-05	0.000226459
TC02001893.hg.1	XPO1	7514	exportin 1 (CRM1 homolog, yeast)	1.634695	2.33158E-05	0.000259527
TC06000226.hg.1	MIR3143	100422934	microRNA 3143	1.633698	1.75647E-05	0.000208037
TC16002056.hg.1	NFATC3	4775	nuclear factor of activated T-cells, cytoplasmic, calcineurin-dependent 3	1.633025	3.60708E-05	0.00036403
TC11000657.hg.1	RAB1B	81876	RAB1B, member RAS oncogene family	1.632474	8.27866E-06	0.000117013
TC12001445.hg.1	ASB8	140461	ankyrin repeat and SOCS box containing 8	1.632459	7.31031E-05	0.000638021
TC12001968.hg.1	PPP1CC	5501	protein phosphatase 1, catalytic subunit, gamma isozyme	1.63194	0.000215923	0.001529992
TC05002103.hg.1	LMAN2	10960	lectin, mannose-binding 2	1.631842	3.39959E-08	2.10597E-06
TC16001220.hg.1	PDF	64146	peptide deformylase (mitochondrial)	1.631115	1.5608E-06	3.26387E-05
TC17001199.hg.1	FLII	2314	flightless I homolog (Drosophila)	1.63093	1.61144E-08	1.25372E-06
TC17000137.hg.1	RANGRF	29098	RAN guanine nucleotide release factor	1.630815	4.32728E-08	2.49715E-06
TC15000864.hg.1	IQGAP1	8826	IQ motif containing GTPase activating protein 1	1.630486	0.007330173	0.02613765
TC16000960.hg.1	COG7	91949	component of oligomeric golgi complex 7	1.630051	8.08909E-09	8.14718E-07
TC02001414.hg.1	INPP5D	3635	inositol polyphosphate-5-phosphatase, 145kDa	1.630049	1.07388E-05	0.000141905
TC01002475.hg.1	YARS	8565	tyrosyl-tRNA synthetase	1.62973	1.71974E-05	0.000204643
TC15001570.hg.1	VWA9	81556	von Willebrand factor A domain containing 9	1.629156	2.44356E-06	4.57449E-05
TC16000548.hg.1	NUTF2	10204	nuclear transport factor 2	1.628191	2.49445E-06	4.6468E-05
TC17000972.hg.1	YWHAE	7531	tyrosine 3-monooxygenase/tryptophan 5-monooxygenase activation protein, epsilon poly	1.627997	2.87126E-07	9.5031E-06
TC07000782.hg.1	CALU	813	calumenin	1.627775	0.000129794	0.001018581
TC07000397.hg.1	INTS4L2	644619	integrator complex subunit 4-like 2	1.627424	0.000114135	0.000915536
TC01002147.hg.1	ICMT	23463	isoprenylcysteine carboxyl methyltransferase	1.626948	2.01592E-08	1.45288E-06
TC17000611.hg.1	KPNB1	3837	karyopherin (importin) beta 1	1.625513	3.46816E-06	5.96509E-05
TC18000088.hg.1	SNRPD1	6632	small nuclear ribonucleoprotein D1 polypeptide 16kDa	1.624539	0.004345683	0.017185
TC07001005.hg.1	GIMAP2	26157	GTPase, IMAP family member 2	1.622751	0.000396836	0.00251487
TC16000542.hg.1	CTCF	10664	CCCTC-binding factor (zinc finger protein)	1.622567	1.38732E-05	0.000173401
TC17001970.hg.1	DCXR	51181	dicarbonyl/L-xylulose reductase	1.622299	0.000383283	0.002440171
TC01002652.hg.1	NRD1	4898	nardilysin (N-arginine dibasic convertase)	1.621168	0.002119592	0.009694031
TC14002293.hg.1	KIAA0391	9692	KIAA0391	1.621151	2.12718E-06	4.11889E-05
TC01002197.hg.1	MTOR	2475	mechanistic target of rapamycin (serine/threonine kinase)	1.620754	1.04156E-07	4.66013E-06
TC05001005.hg.1	TSPAN17	26262	tetraspanin 17	1.619893	8.93573E-07	2.1315E-05
TC19001204.hg.1	DHPS	1725	deoxyhypusine synthase	1.619537	3.5742E-05	0.0003617
TC01003114.hg.1	GPR89A	653519	G protein-coupled receptor 89A	1.619489	2.004E-05	0.000230269
TC08002599.hg.1	FNTA	2339	farnesyltransferase, CAAX box, alpha	1.619397	2.56885E-05	0.000279862
TC22000709.hg.1	TXN2	25828	thioredoxin 2	1.619339	3.45649E-07	1.07391E-05
TC17001626.hg.1	OSBPL7	114881	oxysterol binding protein-like 7	1.618868	8.65727E-06	0.000121051
TC12003230.hg.1	TULP3	7289	tubby like protein 3	1.618714	9.58901E-07	2.25167E-05
TC22001472.hg.1	ADSL	158	adenylosuccinate lyase	1.618155	8.85046E-06	0.000123053
TC01002189.hg.1	LZIC	84328	leucine zipper and CTNNBIP1 domain containing	1.618123	2.48812E-07	8.56908E-06
TC10000724.hg.1	HIF1AN	55662	hypoxia inducible factor 1, alpha subunit inhibitor	1.618004	9.58648E-07	2.25167E-05
TC06001034.hg.1	ABRACL	58527	ABRA C-terminal like	1.616759	1.79285E-06	3.64274E-05
TC17000459.hg.1	MRPL45	84311	mitochondrial ribosomal protein L45	1.615859	4.49558E-06	7.32832E-05
TC0X000370.hg.1	IGBP1	3476	immunoglobulin (CD79A) binding protein 1	1.615495	2.14586E-06	4.14762E-05
TC17001015.hg.1	ATP2A3	489	ATPase, Ca++ transporting, ubiquitous	1.615107	2.12523E-05	0.000241871

transcript cluster id	Gene Symbol	Entrez Gene ID	Description	FoldChange (AIM/control)	P.Value (AIM/control)	adj.P.Val (AIM/control)
TC15000592.hg.1	TRIP4	9325	thyroid hormone receptor interactor 4	1.614766	5.91316E-09	6.74671E-07
TC20000726.hg.1	APMAP	57136	adipocyte plasma membrane associated protein	1.614673	0.000876587	0.004809984
TC19001098.hg.1	DENND1C	79958	DENN/MADD domain containing 1C	1.614208	2.35303E-06	4.44127E-05
TC03003376.hg.1	HIGD1A	25994	HIG1 hypoxia inducible domain family, member 1A	1.613971	9.21763E-06	0.000126477
TC20000337.hg.1	YWHAB	7529	tyrosine 3-monooxygenase/tryptophan 5-monooxygenase activation protein, beta polype	1.613882	0.000113359	0.000910433
TC11000620.hg.1	CAPN1	823	calpain 1, (mu/I) large subunit	1.613129	4.02884E-06	6.71394E-05
TC11001795.hg.1	UBE2L6	9246	ubiquitin-conjugating enzyme E2L 6	1.61311	0.001926425	0.009005914
TC03001998.hg.1	TNIK	23043	TRAF2 and NCK interacting kinase	1.613008	0.001150135	0.005977455
TC16000891.hg.1	NTAN1	123803	N-terminal asparagine amidase	1.610995	2.55546E-05	0.000278885
TC02000721.hg.1	PSD4	23550	pleckstrin and Sec7 domain containing 4	1.609547	9.08206E-06	0.000125369
TC01003174.hg.1	FAM72C	554282	family with sequence similarity 72, member C	1.609174	1.81736E-05	0.000213833
TC11000586.hg.1	FERMT3	83706	fermitin family member 3	1.607843	0.000581902	0.003439686
TC21000260.hg.1	PCNT	5116	pericentrin	1.607587	3.75649E-05	0.00037651
TC03000766.hg.1	ATP1B3	483	ATPase, Na+/K+ transporting, beta 3 polypeptide	1.607306	0.007071696	0.02544585
TC17000600.hg.1	NSF	4905	N-ethylmaleimide-sensitive factor	1.60633	0.000552999	0.003302884
TC19000663.hg.1	PPP5C	5536	protein phosphatase 5, catalytic subunit	1.60384	1.76673E-08	1.34408E-06
TC10001069.hg.1	RSU1	6251	Ras suppressor protein 1	1.603812	0.000269094	0.001827131
TC01003400.hg.1	COPA	1314	coatomer protein complex, subunit alpha	1.603651	2.55754E-08	1.72543E-06
TC02001864.hg.1	SMEK2	57223	SMEK homolog 2, suppressor of mek1 (Dictyostelium)	1.603412	1.55959E-06	3.27184E-05
TC02002104.hg.1	STARD7	56910	StAR-related lipid transfer (START) domain containing 7	1.60283	0.000103112	0.000842933
TC17001147.hg.1	ELAC2	60528	elaC ribonuclease Z 2	1.602414	3.63078E-06	6.20388E-05
TC05000999.hg.1	HIGD2A	192286	HIG1 hypoxia inducible domain family, member 2A	1.602406	0.000220257	0.001553851
TC16000565.hg.1	SNTB2	6645	syntrophin, beta 2 (dystrophin-associated protein A1, 59kDa, basic component 2)	1.602353	5.63215E-06	8.73781E-05
TC0X001065.hg.1	HSD17B10	3028	hydroxysteroid (17-beta) dehydrogenase 10	1.601048	8.39166E-08	4.03576E-06
TC08001071.hg.1	KCTD9	54793	potassium channel tetramerisation domain containing 9	1.600952	5.32051E-05	0.000497475
TC12001443.hg.1	SENP1	29843	SUMO1/sentrin specific peptidase 1	1.600708	9.62348E-07	2.26204E-05
TC17000921.hg.1	RPTOR	57521	regulatory associated protein of MTOR, complex 1	1.600499	1.67057E-05	0.000200784
TC02001171.hg.1	NDUFB3	4709	NADH dehydrogenase (ubiquinone) 1 beta subcomplex, 3, 12kDa	1.600349	0.000145578	0.001117767
TC01000990.hg.1	CAPZA1	829	capping protein (actin filament) muscle Z-line, alpha 1	1.600026	5.5446E-06	8.62961E-05
TC11000483.hg.1	FAM111A	63901	family with sequence similarity 111, member A	1.599458	2.90931E-05	0.000308479
TC17001053.hg.1	SCIMP	388325	SLP adaptor and CSK interacting membrane protein	1.599177	0.01293808	0.04107351
TC20000542.hg.1	CSNK2A1	1457	casein kinase 2, alpha 1 polypeptide	1.599107	8.68598E-05	0.000730331
TC01002511.hg.1	MRPS15	64960	mitochondrial ribosomal protein S15	1.598797	5.53466E-06	8.62609E-05
TC17000714.hg.1	RAD51C	5889	RAD51 homolog C (S. cerevisiae)	1.598505	6.20109E-07	1.6355E-05
TC01002632.hg.1	FOXD2-AS1	84793	FOXD2 antisense RNA 1 (head to head)	1.598464	4.06146E-05	0.000401198
TC17001553.hg.1	BRCA1	672	breast cancer 1, early onset	1.598221	4.89932E-08	2.74221E-06
TC12001278.hg.1	ARHGDIB	397	Rho GDP dissociation inhibitor (GDI) beta	1.597606	0.000246001	0.001701187
TC14000941.hg.1	HAUS4	54930	HAUS augmin-like complex, subunit 4	1.596366	5.8396E-05	0.000537374
TC22000859.hg.1	TTLL12	23170	tubulin tyrosine ligase-like family, member 12	1.596294	2.08243E-06	4.06144E-05
TC06000412.hg.1	SLC39A7	7922	solute carrier family 39 (zinc transporter), member 7	1.596095	1.12543E-07	4.95383E-06
TC0Y000014.hg.1	CD99	4267	CD99 molecule	1.595983	3.71229E-07	1.13865E-05
TC0X000530.hg.1	ATG4A	115201	autophagy related 4A, cysteine peptidase	1.595846	2.16579E-08	1.53649E-06
TC08001723.hg.1	TSTA3	7264	tissue specific transplantation antigen P35B	1.595422	1.80565E-07	6.93873E-06
TC19002690.hg.1	COPE	11316	coatomer protein complex, subunit epsilon	1.595404	2.70538E-05	0.00029271
TC11001064.hg.1	KMT2A	4297	lysine (K)-specific methyltransferase 2A	1.595242	0.000198049	0.001434325
TC07000479.hg.1	MDH2	4191	malate dehydrogenase 2, NAD (mitochondrial)	1.594696	2.71151E-07	9.13495E-06
TC11001005.hg.1	DLAT	1737	dihydrolipoamide S-acetyltransferase	1.594582	2.22802E-06	4.28198E-05
TC11000325.hg.1	NAT10	55226	N-acetyltransferase 10 (GCN5-related)	1.594403	0.000158282	0.001197638
TC04000044.hg.1	HTT	3064	huntingtin	1.594309	9.49778E-08	4.37774E-06
TC20000104.hg.1	SNRPB2	6629	small nuclear ribonucleoprotein polypeptide B	1.594013	3.39669E-05	0.000348563
TC08001086.hg.1	PBK	55872	PDZ binding kinase	1.593275	1.65076E-06	3.43174E-05
TC16001314.hg.1	COTL1	23406	coactosin-like 1 (Dictyostelium)	1.592368	0.001116973	0.005829365
TC19001703.hg.1	GYS1	2997	glycogen synthase 1 (muscle)	1.59216	2.35209E-09	4.0102E-07
TC05000928.hg.1	SPDL1	54908	spindle apparatus coiled-coil protein 1	1.590704	1.50076E-09	2.99739E-07
TC01003617.hg.1	ARPC5	10092	actin related protein 2/3 complex, subunit 5, 16kDa	1.590316	1.97731E-05	0.000228319
TC08000547.hg.1	DECR1	1666	2,4-dienoyl CoA reductase 1, mitochondrial	1.58895	9.94646E-08	4.53006E-06
TC14002205.hg.1	AP1G2	8906	adaptor-related protein complex 1, gamma 2 subunit	1.588827	6.83807E-05	0.000605406
TC11000795.hg.1	SPCS2	9789	signal peptidase complex subunit 2 homolog (S. cerevisiae)	1.588696	0.000295898	0.001973557
TC12000541.hg.1	TSPAN31	6302	tetraspanin 31	1.588311	1.99686E-05	0.000230269
TC15001567.hg.1	DPP8	54878	dipeptidyl-peptidase 8	1.587948	2.02783E-06	3.99103E-05
TC07002054.hg.1	PAXIP1	22976	PAX interacting (with transcription-activation domain) protein 1	1.587364	4.49244E-08	2.59581E-06
TC01001118.hg.1	GPR89B	51463	G protein-coupled receptor 89C -RefSeq NM_001097616 -Entrez_id 728932 (This record w	1.586098	7.05462E-05	0.000621696
TC10001531.hg.1	IDE	3416	insulin-degrading enzyme	1.585806	1.66589E-05	0.00020063
TC12001627.hg.1	DCTN2	10540	dynactin 2 (p50)	1.58568	4.48642E-07	1.30261E-05
TC14001497.hg.1	BCL11B	64919	B-cell CLL/lymphoma 11B (zinc finger protein)	1.585493	0.008765406	0.0301772
TC10001362.hg.1	PPA1	5464	pyrophosphatase (inorganic) 1	1.584865	0.000750231	0.004244962
TC15001561.hg.1	CLPX	10845	ClpX caseinolytic peptidase X homolog (E. coli)	1.584695	5.30005E-06	8.35449E-05
TC08000767.hg.1	EFR3A	23167	EFR3 homolog A (S. cerevisiae)	1.584265	1.26121E-05	0.00016124
TC19001051.hg.1	MATK	4145	megakaryocyte-associated tyrosine kinase	1.584124	0.000220501	0.001555754
TC01000394.hg.1	EPB41	2035	erythrocyte membrane protein band 4.1 (elliptocytosis 1, RH-linked)	1.583535	0.000335196	0.002186372
TC14001170.hg.1	EXOC5	10640	exocyst complex component 5	1.582591	1.6415E-05	0.000198123
TC20000178.hg.1	FRG1B	284802	FSHD region gene 1 family, member B	1.582567	4.8219E-05	0.000459828

transcript cluster id	Gene Symbol	Entrez Gene ID	Description	FoldChange (AIM/control)	P.Value (AIM/control)	adj.P.Val (AIM/control)
TC01001840.hg.1	LOC100287497	100287497	uncharacterized LOC100287497	1.58218	0.006501654	0.0238476
TC18000493.hg.1	IER3IP1	51124	immediate early response 3 interacting protein 1	1.581578	1.99164E-06	3.9381E-05
TC02004930.hg.1	MRPS5	64969	mitochondrial ribosomal protein S5	1.581485	1.11322E-06	2.53599E-05
TC22000840.hg.1	NDUFA6	4700	NADH dehydrogenase (ubiquinone) 1 alpha subcomplex, 6, 14kDa	1.581156	4.57459E-06	7.45748E-05
TC01006356.hg.1	EFCAB14	9813	EF-hand calcium binding domain 14	1.58072	0.000955152	0.005148236
TC0X001312.hg.1	SEPT6	23157	septin 6	1.580192	0.006883956	0.02489947
TC19001628.hg.1	SNRPD2	6633	small nuclear ribonucleoprotein D2 polypeptide 16.5kDa	1.580126	9.90907E-06	0.000133873
TC0X001237.hg.1	TCEAL8	90843	transcription elongation factor A (SII)-like 8	1.579942	2.10257E-07	7.71846E-06
TC14001372.hg.1	SEL1L	6400	sel-1 suppressor of lin-12-like (C. elegans)	1.579816	1.00815E-05	0.000135541
TC02000213.hg.1	BIRC6	57448	baculoviral IAP repeat containing 6	1.579615	3.4874E-05	0.000356429
TC17000022.hg.1	SRR	63826	serine racemase	1.579429	1.71939E-07	6.74689E-06
TC01001591.hg.1	C1orf21	81563	chromosome 1 open reading frame 21	1.579375	1.79623E-05	0.000212656
TC19001635.hg.1	SYMPK	8189	symplekin	1.579256	9.77644E-08	4.47447E-06
TC01001149.hg.1	PPIAL4D	645142	peptidylprolyl isomerase A (cyclophilin A)-like 4D	1.579221	3.76163E-06	6.39358E-05
TC01003149.hg.1	PPIAL4D	645142	peptidylprolyl isomerase A (cyclophilin A)-like 4D	1.579221	3.76163E-06	6.39358E-05
TC13000640.hg.1	ZC3H13	23091	zinc finger CCCH-type containing 13	1.578767	6.97834E-06	0.000103278
TC17000530.hg.1	MLX	6945	MLX, MAX dimerization protein	1.577925	0.000127244	0.001005341
TC11001185.hg.1	FLI1	2313	Friend leukemia virus integration 1	1.577465	0.000417613	0.002622417
TC08000840.hg.1	CYC1	1537	cytochrome c-1	1.577208	6.58924E-06	9.89775E-05
TC01001012.hg.1	VANGL1	81839	VANGL planar cell polarity protein 1	1.57685	5.05001E-07	1.42195E-05
TC05000377.hg.1	AGGF1	55109	angiogenic factor with G patch and FHA domains 1	1.576653	6.93727E-06	0.000102838
TC11000404.hg.1	DDB2	1643	damage-specific DNA binding protein 2, 48kDa	1.57627	1.54657E-05	0.000189294
TC09002905.hg.1	NIPSNAP3A	25934	nipsnap homolog 3A (C. elegans)	1.576245	3.60891E-06	6.19886E-05
TC14001499.hg.1	SETD3	84193	SET domain containing 3	1.576214	1.54177E-06	3.25867E-05
TC19001125.hg.1	ELAVL1	1994	ELAV (embryonic lethal, abnormal vision, Drosophila)-like 1 (Hu antigen R)	1.57613	8.2063E-07	2.01943E-05
TC17000899.hg.1	BIRC5	332	baculoviral IAP repeat containing 5	1.575438	2.0696E-06	4.05367E-05
TC02002867.hg.1	PDE6D	5147	phosphodiesterase 6D, cGMP-specific, rod, delta	1.574714	1.57276E-07	6.32717E-06
TC0X001518.hg.1	IDH3G	3421	isocitrate dehydrogenase 3 (NAD+) gamma	1.574634	8.081E-07	1.99973E-05
TC19000971.hg.1	TRIM28	10155	tripartite motif containing 28	1.574625	0.00051504	0.00311228
TC10000964.hg.1	LARP4B	23185	La ribonucleoprotein domain family, member 4B	1.574076	0.000485451	0.002965631
TC02002948.hg.1	HDLBP	3069	high density lipoprotein binding protein	1.574049	2.68175E-07	9.10159E-06
TC02005053.hg.1	NHEJ1	79840	nonhomologous end-joining factor 1	1.57393	9.63375E-06	0.000131374
TC04001272.hg.1	GRSF1	2926	G-rich RNA sequence binding factor 1	1.57341	2.17486E-05	0.000246579
TC02001240.hg.1	RPE	6120	ribulose-5-phosphate-3-epimerase	1.573203	5.35004E-05	0.000500667
TC19001569.hg.1	GSK3A	2931	glycogen synthase kinase 3 alpha	1.572847	6.09954E-07	1.62065E-05
TC19001717.hg.1	PIH1D1	55011	PIH1 domain containing 1	1.57234	1.53464E-09	3.05244E-07
TC09000204.hg.1	CLTA	1211	clathrin, light chain A	1.572289	5.61199E-07	1.51872E-05
TC16000320.hg.1	LOC606724	606724	coronin, actin binding protein, 1A pseudogene	1.572129	5.40696E-08	2.9444E-06
TC12001154.hg.1	SCARNA12	677777	small Cajal body-specific RNA 12	1.570581	0.002562415	0.01126567
TC10000022.hg.1	PFKP	5214	phosphofructokinase, platelet	1.569719	1.93652E-05	0.000225513
TC11000820.hg.1	C11orf30	56946	chromosome 11 open reading frame 30	1.569101	2.27329E-05	0.000255274
TC16000216.hg.1	C16orf62	57020	chromosome 16 open reading frame 62	1.569041	0.000451539	0.002794875
TC11003489.hg.1	PTPRCAP	5790	protein tyrosine phosphatase, receptor type, C-associated protein	1.56902	1.26048E-06	2.77251E-05
TC12003276.hg.1	PRKAG1	5571	protein kinase, AMP-activated, gamma 1 non-catalytic subunit	1.56902	1.39601E-07	5.78554E-06
TC01001198.hg.1	PRUNE	58497	prune exopolyphosphatase	1.568728	2.84239E-05	0.000303767
TC09000448.hg.1	FAM120A	23196	family with sequence similarity 120A	1.568617	6.69781E-07	1.73561E-05
TC08000839.hg.1	GPAA1	8733	glycosylphosphatidylinositol anchor attachment 1	1.568126	8.19084E-06	0.000116717
TC01003108.hg.1	POLR3GL	84265	polymerase (RNA) III (DNA directed) polypeptide G (32kD)-like	1.567996	4.49805E-06	7.36929E-05
TC07003396.hg.1	TRRAP	8295	transformation/transcription domain-associated protein	1.567844	3.31416E-05	0.000343614
TC0X002306.hg.1	P2RY8	286530	purinergic receptor P2Y, G-protein coupled, 8	1.567827	0.008819948	0.03032445
TC0Y000348.hg.1	P2RY8	286530	purinergic receptor P2Y, G-protein coupled, 8	1.567827	0.008819948	0.03032445
TC22000385.hg.1	ATXN10	25814	ataxin 10	1.567585	2.48619E-05	0.000273729
TC01003916.hg.1	HIST3H2A	92815	histone cluster 3, H2a	1.567114	6.66935E-05	0.000594502
TC09001673.hg.1	SETX	23064	senataxin	1.566974	0.000665476	0.003844825
TC04001797.hg.1	RWDD4	201965	RWD domain containing 4	1.566533	1.54795E-05	0.00018953
TC01001146.hg.1	PPIAL4E	730262	peptidylprolyl isomerase A (cyclophilin A)-like 4E	1.566289	3.38846E-06	5.88745E-05
TC12000856.hg.1	ATP2A2	488	ATPase, Ca++ transporting, cardiac muscle, slow twitch 2	1.566111	0.000135983	0.001060246
TC22000092.hg.1	UBE2L3	7332	ubiquitin-conjugating enzyme E2L 3	1.565981	7.59633E-06	0.000110429
TC06001990.hg.1	SCML4	256380	sex comb on midleg-like 4 (Drosophila)	1.565135	0.000621766	0.003634761
TC05000866.hg.1	CYFIP2	26999	cytoplasmic FMR1 interacting protein 2	1.565068	0.000429781	0.002684727
TC12001886.hg.1	CCDC53	51019	coiled-coil domain containing 53	1.564621	1.38361E-07	5.76993E-06
TC17001035.hg.1	SLC25A11	8402	solute carrier family 25 (mitochondrial carrier; oxoglutarate carrier), member 11	1.564462	3.01824E-07	9.84832E-06
TC07000547.hg.1	ANKIB1	54467	ankyrin repeat and IBR domain containing 1	1.564189	0.001327914	0.006724864
TC01001691.hg.1	LAX1	54900	lymphocyte transmembrane adaptor 1	1.563494	0.000173006	0.001287823
TC11000687.hg.1	RAD9A	5883	RAD9 homolog A (S. pombe)	1.5634	9.34568E-07	2.22854E-05
TC01002156.hg.1	DNAJC11	55735	DnaJ (Hsp40) homolog, subfamily C, member 11	1.562952	1.9551E-06	3.89873E-05
TC20000589.hg.1	TMEM230	29058	transmembrane protein 230	1.561555	0.000222284	0.001566709
TC01002383.hg.1	STMN1	3925	stathmin 1	1.561385	3.84448E-09	5.29653E-07
TC14001260.hg.1	ERH	2079	enhancer of rudimentary homolog (Drosophila)	1.560992	0.000812413	0.004525217
TC14000406.hg.1	EIF2S1	1965	eukaryotic translation initiation factor 2, subunit 1 alpha, 35kDa	1.560749	0.000126516	0.001002267
TC01002196.hg.1	EXOSC10	5394	exosome component 10	1.560085	1.66667E-05	0.000200881
TC09001163.hg.1	LOC642236	642236	FSHD region gene 1 pseudogene	1.559613	5.47342E-05	0.000510134

transcript cluster id	Gene Symbol	Entrez Gene ID	Description	FoldChange (AIM/control)	P.Value (AIM/control)	adj.P.Val (AIM/control)
TC03000684.hg.1	ACAD9	28976	acyl-CoA dehydrogenase family, member 9	1.55941	1.65027E-08	1.29339E-06
TC18000413.hg.1	NPC1	4864	Niemann-Pick disease, type C1	1.559107	0.000112524	0.000907741
TC02000363.hg.1	MDH1	4190	malate dehydrogenase 1, NAD (soluble)	1.559049	6.6426E-07	1.72882E-05
TC06000170.hg.1	HIST1H2BE	8344	histone cluster 1, H2be	1.558714	3.37921E-05	0.000348118
TC02001024.hg.1	HAT1	8520	histone acetyltransferase 1	1.558513	3.76576E-05	0.000378717
TC19001095.hg.1	KHSRP	8570	KH-type splicing regulatory protein	1.558513	6.18464E-06	9.47227E-05
TC20000899.hg.1	SLC35C2	51006	solute carrier family 35, member C2	1.558457	1.24376E-06	2.75199E-05
TC12001155.hg.1	LPCAT3	10162	lysophosphatidylcholine acyltransferase 3	1.558318	0.000154589	0.001176002
TC09001654.hg.1	TOR1A	1861	torsin family 1, member A (torsin A)	1.558214	1.66405E-05	0.000200821
TC16000063.hg.1	TRAF7	84231	TNF receptor-associated factor 7, E3 ubiquitin protein ligase	1.5578	1.05197E-07	4.75331E-06
TC01002655.hg.1	TXNDC12	51060	thioredoxin domain containing 12 (endoplasmic reticulum)	1.557661	0.000259218	0.001777277
TC19000747.hg.1	AP2A1	160	adaptor-related protein complex 2, alpha 1 subunit	1.557118	9.06479E-07	2.17226E-05
TC21001058.hg.1	DONSON	29980	downstream neighbor of SON	1.557118	4.08235E-06	6.8392E-05
TC11001251.hg.1	POLR2L	5441	polymerase (RNA) II (DNA directed) polypeptide L, 7.6kDa	1.556834	9.2168E-06	0.00012718
TC0X000661.hg.1	HTATSF1	27336	HIV-1 Tat specific factor 1	1.556748	6.78497E-05	0.000603025
TC07001509.hg.1	RFC2	5982	replication factor C (activator 1) 2, 40kDa	1.556483	2.39155E-07	8.45615E-06
TC12000843.hg.1	UNG	7374	uracil-DNA glycosylase	1.556451	1.14723E-10	7.61842E-08
TC06000366.hg.1	MICB	4277	MHC class I polypeptide-related sequence B	1.556444	6.38263E-05	0.000575532
TC02002536.hg.1	OLA1	29789	Obg-like ATPase 1	1.556021	4.70008E-06	7.62244E-05
TC09001538.hg.1	FBXW2	26190	F-box and WD repeat domain containing 2	1.555209	8.67703E-08	4.14271E-06
TC02002067.hg.1	KRCC1	51315	lysine-rich coiled-coil 1	1.555108	0.003856831	0.01561411
TC01002498.hg.1	PSMB2	5690	proteasome (prosome, macropain) subunit, beta type, 2	1.55485	4.3195E-06	7.14604E-05
TC0X000014.hg.1	CD99	4267	CD99 molecule	1.554837	5.19564E-07	1.45316E-05
TC16001218.hg.1	CHTF8	54921	CTF8, chromosome transmission fidelity factor 8 homolog (S. cerevisiae)	1.554814	4.98929E-07	1.41396E-05
TC11000088.hg.1	STIM1	6786	stromal interaction molecule 1	1.554674	1.73286E-05	0.000207129
TC19000164.hg.1	UBL5	59286	ubiquitin-like 5	1.554411	6.36576E-08	3.32893E-06
TC02000515.hg.1	USP39	10713	ubiquitin specific peptidase 39	1.553593	5.90171E-08	3.16758E-06
TC07000673.hg.1	PSMC2	5701	proteasome (prosome, macropain) 26S subunit, ATPase, 2	1.553528	2.41679E-05	0.000268288
TC19000548.hg.1	TIMM50	92609	translocase of inner mitochondrial membrane 50 homolog (S. cerevisiae)	1.553198	0.000145464	0.001119528
TC01000508.hg.1	ZMPSTE24	10269	zinc metallopeptidase STE24	1.553173	8.26897E-06	0.000117565
TC06000581.hg.1	UBR2	23304	ubiquitin protein ligase E3 component n-recognin 2	1.553171	0.003923686	0.0158306
TC17000981.hg.1	PRPF8	10594	pre-mRNA processing factor 8	1.553128	0.000211985	0.001513777
TC06004096.hg.1	SLC35A1	10559	solute carrier family 35 (CMP-sialic acid transporter), member A1	1.552823	9.23055E-05	0.000771037
TC03001372.hg.1	MAP4	4134	microtubule-associated protein 4	1.552247	3.8718E-06	6.54921E-05
TC17001019.hg.1	ANKFY1	51479	ankyrin repeat and FYVE domain containing 1	1.551374	1.1019E-05	0.000145597
TC03001850.hg.1	GK5	256356	glycerol kinase 5 (putative)	1.551127	0.000154138	0.001175081
TC01002649.hg.1	EPS15	2060	epidermal growth factor receptor pathway substrate 15	1.550573	0.000371204	0.002379318
TC04001634.hg.1	LRBA	987	LPS-responsive vesicle trafficking, beach and anchor containing	1.550348	0.001785277	0.008502867
TC09001609.hg.1	ENG	2022	endoglin	1.550199	2.56796E-06	4.79729E-05
TC07000863.hg.1	MIR4468	100616226	microRNA 4468	1.547311	9.10878E-06	0.000126165
TC08001097.hg.1	KIF13B	23303	kinesin family member 13B	1.546916	7.92177E-06	0.000114057
TC09001490.hg.1	SUSD1	64420	sushi domain containing 1	1.546666	0.000326672	0.00214592
TC17001521.hg.1	DNAJC7	7266	DnaJ (Hsp40) homolog, subfamily C, member 7	1.546577	1.64182E-05	0.000198885
TC13000435.hg.1	CDC16	8881	cell division cycle 16	1.545794	0.000334762	0.002187345
TC02002106.hg.1	SNRNP200	23020	small nuclear ribonucleoprotein 200kDa (U5)	1.54576	9.00932E-06	0.000125369
TC01001381.hg.1	NDUFS2	4720	NADH dehydrogenase (ubiquinone) Fe-S protein 2, 49kDa (NADH-coenzyme Q reductase)	1.545394	5.61486E-09	6.67165E-07
TC13000419.hg.1	CUL4A	8451	cullin 4A	1.545173	9.32517E-07	2.23043E-05
TC0X001009.hg.1	SLC38A5	92745	solute carrier family 38, member 5	1.545114	5.61674E-07	1.52311E-05
TC13000689.hg.1	VPS36	51028	vacuolar protein sorting 36 homolog (S. cerevisiae)	1.5451	2.34838E-05	0.000262971
TC06001948.hg.1	MAP3K7	6885	mitogen-activated protein kinase kinase kinase 7	1.545039	1.5348E-05	0.000189014
TC17001166.hg.1	UBE2S	27338	ubiquitin-conjugating enzyme E2S	1.544704	0.003641456	0.01494945
TC07001206.hg.1	OSBPL3	26031	oxysterol binding protein-like 3	1.544484	7.33559E-06	0.000107769
TC08000726.hg.1	NDUFB9	4715	NADH dehydrogenase (ubiquinone) 1 beta subcomplex, 9, 22kDa	1.543812	2.80772E-05	0.000301904
TC07001126.hg.1	EIF2AK1	27102	eukaryotic translation initiation factor 2-alpha kinase 1	1.543717	2.05805E-07	7.66471E-06
TC22001491.hg.1	MFNG	4242	MFNG O-fucosylpeptide 3-beta-N-acetylglucosaminyltransferase	1.543574	6.44389E-05	0.000580034
TC05001605.hg.1	TTC37	9652	tetratricopeptide repeat domain 37	1.543473	0.004492966	0.01767901
TC08000479.hg.1	TERF1	7013	telomeric repeat binding factor (NIMA-interacting) 1	1.543284	1.39067E-05	0.00017506
TC01003709.hg.1	KLHL12	59349	kelch-like family member 12	1.543157	1.29354E-08	1.10304E-06
TC15000253.hg.1	C15orf41	84529	chromosome 15 open reading frame 41	1.543148	1.91208E-05	0.000223635
TC15000868.hg.1	BLM	641	Bloom syndrome, RecQ helicase-like	1.542633	2.76402E-06	5.06656E-05
TC06000916.hg.1	NT5DC1	221294	5'-nucleotidase domain containing 1	1.54223	0.01037417	0.03440714
TC01000872.hg.1	ABCD3	5825	ATP-binding cassette, sub-family D (ALD), member 3	1.542195	1.69286E-07	6.72616E-06
TC01002479.hg.1	AK2	204	adenylate kinase 2	1.541863	6.28956E-09	7.25633E-07
TC09001064.hg.1	HINT2	84681	histidine triad nucleotide binding protein 2	1.541634	1.28979E-07	5.5059E-06
TC08001183.hg.1	THAP1	55145	THAP domain containing, apoptosis associated protein 1	1.541557	1.43059E-06	3.0888E-05
TC11000239.hg.1	GTF2H1	2965	general transcription factor IIH, polypeptide 1, 62kDa	1.541363	4.86773E-06	7.81442E-05
TC05001033.hg.1	HNRNPAB	3182	heterogeneous nuclear ribonucleoprotein A/B	1.541165	0.002588776	0.01135461
TC16000328.hg.1	MAZ	4150	MYC-associated zinc finger protein (purine-binding transcription factor)	1.541162	2.25386E-07	8.17777E-06
TC01002319.hg.1	HP1BP3	50809	heterochromatin protein 1, binding protein 3	1.540544	6.11911E-05	0.000557183
TC17000833.hg.1	RAB37	326624	RAB37, member RAS oncogene family	1.540475	6.94238E-07	1.79242E-05
TC01001158.hg.1	LOC388692	388692	uncharacterized LOC388692	1.540379	0.003096844	0.01311379
TC17000315.hg.1	NLK	51701	nemo-like kinase	1.540153	0.000179444	0.001327785

transcript cluster id	Gene Symbol	Entrez Gene ID	Description	FoldChange (AIM/control)	P.Value (AIM/control)	adj.P.Val (AIM/control)
TC01002529.hg.1	SF3A3	10946	splicing factor 3a, subunit 3, 60kDa	1.539667	1.5698E-05	0.000192494
TC12001090.hg.1	FBXL14	144699	F-box and leucine-rich repeat protein 14	1.539459	1.78637E-06	3.66321E-05
TC05000275.hg.1	CWC27	10283	CWC27 spliceosome-associated protein homolog (S. cerevisiae)	1.539286	2.7415E-05	0.000296996
TC14002203.hg.1	SLIRP	81892	SRA stem-loop interacting RNA binding protein	1.539224	0.003064083	0.01300833
TC12001632.hg.1	AGAP2	116986	ArfGAP with GTPase domain, ankyrin repeat and PH domain 2	1.539036	1.49861E-06	3.20592E-05
TC12000848.hg.1	UBE3B	89910	ubiquitin protein ligase E3B	1.538799	4.11196E-05	0.000406933
TC19000578.hg.1	CCDC97	90324	coiled-coil domain containing 97	1.537292	0.000100581	0.000828056
TC17000204.hg.1	DRG2	1819	developmentally regulated GTP binding protein 2	1.537205	1.59316E-06	3.36941E-05
TC11000652.hg.1	SF3B2	10992	splicing factor 3b, subunit 2, 145kDa	1.537065	3.25555E-07	1.05586E-05
TC19001135.hg.1	MYO1F	4542	myosin IF	1.53701	0.001729377	0.00828639
TC03003351.hg.1	RBM6	10180	RNA binding motif protein 6	1.536772	0.000233231	0.001634306
TC01003949.hg.1	ABCB10	23456	ATP-binding cassette, sub-family B (MDR/TAP), member 10	1.536667	1.77684E-06	3.64927E-05
TC06001385.hg.1	HIST1H2BJ	8970	histone cluster 1, H2bj	1.536657	5.79782E-06	9.00335E-05
TC12000497.hg.1	RAB5B	5869	RAB5B, member RAS oncogene family	1.536115	1.29847E-05	0.000166151
TC16000215.hg.1	CCP110	9738	centriolar coiled coil protein 110kDa	1.535395	2.3205E-06	4.44127E-05
TC02000580.hg.1	CIAO1	9391	cytosolic iron-sulfur protein assembly 1	1.535361	8.89282E-08	4.24123E-06
TC05000004.hg.1	SDHA	6389	succinate dehydrogenase complex, subunit A, flavoprotein (Fp)	1.534576	6.38982E-08	3.34488E-06
TC06000966.hg.1	CENPW	387103	centromere protein W	1.534546	6.03094E-05	0.000551378
TC03001499.hg.1	PDHB	5162	pyruvate dehydrogenase (lipoamide) beta	1.534454	1.80764E-05	0.000214248
TC03001695.hg.1	POLQ	10721	polymerase (DNA directed), theta	1.534431	2.16764E-06	4.22619E-05
TC0X000385.hg.1	NONO	4841	non-POU domain containing, octamer-binding	1.53431	6.0609E-05	0.000553577
TC12003229.hg.1	RHNO1	83695	RAD9-HUS1-RAD1 interacting nuclear orphan 1	1.534253	9.77026E-06	0.000133079
TC01002195.hg.1	SRM	6723	spermidine synthase	1.534179	0.00117214	0.006083406
TC11000908.hg.1	KIAA1731	85459	KIAA1731	1.533789	2.20282E-05	0.000249728
TC19000319.hg.1	FAM32A	26017	family with sequence similarity 32, member A	1.533543	4.88151E-05	0.000466754
TC13000663.hg.1	KPNA3	3839	karyopherin alpha 3 (importin alpha 4)	1.533539	0.000229985	0.001615356
TC14002327.hg.1	VTI1B	10490	vesicle transport through interaction with t-SNAREs 1B	1.533498	1.13082E-06	2.58884E-05
TC01001177.hg.1	VPS45	11311	vacuolar protein sorting 45 homolog (S. cerevisiae)	1.533399	2.33239E-06	4.45596E-05
TC05000906.hg.1	CCNG1	900	cyclin G1	1.532983	1.71655E-05	0.000205994
TC14001105.hg.1	POLE2	5427	polymerase (DNA directed), epsilon 2, accessory subunit	1.532337	2.09216E-08	1.5273E-06
TC01003650.hg.1	UCHL5	51377	ubiquitin carboxyl-terminal hydrolase L5	1.532245	9.92295E-06	0.000134693
TC17000958.hg.1	TBCD	6904	tubulin folding cofactor D	1.532094	1.09641E-05	0.000145363
TC11002076.hg.1	C2CD3	26005	C2 calcium-dependent domain containing 3	1.53202	1.18494E-07	5.19386E-06
TC12002016.hg.1	WSB2	55884	WD repeat and SOCS box containing 2	1.531551	1.76833E-06	3.64521E-05
TC09000510.hg.1	INVS	27130	inversin	1.531362	0.000912367	0.004977751
TC05001061.hg.1	CNOT6	57472	CCR4-NOT transcription complex, subunit 6	1.531215	9.35966E-07	2.24156E-05
TC11003434.hg.1	REXO2	25996	RNA exonuclease 2	1.530978	0.000109438	0.000890048
TC02001069.hg.1	RBM45	129831	RNA binding motif protein 45	1.530559	4.67589E-05	0.000450229
TC06000886.hg.1	GTF3C6	112495	general transcription factor IIIC, polypeptide 6, alpha 35kDa	1.530488	1.86061E-05	0.000219184
TC11000170.hg.1	IPO7	10527	importin 7	1.529982	0.0128396	0.04086252
TC05002087.hg.1	THOC3	84321	THO complex 3	1.528585	1.1298E-06	2.58884E-05
TC06001282.hg.1	MCUR1	63933	mitochondrial calcium uniporter regulator 1	1.528184	1.58911E-05	0.000194402
TC11003449.hg.1	TMX2	51075	thioredoxin-related transmembrane protein 2	1.528151	0.000356904	0.002308245
TC08001152.hg.1	RNF5P1	286140	ring finger protein 5, E3 ubiquitin protein ligase pseudogene 1	1.528073	4.6993E-06	7.63849E-05
TC17000162.hg.1	COX10	1352	cytochrome c oxidase assembly homolog 10 (yeast)	1.527309	3.95388E-05	0.000395315
TC15001217.hg.1	RASGRP1	10125	RAS guanyl releasing protein 1 (calcium and DAG-regulated)	1.527259	0.005103934	0.01962675
TC08001084.hg.1	CCDC25	55246	coiled-coil domain containing 25	1.526772	1.86759E-06	3.79369E-05
TC05001011.hg.1	NSD1	64324	nuclear receptor binding SET domain protein 1	1.526499	2.6994E-05	0.000293799
TC05000615.hg.1	ISOC1	51015	isochorismatase domain containing 1	1.526208	6.31589E-05	0.000572836
TC06001351.hg.1	HIST1H1D	3007	histone cluster 1, H1d	1.525912	1.81429E-06	3.71445E-05
TC18000285.hg.1	LPIN2	9663	lipin 2	1.525133	3.92783E-06	6.65536E-05
TC02000082.hg.1	TRIB2	28951	tribbles homolog 2 (Drosophila)	1.52462	0.01420009	0.04423776
TC03000256.hg.1	CCR5	1234	chemokine (C-C motif) receptor 5 (gene/pseudogene)	1.52364	2.27491E-06	4.38818E-05
TC11003457.hg.1	ARL2	402	ADP-ribosylation factor-like 2	1.523402	4.32982E-07	1.28084E-05
TC03003336.hg.1	HACL1	26061	2-hydroxyacyl-CoA lyase 1	1.52339	1.84317E-07	7.14569E-06
TC03001374.hg.1	CDC25A	993	cell division cycle 25A	1.523047	7.07815E-07	1.82541E-05
TC14000310.hg.1	STYX	6815	serine/threonine/tyrosine interacting protein	1.522946	0.001345894	0.006807714
TC06000789.hg.1	ORC3	23595	origin recognition complex, subunit 3	1.522719	9.19122E-06	0.000127405
TC04000500.hg.1	SMARCAD1	56916	SWI/SNF-related, matrix-associated actin-dependent regulator of chromatin, subfamily a,	1.522695	7.93511E-05	0.000685465
TC01002450.hg.1	SNRNP40	9410	small nuclear ribonucleoprotein 40kDa (U5)	1.522074	1.02081E-05	0.000137833
TC07002047.hg.1	XRCC2	7516	X-ray repair complementing defective repair in Chinese hamster cells 2	1.52188	2.45325E-08	1.71466E-06
TC14002192.hg.1	DCAF11	80344	DDB1 and CUL4 associated factor 11	1.521467	5.43257E-08	3.00104E-06
TC19001438.hg.1	ZNF792	126375	zinc finger protein 792	1.521311	0.002593539	0.01137283
TC19000665.hg.1	CALM3	808	calmodulin 3 (phosphorylase kinase, delta)	1.520617	1.19932E-07	5.2526E-06
TC15001507.hg.1	RORA	6095	RAR-related orphan receptor A	1.520592	8.12021E-05	0.000698041
TC10001652.hg.1	XPNPEP1	7511	X-prolyl aminopeptidase (aminopeptidase P) 1, soluble	1.52022	3.83168E-08	2.3496E-06
TC06001692.hg.1	GLO1	2739	glyoxalase I	1.519976	0.003304746	0.01384739
TC07003337.hg.1	PVRIG	79037	poliovirus receptor related immunoglobulin domain containing	1.519769	3.65072E-05	0.00037054
TC22001459.hg.1	SNRPD3	6634	small nuclear ribonucleoprotein D3 polypeptide 18kDa	1.519762	0.000143013	0.001106911
TC19000531.hg.1	EIF3K	27335	eukaryotic translation initiation factor 3, subunit K	1.519525	0.000396885	0.002523124
TC19001658.hg.1	AP2S1	1175	adaptor-related protein complex 2, sigma 1 subunit	1.519519	9.5983E-07	2.28659E-05
TC12000993.hg.1	ATP6V0A2	23545	ATPase, H+ transporting, lysosomal V0 subunit a2	1.519433	1.63148E-06	3.43605E-05

transcript cluster id	Gene Symbol	Entrez Gene ID	Description	FoldChange (AIM/control)	P.Value (AIM/control)	adj.P.Val (AIM/control)
TC15000377.hg.1	DUT	1854	deoxyuridine triphosphatase	1.519395	4.81845E-06	7.7932E-05
TC22000791.hg.1	DDX17	10521	DEAD (Asp-Glu-Ala-Asp) box helicase 17	1.519372	0.009889063	0.03319795
TC09000846.hg.1	NELFB	25920	negative elongation factor complex member B	1.519361	2.99587E-07	9.88115E-06
TC17000026.hg.1	RAP1GAP2	23108	RAP1 GTPase activating protein 2	1.519144	0.000241428	0.001684393
TC09001283.hg.1	C9orf64	84267	chromosome 9 open reading frame 64	1.51904	2.23172E-06	4.33146E-05
TC11001750.hg.1	NUP160	23279	nucleoporin 160kDa	1.519002	0.000161672	0.001221412
TC19001720.hg.1	NOSIP	51070	nitric oxide synthase interacting protein	1.5189	0.00203553	0.009410351
TC17001892.hg.1	SRP68	6730	signal recognition particle 68kDa	1.518719	5.50744E-05	0.00051446
TC07003379.hg.1	COPG2	26958	coatomer protein complex, subunit gamma 2	1.51857	3.29678E-05	0.000343614
TC0X000470.hg.1	CSTF2	1478	cleavage stimulation factor, 3' pre-RNA, subunit 2, 64kDa	1.517928	2.00461E-09	3.71801E-07
TC17000369.hg.1	SUZ12	23512	SUZ12 polycomb repressive complex 2 subunit	1.517902	5.71377E-06	8.92785E-05
TC17000469.hg.1	LASP1	3927	LIM and SH3 protein 1	1.517675	1.08157E-05	0.000144079
TC02000527.hg.1	ANAPC1P1	100286979	anaphase promoting complex subunit 1 pseudogene 1	1.517621	4.80792E-06	7.78441E-05
TC11000970.hg.1	AASDHPPT	60496	aminoadipate-semialdehyde dehydrogenase-phosphopantetheinyl transferase	1.517255	1.44859E-06	3.13277E-05
TC11002284.hg.1	PPP2R1B	5519	protein phosphatase 2, regulatory subunit A, beta	1.517009	6.25579E-05	0.000568265
TC0X001266.hg.1	PSMD10	5716	proteasome (prosome, macropain) 26S subunit, non-ATPase, 10	1.516988	5.45009E-05	0.000510134
TC21000212.hg.1	RRP1B	23076	ribosomal RNA processing 1 homolog B (S. cerevisiae)	1.516978	0.001968936	0.009172586
TC16000994.hg.1	TUFM	7284	Tu translation elongation factor, mitochondrial	1.51658	0.000189013	0.001387016
TC04000454.hg.1	ENOPH1	58478	enolase-phosphatase 1	1.515801	1.52871E-05	0.000189014
TC22001463.hg.1	SH3BP1	23616	SH3-domain binding protein 1	1.515561	5.90368E-08	3.19516E-06
TC02000514.hg.1	RNF181	51255	ring finger protein 181	1.514871	1.58792E-05	0.000194535
TC07000930.hg.1	GSTK1	373156	glutathione S-transferase kappa 1	1.514871	0.001340597	0.006790019
TC15001271.hg.1	UBR1	197131	ubiquitin protein ligase E3 component n-recognin 1	1.51477	0.0004064	0.002574496
TC07000646.hg.1	ZNHIT1	10467	zinc finger, HIT-type containing 1	1.514595	3.61035E-09	5.24455E-07
TC06000343.hg.1	MRPS18B	28973	mitochondrial ribosomal protein S18B	1.514439	4.32572E-06	7.1931E-05
TC03001538.hg.1	UBA3	9039	ubiquitin-like modifier activating enzyme 3	1.514411	6.60614E-07	1.73561E-05
TC11001461.hg.1	SAAL1	113174	serum amyloid A-like 1	1.514245	6.26909E-06	9.6196E-05
TC11003486.hg.1	C11orf48	79081	chromosome 11 open reading frame 48	1.514217	1.18027E-08	1.06912E-06
TC04001807.hg.1	CASP3	836	caspase 3, apoptosis-related cysteine peptidase	1.513932	9.16545E-05	0.000768943
TC15001788.hg.1	SEC11A	23478	SEC11 homolog A (S. cerevisiae)	1.51326	8.11793E-05	0.000698104
TC19000698.hg.1	LOC100505812	100505812	uncharacterized LOC100505812	1.51282	6.93649E-06	0.000103593
TC11001050.hg.1	RNF214	257160	ring finger protein 214	1.512502	3.73677E-05	0.000377946
TC05000886.hg.1	UBLCP1	134510	ubiquitin-like domain containing CTD phosphatase 1	1.512432	0.000199757	0.001447951
TC19001041.hg.1	AES	166	amino-terminal enhancer of split	1.512423	6.03202E-05	0.000552537
TC05001871.hg.1	HDAC3	8841	histone deacetylase 3	1.512409	4.61008E-07	1.34536E-05
TC0X001141.hg.1	HDAC8	55869	histone deacetylase 8	1.51228	8.6581E-10	2.12817E-07
TC13000111.hg.1	BRCA2	675	breast cancer 2, early onset	1.512143	0.000631007	0.003686578
TC20000818.hg.1	DSN1	79980	DSN1, MIND kinetochore complex component, homolog (S. cerevisiae)	1.51203	1.98981E-05	0.000231202
TC18000046.hg.1	RALBP1	10928	ralA binding protein 1	1.511998	0.001293622	0.006590464
TC02000546.hg.1	RPIA	22934	ribose 5-phosphate isomerase A	1.511756	0.000244648	0.001701187
TC0X001164.hg.1	ABCB7	22	ATP-binding cassette, sub-family B (MDR/TAP), member 7	1.511419	0.000939148	0.005089598
TC16001357.hg.1	FANCA	2175	Fanconi anemia, complementation group A	1.511389	4.45782E-08	2.63763E-06
TC12003221.hg.1	ANAPC7	51434	anaphase promoting complex subunit 7	1.511303	1.12807E-07	5.06175E-06
TC12002009.hg.1	C12orf49	79794	chromosome 12 open reading frame 49	1.510921	1.21071E-05	0.000157761
TC12000144.hg.1	LOC642846	642846	DEAD/H (Asp-Glu-Ala-Asp/His) box polypeptide 11-like	1.510417	7.64101E-05	0.000665411
TC16001278.hg.1	CFDP1	10428	craniofacial development protein 1	1.50992	2.08505E-05	0.000240555
TC13000341.hg.1	RAP2A	5911	RAP2A, member of RAS oncogene family	1.509681	4.99987E-07	1.42645E-05
TC13000226.hg.1	SUGT1	10910	SGT1, suppressor of G2 allele of SKP1 (S. cerevisiae)	1.509487	0.000467305	0.002881799
TC10000002.hg.1	ZMYND11	10771	zinc finger, MYND-type containing 11	1.509056	0.000638402	0.003722583
TC0X000618.hg.1	SASH3	54440	SAM and SH3 domain containing 3	1.509032	0.001051377	0.005583451
TC05000010.hg.1	TRIP13	9319	thyroid hormone receptor interactor 13	1.508694	3.1395E-09	4.90755E-07
TC12001094.hg.1	DCP1B	196513	decapping mRNA 1B	1.508689	3.30084E-07	1.06488E-05
TC20000968.hg.1	AURKA	6790	aurora kinase A	1.508507	8.49812E-08	4.13761E-06
TC07001346.hg.1	H2AFV	94239	H2A histone family, member V	1.508096	6.41723E-05	0.000580034
TC16000482.hg.1	NLRC5	84166	NLR family, CARD domain containing 5	1.508053	0.000281765	0.001905044
TC20001725.hg.1	SNX5	27131	sorting nexin 5	1.507969	4.02189E-05	0.000401163
TC07003350.hg.1	KDELR2	11014	KDEL (Lys-Asp-Glu-Leu) endoplasmic reticulum protein retention receptor 2	1.507624	4.96118E-05	0.00047481
TC16001178.hg.1	FAM96B	51647	family with sequence similarity 96, member B	1.50745	1.4885E-07	6.16612E-06
TC03001488.hg.1	ARHGEF3	50650	Rho guanine nucleotide exchange factor (GEF) 3	1.507213	7.84914E-06	0.0001139
TC18000166.hg.1	HAUS1	115106	HAUS augmin-like complex, subunit 1	1.506773	1.04612E-08	9.92906E-07
TC04000512.hg.1	METAP1	23173	methionyl aminopeptidase 1	1.50676	1.80175E-05	0.000214296
TC0X000092.hg.1	PDHA1	5160	pyruvate dehydrogenase (lipoamide) alpha 1	1.505243	1.33938E-06	2.94722E-05
TC19002700.hg.1	SARS2	54938	seryl-tRNA synthetase 2, mitochondrial	1.505094	1.56477E-08	1.26817E-06
TC12003266.hg.1	PXMP2	5827	peroxisomal membrane protein 2, 22kDa	1.505009	4.37888E-09	5.80124E-07
TC0X001370.hg.1	MBNL3	55796	muscleblind-like splicing regulator 3	1.504332	0.001838047	0.00870212
TC01001348.hg.1	IFI16	3428	interferon, gamma-inducible protein 16	1.503924	0.001559474	0.007639711
TC12000595.hg.1	CAND1	55832	cullin-associated and neddylation-dissociated 1	1.503333	6.37299E-05	0.0005773
TC05000019.hg.1	NDUFS6	4726	NADH dehydrogenase (ubiquinone) Fe-S protein 6, 13kDa (NADH-coenzyme Q reductase)	1.502512	9.10427E-08	4.34114E-06
TC01002327.hg.1	USP48	84196	ubiquitin specific peptidase 48	1.502273	0.000175858	0.001310004
TC05001331.hg.1	EMB	133418	embigin	1.501971	0.003794366	0.0154428
TC08000332.hg.1	SGK196	84197	protein kinase-like protein SgK196	1.501291	2.80226E-05	0.000302541
TC16001208.hg.1	SLC7A6OS	84138	solute carrier family 7, member 6 opposite strand	1.50038	7.61636E-05	0.000664946

transcript cluster id	Gene Symbol	Entrez Gene ID	Description	FoldChange (AIM/control)	P.Value (AIM/control)	adj.P.Val (AIM/control)
TC0X000353.hg.1	MSN	4478	moesin	1.500032	6.77469E-05	0.000605077
TC12001966.hg.1	PPTC7	160760	PTC7 protein phosphatase homolog (S. cerevisiae)	-1.500297	0.000195753	0.00142998
TC19001901.hg.1	ZNF154	7710	zinc finger protein 154	-1.501258	0.00107491	0.005689988
TC16000312.hg.1	CD19	930	CD19 molecule	-1.50131	0.000549444	0.003300654
TC12000393.hg.1	DIP2B	57609	DIP2 disco-interacting protein 2 homolog B (Drosophila)	-1.501384	7.24943E-05	0.000638566
TC22001425.hg.1	APOBEC3B	9582	apolipoprotein B mRNA editing enzyme, catalytic polypeptide-like 3B	-1.50154	0.01037821	0.03443971
TC15001582.hg.1	SNORD18A	595098	small nucleolar RNA, C/D box 18A	-1.5022	0.008355384	0.02903858
TC06001529.hg.1	SNORD117	692233	small nucleolar RNA, C/D box 117	-1.502644	0.004996951	0.01927538
TC17002907.hg.1	JUP	3728	junction plakoglobin	-1.502891	1.59969E-07	6.50297E-06
TC11001143.hg.1	NRGN	4900	neurogranin (protein kinase C substrate, RC3)	-1.504603	0.01618366	0.0491623
TC01001505.hg.1	SUCO	51430	SUN domain containing ossification factor	-1.505896	0.004094118	0.01639564
TC03001911.hg.1	MBNL1-AS1	401093	MBNL1 antisense RNA 1	-1.505923	1.78447E-06	3.68413E-05
TC01001748.hg.1	CAMK1G	57172	calcium/calmodulin-dependent protein kinase IG	-1.505937	1.76943E-07	6.96592E-06
TC12001842.hg.1	HAL	3034	histidine ammonia-lyase	-1.505965	1.14637E-05	0.000150707
TC06001997.hg.1	SESN1	27244	sestrin 1	-1.50636	8.70372E-05	0.000735837
TC15000824.hg.1	PDE8A	5151	phosphodiesterase 8A	-1.506771	1.12121E-06	2.58858E-05
TC10001037.hg.1	USP6NL	9712	USP6 N-terminal like	-1.506809	0.000408748	0.002589737
TC08000946.hg.1	PPP1R3B	79660	protein phosphatase 1, regulatory subunit 3B	-1.507382	0.000325269	0.002143718
TC14000303.hg.1	GNG2	54331	guanine nucleotide binding protein (G protein), gamma 2	-1.507807	0.000799919	0.004472591
TC01002271.hg.1	NBPF1	55672	neuroblastoma breakpoint family, member 1	-1.50804	7.50069E-06	0.00011036
TC20000429.hg.1	CASS4	57091	Cas scaffolding protein family member 4	-1.508689	0.002754766	0.0119497
TC05000835.hg.1	SMIM3	85027	small integral membrane protein 3	-1.509421	4.31722E-05	0.000424449
TC08000873.hg.1	LOC101060626	101060626	F-box only protein 25-like	-1.510005	0.001624605	0.007887446
TC22000165.hg.1	ADRBK2	157	adrenergic, beta, receptor kinase 2	-1.510377	0.01301575	0.04131577
TC15000048.hg.1	SNORD116-1	100033413	small nucleolar RNA, C/D box 116-1	-1.510759	0.009832632	0.03303303
TC12001456.hg.1	CCNT1	904	cyclin T1	-1.511358	0.009394482	0.0318486
TC05001986.hg.1	ADAM19	8728	ADAM metallopeptidase domain 19	-1.512381	3.59362E-05	0.000366315
TC01006302.hg.1	LOC101059961	101059961	neuroblastoma breakpoint family member 21-like	-1.512559	3.39992E-05	0.000350956
TC19001340.hg.1	ZNF506	440515	zinc finger protein 506	-1.513061	0.000260675	0.001790785
TC11000775.hg.1	RELT	84957	RELT tumor necrosis factor receptor	-1.513078	0.000248565	0.001722792
TC14000323.hg.1	LGALS3	3958	lectin, galactoside-binding, soluble, 3	-1.51316	2.63361E-06	4.9444E-05
TC19000099.hg.1	KDM4B	23030	lysine (K)-specific demethylase 4B	-1.514021	4.6469E-05	0.0004487
TC03001270.hg.1	CMTM6	54918	CKLF-like MARVEL transmembrane domain containing 6	-1.514325	0.001096179	0.005775989
TC01001733.hg.1	C4BPA	722	complement component 4 binding protein, alpha	-1.514659	0.01504498	0.0463998
TC19001367.hg.1	ZNF91	7644	zinc finger protein 91	-1.514915	0.003015306	0.01284169
TC04001373.hg.1	PPM1K	152926	protein phosphatase, Mg2+/Mn2+ dependent, 1K	-1.515006	3.2231E-06	5.70021E-05
TC15001348.hg.1	HDC	3067	histidine decarboxylase	-1.515053	2.37851E-07	8.4947E-06
TC02001885.hg.1	BCL11A	53335	B-cell CLL/lymphoma 11A (zinc finger protein)	-1.515806	6.54998E-05	0.000588733
TC07001101.hg.1	GNA12	2768	guanine nucleotide binding protein (G protein) alpha 12	-1.51678	1.24193E-06	2.76886E-05
TC15000356.hg.1	SPATA5L1	79029	spermatogenesis associated 5-like 1	-1.516813	0.001438473	0.007183472
TC10000967.hg.1	IDI1	3422	isopentenyl-diphosphate delta isomerase 1	-1.517311	0.005576103	0.02109188
TC02002538.hg.1	CIR1	9541	corepressor interacting with RBPJ, 1	-1.517779	0.008534459	0.02953688
TC15001690.hg.1	TSPAN3	10099	tetraspanin 3	-1.518666	0.000554826	0.003319889
TC11001939.hg.1	RELA	5970	v-rel reticuloendotheliosis viral oncogene homolog A (avian)	-1.518675	0.007977081	0.02800463
TC18000133.hg.1	RNF138	51444	ring finger protein 138, E3 ubiquitin protein ligase	-1.518959	0.01107339	0.03629089
TC04001609.hg.1	OTUD4	54726	OTU domain containing 4	-1.519145	0.005738185	0.02157544
TC05000525.hg.1	EPB41L4A-AS1	114915	EPB41L4A antisense RNA 1	-1.519423	0.000376915	0.002414241
TC19000260.hg.1	CLEC17A	388512	C-type lectin domain family 17, member A	-1.519897	0.00018398	0.001357468
TC07000650.hg.1	CUX1	1523	cut-like homeobox 1	-1.520455	3.61431E-06	6.25031E-05
TC11000770.hg.1	ATG16L2	89849	autophagy related 16-like 2 (S. cerevisiae)	-1.520506	1.12368E-05	0.000148336
TC08001658.hg.1	SLA	6503	Src-like-adaptor	-1.521415	0.004012253	0.01613188
TC11001193.hg.1	LINC00167	440072	long intergenic non-protein coding RNA 167	-1.521745	7.35063E-06	0.000108249
TC0X000986.hg.1	MIR222	407007	microRNA 222	-1.521829	0.01276019	0.0406614
TC07001934.hg.1	CLEC5A	23601	C-type lectin domain family 5, member A	-1.52365	0.003300899	0.01383479
TC01000023.hg.1	ISG15	9636	ISG15 ubiquitin-like modifier	-1.524041	0.009505556	0.03216052
TC08000590.hg.1	SDC2	6383	syndecan 2	-1.524286	1.01219E-05	0.000136802
TC14000306.hg.1	PTGER2	5732	prostaglandin E receptor 2 (subtype EP2), 53kDa	-1.524346	0.001512981	0.007469763
TC05000262.hg.1	ZSWIM6	57688	zinc finger, SWIM-type containing 6	-1.524687	0.006368709	0.02346946
TC09001222.hg.1	MIR548H3	100302287	microRNA 548h-3	-1.526554	0.01596255	0.0485899
TC03000342.hg.1	SNORD69	692109	small nucleolar RNA, C/D box 69	-1.527847	0.003104295	0.01314703
TC19001779.hg.1	SIGLEC10	89790	sialic acid binding Ig-like lectin 10	-1.527919	0.00048728	0.002979552
TC16000222.hg.1	ACSM3	6296	acyl-CoA synthetase medium-chain family member 3	-1.528189	0.000158533	0.001203269
TC15000067.hg.1	SNORD116-24	100033435	small nucleolar RNA, C/D box 116-24	-1.529019	0.009787839	0.03290949
TC19000566.hg.1	ITPKC	80271	inositol-trisphosphate 3-kinase C	-1.52933	8.92068E-05	0.000750729
TC18000018.hg.1	TGIF1	7050	TGFB-induced factor homeobox 1	-1.529535	4.15709E-08	2.49155E-06
TC19000673.hg.1	MIR3190	100422899	microRNA 3190	-1.529624	0.004091538	0.0163807
TC08000487.hg.1	LY96	23643	lymphocyte antigen 96	-1.53003	0.003610927	0.01485863
TC01001857.hg.1	H3F3A	3020	H3 histone, family 3A	-1.530847	0.000490507	0.002995136
TC03000637.hg.1	LOC100129550	100129550	uncharacterized LOC100129550	-1.531096	0.003753809	0.01530789
TC17001923.hg.1	LOC100653515	100653515	differential display clone 8	-1.53128	0.001638463	0.007938049
TC11001582.hg.1	TP53I11	9537	tumor protein p53 inducible protein 11	-1.531342	2.14657E-06	4.19869E-05
TC19001261.hg.1	CYP4F2	8529	cytochrome P450, family 4, subfamily F, polypeptide 2	-1.532209	1.44668E-05	0.000181028

transcript cluster id	Gene Symbol	Entrez Gene ID	Description	FoldChange (AIM/control)	P.Value (AIM/control)	adj.P.Val (AIM/control)
TC22000856.hg.1	ARFGAP3	26286	ADP-ribosylation factor GTPase activating protein 3	-1.532677	2.98491E-06	5.35865E-05
TC16001058.hg.1	YBX3P1	440359	Y box binding protein 3 pseudogene 1	-1.533233	2.71787E-06	5.03316E-05
TC12001361.hg.1	FAM60A	58516	family with sequence similarity 60, member A	-1.533296	0.001692015	0.008154493
TC02002693.hg.1	INO80D	54891	INO80 complex subunit D	-1.534236	0.000316269	0.002093384
TC01001559.hg.1	QSOX1	5768	quiescin Q6 sulfhydryl oxidase 1	-1.534367	0.000217425	0.001543994
TC0X000230.hg.1	CDK16	5127	cyclin-dependent kinase 16	-1.534503	0.000706318	0.004046724
TC02001956.hg.1	LOC100133985	100133985	uncharacterized LOC100133985	-1.534881	2.82945E-05	0.000303667
TC19000187.hg.1	C19orf38	255809	chromosome 19 open reading frame 38	-1.535546	0.009493828	0.03212
TC04000224.hg.1	KLHL5	51088	kelch-like family member 5	-1.535558	0.000332466	0.002177162
TC06001508.hg.1	FLOT1	10211	flotillin 1	-1.537195	0.000205676	0.001480521
TC06002020.hg.1	TRAF3IP2	10758	TRAF3 interacting protein 2	-1.537497	1.01346E-06	2.38127E-05
TC19001347.hg.1	ZNF737	100129842	zinc finger protein 737	-1.538383	5.25407E-05	0.000495248
TC01003711.hg.1	ADIPOR1	51094	adiponectin receptor 1	-1.538399	0.00456024	0.01791227
TC01002441.hg.1	LAPTM5	7805	lysosomal protein transmembrane 5	-1.539037	0.000428402	0.002682178
TC05000659.hg.1	UBE2B	7320	ubiquitin-conjugating enzyme E2B	-1.53932	0.004154945	0.01657393
TC05000957.hg.1	SNORA74B	677841	small nucleolar RNA, H/ACA box 74B	-1.539797	0.004859766	0.01884517
TC09001079.hg.1	ZBTB5	9925	zinc finger and BTB domain containing 5	-1.540496	1.79682E-08	1.38123E-06
TC19000049.hg.1	DOT1L	84444	DOT1-like histone H3K79 methyltransferase	-1.540583	0.000556929	0.003326461
TC09001201.hg.1	KLF9	687	Kruppel-like factor 9	-1.540891	0.000916085	0.004991832
TC17001807.hg.1	AMZ2P1	201283	archaelysin family metallopeptidase 2 pseudogene 1	-1.54145	5.23079E-05	0.00049436
TC06000526.hg.1	PNPLA1	285848	patatin-like phospholipase domain containing 1	-1.541634	4.22432E-07	1.26248E-05
TC17000974.hg.1	MYO1C	4641	myosin IC	-1.542216	0.003620801	0.01488237
TC19000177.hg.1	PDE4A	5141	phosphodiesterase 4A, cAMP-specific	-1.542229	1.20063E-06	2.68701E-05
TC15001630.hg.1	ADPGK	83440	ADP-dependent glucokinase	-1.54259	1.70459E-05	0.000204643
TC01000567.hg.1	BTBD19	149478	BTB (POZ) domain containing 19	-1.542811	8.57641E-09	8.59632E-07
TC01002886.hg.1	F3	2152	coagulation factor III (thromboplastin, tissue factor)	-1.544493	5.30743E-07	1.47445E-05
TC0X000998.hg.1	CFP	5199	complement factor properdin	-1.545305	0.000521517	0.003150021
TC16001252.hg.1	AP1G1	164	adaptor-related protein complex 1, gamma 1 subunit	-1.546228	0.001128514	0.005891325
TC01002828.hg.1	BCL10	8915	B-cell CLL/lymphoma 10	-1.546793	0.001388061	0.006973706
TC01006368.hg.1	NBPF14	25832	neuroblastoma breakpoint family, member 14	-1.547436	3.57745E-05	0.000364027
TC0X001412.hg.1	MMGT1	93380	membrane magnesium transporter 1	-1.548377	0.00014633	0.001124547
TC10001563.hg.1	RRP12	23223	ribosomal RNA processing 12 homolog (S. cerevisiae)	-1.549225	0.003406148	0.01417061
TC19001284.hg.1	KIAA1683	80726	KIAA1683	-1.54927	8.71154E-07	2.1175E-05
TC10001718.hg.1	IKZF5	64376	IKAROS family zinc finger 5 (Pegasus)	-1.549527	0.001770871	0.008446687
TC10000189.hg.1	MASTL	84930	microtubule associated serine/threonine kinase-like	-1.550357	0.00796765	0.0279599
TC21000340.hg.1	APP	351	amyloid beta (A4) precursor protein	-1.550987	0.000139515	0.001083221
TC03001109.hg.1	FYTTD1	84248	forty-two-three domain containing 1	-1.55119	2.52308E-07	8.80159E-06
TC01004062.hg.1	AHCTF1	25909	AT hook containing transcription factor 1	-1.552292	0.00042064	0.002640689
TC22000588.hg.1	ADORA2A-AS1	646023	ADORA2A antisense RNA 1	-1.55243	1.96778E-07	7.45609E-06
TC11001106.hg.1	SC5D	6309	sterol-C5-desaturase	-1.552493	2.62713E-05	0.000286687
TC13000322.hg.1	MIR20A	406982	microRNA 20a	-1.553191	0.009369313	0.03179201
TC04000542.hg.1	TET2	54790	tet methylcytosine dioxygenase 2	-1.553433	8.47007E-05	0.000719148
TC0X001242.hg.1	MORF4L2	9643	mortality factor 4 like 2	-1.554478	0.001718999	0.008256254
TC11001406.hg.1	LOC440028	440028	uncharacterized LOC440028	-1.554562	0.002032029	0.009387151
TC08001042.hg.1	BIN3	55909	bridging integrator 3	-1.554882	0.001838122	0.00869377
TC20000715.hg.1	CST3	1471	cystatin C	-1.555071	0.00307152	0.01302868
TC01006309.hg.1	NBPF10	100132406	neuroblastoma breakpoint family, member 10	-1.555532	6.67374E-05	0.000595235
TC19001071.hg.1	TICAM1	148022	toll-like receptor adaptor molecule 1	-1.555658	9.099E-06	0.000125974
TC19001844.hg.1	LAIR1	3903	leukocyte-associated immunoglobulin-like receptor 1	-1.555827	3.62434E-05	0.000367419
TC19002689.hg.1	MED26	9441	mediator complex subunit 26	-1.556181	0.000264424	0.001806424
TC03000016.hg.1	ARL8B	55207	ADP-ribosylation factor-like 8B	-1.556543	6.904E-05	0.000611988
TC04002924.hg.1	SLC39A8	64116	solute carrier family 39 (zinc transporter), member 8	-1.55671	0.000304902	0.00202935
TC10000909.hg.1	ZRANB1	54764	zinc finger, RAN-binding domain containing 1	-1.556881	0.00013223	0.001036834
TC01001727.hg.1	MAPKAPK2	9261	mitogen-activated protein kinase-activated protein kinase 2	-1.557071	0.000368558	0.002367594
TC03000824.hg.1	MIR548H2	100313773	microRNA 548h-2	-1.55721	0.009188143	0.03131944
TC03001207.hg.1	SH3BP5	9467	SH3-domain binding protein 5 (BTK-associated)	-1.557881	1.69778E-06	3.51377E-05
TC03000214.hg.1	CTNNB1	1499	catenin (cadherin-associated protein), beta 1, 88kDa	-1.558423	2.01184E-05	0.000232205
TC20000980.hg.1	PMEPA1	56937	prostate transmembrane protein, androgen induced 1	-1.558927	9.57761E-07	2.26736E-05
TC14000220.hg.1	FAM177A1	283635	family with sequence similarity 177, member A1	-1.558956	4.22932E-07	1.25983E-05
TC01000883.hg.1	PTBP2	58155	polypyrimidine tract binding protein 2	-1.559624	0.001480057	0.007325239
TC16000871.hg.1	TXNDC11	51061	thioredoxin domain containing 11	-1.559941	0.003257062	0.01365634
TC01002074.hg.1	LINC00115	79854	long intergenic non-protein coding RNA 115	-1.560322	0.000109747	0.000890092
TC11003433.hg.1	RBM7	10179	RNA binding motif protein 7	-1.560884	7.06857E-05	0.00062301
TC17000872.hg.1	SPHK1	8877	sphingosine kinase 1	-1.560915	8.87063E-06	0.000123869
TC17001886.hg.1	WBP2	23558	WW domain binding protein 2	-1.561946	0.000618793	0.003621239
TC05003426.hg.1	RAPGEF6	51735	Rap guanine nucleotide exchange factor (GEF) 6	-1.562455	0.003268186	0.01369877
TC14002345.hg.1	LOC100506499	100506499	uncharacterized LOC100506499	-1.562531	5.57862E-07	1.51482E-05
TC01002211.hg.1	DHRS3	9249	dehydrogenase/reductase (SDR family) member 3	-1.563676	0.000511789	0.003095575
TC14000887.hg.1	TMEM55B	90809	transmembrane protein 55B	-1.565315	0.000396762	0.002517938
TC10002942.hg.1	FAS	355	Fas cell surface death receptor	-1.565824	7.10957E-05	0.000625742
TC04000242.hg.1	RHOH	399	ras homolog family member H	-1.567898	0.000421396	0.002642364
TC01006367.hg.1	NBPF11	200030	neuroblastoma breakpoint family, member 24 -RefSeq NM_001101663 -Entrez_id 728912	-1.568469	4.2821E-06	7.1004E-05

transcript cluster id	Gene Symbol	Entrez Gene ID	Description	FoldChange (AIM/control)	P.Value (AIM/control)	adj.P.Val (AIM/control)
TC19001791.hg.1	ZNF350	59348	zinc finger protein 350	-1.569342	0.000529319	0.003187413
TC14001336.hg.1	IRF2BPL	64207	interferon regulatory factor 2 binding protein-like	-1.569821	4.44438E-05	0.000431568
TC11001936.hg.1	SSSCA1-AS1	254100	SSSCA1 antisense RNA 1 (head to head)	-1.57001	0.001541944	0.007557043
TC09001073.hg.1	MIR4540	100616278	microRNA 4540	-1.5702	0.000866071	0.004772507
TC14000781.hg.1	TECPR2	9895	tectonin beta-propeller repeat containing 2	-1.570338	0.001700847	0.008184439
TC11000066.hg.1	KCNQ1	3784	potassium voltage-gated channel, KQT-like subfamily, member 1	-1.570897	2.89387E-06	5.21465E-05
TC17000973.hg.1	CRK	1398	v-crk sarcoma virus CT10 oncogene homolog (avian)	-1.5724	0.001730271	0.008286346
TC21000284.hg.1	HSPA13	6782	heat shock protein 70kDa family, member 13	-1.573081	0.00186124	0.008773683
TC07001452.hg.1	ERV3-1	2086	endogenous retrovirus group 3, member 1	-1.573585	1.08476E-05	0.000143613
TC01000898.hg.1	HIAT1	64645	hippocampus abundant transcript 1	-1.573781	0.000607658	0.003570297
TC22000845.hg.1	NFAM1	150372	NFAT activating protein with ITAM motif 1	-1.573799	0.001360144	0.006851409
TC01001740.hg.1	CR1L	1379	complement component (3b/4b) receptor 1-like	-1.573832	6.37138E-05	0.000574152
TC01002763.hg.1	WLS	79971	wntless homolog (Drosophila)	-1.574509	0.000032534	0.000338647
TC01001724.hg.1	RASSF5	83593	Ras association (RalGDS/AF-6) domain family member 5	-1.574561	0.000173348	0.001288565
TC17000764.hg.1	TANC2	26115	tetratricopeptide repeat, ankyrin repeat and coiled-coil containing 2	-1.574647	6.38062E-07	1.67216E-05
TC04001259.hg.1	YTHDC1	91746	YTH domain containing 1	-1.576558	0.000269341	0.00183002
TC05001294.hg.1	RICTOR	253260	RPTOR independent companion of MTOR, complex 2	-1.580178	0.01113287	0.03639926
TC20000900.hg.1	ELMO2	63916	engulfment and cell motility 2	-1.580314	0.001742621	0.008328096
TC08001099.hg.1	DUSP4	1846	dual specificity phosphatase 4	-1.580528	0.000620944	0.003628625
TC03003367.hg.1	LRRC33	375387	leucine rich repeat containing 33	-1.580882	5.1381E-07	1.43221E-05
TC03000111.hg.1	RAB5A	5868	RAB5A, member RAS oncogene family	-1.581463	0.000501112	0.003040212
TC01001144.hg.1	NBPF15	284565	neuroblastoma breakpoint family, member 16 -RefSeq NM_001102663 -Entrez_id 72893(	-1.581602	1.32301E-05	0.000167633
TC01000322.hg.1	TMEM57	55219	transmembrane protein 57	-1.582536	0.005605935	0.02115656
TC19001586.hg.1	LYPD3	27076	LY6/PLAUR domain containing 3	-1.583077	2.35756E-05	0.000262971
TC08001602.hg.1	MTSS1	9788	metastasis suppressor 1	-1.583904	0.000782008	0.004375729
TC18000059.hg.1	CHMP1B	57132	charged multivesicular body protein 1B	-1.584457	0.000611242	0.003584643
TC14001462.hg.1	DDX24	57062	DEAD (Asp-Glu-Ala-Asp) box polypeptide 24	-1.584843	0.000847254	0.004684545
TC07000307.hg.1	UPP1	7378	uridine phosphorylase 1	-1.585008	0.000050089	0.000475153
TC17001124.hg.1	PIK3R5	23533	phosphoinositide-3-kinase, regulatory subunit 5	-1.585308	0.003673087	0.0150386
TC20000526.hg.1	TPD52L2	7165	tumor protein D52-like 2	-1.585918	0.008330707	0.02892262
TC09001653.hg.1	PTGES	9536	prostaglandin E synthase	-1.586834	1.33195E-05	0.000168465
TC06001273.hg.1	NEDD9	4739	neural precursor cell expressed, developmentally down-regulated 9	-1.587038	0.000472126	0.00289463
TC09000760.hg.1	GTF3C4	9329	general transcription factor IIIC, polypeptide 4, 90kDa	-1.587524	0.00095094	0.005129731
TC16000443.hg.1	CYLD	1540	cylindromatosis (turban tumor syndrome)	-1.587548	4.24752E-06	7.04836E-05
TC08001210.hg.1	RB1CC1	9821	RB1-inducible coiled-coil 1	-1.58803	0.00062283	0.003636441
TC01002701.hg.1	PPAP2B	8613	phosphatidic acid phosphatase type 2B	-1.588395	3.25618E-07	1.04513E-05
TC02000910.hg.1	ZEB2-AS1	100303491	ZEB2 antisense RNA 1	-1.588878	1.69215E-07	6.65905E-06
TC19000214.hg.1	ZNF844	284391	zinc finger protein 844	-1.589291	0.001056045	0.00558772
TC04002929.hg.1	BST1	683	bone marrow stromal cell antigen 1	-1.589427	0.01356956	0.04267382
TC02001206.hg.1	NRP2	8828	neuropilin 2	-1.590557	5.52524E-08	2.98345E-06
TC17001984.hg.1	WDR45B	56270	WD repeat domain 45B	-1.591207	0.01247188	0.03989925
TC16000167.hg.1	SNX29	92017	sorting nexin 29	-1.591376	0.000152557	0.001162302
TC19000649.hg.1	VASP	7408	vasodilator-stimulated phosphoprotein	-1.592191	0.004447533	0.01751725
TC06001488.hg.1	IFITM4P	340198	interferon induced transmembrane protein 4 pseudogene	-1.592487	0.004723478	0.01840811
TC08000546.hg.1	OSGIN2	734	oxidative stress induced growth inhibitor family member 2	-1.594138	0.006815993	0.02469797
TC13000524.hg.1	UBL3	5412	ubiquitin-like 3	-1.594374	0.000294329	0.001965986
TC17001730.hg.1	MIR4736	100616220	microRNA 4736	-1.596241	0.003719269	0.01518383
TC02002145.hg.1	SNORD89	692205	small nucleolar RNA, C/D box 89	-1.596287	0.009542487	0.03223276
TC04000181.hg.1	RBPJ	3516	recombination signal binding protein for immunoglobulin kappa J region	-1.597464	0.002027569	0.009359722
TC16000597.hg.1	ATXN1L	342371	ataxin 1-like	-1.597986	5.27132E-05	0.000495195
TC03000197.hg.1	WDR48	57599	WD repeat domain 48	-1.600545	0.000183622	0.001348615
TC17000141.hg.1	NDEL1	81565	nudE nuclear distribution E homolog (A. nidulans)-like 1	-1.601733	5.62246E-05	0.000520057
TC05001142.hg.1	MIR4454	100616234	microRNA 4454	-1.602034	0.01136642	0.03698952
TC19000729.hg.1	CD37	951	CD37 molecule	-1.602171	0.000411247	0.002591274
TC08001641.hg.1	ASAP1	50807	ArfGAP with SH3 domain, ankyrin repeat and PH domain 1	-1.602251	0.000135791	0.001057105
TC12000555.hg.1	USP15	9958	ubiquitin specific peptidase 15	-1.602886	0.000740606	0.004198371
TC20000762.hg.1	PLAGL2	5326	pleiomorphic adenoma gene-like 2	-1.603084	1.81801E-05	0.000213921
TC01001276.hg.1	IL6R	3570	interleukin 6 receptor	-1.603349	0.000312123	0.002064869
TC15000754.hg.1	MESDC1	59274	mesoderm development candidate 1	-1.604431	3.15895E-05	0.000329764
TC18000074.hg.1	RNMT	8731	RNA (guanine-7-) methyltransferase	-1.60833	0.000442149	0.002741429
TC12001865.hg.1	UHRF1BP1L	23074	UHRF1 binding protein 1-like	-1.609644	0.005647841	0.02126536
TC12001168.hg.1	SLC2A14	144195	solute carrier family 2 (facilitated glucose transporter), member 14	-1.610717	0.004888434	0.0189167
TC01001147.hg.1	NBPF15	284565	neuroblastoma breakpoint family, member 16 -RefSeq NM_001102663 -Entrez_id 72893(	-1.611847	5.82875E-06	8.96239E-05
TC01006301.hg.1	NBPF11	200030	neuroblastoma breakpoint family, member 24 -RefSeq NM_001101663 -Entrez_id 728912	-1.612335	5.10669E-06	8.0705E-05
TC0X000220.hg.1	CHST7	56548	carbohydrate (N-acetylglucosamine 6-O) sulfotransferase 7	-1.612462	3.5787E-07	1.10298E-05
TC0X001025.hg.1	TFE3	7030	transcription factor binding to IGHM enhancer 3	-1.61257	0.000948308	0.005115379
TC13000064.hg.1	NUPL1	9818	nucleoporin like 1	-1.613334	0.004661473	0.01820988
TC11001948.hg.1	FOSL1	8061	FOS-like antigen 1	-1.614572	5.98634E-05	0.000545757
TC10000126.hg.1	VIM	7431	vimentin	-1.615406	1.1974E-08	1.04999E-06
TC06000108.hg.1	MYLIP	29116	myosin regulatory light chain interacting protein	-1.617157	0.000975879	0.005235187
TC02002577.hg.1	SESTD1	91404	SEC14 and spectrin domains 1	-1.619563	0.002330567	0.01045813
TC01002442.hg.1	MIR4420	100616164	microRNA 4420	-1.620009	0.003723019	0.01518928

transcript cluster id	Gene Symbol	Entrez Gene ID	Description	FoldChange (AIM/control)	P.Value (AIM/control)	adj.P.Val (AIM/control)
TC03000127.hg.1	NR1D2	9975	nuclear receptor subfamily 1, group D, member 2	-1.621327	0.001273759	0.006493482
TC05000633.hg.1	SLC22A4	6583	solute carrier family 22 (organic cation/ergothioneine transporter), member 4	-1.622103	0.01034467	0.03432952
TC15001352.hg.1	FLJ10038	55056	uncharacterized protein FLJ10038	-1.623465	0.009978036	0.03340058
TC06000918.hg.1	DSE	29940	dermatan sulfate epimerase	-1.624814	3.4142E-10	1.22343E-07
TC01003240.hg.1	S100A11	6282	S100 calcium binding protein A11	-1.625611	0.0139858	0.04364461
TC05000247.hg.1	GPBP1	65056	GC-rich promoter binding protein 1	-1.625937	2.79664E-06	5.06656E-05
TC05000665.hg.1	SEC24A	10802	SEC24 family, member A (S. cerevisiae)	-1.626492	0.002453561	0.01089357
TC19000118.hg.1	TRIP10	9322	thyroid hormone receptor interactor 10	-1.62702	2.58821E-08	1.72547E-06
TC05000526.hg.1	SNORA13	654322	small nucleolar RNA, H/ACA box 13	-1.627384	0.002067534	0.009510415
TC10000808.hg.1	PDCD4	27250	programmed cell death 4 (neoplastic transformation inhibitor)	-1.629992	0.000250838	0.001725594
TC05001776.hg.1	AFF4	27125	AF4/FMR2 family, member 4	-1.630414	4.11222E-05	0.000404233
TC17000884.hg.1	SCARNA16	677781	small Cajal body-specific RNA 16	-1.631139	0.000937121	0.005062326
TC02005047.hg.1	TTN	7273	titin	-1.631303	5.91601E-05	0.000540813
TC13000463.hg.1	LATS2	26524	large tumor suppressor kinase 2	-1.632206	0.000934936	0.005054735
TC10001783.hg.1	ADAM8	101	ADAM metallopeptidase domain 8	-1.632604	0.002088067	0.009584013
TC12001439.hg.1	VDR	7421	vitamin D (1,25- dihydroxyvitamin D3) receptor	-1.634661	0.005645121	0.02125872
TC19000350.hg.1	ARRDC2	27106	arrestin domain containing 2	-1.634901	0.000438099	0.002717804
TC11001544.hg.1	ABTB2	25841	ankyrin repeat and BTB (POZ) domain containing 2	-1.636884	0.000275424	0.00185972
TC10000865.hg.1	GRK5	2869	G protein-coupled receptor kinase 5	-1.63729	0.00637137	0.02344536
TC04001116.hg.1	RELL1	768211	RELT-like 1	-1.637863	0.000176933	0.001306883
TC19000339.hg.1	FAM129C	199786	family with sequence similarity 129, member C	-1.639418	1.3561E-06	2.91847E-05
TC19002654.hg.1	LILRB1	10859	leukocyte immunoglobulin-like receptor, subfamily B (with TM and ITIM domains), memb	-1.643115	0.000176703	0.001305332
TC15001230.hg.1	BMF	90427	Bcl2 modifying factor	-1.643164	0.002438316	0.01084344
TC07003336.hg.1	CDK14	5218	cyclin-dependent kinase 14	-1.643371	3.54153E-06	6.0674E-05
TC02001152.hg.1	PLCL1	5334	phospholipase C-like 1	-1.645934	0.002626033	0.01146616
TC03002054.hg.1	LAMP3	27074	lysosomal-associated membrane protein 3	-1.64629	0.005701897	0.02140937
TC19002659.hg.1	KIR2DL1	3802	killer cell immunoglobulin-like receptor, two domains, long cytoplasmic tail, 1	-1.646577	0.006649294	0.02419925
TC08001690.hg.1	SLC45A4	57210	solute carrier family 45, member 4	-1.647271	0.000277902	0.001873704
TC13000498.hg.1	MTMR6	9107	myotubularin related protein 6	-1.648665	3.62601E-05	0.000365138
TC09000169.hg.1	UBAP1	51271	ubiquitin associated protein 1	-1.649608	1.25842E-05	0.000160286
TC01003205.hg.1	MCL1	4170	myeloid cell leukemia sequence 1 (BCL2-related)	-1.650353	2.2401E-05	0.000250861
TC16000294.hg.1	IL21R	50615	interleukin 21 receptor	-1.651135	0.003889159	0.01570257
TC14000919.hg.1	SNORD8	319103	small nucleolar RNA, C/D box 8	-1.652357	0.01515261	0.04656233
TC20000399.hg.1	CEBPB	1051	CCAAT/enhancer binding protein (C/EBP), beta	-1.653758	0.002507145	0.01106515
TC19001458.hg.1	TYROBP	7305	TYRO protein tyrosine kinase binding protein	-1.65376	0.007520114	0.02666824
TC15002768.hg.1	LOC100507217	100507217	uncharacterized LOC100507217	-1.655196	0.003099637	0.01309572
TC11001395.hg.1	NRIP3	56675	nuclear receptor interacting protein 3	-1.656244	5.39192E-09	6.28259E-07
TC04000447.hg.1	PRDM8	56978	PR domain containing 8	-1.656736	5.41664E-07	1.47315E-05
TC19000454.hg.1	GRAMD1A	57655	GRAM domain containing 1A	-1.657046	0.000103678	0.00084435
TC04001328.hg.1	ANTXR2	118429	anthrax toxin receptor 2	-1.657362	1.34904E-07	5.56378E-06
TC07000251.hg.1	LINC00265	349114	long intergenic non-protein coding RNA 265	-1.660127	1.69395E-05	0.000201829
TC03000630.hg.1	CSTA	1475	cystatin A (stefin A)	-1.660884	0.000988291	0.005282837
TC01000194.hg.1	PLEKHM2	23207	pleckstrin homology domain containing, family M (with RUN domain) member 2	-1.661666	0.000171315	0.001273557
TC05001110.hg.1	LPCAT1	79888	lysophosphatidylcholine acyltransferase 1	-1.664486	0.000740248	0.004189582
TC06004102.hg.1	TAB2	23118	TGF-beta activated kinase 1/MAP3K7 binding protein 2	-1.665074	1.63299E-08	1.25397E-06
TC20000386.hg.1	ZFAS1	441951	ZNFX1 antisense RNA 1	-1.66549	8.23312E-05	0.000699578
TC19000195.hg.1	LPPR2	64748	lipid phosphate phosphatase-related protein type 2	-1.666129	0.0001049	0.000852532
TC02002914.hg.1	PER2	8864	period circadian clock 2	-1.666864	0.000532466	0.003192029
TC03001474.hg.1	SELK	58515	selenoprotein K	-1.667583	0.000244317	0.00168936
TC03001495.hg.1	ARF4	378	ADP-ribosylation factor 4	-1.6687	0.000155226	0.001175081
TC04000599.hg.1	SNHG8	100093630	small nucleolar RNA host gene 8 (non-protein coding)	-1.66962	0.000249336	0.001715912
TC17001922.hg.1	USP36	57602	ubiquitin specific peptidase 36	-1.670033	0.00270757	0.01175721
TC19000466.hg.1	FFAR1	2864	free fatty acid receptor 1	-1.670276	6.93438E-06	0.000101965
TC01001469.hg.1	XCL1	6375	chemokine (C motif) ligand 1	-1.673339	0.00934991	0.03169631
TC10000484.hg.1	SAMD8	142891	sterile alpha motif domain containing 8	-1.673374	0.000635226	0.003686578
TC01000555.hg.1	ATP6V0B	533	ATPase, H+ transporting, lysosomal 21kDa, V0 subunit b	-1.674178	0.008434628	0.02921311
TC07000098.hg.1	ARL4A	10124	ADP-ribosylation factor-like 4A	-1.674353	2.30804E-06	4.35698E-05
TC19002668.hg.1	ZNF776	284309	zinc finger protein 776	-1.675328	0.001879517	0.008822266
TC0X000180.hg.1	MID1IP1	58526	MID1 interacting protein 1	-1.67642	0.002382863	0.01065348
TC01000624.hg.1	RNF11	26994	ring finger protein 11	-1.676656	2.43397E-06	4.53905E-05
TC08000087.hg.1	BLK	640	B lymphoid tyrosine kinase	-1.67795	0.000579692	0.003424733
TC03000953.hg.1	MFN1	55669	mitofusin 1	-1.679059	0.001754685	0.008358391
TC15000050.hg.1	SNORD116-3	100033415	small nucleolar RNA, C/D box 116-3	-1.67936	0.009650085	0.03251067
TC15000055.hg.1	SNORD116-9	100033421	small nucleolar RNA, C/D box 116-9	-1.67936	0.009650085	0.03251067
TC0X001369.hg.1	RAP2C	57826	RAP2C, member of RAS oncogene family	-1.679541	6.52937E-06	9.72514E-05
TC06001165.hg.1	QKI	9444	QKI, KH domain containing, RNA binding	-1.680688	0.000146201	0.001117767
TC13000875.hg.1	RAB20	55647	RAB20, member RAS oncogene family	-1.680711	0.01598432	0.0485899
TC15000584.hg.1	USP3	9960	ubiquitin specific peptidase 3	-1.680769	0.000186256	0.001361365
TC07000161.hg.1	SNX10	29887	sorting nexin 10	-1.681517	0.000154828	0.001172983
TC07001970.hg.1	LOC100507507	100507507	uncharacterized LOC100507507	-1.685932	1.64816E-05	0.000197382
TC02000237.hg.1	QPCT	25797	glutaminyl-peptide cyclotransferase	-1.68608	0.00020114	0.001445832
TC19000882.hg.1	LILRB4	11006	leukocyte immunoglobulin-like receptor, subfamily B (with TM and ITIM domains), memb	-1.687172	3.91058E-06	6.50875E-05

transcript cluster id	Gene Symbol	Entrez Gene ID	Description	FoldChange (AIM/control)	P.Value (AIM/control)	adj.P.Val (AIM/control)
TC22000358.hg.1	RNU12	267010	RNA, U12 small nuclear	-1.688318	0.002924788	0.01247827
TC03001187.hg.1	IQSEC1	9922	IQ motif and Sec7 domain 1	-1.688966	3.81745E-05	0.00037965
TC14001116.hg.1	SOS2	6655	son of sevenless homolog 2 (Drosophila)	-1.69048	0.000105883	0.000858217
TC02001217.hg.1	ZDBF2	57683	zinc finger, DBF-type containing 2	-1.691701	0.00188579	0.008837184
TC07000777.hg.1	FLJ45340	402483	uncharacterized LOC402483	-1.693049	4.31054E-05	0.000418689
TC15000052.hg.1	SNORD116-2	100033414	small nucleolar RNA, C/D box 116-2	-1.697049	0.01638616	0.04950916
TC15000051.hg.1	SNORD116-5	100033417	small nucleolar RNA, C/D box 116-5	-1.697343	0.01249224	0.03991321
TC15000053.hg.1	SNORD116-7	100033419	small nucleolar RNA, C/D box 116-7	-1.697343	0.01249224	0.03991321
TC0X000992.hg.1	SLC9A7	84679	solute carrier family 9, subfamily A (NHE7, cation proton antiporter 7), member 7	-1.697347	0.001380373	0.006916091
TC15001339.hg.1	SECISBP2L	9728	SECIS binding protein 2-like	-1.700205	5.30793E-05	0.000495195
TC19002652.hg.1	LILRA2	11027	leukocyte immunoglobulin-like receptor, subfamily A (with TM domain), member 2	-1.700678	0.000489299	0.002974825
TC17000309.hg.1	WSB1	26118	WD repeat and SOCS box containing 1	-1.701533	0.00043351	0.002691752
TC02002101.hg.1	ASTL	431705	astacin-like metallo-endopeptidase (M12 family)	-1.703468	2.31164E-07	8.13218E-06
TC05001588.hg.1	LOC729040	729040	uncharacterized LOC729040	-1.704143	6.66312E-05	0.000589789
TC17001459.hg.1	NR1D1	9572	nuclear receptor subfamily 1, group D, member 1	-1.70461	0.00029133	0.001942428
TC06000532.hg.1	CDKN1A	1026	cyclin-dependent kinase inhibitor 1A (p21, Cip1)	-1.705431	6.85564E-06	0.000100855
TC17000897.hg.1	SYNGR2	9144	synaptogyrin 2	-1.705857	0.0002994	0.001985761
TC0X000324.hg.1	SNORA11	677799	small nucleolar RNA, H/ACA box 11	-1.706547	0.01082334	0.03555531
TC07000942.hg.1	FAM115C	285966	family with sequence similarity 115, member C	-1.707245	8.11353E-05	0.000690588
TC06000372.hg.1	LST1	7940	leukocyte specific transcript 1	-1.707247	0.00123006	0.006292845
TC14001451.hg.1	LGMN	5641	legumain	-1.708818	2.76074E-06	0.00005
TC10000008.hg.1	GTPBP4	23560	GTP binding protein 4	-1.710581	0.001491607	0.007349472
TC01000939.hg.1	KIAA1324	57535	KIAA1324	-1.712869	7.79593E-05	0.000667854
TC17000902.hg.1	PGS1	9489	phosphatidylglycerophosphate synthase 1	-1.713026	0.000231696	0.001614496
TC06000370.hg.1	LTA	4049	lymphotoxin alpha (TNF superfamily, member 1)	-1.713706	0.000171889	0.001273982
TC09001209.hg.1	ZFAND5	7763	zinc finger, AN1-type domain 5	-1.713997	8.70739E-07	2.07834E-05
TC13001719.hg.1	FOXO1	2308	forkhead box O1	-1.714497	4.02704E-05	0.000396161
TC02000221.hg.1	RASGRP3	25780	RAS guanyl releasing protein 3 (calcium and DAG-regulated)	-1.715259	1.26369E-06	2.74025E-05
TC0X001120.hg.1	CXorf65	158830	chromosome X open reading frame 65	-1.715555	0.000136205	0.00105563
TC19000758.hg.1	SPIB	6689	Spi-B transcription factor (Spi-1/PU.1 related)	-1.716225	9.51413E-06	0.000128681
TC11000631.hg.1	MALAT1	378938	metastasis associated lung adenocarcinoma transcript 1 (non-protein coding)	-1.720004	0.001426895	0.007108371
TC17001976.hg.1	CSNK1D	1453	casein kinase 1, delta	-1.720405	0.000363806	0.002330801
TC20000959.hg.1	SUMO1P1	391257	SUMO1 pseudogene 1	-1.720439	0.001546101	0.007557043
TC02000829.hg.1	PLEKHB2	55041	pleckstrin homology domain containing, family B (evectins) member 2	-1.720721	1.96412E-05	0.00022574
TC16002106.hg.1	SLC7A5P1	81893	solute carrier family 7 (amino acid transporter light chain, L system), member 5 pseudoge	-1.721992	0.000449799	0.002771894
TC01002977.hg.1	KCNA3	3738	potassium voltage-gated channel, shaker-related subfamily, member 3	-1.722674	0.001307967	0.006621203
TC17000992.hg.1	SNORD91B	692208	small nucleolar RNA, C/D box 91B	-1.723343	0.009795557	0.03287245
TC07000689.hg.1	HBP1	26959	HMG-box transcription factor 1	-1.725608	0.000705971	0.004025905
TC03000461.hg.1	CHMP2B	25978	charged multivesicular body protein 2B	-1.72643	0.003092186	0.01306413
TC01000116.hg.1	SLC25A33	84275	solute carrier family 25 (pyrimidine nucleotide carrier), member 33	-1.726757	0.000132672	0.001033175
TC08000298.hg.1	ADAM9	8754	ADAM metallopeptidase domain 9	-1.727379	0.000533745	0.003193667
TC06002209.hg.1	ZC3H12D	340152	zinc finger CCCH-type containing 12D	-1.728691	1.21855E-05	0.000155949
TC13000099.hg.1	USPL1	10208	ubiquitin specific peptidase like 1	-1.730429	0.000165207	0.00123313
TC01002163.hg.1	TNFRSF9	3604	tumor necrosis factor receptor superfamily, member 9	-1.730865	0.000287429	0.001921134
TC02000617.hg.1	MAP4K4	9448	mitogen-activated protein kinase kinase kinase kinase 4	-1.731265	5.41253E-07	1.46428E-05
TC05001385.hg.1	PLK2	10769	polo-like kinase 2	-1.732365	9.74376E-05	0.00079988
TC12001656.hg.1	C12orf61	283416	chromosome 12 open reading frame 61	-1.73629	6.69974E-09	7.25633E-07
TC15000054.hg.1	SNORD116-8	100033420	small nucleolar RNA, C/D box 116-8	-1.736698	0.009375837	0.03176161
TC01003204.hg.1	C1orf138	574406	chromosome 1 open reading frame 138	-1.738081	7.66536E-05	0.000660181
TC08001051.hg.1	TNFRSF10D	8793	tumor necrosis factor receptor superfamily, member 10d, decoy with truncated death do	-1.738962	2.91852E-05	0.000306941
TC19000256.hg.1	CD97	976	CD97 molecule	-1.740442	0.000784915	0.004375729
TC12001255.hg.1	DUSP16	80824	dual specificity phosphatase 16	-1.740689	4.17837E-05	0.000407221
TC14001115.hg.1	METTL21D	79609	methyltransferase like 21D	-1.741025	1.65255E-05	0.000197382
TC18000042.hg.1	ANKRD12	23253	ankyrin repeat domain 12	-1.741182	1.04854E-05	0.000138388
TC11000161.hg.1	SNORA45	677826	small nucleolar RNA, H/ACA box 45	-1.741556	0.000229628	0.00159945
TC0X000222.hg.1	RP2	6102	retinitis pigmentosa 2 (X-linked recessive)	-1.742047	0.001484633	0.007318479
TC15000150.hg.1	WHAMMP2	440253	WAS protein homolog associated with actin, golgi membranes and microtubules pseudog	-1.742397	1.22718E-06	2.67706E-05
TC14000431.hg.1	SNORD56B	319139	small nucleolar RNA, C/D box 56B	-1.745385	0.003837323	0.01551282
TC15000945.hg.1	ARRDC4	91947	arrestin domain containing 4	-1.745548	0.002197648	0.009963105
TC14001254.hg.1	ZFP36L1	677	ZFP36 ring finger protein-like 1	-1.746361	1.08154E-05	0.000141763
TC01003678.hg.1	ZNF281	23528	zinc finger protein 281	-1.746534	9.15695E-07	2.14491E-05
TC06000051.hg.1	LY86	9450	lymphocyte antigen 86	-1.746627	0.00610719	0.02267752
TC07000428.hg.1	SBDSP1	155370	Shwachman-Bodian-Diamond syndrome pseudogene 1	-1.747434	1.3919E-05	0.000172696
TC0X000518.hg.1	MIR548AN	100616144	microRNA 548an	-1.747533	0.001379391	0.006910087
TC02000197.hg.1	YPEL5	51646	yippee-like 5 (Drosophila)	-1.748577	0.000578728	0.003415202
TC19000318.hg.1	HSH2D	84941	hematopoietic SH2 domain containing	-1.749824	0.000145557	0.001111709
TC12001726.hg.1	ZFC3H1	196441	zinc finger, C3H1-type containing	-1.751054	5.32299E-05	0.000495195
TC05001053.hg.1	SQSTM1	8878	sequestosome 1	-1.752213	0.00020821	0.001481723
TC01006431.hg.1	NBPF9	400818	neuroblastoma breakpoint family, member 9	-1.754709	1.04089E-06	2.37109E-05
TC01002745.hg.1	MIR3671	100500854	microRNA 3671	-1.756311	1.38976E-05	0.000172443
TC06000058.hg.1	RIOK1	83732	RIO kinase 1	-1.758587	0.007023465	0.02526494
TC02000879.hg.1	RAB3GAP1	22930	RAB3 GTPase activating protein subunit 1 (catalytic)	-1.760697	3.23601E-06	5.59136E-05

transcript cluster id	Gene Symbol	Entrez Gene ID	Description	FoldChange (AIM/control)	P.Value (AIM/control)	adj.P.Val (AIM/control)
TC19001019.hg.1	MKNK2	2872	MAP kinase interacting serine/threonine kinase 2	-1.761399	0.000203869	0.001457302
TC07000125.hg.1	ITGB8	3696	integrin, beta 8	-1.763001	1.97792E-08	1.40511E-06
TC10000565.hg.1	ZMIZ1	57178	zinc finger, MIZ-type containing 1	-1.76348	3.3094E-05	0.000339929
TC10000057.hg.1	LOC399715	399715	uncharacterized LOC399715	-1.764256	8.82933E-05	0.000735318
TC10001145.hg.1	MIR604	693189	microRNA 604	-1.765153	0.000179686	0.001319125
TC05000682.hg.1	TGFBI	7045	transforming growth factor, beta-induced, 68kDa	-1.766775	0.000680759	0.003900341
TC0X000007.hg.1	MIR3690	100500894	microRNA 3690	-1.767896	1.97453E-05	0.000226135
TC0Y000007.hg.1	MIR3690	100500894	microRNA 3690	-1.767896	1.97453E-05	0.000226135
TC20000273.hg.1	SRC	6714	v-src sarcoma (Schmidt-Ruppin A-2) viral oncogene homolog (avian)	-1.769729	3.00733E-09	4.42055E-07
TC02000432.hg.1	DYSF	8291	dysferlin, limb girdle muscular dystrophy 2B (autosomal recessive)	-1.77088	0.01394402	0.04353734
TC02005036.hg.1	RNF103	7844	ring finger protein 103	-1.772204	0.000140388	0.001079478
TC22000835.hg.1	TNFRSF13C	115650	tumor necrosis factor receptor superfamily, member 13C	-1.772331	2.12138E-05	0.000239991
TC12003267.hg.1	ZNF10	7556	zinc finger protein 10	-1.772903	8.68666E-06	0.00012025
TC20000365.hg.1	CD40	958	CD40 molecule, TNF receptor superfamily member 5	-1.77561	3.46195E-06	5.88715E-05
TC01001138.hg.1	LOC100132999	100132999	uncharacterized LOC100132999	-1.775758	1.42759E-05	0.000176008
TC07000584.hg.1	BRI3	25798	brain protein I3	-1.776942	0.000208069	0.001480621
TC19001837.hg.1	MBOAT7	79143	membrane bound O-acyltransferase domain containing 7	-1.778018	0.000612998	0.003577651
TC19000812.hg.1	ZNF331	55422	zinc finger protein 331	-1.77915	1.27954E-05	0.00016124
TC02000521.hg.1	SNORD94	692225	small nucleolar RNA, C/D box 94	-1.779771	0.006927688	0.02497609
TC01003608.hg.1	RGS16	6004	regulator of G-protein signaling 16	-1.783157	5.54599E-09	6.28082E-07
TC06000273.hg.1	ZNF165	7718	zinc finger protein 165	-1.783245	1.21555E-07	5.09894E-06
TC03001089.hg.1	LOC100128262	100128262	uncharacterized LOC100128262	-1.784803	2.94655E-05	0.000308644
TC01000008.hg.1	LOC101060495	101060495	uncharacterized LOC101060495	-1.788105	4.56863E-05	0.000436597
TC11002116.hg.1	LRRC32	2615	leucine rich repeat containing 32	-1.789573	6.745E-07	1.7022E-05
TC20000405.hg.1	MIR645	693230	microRNA 645	-1.789785	0.001024896	0.005427454
TC15000659.hg.1	MIR630	693215	microRNA 630	-1.791012	7.52304E-07	1.85793E-05
TC08001049.hg.1	TNFRSF10B	8795	tumor necrosis factor receptor superfamily, member 10b	-1.791668	2.67648E-09	4.10895E-07
TC17001434.hg.1	CWC25	54883	CWC25 spliceosome-associated protein homolog (S. cerevisiae)	-1.794255	0.001084895	0.005693043
TC02000978.hg.1	GCA	25801	grancalcin, EF-hand calcium binding protein	-1.794525	0.005304883	0.02017182
TC15000436.hg.1	LINC00926	283663	long intergenic non-protein coding RNA 926	-1.794578	0.002297415	0.01031704
TC01006310.hg.1	NOTCH2NL	388677	notch 2 N-terminal like	-1.797801	1.89757E-06	3.73233E-05
TC12001826.hg.1	TMCC3	57458	transmembrane and coiled-coil domain family 3	-1.798073	0.001933898	0.009010651
TC12000914.hg.1	MAP1LC3B2	643246	microtubule-associated protein 1 light chain 3 beta 2	-1.798096	0.000278256	0.00186837
TC04000808.hg.1	FNIP2	57600	folliculin interacting protein 2	-1.802495	7.65321E-07	1.87855E-05
TC05001831.hg.1	ETF1	2107	eukaryotic translation termination factor 1	-1.802601	0.00110868	0.005791954
TC09001377.hg.1	AAED1	195827	AhpC/TSA antioxidant enzyme domain containing 1	-1.803857	1.59984E-06	3.28157E-05
TC10000269.hg.1	CSGALNACT2	55454	chondroitin sulfate N-acetylgalactosaminyltransferase 2	-1.80407	5.10474E-07	1.39869E-05
TC0X000199.hg.1	DDX3X	1654	DEAD (Asp-Glu-Ala-Asp) box polypeptide 3, X-linked	-1.804503	4.51769E-05	0.000432644
TC06002071.hg.1	SERINC1	57515	serine incorporator 1	-1.805095	1.52903E-05	0.000185791
TC02002452.hg.1	CYTIP	9595	cytohesin 1 interacting protein	-1.809601	0.000303315	0.002004955
TC11000484.hg.1	DTX4	23220	deltex homolog 4 (Drosophila)	-1.809618	3.68411E-05	0.000367517
TC19000120.hg.1	EMR1	2015	egf-like module containing, mucin-like, hormone receptor-like 1	-1.810967	0.005446146	0.02059984
TC01001777.hg.1	ATF3	467	activating transcription factor 3	-1.811906	0.009347906	0.03166338
TC03001071.hg.1	HES1	3280	hairy and enhancer of split 1, (Drosophila)	-1.812344	8.15217E-06	0.0001145
TC01000831.hg.1	PKN2	5586	protein kinase N2	-1.813165	6.34318E-06	9.48234E-05
TC10000257.hg.1	LOC399744	399744	uncharacterized LOC399744	-1.814334	3.39257E-06	5.77787E-05
TC15000690.hg.1	PPCDC	60490	phosphopantothenoylcysteine decarboxylase	-1.815944	1.99944E-06	3.87966E-05
TC01002155.hg.1	KLHL21	9903	kelch-like family member 21	-1.816469	1.88623E-06	3.71486E-05
TC02001922.hg.1	RAB1A	5861	RAB1A, member RAS oncogene family	-1.816651	0.00091827	0.004975664
TC05001746.hg.1	MARCH3	115123	membrane-associated ring finger (C3HC4) 3, E3 ubiquitin protein ligase	-1.818392	8.77811E-08	4.05377E-06
TC20000403.hg.1	PTPN1	5770	protein tyrosine phosphatase, non-receptor type 1	-1.821931	1.53329E-05	0.00018597
TC15002005.hg.1	CHSY1	22856	chondroitin sulfate synthase 1	-1.823394	0.000856025	0.004702894
TC19000585.hg.1	CEACAM3	1084	carcinoembryonic antigen-related cell adhesion molecule 3	-1.824566	0.001204766	0.006186186
TC14000915.hg.1	HNRNPC	3183	heterogeneous nuclear ribonucleoprotein C (C1/C2)	-1.82681	8.45315E-06	0.000117565
TC17001700.hg.1	SPAG9	9043	sperm associated antigen 9	-1.827424	0.000932868	0.005038526
TC0X000006.hg.1	CSF2RA	1438	colony stimulating factor 2 receptor, alpha, low-affinity (granulocyte-macrophage)	-1.827832	6.04551E-07	1.56597E-05
TC15000840.hg.1	AEN	64782	apoptosis enhancing nuclease	-1.830943	2.14348E-05	0.000241147
TC14001288.hg.1	NUMB	8650	numb homolog (Drosophila)	-1.831228	0.00014288	0.00109402
TC12001227.hg.1	YBX3	8531	Y box binding protein 3	-1.831569	6.16622E-05	0.000553577
TC19000937.hg.1	ZNF460	10794	zinc finger protein 460	-1.832676	1.45511E-06	3.04365E-05
TC08001656.hg.1	TMEM71	137835	transmembrane protein 71	-1.83676	0.000224179	0.00156535
TC02002244.hg.1	CCDC93	54520	coiled-coil domain containing 93	-1.839784	0.000916049	0.004967863
TC12001571.hg.1	ITGA5	3678	integrin, alpha 5 (fibronectin receptor, alpha polypeptide)	-1.840171	5.8594E-05	0.000533728
TC05002152.hg.1	HEIH	100859930	hepatocellular carcinoma up-regulated EZH2-associated long non-coding RNA	-1.841117	0.000627417	0.003638346
TC14001236.hg.1	ATP6V1D	51382	ATPase, H+ transporting, lysosomal 34kDa, V1 subunit D	-1.842094	0.003817134	0.01544478
TC01001587.hg.1	RGL1	23179	ral guanine nucleotide dissociation stimulator-like 1	-1.842628	0.00951784	0.03211306
TC04000524.hg.1	BANK1	55024	B-cell scaffold protein with ankyrin repeats 1	-1.845025	0.004788109	0.01854968
TC11000985.hg.1	ZC3H12C	85463	zinc finger CCCH-type containing 12C	-1.847164	4.07663E-07	1.19351E-05
TC02002256.hg.1	TMEM185B	79134	transmembrane protein 185B	-1.849079	0.000560978	0.003322158
TC08000591.hg.1	CPQ	10404	carboxypeptidase Q	-1.849386	2.03297E-05	0.000230446
TC02001174.hg.1	CFLAR	8837	CASP8 and FADD-like apoptosis regulator	-1.851911	2.97537E-07	9.53021E-06
TC01003193.hg.1	HIST2H2BE	8349	histone cluster 2, H2be	-1.852159	0.008569395	0.02955029

transcript cluster id	Gene Symbol	Entrez Gene ID	Description	FoldChange (AIM/control)	P.Value (AIM/control)	adj.P.Val (AIM/control)
TC12000730.hg.1	PLXNC1	10154	plexin C1	-1.852425	0.000878493	0.004804522
TC17001032.hg.1	CHRNE	1145	cholinergic receptor, nicotinic, epsilon (muscle)	-1.85265	4.64771E-07	1.30261E-05
TC01000510.hg.1	SMAP2	64744	small ArfGAP2	-1.854022	3.60012E-05	0.000360754
TC12000822.hg.1	TCP11L2	255394	t-complex 11, testis-specific-like 2	-1.855616	6.29119E-05	0.000561858
TC19001575.hg.1	CEACAM1	634	carcinoembryonic antigen-related cell adhesion molecule 1 (biliary glycoprotein)	-1.856789	0.0101457	0.0337642
TC12000512.hg.1	IL23A	51561	interleukin 23, alpha subunit p19	-1.858053	2.56735E-09	4.01975E-07
TC10001144.hg.1	SVIL	6840	supervillin	-1.858538	1.3168E-08	1.07713E-06
TC07001039.hg.1	INSIG1	3638	insulin induced gene 1	-1.860098	0.00017609	0.001294465
TC12001806.hg.1	C12orf79	256021	chromosome 12 open reading frame 79	-1.863446	0.000283705	0.001895653
TC05001315.hg.1	LOC100132356	100132356	uncharacterized LOC100132356	-1.864633	3.43051E-07	1.05648E-05
TC01001077.hg.1	LOC101060698	101060698	uncharacterized LOC101060698	-1.868169	0.000111575	0.00089256
TC09001691.hg.1	SLC2A6	11182	solute carrier family 2 (facilitated glucose transporter), member 6	-1.868851	2.44911E-07	8.34849E-06
TC17001883.hg.1	MIR4738	100616282	microRNA 4738	-1.870123	0.000608681	0.003553307
TC11001407.hg.1	SBF2	81846	SET binding factor 2	-1.871145	0.000603096	0.003524876
TC13000109.hg.1	FRY	10129	furry homolog (Drosophila)	-1.87177	6.81998E-05	0.000598541
TC07001639.hg.1	SMURF1	57154	SMAD specific E3 ubiquitin protein ligase 1	-1.872642	5.97248E-05	0.000540813
TC22000297.hg.1	MAFF	23764	v-maf musculoaponeurotic fibrosarcoma oncogene homolog F (avian)	-1.872867	9.01255E-07	2.10919E-05
TC15002769.hg.1	CHD2	1106	chromodomain helicase DNA binding protein 2	-1.873912	0.000352617	0.002266136
TC07001177.hg.1	TWISTNB	221830	TWIST neighbor	-1.874724	0.001482325	0.007298546
TC03000188.hg.1	OXSR1	9943	oxidative stress responsive 1	-1.875357	0.000222073	0.001553851
TC03001649.hg.1	BTLA	151888	B and T lymphocyte associated	-1.876913	0.000976111	0.005212459
TC17000055.hg.1	C17orf107	100130311	chromosome 17 open reading frame 107	-1.878431	4.40977E-07	1.25921E-05
TC0X000310.hg.1	TSPYL2	64061	TSPY-like 2	-1.878525	0.007836806	0.02753207
TC0X001162.hg.1	RLIM	51132	ring finger protein, LIM domain interacting	-1.880325	2.29106E-05	0.000253035
TC10000168.hg.1	OTUD1	220213	OTU domain containing 1	-1.880605	3.60263E-08	2.1218E-06
TC15001350.hg.1	GABPB1	2553	GA binding protein transcription factor, beta subunit 1	-1.881081	0.001830343	0.008631847
TC07001927.hg.1	BRAF	673	v-raf murine sarcoma viral oncogene homolog B1	-1.88185	1.27076E-05	0.00016003
TC07000679.hg.1	KMT2E	55904	lysine (K)-specific methyltransferase 2E	-1.882718	1.02277E-06	2.32166E-05
TC15001051.hg.1	WHAMMP3	339005	WAS protein homolog associated with actin, golgi membranes and microtubules pseudog	-1.882983	4.7387E-06	7.51438E-05
TC12000868.hg.1	SH2B3	10019	SH2B adaptor protein 3	-1.884112	1.67524E-06	3.39894E-05
TC15001176.hg.1	WHAMMP1	100288615	WAS protein homolog associated with actin, golgi membranes and microtubules pseudog	-1.885382	4.83473E-05	0.000455445
TC16000870.hg.1	LITAF	9516	lipopolysaccharide-induced TNF factor	-1.88909	6.21588E-05	0.000556445
TC11000229.hg.1	NCR3LG1	374383	natural killer cell cytotoxicity receptor 3 ligand 1	-1.890883	2.50028E-06	4.57495E-05
TC06002265.hg.1	EZR	7430	ezrin	-1.891733	0.00013593	0.001049931
TC19000865.hg.1	MYADM	91663	myeloid-associated differentiation marker	-1.892496	0.0007596	0.004264638
TC07001729.hg.1	LOC100216545	100216545	uncharacterized LOC100216545	-1.894086	6.98399E-08	3.37462E-06
TC01001382.hg.1	FCER1G	2207	Fc fragment of IgE, high affinity I, receptor for; gamma polypeptide	-1.894545	0.000803028	0.004448677
TC22000149.hg.1	UPB1	51733	ureidopropionase, beta	-1.894657	9.88264E-05	0.000806771
TC17000386.hg.1	CCL8	6355	chemokine (C-C motif) ligand 8	-1.896518	0.005275482	0.02005885
TC05000389.hg.1	JMY	133746	junction mediating and regulatory protein, p53 cofactor	-1.897369	0.000595571	0.003487759
TC11001073.hg.1	CXCR5	643	chemokine (C-X-C motif) receptor 5	-1.898125	1.51638E-06	3.13277E-05
TC06001777.hg.1	TNFRSF21	27242	tumor necrosis factor receptor superfamily, member 21	-1.908115	6.11617E-09	6.63579E-07
TC15000581.hg.1	RAB8B	51762	RAB8B, member RAS oncogene family	-1.909077	0.003245841	0.01358279
TC14000433.hg.1	SIPA1L1	26037	signal-induced proliferation-associated 1 like 1	-1.912288	0.000595156	0.003486592
TC18000527.hg.1	TCF4	6925	transcription factor 4	-1.912489	6.33086E-08	3.17446E-06
TC17001808.hg.1	GNA13	10672	guanine nucleotide binding protein (G protein), alpha 13	-1.915733	9.87909E-06	0.000131488
TC16000355.hg.1	FBRS	64319	fibrosin	-1.91574	0.000173731	0.001278999
TC0X000051.hg.1	TLR7	51284	toll-like receptor 7	-1.917686	0.001990951	0.009198643
TC07001349.hg.1	SNORA9	677798	small nucleolar RNA, H/ACA box 9	-1.917839	3.39627E-06	5.75816E-05
TC05003427.hg.1	FNIP1	96459	folliculin interacting protein 1	-1.919595	0.001331698	0.006704183
TC17001903.hg.1	ST6GALNAC2	10610	ST6 (alpha-N-acetyl-neuraminyl-2,3-beta-galactosyl-1,3)-N-acetylgalactosaminide alpha-2,	-1.919967	3.27705E-05	0.000335995
TC07001467.hg.1	SBDS	51119	Shwachman-Bodian-Diamond syndrome	-1.921163	6.03632E-06	9.06295E-05
TC05002142.hg.1	RNF130	55819	ring finger protein 130	-1.921778	0.000743542	0.004189582
TC08001015.hg.1	ASAH1	427	N-acylsphingosine amidohydrolase (acid ceramidase) 1	-1.921818	0.003299792	0.01376135
TC19002614.hg.1	LILRB3	11025	leukocyte immunoglobulin-like receptor, subfamily B (with TM and ITIM domains), memb	-1.921905	7.88724E-06	0.000111467
TC10001312.hg.1	IPMK	253430	inositol polyphosphate multikinase	-1.924093	0.000515116	0.003090394
TC18000223.hg.1	ZCCHC2	54877	zinc finger, CCHC domain containing 2	-1.924344	0.01076418	0.03535633
TC17000165.hg.1	HS3ST3B1	9953	heparan sulfate (glucosamine) 3-O-sulfotransferase 3B1	-1.925905	4.90069E-05	0.000459955
TC22000796.hg.1	JOSD1	9929	Josephin domain containing 1	-1.927577	2.95022E-05	0.000308
TC01003706.hg.1	KDM5B	10765	lysine (K)-specific demethylase 5B	-1.927646	6.69533E-05	0.000589294
TC01003359.hg.1	ETV3	2117	ets variant 3	-1.928439	1.9697E-06	3.82107E-05
TC08001120.hg.1	RNF122	79845	ring finger protein 122	-1.928621	0.000126618	0.000990327
TC01003847.hg.1	DUSP10	11221	dual specificity phosphatase 10	-1.930064	0.001416149	0.00704733
TC08001684.hg.1	AGO2	27161	argonaute RISC catalytic component 2	-1.931232	8.66029E-05	0.000721803
TC15000049.hg.1	SNORD116-2	100033414	small nucleolar RNA, C/D box 116-2	-1.933659	0.008786194	0.03017397
TC15000060.hg.1	SNORD116-14	100033426	small nucleolar RNA, C/D box 116-14	-1.93424	0.00764369	0.02699436
TC15000949.hg.1	IGF1R	3480	insulin-like growth factor 1 receptor	-1.934995	5.94476E-05	0.000538369
TC12000113.hg.1	NECAP1	25977	NECAP endocytosis associated 1	-1.937133	0.000502313	0.00302743
TC06001146.hg.1	WTAP	9589	Wilms tumor 1 associated protein	-1.941038	9.6989E-06	0.000129464
TC02000874.hg.1	MGAT5	4249	mannosyl (alpha-1,6-)-glycoprotein beta-1,6-N-acetyl-glucosaminyltransferase	-1.941074	1.74373E-05	0.000204406
TC17001882.hg.1	H3F3B	3021	H3 histone, family 3B (H3.3B)	-1.942137	0.000211635	0.001495546
TC18000268.hg.1	ADNP2	22850	ADNP homeobox 2	-1.942166	0.000388417	0.002453872

transcript cluster id	Gene Symbol	Entrez Gene ID	Description	FoldChange (AIM/control)	P.Value (AIM/control)	adj.P.Val (AIM/control)
TC07000840.hg.1	TMEM140	55281	transmembrane protein 140	-1.943892	0.0061271	0.02271461
TC05001834.hg.1	SNORD63	26785	small nucleolar RNA, C/D box 63	-1.947968	0.001999467	0.009223485
TC01000503.hg.1	MFSD2A	84879	major facilitator superfamily domain containing 2A	-1.949123	5.85199E-06	8.84952E-05
TC04000517.hg.1	DAPP1	27071	dual adaptor of phosphotyrosine and 3-phosphoinositides	-1.950055	0.001463101	0.00722998
TC01002506.hg.1	STK40	83931	serine/threonine kinase 40	-1.9527	7.69485E-06	0.00010934
TC12000956.hg.1	P2RX4	5025	purinergic receptor P2X, ligand-gated ion channel, 4	-1.953959	2.55987E-06	4.64963E-05
TC01001254.hg.1	S100A9	6280	S100 calcium binding protein A9	-1.956118	0.000557073	0.003302884
TC11001941.hg.1	AP5B1	91056	adaptor-related protein complex 5, beta 1 subunit	-1.957386	0.000987007	0.005256502
TC11000184.hg.1	AMPD3	272	adenosine monophosphate deaminase 3	-1.965478	6.84371E-06	9.98235E-05
TC19000237.hg.1	IER2	9592	immediate early response 2	-1.966409	7.83909E-05	0.000667322
TC04000362.hg.1	STAP1	26228	signal transducing adaptor family member 1	-1.967629	5.37784E-05	0.000495944
TC20000387.hg.1	SNORD12C	26765	small nucleolar RNA, C/D box 12C	-1.96959	4.56317E-05	0.00043426
TC18000556.hg.1	KDSR	2531	3-ketodihydrosphingosine reductase	-1.970664	0.000505861	0.003041505
TC06000950.hg.1	SMPDL3A	10924	sphingomyelin phosphodiesterase, acid-like 3A	-1.972012	0.000108121	0.000868239
TC12001796.hg.1	DUSP6	1848	dual specificity phosphatase 6	-1.972422	0.003723523	0.01514512
TC14000784.hg.1	RCOR1	23186	REST corepressor 1	-1.972557	4.47274E-05	0.00042737
TC15000580.hg.1	LACTB	114294	lactamase, beta	-1.974362	0.001921038	0.008958901
TC01006339.hg.1	FCGR2C	9103	Fc fragment of IgG, low affinity IIc, receptor for (CD32) (gene/pseudogene)	-1.97454	0.000469242	0.002862647
TC14001255.hg.1	ACTN1	87	actinin, alpha 1	-1.978696	0.000312108	0.002051191
TC14000056.hg.1	PNP	4860	purine nucleoside phosphorylase	-1.979183	8.33411E-05	0.000701056
TC11002211.hg.1	MAML2	84441	mastermind-like 2 (Drosophila)	-1.979887	0.000442559	0.002723109
TC16000939.hg.1	IGSF6	10261	immunoglobulin superfamily, member 6	-1.980146	0.007669816	0.02707156
TC13000273.hg.1	KLF5	688	Kruppel-like factor 5 (intestinal)	-1.980246	9.28691E-05	0.000765159
TC07001582.hg.1	STEAP4	79689	STEAP family member 4	-1.981695	0.01046365	0.03454163
TC06000541.hg.1	PIM1	5292	pim-1 oncogene	-1.985921	5.02404E-06	7.81048E-05
TC19001116.hg.1	FCER2	2208	Fc fragment of IgE, low affinity II, receptor for (CD23)	-1.988309	4.524E-06	7.2202E-05
TC17000486.hg.1	CSF3	1440	colony stimulating factor 3 (granulocyte)	-1.990011	0.000144705	0.001102613
TC05000253.hg.1	GAPT	202309	GRB2-binding adaptor protein, transmembrane	-1.991759	0.002438397	0.01081238
TC04000678.hg.1	CCRN4L	25819	CCR4 carbon catabolite repression 4-like (S. cerevisiae)	-1.992855	2.33774E-06	4.33369E-05
TC14000798.hg.1	SNORA28	677811	small nucleolar RNA, H/ACA box 28	-1.994785	6.73527E-05	0.000590828
TC08001440.hg.1	TP53INP1	94241	tumor protein p53 inducible nuclear protein 1	-1.996523	0.001152205	0.005955031
TC07000111.hg.1	TSPAN13	27075	tetraspanin 13	-1.999693	6.99206E-06	0.000101275
TC12002122.hg.1	SLC15A4	121260	solute carrier family 15, member 4	-2.001632	0.00103692	0.005468315
TC03001939.hg.1	CCNL1	57018	cyclin L1	-2.002305	7.72935E-05	0.000660877
TC06000104.hg.1	JARID2	3720	jumonji, AT rich interactive domain 2	-2.006635	2.77508E-05	0.000294016
TC20000702.hg.1	THBD	7056	thrombomodulin	-2.010578	0.0080796	0.02820919
TC19000545.hg.1	PLEKHG2	64857	pleckstrin homology domain containing, family G (with RhoGef domain) member 2	-2.014037	6.53083E-07	1.65198E-05
TC04001137.hg.1	RBM47	54502	RNA binding motif protein 47	-2.016225	3.46114E-05	0.000348563
TC08000412.hg.1	CHD7	55636	chromodomain helicase DNA binding protein 7	-2.017889	1.34597E-05	0.00016676
TC09001008.hg.1	B4GALT1	2683	UDP-Gal:betaGlcNAc beta 1,4- galactosyltransferase, polypeptide 1	-2.019066	2.13156E-05	0.000239348
TC08000835.hg.1	GRINA	2907	glutamate receptor, ionotropic, N-methyl D-aspartate-associated protein 1 (glutamate bir	-2.02179	0.000136857	0.001053988
TC17001711.hg.1	MMD	23531	monocyte to macrophage differentiation-associated	-2.021881	2.52574E-05	0.000272576
TC17001014.hg.1	P2RX1	5023	purinergic receptor P2X, ligand-gated ion channel, 1	-2.024197	0.003828386	0.01546491
TC06004068.hg.1	TRIM39	56658	tripartite motif containing 39	-2.025802	9.92988E-07	2.25209E-05
TC03001684.hg.1	CD80	941	CD80 molecule	-2.027036	3.15379E-07	9.8224E-06
TC0Y000008.hg.1	IL3RA	3563	interleukin 3 receptor, alpha (low affinity)	-2.028163	1.015E-07	4.41028E-06
TC05000967.hg.1	CPEB4	80315	cytoplasmic polyadenylation element binding protein 4	-2.028774	0.000209063	0.001480621
TC16000812.hg.1	MEFV	4210	Mediterranean fever	-2.030629	0.001492567	0.007330811
TC20000582.hg.1	RNF24	11237	ring finger protein 24	-2.032565	0.002498683	0.01101675
TC0X000219.hg.1	ZNF674-AS1	401588	ZNF674 antisense RNA 1 (head to head)	-2.034299	3.11666E-08	1.88516E-06
TC01003720.hg.1	LOC730227	730227	uncharacterized LOC730227	-2.03522	9.59892E-10	2.02999E-07
TC10000946.hg.1	INPP5A	3632	inositol polyphosphate-5-phosphatase, 40kDa	-2.038352	8.18233E-06	0.000114057
TC0X000963.hg.1	BCOR	54880	BCL6 corepressor	-2.040671	3.38943E-05	0.000343723
TC01001985.hg.1	ZBTB18	10472	zinc finger and BTB domain containing 18	-2.040852	0.001412462	0.007028335
TC14000797.hg.1	EIF5	1983	eukaryotic translation initiation factor 5	-2.041371	3.82604E-06	6.32996E-05
TC19001500.hg.1	RASGRP4	115727	RAS guanyl releasing protein 4	-2.041633	0.000407181	0.002549612
TC16000862.hg.1	SOCS1	8651	suppressor of cytokine signaling 1	-2.046291	3.42385E-05	0.000346067
TC03000407.hg.1	KBTBD8	84541	kelch repeat and BTB (POZ) domain containing 8	-2.046691	8.47506E-06	0.000117122
TC0X000087.hg.1	SCML1	6322	sex comb on midleg-like 1 (Drosophila)	-2.047535	7.10051E-07	1.75368E-05
TC22000304.hg.1	GTPBP1	9567	GTP binding protein 1	-2.048546	1.53479E-05	0.00018526
TC17000103.hg.1	CD68	968	CD68 molecule	-2.049007	8.46087E-05	0.000708686
TC03001546.hg.1	FOXP1	27086	forkhead box P1	-2.051331	1.55582E-05	0.000187159
TC14001124.hg.1	PYGL	5836	phosphorylase, glycogen, liver	-2.051835	0.008455942	0.02921311
TC14002198.hg.1	SNAPC1	6617	small nuclear RNA activating complex, polypeptide 1, 43kDa	-2.052006	0.000325515	0.002119724
TC01004036.hg.1	LOC731275	731275	uncharacterized LOC731275	-2.053797	8.2827E-06	0.000115113
TC20000244.hg.1	TP53INP2	58476	tumor protein p53 inducible nuclear protein 2	-2.054513	5.8073E-08	2.9444E-06
TC01002819.hg.1	CTBS	1486	chitobiase, di-N-acetyl-	-2.05608	0.000211582	0.001493911
TC03000926.hg.1	FNDC3B	64778	fibronectin type III domain containing 3B	-2.05826	4.86084E-05	0.000456038
TC22000667.hg.1	PIK3IP1	113791	phosphoinositide-3-kinase interacting protein 1	-2.05967	1.75486E-06	3.49863E-05
TC10000293.hg.1	ALOX5	240	arachidonate 5-lipoxygenase	-2.061417	0.000858763	0.004705201
TC03000096.hg.1	EAF1	85403	ELL associated factor 1	-2.061634	0.000278489	0.001863104
TC02000902.hg.1	KYNU	8942	kynureninase	-2.063934	0.003594542	0.01472754

transcript cluster id	Gene Symbol	Entrez Gene ID	Description	FoldChange (AIM/control)	P.Value (AIM/control)	adj.P.Val (AIM/control)
TC18000500.hg.1	SMAD7	4092	SMAD family member 7	-2.069863	2.53805E-05	0.000273239
TC0X000008.hg.1	IL3RA	3563	interleukin 3 receptor, alpha (low affinity)	-2.0703	9.03309E-08	4.06501E-06
TC01000372.hg.1	THEMIS2	9473	thymocyte selection associated family member 2	-2.072171	0.002525647	0.01108857
TC12001415.hg.1	NELL2	4753	NEL-like 2 (chicken)	-2.080017	0.000630658	0.003645669
TC02002136.hg.1	AFF3	3899	AF4/FMR2 family, member 3	-2.082046	7.92812E-05	0.000671951
TC19002665.hg.1	ZNF134	7693	zinc finger protein 134	-2.084077	6.13396E-05	0.00054938
TC05001505.hg.1	SNORA47	677828	small nucleolar RNA, H/ACA box 47	-2.087018	0.000416614	0.002597904
TC11001736.hg.1	SPI1	6688	spleen focus forming virus (SFFV) proviral integration oncogene spi1	-2.091143	0.000167669	0.001240221
TC12002004.hg.1	MED13L	23389	mediator complex subunit 13-like	-2.091776	7.29876E-08	3.4311E-06
TC09002922.hg.1	RALGDS	5900	ral guanine nucleotide dissociation stimulator	-2.094414	0.000291492	0.001932426
TC17001907.hg.1	JMJD6	23210	jumonji domain containing 6	-2.097487	6.05713E-05	0.000544659
TC15001596.hg.1	ANP32A-IT1	80035	ANP32A intronic transcript 1 (non-protein coding)	-2.097988	0.000120606	0.000948264
TC12001925.hg.1	CRY1	1407	cryptochrome 1 (photolyase-like)	-2.098253	4.40034E-05	0.000422105
TC06000423.hg.1	PHF1	5252	PHD finger protein 1	-2.100234	4.75888E-06	7.49842E-05
TC10000801.hg.1	DUSP5	1847	dual specificity phosphatase 5	-2.103804	0.001906169	0.008889631
TC08000180.hg.1	LOC254896	254896	uncharacterized LOC254896	-2.107265	0.00126214	0.006409694
TC02000968.hg.1	TANK	10010	TRAF family member-associated NFKB activator	-2.111095	0.002532257	0.01111072
TC17001729.hg.1	MIR142	406934	microRNA 142	-2.113797	0.000425458	0.002639711
TC17001857.hg.1	CD300LB	124599	CD300 molecule-like family member b	-2.117622	0.000203348	0.001447951
TC01002421.hg.1	PTAFR	5724	platelet-activating factor receptor	-2.119609	9.34045E-06	0.000125697
TC02000528.hg.1	RGPD1	400966	RANBP2-like and GRIP domain containing 1	-2.122403	2.27771E-06	4.23613E-05
TC06001217.hg.1	SERPINB1	1992	serpin peptidase inhibitor, clade B (ovalbumin), member 1	-2.122653	1.16157E-05	0.000148209
TC10000093.hg.1	CAMK1D	57118	calcium/calmodulin-dependent protein kinase ID	-2.127649	3.5918E-07	1.06963E-05
TC10001659.hg.1	PDCD4-AS1	282997	PDCD4 antisense RNA 1	-2.12983	4.88801E-07	1.3369E-05
TC02000923.hg.1	ACVR2A	92	activin A receptor, type IIA	-2.129933	0.000109265	0.000874456
TC02002064.hg.1	RGPD1	400966	RANBP2-like and GRIP domain containing 1	-2.130632	3.03858E-06	5.25697E-05
TC18000047.hg.1	RAB31	11031	RAB31, member RAS oncogene family	-2.130647	0.009155759	0.03112501
TC0Y000006.hg.1	CSF2RA	1438	colony stimulating factor 2 receptor, alpha, low-affinity (granulocyte-macrophage)	-2.130975	9.73513E-08	4.262E-06
TC01001619.hg.1	RGS18	64407	regulator of G-protein signaling 18	-2.131132	0.001200221	0.006151914
TC17000868.hg.1	UBALD2	283991	UBA-like domain containing 2	-2.131556	5.51767E-06	8.40176E-05
TC19000228.hg.1	JUNB	3726	jun B proto-oncogene	-2.131657	0.000114157	0.000905648
TC19001784.hg.1	SIGLEC14	100049587	sialic acid binding Ig-like lectin 14	-2.132654	1.21661E-06	2.62351E-05
TC0X002305.hg.1	CRLF2	64109	cytokine receptor-like factor 2	-2.133115	2.21083E-05	0.000245174
TC0Y000347.hg.1	CRLF2	64109	cytokine receptor-like factor 2	-2.133115	2.21083E-05	0.000245174
TC01003260.hg.1	S100A12	6283	S100 calcium binding protein A12	-2.133297	0.004906163	0.0189167
TC20000051.hg.1	PRNP	5621	prion protein	-2.135314	3.53632E-07	1.06369E-05
TC06000135.hg.1	SOX4	6659	SRY (sex determining region Y)-box 4	-2.138899	9.88762E-06	0.000131066
TC02005063.hg.1	BAZ2B	29994	bromodomain adjacent to zinc finger domain, 2B	-2.140397	2.60455E-05	0.000278773
TC06001094.hg.1	PLEKHG1	57480	pleckstrin homology domain containing, family G (with RhoGef domain) member 1	-2.141448	0.000111008	0.000885879
TC19000638.hg.1	RELB	5971	v-rel reticuloendotheliosis viral oncogene homolog B	-2.141876	2.18129E-06	4.09657E-05
TC04001813.hg.1	LOC100506229	100506229	uncharacterized LOC100506229	-2.145177	3.54518E-07	1.06369E-05
TC09000490.hg.1	TDRD7	23424	tudor domain containing 7	-2.14603	0.000731489	0.004132915
TC09001228.hg.1	GNAQ	2776	guanine nucleotide binding protein (G protein), q polypeptide	-2.148691	4.84521E-06	7.58793E-05
TC17000959.hg.1	METRNL	284207	meteorin, glial cell differentiation regulator-like	-2.148771	0.000920777	0.004975664
TC06000005.hg.1	IRF4	3662	interferon regulatory factor 4	-2.148823	0.006634069	0.02412225
TC03000862.hg.1	PTX3	5806	pentraxin 3, long	-2.15079	8.00809E-06	0.000112123
TC14000332.hg.1	PELI2	57161	pellino E3 ubiquitin protein ligase family member 2	-2.157905	2.7012E-08	1.70216E-06
TC07001740.hg.1	CCDC71L	168455	coiled-coil domain containing 71-like	-2.16208	4.98578E-07	1.35254E-05
TC16000674.hg.1	MAP1LC3B	81631	microtubule-associated protein 1 light chain 3 beta	-2.166215	7.76106E-05	0.000661552
TC19001290.hg.1	ELL	8178	elongation factor RNA polymerase II	-2.166348	0.004751929	0.01842569
TC11001401.hg.1	LOC644656	644656	uncharacterized LOC644656	-2.171409	4.06082E-07	1.17479E-05
TC14001564.hg.1	GPR132	29933	G protein-coupled receptor 132	-2.171414	1.52259E-07	5.87023E-06
TC01003406.hg.1	SLAMF1	6504	signaling lymphocytic activation molecule family member 1	-2.172887	7.60065E-06	0.000107769
TC17000727.hg.1	VMP1	81671	vacuole membrane protein 1	-2.172895	9.93628E-05	0.000807177
TC03000087.hg.1	SLC6A6	6533	solute carrier family 6 (neurotransmitter transporter, taurine), member 6	-2.173209	0.000296548	0.001960052
TC16000381.hg.1	CLUHP3	100132341	clustered mitochondria (cluA/CLU1) homolog pseudogene 3	-2.173445	6.45552E-08	3.17609E-06
TC19002672.hg.1	LRG1	116844	leucine-rich alpha-2-glycoprotein 1	-2.175464	0.002339064	0.01044835
TC02001600.hg.1	FAM49A	81553	family with sequence similarity 49, member A	-2.177247	3.35663E-05	0.000340795
TC01000955.hg.1	CSF1	1435	colony stimulating factor 1 (macrophage)	-2.178246	1.53693E-05	0.00018516
TC11001829.hg.1	PATL1	219988	protein associated with topoisomerase II homolog 1 (yeast)	-2.179036	6.22333E-05	0.00055482
TC05000226.hg.1	SNX18	112574	sorting nexin 18	-2.179117	3.88208E-05	0.000380709
TC04001592.hg.1	TBC1D9	23158	TBC1 domain family, member 9 (with GRAM domain)	-2.183678	8.17112E-05	0.000688543
TC02002172.hg.1	RGPD3	653489	RANBP2-like and GRIP domain containing 3	-2.186745	6.34987E-07	1.60644E-05
TC16000230.hg.1	SNX29P1	100652781	sorting nexin 29 pseudogene 1	-2.190996	3.66869E-06	6.10442E-05
TC11000957.hg.1	BIRC2	329	baculoviral IAP repeat containing 2	-2.195848	1.56006E-07	5.97853E-06
TC11003506.hg.1	CARD16	114769	caspase recruitment domain family, member 16	-2.196137	0.000137895	0.001057977
TC01006389.hg.1	ARID4B	51742	AT rich interactive domain 4B (RBP1-like)	-2.201701	1.36978E-07	5.41993E-06
TC20000554.hg.1	SIRPB1	10326	signal-regulatory protein beta 1	-2.202973	0.000181337	0.001320855
TC06001502.hg.1	PPP1R10	5514	protein phosphatase 1, regulatory subunit 10	-2.203721	1.33975E-05	0.000165934
TC07001347.hg.1	PURB	5814	purine-rich element binding protein B	-2.204276	8.64012E-07	2.02311E-05
TC15001192.hg.1	SLC12A6	9990	solute carrier family 12 (potassium/chloride transporters), member 6	-2.209268	7.21386E-07	1.76629E-05
TC04000140.hg.1	CPEB2	132864	cytoplasmic polyadenylation element binding protein 2	-2.212949	2.67108E-05	0.000284424

transcript cluster id	Gene Symbol	Entrez Gene ID	Description	FoldChange (AIM/control)	P.Value (AIM/control)	adj.P.Val (AIM/control)
TC01003874.hg.1	TP53BP2	7159	tumor protein p53 binding protein, 2	-2.213542	7.68613E-06	0.000108673
TC01003745.hg.1	NUAK2	81788	NUAK family, SNF1-like kinase, 2	-2.216528	0.000191435	0.001380525
TC22000476.hg.1	BID	637	BH3 interacting domain death agonist	-2.222115	0.000687174	0.003916553
TC18000278.hg.1	YES1	7525	v-yes-1 Yamaguchi sarcoma viral oncogene homolog 1	-2.222171	0.009973625	0.03330554
TC11003507.hg.1	CARD17	440068	caspase recruitment domain family, member 17	-2.23026	0.006220906	0.02296073
TC11002205.hg.1	SESN3	143686	sestrin 3	-2.23035	0.000320673	0.002091322
TC02001686.hg.1	FTH1P3	2498	ferritin, heavy polypeptide 1 pseudogene 3	-2.235287	1.03837E-06	2.32249E-05
TC10000323.hg.1	WDFY4	57705	WDFY family member 4	-2.236243	6.04167E-06	9.00454E-05
TC0X000238.hg.1	TIMP1	7076	TIMP metallopeptidase inhibitor 1	-2.237648	1.23979E-06	2.65637E-05
TC20000235.hg.1	CHMP4B	128866	charged multivesicular body protein 4B	-2.237682	4.32541E-06	6.94096E-05
TC15000695.hg.1	NEIL1	79661	nei endonuclease VIII-like 1 (E. coli)	-2.244024	1.41335E-05	0.00017276
TC01001544.hg.1	RALGPS2	55103	Ral GEF with PH domain and SH3 binding motif 2	-2.24433	0.004250262	0.01679646
TC11000332.hg.1	CD44	960	CD44 molecule (Indian blood group)	-2.246369	2.09491E-06	3.96815E-05
TC10001725.hg.1	CHST15	51363	carbohydrate (N-acetylgalactosamine 4-sulfate 6-O) sulfotransferase 15	-2.247342	0.001880374	0.008794236
TC01001399.hg.1	FCGR2A	2212	Fc fragment of IgG, low affinity IIa, receptor (CD32)	-2.250073	0.000561428	0.003316365
TC05001689.hg.1	FEM1C	56929	fem-1 homolog c (C. elegans)	-2.253059	0.000473192	0.0028761
TC02000660.hg.1	RGPD4	285190	RANBP2-like and GRIP domain containing 4	-2.253642	4.16854E-07	1.1953E-05
TC06000697.hg.1	PTP4A1	7803	protein tyrosine phosphatase type IVA, member 1	-2.258341	3.01982E-05	0.000312198
TC10000449.hg.1	DDIT4	54541	DNA-damage-inducible transcript 4	-2.25962	0.001277946	0.006476636
TC01001346.hg.1	MNDA	4332	myeloid cell nuclear differentiation antigen	-2.25986	0.009003742	0.03073036
TC16000879.hg.1	CPPED1	55313	calcineurin-like phosphoesterase domain containing 1	-2.260826	0.00051361	0.00307471
TC03000164.hg.1	CCR4	1233	chemokine (C-C motif) receptor 4	-2.261725	8.13438E-05	0.000685794
TC06001057.hg.1	STX11	8676	syntaxin 11	-2.271072	5.46686E-05	0.000500685
TC19000065.hg.1	GNA15	2769	guanine nucleotide binding protein (G protein), alpha 15 (Gq class)	-2.276302	7.54757E-05	0.000645591
TC03002133.hg.1	ATP13A3	79572	ATPase type 13A3	-2.278783	0.00022945	0.001587847
TC17002849.hg.1	PER1	5187	period circadian clock 1	-2.282078	0.001216603	0.006216292
TC02002279.hg.1	MAP3K2	10746	mitogen-activated protein kinase kinase kinase 2	-2.284985	0.00014932	0.001126466
TC11002002.hg.1	CHKA	1119	choline kinase alpha	-2.290305	0.00018236	0.001326023
TC20000876.hg.1	SLPI	6590	secretory leukocyte peptidase inhibitor	-2.290401	0.000343732	0.002208677
TC18000231.hg.1	SERPINB10	5273	serpin peptidase inhibitor, clade B (ovalbumin), member 10	-2.296473	6.41221E-05	0.000567814
TC01003367.hg.1	FCRL1	115350	Fc receptor-like 1	-2.29776	0.003421727	0.0141479
TC21000516.hg.1	ICOSLG	23308	inducible T-cell co-stimulator ligand	-2.301956	1.07532E-05	0.000139365
TC02000365.hg.1	LOC100507006	100507006	uncharacterized LOC100507006	-2.302264	2.13092E-08	1.43142E-06
TC11002226.hg.1	TMEM123	114908	transmembrane protein 123	-2.304611	4.71478E-09	5.48748E-07
TC20000913.hg.1	SULF2	55959	sulfatase 2	-2.304973	0.003665012	0.01494945
TC17000330.hg.1	TRAF4	9618	TNF receptor-associated factor 4	-2.309486	1.97856E-06	3.79859E-05
TC20000599.hg.1	GPCPD1	56261	glycerophosphocholine phosphodiesterase GDE1 homolog (S. cerevisiae)	-2.309518	4.46001E-05	0.000425397
TC09000400.hg.1	CTSL1	1514	cathepsin L1	-2.311335	0.00010917	0.000872557
TC11001194.hg.1	APLP2	334	amyloid beta (A4) precursor-like protein 2	-2.311672	1.35923E-05	0.000167426
TC09000990.hg.1	C9orf72	203228	chromosome 9 open reading frame 72	-2.312837	0.000783412	0.00435552
TC01006308.hg.1	FCGR3B	2215	Fc fragment of IgG, low affinity IIIb, receptor (CD16b)	-2.317788	0.015127	0.04644328
TC06001027.hg.1	TNFAIP3	7128	tumor necrosis factor, alpha-induced protein 3	-2.322194	0.000658156	0.003773175
TC14000434.hg.1	LOC145474	145474	uncharacterized LOC145474	-2.323879	0.000145074	0.001102046
TC05001366.hg.1	IL6ST	3572	interleukin 6 signal transducer (gp130, oncostatin M receptor)	-2.32498	2.63849E-05	0.000281159
TC05001995.hg.1	EBF1	1879	early B-cell factor 1	-2.327735	1.50511E-05	0.000181581
TC19000134.hg.1	C19orf59	199675	chromosome 19 open reading frame 59	-2.328959	0.000883426	0.004809398
TC06000720.hg.1	OGFRL1	79627	opioid growth factor receptor-like 1	-2.330783	2.76559E-09	4.09334E-07
TC07000793.hg.1	TSPAN33	340348	tetraspanin 33	-2.332637	3.21422E-05	0.000328598
TC20000587.hg.1	RASSF2	9770	Ras association (RalGDS/AF-6) domain family member 2	-2.335109	0.000200781	0.001434325
TC19002611.hg.1	CD22	933	CD22 molecule	-2.336612	0.002062283	0.009440014
TC17000965.hg.1	FAM101B	359845	family with sequence similarity 101, member B	-2.336723	4.46099E-06	7.10437E-05
TC19000274.hg.1	CYP4F3	4051	cytochrome P450, family 4, subfamily F, polypeptide 3	-2.33813	0.000285478	0.00189743
TC16000098.hg.1	MMP25	64386	matrix metallopeptidase 25	-2.340313	4.42462E-06	7.06622E-05
TC02000116.hg.1	RHOB	388	ras homolog family member B	-2.341276	0.00064133	0.003693196
TC19000993.hg.1	R3HDM4	91300	R3H domain containing 4	-2.342088	1.84055E-05	0.000212183
TC16000570.hg.1	NFAT5	10725	nuclear factor of activated T-cells 5, tonicity-responsive	-2.344971	1.91944E-06	3.71486E-05
TC05000096.hg.1	BASP1	10409	brain abundant, membrane attached signal protein 1	-2.354287	2.92728E-07	9.26414E-06
TC04001501.hg.1	FLJ14186	401149	uncharacterized LOC401149	-2.35633	5.96033E-07	1.5203E-05
TC03001550.hg.1	PROK2	60675	prokineticin 2	-2.35967	0.000268948	0.001808139
TC17000983.hg.1	MIR22HG	84981	MIR22 host gene (non-protein coding)	-2.360166	0.000342519	0.002201451
TC20000206.hg.1	HCK	3055	hemopoietic cell kinase	-2.36108	0.000204841	0.001453586
TC08000190.hg.1	ADAM28	10863	ADAM metallopeptidase domain 28	-2.363162	1.84957E-05	0.000212762
TC04002943.hg.1	THAP9-AS1	100499177	THAP9 antisense RNA 1	-2.364186	1.34767E-05	0.000166365
TC19001242.hg.1	EMR2	30817	egf-like module containing, mucin-like, hormone receptor-like 2	-2.367944	5.64451E-05	0.000513359
TC22000270.hg.1	NCF4	4689	neutrophil cytosolic factor 4, 40kDa	-2.373687	0.00046397	0.002827714
TC03000015.hg.1	BHLHE40	8553	basic helix-loop-helix family, member e40	-2.378626	0.000220504	0.001538846
TC10000922.hg.1	PTPRE	5791	protein tyrosine phosphatase, receptor type, E	-2.381521	6.33491E-05	0.000561811
TC22000620.hg.1	MN1	4330	meningioma (disrupted in balanced translocation) 1	-2.387433	3.38676E-10	1.12224E-07
TC12000159.hg.1	GABARAPL1	23710	GABA(A) receptor-associated protein like 1	-2.398028	0.000144487	0.001098234
TC15000870.hg.1	FURIN	5045	furin (paired basic amino acid cleaving enzyme)	-2.400483	1.97606E-06	3.79369E-05
TC14001466.hg.1	SERPINA1	5265	serpin peptidase inhibitor, clade A (alpha-1 antiproteinase, antitrypsin), member 1	-2.404088	2.91078E-06	5.07573E-05
TC08001500.hg.1	KLF10	7071	Kruppel-like factor 10	-2.407051	2.09914E-07	7.3924E-06

transcript cluster id	Gene Symbol	Entrez Gene ID	Description	FoldChange (AIM/control)	P.Value (AIM/control)	adj.P.Val (AIM/control)
TC20000154.hg.1	GZF1	64412	GDNF-inducible zinc finger protein 1	-2.414186	0.000603172	0.003513261
TC03002102.hg.1	LPP-AS2	339929	LPP antisense RNA 2	-2.422368	1.05276E-07	4.47447E-06
TC01003784.hg.1	LAMB3	3914	laminin, beta 3	-2.424267	7.1109E-09	7.23564E-07
TC04001650.hg.1	TMEM154	201799	transmembrane protein 154	-2.429448	0.000219528	0.001532688
TC0X000952.hg.1	DYNLT3	6990	dynein, light chain, Tctex-type 3	-2.432177	1.54193E-07	5.88349E-06
TC06001946.hg.1	BACH2	60468	BTB and CNC homology 1, basic leucine zipper transcription factor 2	-2.435149	6.78817E-05	0.000592283
TC10000415.hg.1	SRGN	5552	serglycin	-2.436553	4.57687E-06	7.22687E-05
TC22000257.hg.1	TOM1	10043	target of myb1 (chicken)	-2.438699	1.15385E-05	0.00014699
TC08000403.hg.1	SDCBP	6386	syndecan binding protein (syntenin)	-2.440923	8.76532E-06	0.000119519
TC08000997.hg.1	LONRF1	91694	LON peptidase N-terminal domain and ring finger 1	-2.441312	0.000372257	0.002360088
TC12001807.hg.1	BTG1	694	B-cell translocation gene 1, anti-proliferative	-2.442612	4.19849E-08	2.33194E-06
TC11003501.hg.1	LOC100133161	100133161	uncharacterized LOC100133161	-2.443925	3.69602E-06	6.1193E-05
TC21000188.hg.1	MX2	4600	myxovirus (influenza virus) resistance 2 (mouse)	-2.457466	2.24551E-05	0.000247409
TC03000207.hg.1	EIF1B	10289	eukaryotic translation initiation factor 1B	-2.457597	1.07973E-08	9.1904E-07
TC04001355.hg.1	WDFY3	23001	WD repeat and FYVE domain containing 3	-2.458483	0.000554312	0.003280744
TC11001107.hg.1	SORL1	6653	sortilin-related receptor, L(DLR class) A repeats containing	-2.461094	3.60284E-05	0.000358132
TC01000057.hg.1	SKI	6497	v-ski sarcoma viral oncogene homolog (avian)	-2.482052	7.08001E-05	0.000612916
TC06001219.hg.1	MGC39372	221756	serpin peptidase inhibitor, clade B (ovalbumin), member 9 pseudogene	-2.485004	2.0666E-08	1.3948E-06
TC12001215.hg.1	CLEC7A	64581	C-type lectin domain family 7, member A	-2.487684	0.00100444	0.005314168
TC06000795.hg.1	PNRC1	10957	proline-rich nuclear receptor coactivator 1	-2.488434	5.96662E-09	6.29927E-07
TC04000632.hg.1	SPRY1	10252	sprouty homolog 1, antagonist of FGF signaling (Drosophila)	-2.488857	0.000444022	0.002723109
TC15000417.hg.1	MAPK6	5597	mitogen-activated protein kinase 6	-2.4902	4.3464E-05	0.000416035
TC01001685.hg.1	BTG2	7832	BTG family, member 2	-2.492606	1.26731E-06	2.68163E-05
TC11000544.hg.1	BEST1	7439	bestrophin 1	-2.517098	4.17428E-07	1.19289E-05
TC12001625.hg.1	DDIT3	1649	DNA-damage-inducible transcript 3	-2.521158	0.002128815	0.009684753
TC09001072.hg.1	PAX5	5079	paired box 5	-2.521254	0.000268719	0.001806424
TC02000668.hg.1	RANBP2	5903	RAN binding protein 2	-2.521785	7.34774E-07	1.77977E-05
TC22000416.hg.1	PIM3	415116	pim-3 oncogene	-2.523857	7.19066E-08	3.37462E-06
TC19001842.hg.1	LILRB2	10288	leukocyte immunoglobulin-like receptor, subfamily B (with TM and ITIM domains), memb	-2.523874	1.19262E-05	0.000150401
TC11000510.hg.1	MS4A1	931	membrane-spanning 4-domains, subfamily A, member 1	-2.52439	0.006048041	0.02243643
TC20000391.hg.1	SLC9A8	23315	solute carrier family 9, subfamily A (NHE8, cation proton antiporter 8), member 8	-2.52523	6.38221E-06	9.41313E-05
TC16001098.hg.1	N4BP1	9683	NEDD4 binding protein 1	-2.529309	1.00079E-05	0.000131614
TC15001571.hg.1	DENND4A	10260	DENN/MADD domain containing 4A	-2.529677	1.83226E-06	3.57928E-05
TC03003371.hg.1	FAM157A	728262	family with sequence similarity 157, member A	-2.534419	2.64594E-06	4.71993E-05
TC09000560.hg.1	UGCG	7357	UDP-glucose ceramide glucosyltransferase	-2.537266	1.72474E-07	6.38425E-06
TC02000672.hg.1	RGPD6	729540	RANBP2-like and GRIP domain containing 6	-2.537348	6.21294E-07	1.56631E-05
TC02001300.hg.1	SLC11A1	6556	solute carrier family 11 (proton-coupled divalent metal ion transporters), member 1	-2.540968	2.51643E-06	4.54221E-05
TC10001329.hg.1	JMJD1C	221037	jumonji domain containing 1C	-2.543047	1.33776E-07	5.31393E-06
TC13000506.hg.1	USP12	219333	ubiquitin specific peptidase 12	-2.546049	4.48057E-06	7.11162E-05
TC03000348.hg.1	PRKCD	5580	protein kinase C, delta	-2.557948	1.31169E-06	2.74956E-05
TC05001590.hg.1	ARRDC3	57561	arrestin domain containing 3	-2.568267	8.79344E-05	0.000726671
TC05000184.hg.1	PTGER4	5734	prostaglandin E receptor 4 (subtype EP4)	-2.570995	1.68501E-06	3.3694E-05
TC03001468.hg.1	DCP1A	55802	decapping mRNA 1A	-2.571146	4.26334E-07	1.2089E-05
TC10001641.hg.1	ITPRIP	85450	inositol 1,4,5-trisphosphate receptor interacting protein	-2.572917	2.22874E-06	4.14005E-05
TC05001628.hg.1	CHD1	1105	chromodomain helicase DNA binding protein 1	-2.573503	0.00011429	0.000904685
TC02002203.hg.1	RGPD5	84220	RANBP2-like and GRIP domain containing 5	-2.579128	4.67067E-07	1.28084E-05
TC15000793.hg.1	WHAMM	123720	WAS protein homolog associated with actin, golgi membranes and microtubules	-2.587846	1.04494E-05	0.000135754
TC08001400.hg.1	NBN	4683	nibrin	-2.588055	0.001966318	0.009089498
TC03000636.hg.1	DIRC2	84925	disrupted in renal carcinoma 2	-2.590107	8.15359E-06	0.000113214
TC02005045.hg.1	NFE2L2	4780	nuclear factor (erythroid-derived 2)-like 2	-2.591274	1.759E-06	3.476E-05
TC12000558.hg.1	MIRLET7I	406891	microRNA let-7i	-2.59208	4.19333E-09	5.15501E-07
TC0X000575.hg.1	IL13RA1	3597	interleukin 13 receptor, alpha 1	-2.605713	0.000763445	0.004266651
TC16000666.hg.1	IRF8	3394	interferon regulatory factor 8	-2.610374	6.65101E-09	6.7602E-07
TC02002214.hg.1	RGPD8	727851	RANBP2-like and GRIP domain containing 8	-2.611994	3.70839E-07	1.0845E-05
TC01000267.hg.1	LOC100506801	100506801	uncharacterized LOC100506801	-2.613035	2.54248E-09	3.79989E-07
TC20000527.hg.1	LOC100505815	100505815	uncharacterized LOC100505815	-2.62205	8.05301E-06	0.000112123
TC22001457.hg.1	ADORA2A	135	adenosine A2a receptor	-2.622759	3.65446E-07	1.07391E-05
TC20000924.hg.1	ZNFX1	57169	zinc finger, NFX1-type containing 1	-2.629141	4.50705E-06	7.14244E-05
TC07001318.hg.1	INHBA	3624	inhibin, beta A	-2.630129	0.000195738	0.001403408
TC01003732.hg.1	PPP1R15B	84919	protein phosphatase 1, regulatory subunit 15B	-2.639296	3.00834E-05	0.000310458
TC14000417.hg.1	KIAA0247	9766	KIAA0247	-2.640974	2.28935E-07	7.79297E-06
TC06001715.hg.1	TREML3P	340206	triggering receptor expressed on myeloid cells-like 3, pseudogene	-2.641734	0.001252452	0.006354051
TC08000383.hg.1	LYN	4067	v-yes-1 Yamaguchi sarcoma viral related oncogene homolog	-2.642124	3.1373E-05	0.000321393
TC01002526.hg.1	MTF1	4520	metal-regulatory transcription factor 1	-2.651855	9.88501E-06	0.0001303
TC02002767.hg.1	CXCR1	3577	chemokine (C-X-C motif) receptor 1	-2.654926	0.01645988	0.04960618
TC07003301.hg.1	PNPLA8	50640	patatin-like phospholipase domain containing 8	-2.661707	4.14155E-06	6.6646E-05
TC13000147.hg.1	RGCC	28984	regulator of cell cycle	-2.669088	5.72927E-08	2.88557E-06
TC11001260.hg.1	DUSP8	1850	dual specificity phosphatase 8	-2.673562	6.75444E-07	1.67035E-05
TC16000375.hg.1	ITGAX	3687	integrin, alpha X (complement component 3 receptor 4 subunit)	-2.694455	6.45201E-06	9.47647E-05
TC17000728.hg.1	MIR21	406991	microRNA 21	-2.705496	0.001095262	0.005712871
TC16000293.hg.1	IL4R	3566	interleukin 4 receptor	-2.711175	7.39932E-09	7.34197E-07
TC11001860.hg.1	FTH1	2495	ferritin, heavy polypeptide 1	-2.713964	5.37686E-08	2.75173E-06

transcript cluster id	Gene Symbol	Entrez Gene ID	Description	FoldChange (AIM/control)	P.Value (AIM/control)	adj.P.Val (AIM/control)
TC01000733.hg.1	PDE4B	5142	phosphodiesterase 4B, cAMP-specific	-2.714886	2.98499E-07	9.34646E-06
TC21000480.hg.1	ZBTB21	49854	zinc finger and BTB domain containing 21	-2.728576	1.88339E-05	0.0002149
TC21000198.hg.1	ABCG1	9619	ATP-binding cassette, sub-family G (WHITE), member 1	-2.72891	6.89897E-07	1.69388E-05
TC20000401.hg.1	LOC100506115	100506115	uncharacterized LOC100506115	-2.734367	7.27798E-06	0.000103593
TC01000352.hg.1	ZDHHC18	84243	zinc finger, DHHC-type containing 18	-2.736971	0.000257229	0.001742716
TC08000793.hg.1	DENND3	22898	DENN/MADD domain containing 3	-2.745848	0.000100334	0.000810861
TC0X001544.hg.1	MPP1	4354	membrane protein, palmitoylated 1, 55kDa	-2.756428	9.25473E-09	8.26548E-07
TC05003443.hg.1	LOC100132287	100132287	uncharacterized LOC100132287	-2.765605	2.65523E-06	4.72043E-05
TC19002719.hg.1	LILRA5	353514	leukocyte immunoglobulin-like receptor, subfamily A (with TM domain), member 5	-2.77123	0.000381046	0.002401401
TC17001820.hg.1	SLC16A6	9120	solute carrier family 16, member 6 (monocarboxylic acid transporter 7)	-2.774334	0.002492932	0.01097707
TC11000499.hg.1	STX3	6809	syntaxin 3	-2.778103	0.000132659	0.001023543
TC03002096.hg.1	BCL6	604	B-cell CLL/lymphoma 6	-2.779894	0.000192946	0.001386103
TC10000413.hg.1	DDX21	9188	DEAD (Asp-Glu-Ala-Asp) box helicase 21	-2.781568	1.22336E-06	2.61629E-05
TC02002378.hg.1	CXCR4	7852	chemokine (C-X-C motif) receptor 4	-2.782134	0.000620946	0.003593579
TC15002764.hg.1	PLEKHO2	80301	pleckstrin homology domain containing, family O member 2	-2.79655	0.000126743	0.000985611
TC02000619.hg.1	IL1R2	7850	interleukin 1 receptor, type II	-2.797759	7.47169E-06	0.000105745
TC01003616.hg.1	NCF2	4688	neutrophil cytosolic factor 2	-2.804824	2.59077E-05	0.000276304
TC02001202.hg.1	ICOS	29851	inducible T-cell co-stimulator	-2.806862	0.000154693	0.001158452
TC10000360.hg.1	UBE2D1	7321	ubiquitin-conjugating enzyme E2D 1	-2.819967	8.92539E-06	0.000120945
TC06001279.hg.1	TBC1D7	51256	TBC1 domain family, member 7	-2.82295	3.54545E-08	2.04364E-06
TC06002152.hg.1	IFNGR1	3459	interferon gamma receptor 1	-2.826395	6.92647E-05	0.000600904
TC21000097.hg.1	GRIK1-AS2	100379661	GRIK1 antisense RNA 2	-2.826924	7.37031E-06	0.000104632
TC0X000925.hg.1	KLHL15	80311	kelch-like family member 15	-2.837922	5.89589E-07	1.50343E-05
TC16000165.hg.1	SNN	8303	stannin	-2.838594	9.1924E-06	0.000123553
TC18000554.hg.1	BCL2	596	B-cell CLL/lymphoma 2	-2.84051	1.74807E-05	0.000203103
TC17000297.hg.1	MAP2K3	5606	mitogen-activated protein kinase kinase 3	-2.840782	9.85418E-10	2.01408E-07
TC08000188.hg.1	SLC25A37	51312	solute carrier family 25 (mitochondrial iron transporter), member 37	-2.842598	0.001065436	0.005583451
TC22000271.hg.1	CSF2RB	1439	colony stimulating factor 2 receptor, beta, low-affinity (granulocyte-macrophage)	-2.845893	7.82537E-05	0.000663612
TC01003718.hg.1	CHI3L1	1116	chitinase 3-like 1 (cartilage glycoprotein-39)	-2.848867	1.01509E-08	8.73695E-07
TC03000951.hg.1	PIK3CA	5290	phosphatidylinositol-4,5-bisphosphate 3-kinase, catalytic subunit alpha	-2.851462	3.85477E-07	1.11518E-05
TC01000505.hg.1	RLF	6018	rearranged L-myc fusion	-2.851488	7.04266E-05	0.000609379
TC03000814.hg.1	TSC22D2	9819	TSC22 domain family, member 2	-2.851758	1.11578E-08	9.33519E-07
TC16000480.hg.1	HERPUD1	9709	homocysteine-inducible, endoplasmic reticulum stress-inducible, ubiquitin-like domain m	-2.864335	2.15839E-05	0.000240005
TC21000506.hg.1	SIK1	150094	salt-inducible kinase 1	-2.868845	0.001150091	0.005930589
TC03000846.hg.1	MME	4311	membrane metallo-endopeptidase	-2.870904	0.001818141	0.008566226
TC19000627.hg.1	BCL3	602	B-cell CLL/lymphoma 3	-2.872168	7.57426E-06	0.000106931
TC18000213.hg.1	PMAIP1	5366	phorbol-12-myristate-13-acetate-induced protein 1	-2.872964	0.000990369	0.005256502
TC05001411.hg.1	SGTB	54557	small glutamine-rich tetratricopeptide repeat (TPR)-containing, beta	-2.881613	0.000104146	0.000836323
TC01001736.hg.1	CD55	1604	CD55 molecule, decay accelerating factor for complement (Cromer blood group)	-2.893054	1.65017E-08	1.19371E-06
TC03001355.hg.1	CCR1	1230	chemokine (C-C motif) receptor 1	-2.895023	0.001875074	0.008769316
TC06000086.hg.1	HIVEP1	3096	human immunodeficiency virus type I enhancer binding protein 1	-2.896408	2.02383E-06	3.84791E-05
TC05002050.hg.1	LCP2	3937	lymphocyte cytosolic protein 2 (SH2 domain containing leukocyte protein of 76kDa)	-2.902236	4.38852E-09	5.26348E-07
TC10000219.hg.1	ZEB1	6935	zinc finger E-box binding homeobox 1	-2.903051	3.07794E-06	5.26692E-05
TC17001661.hg.1	PHOSPHO1	162466	phosphatase, orphan 1	-2.903712	1.20542E-08	9.86787E-07
TC0X001020.hg.1	PIM2	11040	pim-2 oncogene	-2.904302	1.94397E-08	1.33703E-06
TC01002054.hg.1	LOC100996554	100996554	uncharacterized LOC100996554	-2.907418	3.37364E-06	5.65748E-05
TC02000583.hg.1	ARID5A	10865	AT rich interactive domain 5A (MRF1-like)	-2.910136	6.02181E-05	0.000540403
TC02005020.hg.1	CMPK2	129607	cytidine monophosphate (UMP-CMP) kinase 2, mitochondrial	-2.916369	0.001590746	0.007694072
TC17000118.hg.1	TMEM88	92162	transmembrane protein 88	-2.916504	1.5942E-06	3.20715E-05
TC09001325.hg.1	NFIL3	4783	nuclear factor, interleukin 3 regulated	-2.924848	0.001746245	0.008286346
TC11000374.hg.1	CD82	3732	CD82 molecule	-2.925406	8.77752E-05	0.000724627
TC21000189.hg.1	MX1	4599	myxovirus (influenza virus) resistance 1, interferon-inducible protein p78 (mouse)	-2.928006	0.000250042	0.001702186
TC05001189.hg.1	FTH1P3	2498	ferritin, heavy polypeptide 1 pseudogene 3	-2.93523	6.47901E-07	1.61732E-05
TC06000868.hg.1	FOXO3	2309	forkhead box O3	-2.939407	3.50055E-07	1.04861E-05
TC15001441.hg.1	DMXL2	23312	Dmx-like 2	-2.948842	4.65726E-05	0.000438799
TC0X000082.hg.1	REPS2	9185	RALBP1 associated Eps domain containing 2	-2.950099	2.72297E-05	0.000287529
TC08001044.hg.1	EGR3	1960	early growth response 3	-2.961107	5.03071E-07	1.34914E-05
TC01001738.hg.1	CR1	1378	complement component (3b/4b) receptor 1 (Knops blood group)	-2.976135	4.84659E-05	0.000452001
TC02002705.hg.1	KLF7	8609	Kruppel-like factor 7 (ubiquitous)	-2.98455	2.87646E-07	9.07891E-06
TC08000749.hg.1	MYC	4609	v-myc myelocytomatosis viral oncogene homolog (avian)	-2.997269	1.03822E-05	0.000134864
TC01001569.hg.1	IER5	51278	immediate early response 5	-2.997642	3.04253E-08	1.81649E-06
TC09000857.hg.1	FAM157B	100132403	family with sequence similarity 157, member B	-3.001853	6.81251E-07	1.67388E-05
TC19001102.hg.1	TNFSF14	8740	tumor necrosis factor (ligand) superfamily, member 14	-3.014604	2.30626E-06	4.23613E-05
TC08001320.hg.1	MSC	9242	musculin	-3.01623	4.94044E-07	1.33214E-05
TC01000005.hg.1	LOC100132287	100132287	uncharacterized LOC100132287	-3.018357	1.54508E-06	3.12891E-05
TC20000878.hg.1	SDC4	6385	syndecan 4	-3.03431	1.51746E-07	5.78554E-06
TC04002952.hg.1	AREG	374	amphiregulin	-3.03438	8.49943E-05	0.000707771
TC0X000141.hg.1	GK	2710	glycerol kinase	-3.046519	0.000356028	0.002271163
TC07001869.hg.1	MIR29A	407021	microRNA 29a	-3.051348	3.74554E-06	6.15607E-05
TC06001220.hg.1	SERPINB9	5272	serpin peptidase inhibitor, clade B (ovalbumin), member 9	-3.054942	8.10535E-07	1.91853E-05
TC19001285.hg.1	JUND	3727	jun D proto-oncogene	-3.055126	8.91236E-07	2.05372E-05
TC11002153.hg.1	RAB30	27314	RAB30, member RAS oncogene family	-3.05741	1.37521E-07	5.36949E-06

transcript cluster id	Gene Symbol	Entrez Gene ID	Description	FoldChange (AIM/control)	P.Value (AIM/control)	adj.P.Val (AIM/control)
TC0X000939.hg.1	CXorf21	80231	chromosome X open reading frame 21	-3.06428	2.02368E-05	0.000227259
TC12000591.hg.1	IRAK3	11213	interleukin-1 receptor-associated kinase 3	-3.075086	7.86583E-05	0.000665411
TC20000363.hg.1	MMP9	4318	matrix metallopeptidase 9 (gelatinase B, 92kDa gelatinase, 92kDa type IV collagenase)	-3.078203	0.000437445	0.002690768
TC12002059.hg.1	OASL	8638	2'-5'-oligoadenylate synthetase-like	-3.090964	0.001801412	0.008499334
TC02000348.hg.1	REL	5966	v-rel reticuloendotheliosis viral oncogene homolog (avian)	-3.092328	1.25551E-06	2.65852E-05
TC10000979.hg.1	KLF6	1316	Kruppel-like factor 6	-3.097283	1.4378E-08	1.08864E-06
TC04002953.hg.1	AREG	374	amphiregulin	-3.101322	0.000102032	0.000822123
TC17000496.hg.1	RARA	5914	retinoic acid receptor, alpha	-3.148952	1.12645E-05	0.000143633
TC06002121.hg.1	VNN2	8875	vanin 2	-3.16228	0.003063333	0.01291853
TC07000112.hg.1	AHR	196	aryl hydrocarbon receptor	-3.165438	9.35017E-06	0.00012516
TC15001606.hg.1	TLE3	7090	transducin-like enhancer of split 3 (E(sp1) homolog, Drosophila)	-3.181271	5.42535E-05	0.000495944
TC02002074.hg.1	EIF2AK3	9451	eukaryotic translation initiation factor 2-alpha kinase 3	-3.181712	1.77054E-06	3.47948E-05
TC12001626.hg.1	MIR616	693201	microRNA 616	-3.19919	9.39545E-05	0.000767044
TC09001541.hg.1	TRAF1	7185	TNF receptor-associated factor 1	-3.20345	4.56458E-09	5.3365E-07
TC12000627.hg.1	RAB21	23011	RAB21, member RAS oncogene family	-3.205505	5.002E-06	7.70362E-05
TC13000100.hg.1	ALOX5AP	241	arachidonate 5-lipoxygenase-activating protein	-3.216912	2.88781E-05	0.000300784
TC21000167.hg.1	KCNJ15	3772	potassium inwardly-rectifying channel, subfamily J, member 15	-3.219322	0.000893501	0.004844061
TC07003328.hg.1	RABGEF1	27342	RAB guanine nucleotide exchange factor (GEF) 1	-3.224357	2.17868E-07	7.51044E-06
TC01002072.hg.1	LOC100288069	100288069	general transcription factor IIi pseudogene	-3.235488	3.69021E-07	1.0752E-05
TC12001751.hg.1	PHLDA1	22822	pleckstrin homology-like domain, family A, member 1	-3.239932	7.65499E-08	3.49146E-06
TC20000928.hg.1	B4GALT5	9334	UDP-Gal:betaGlcNAc beta 1,4- galactosyltransferase, polypeptide 5	-3.252889	9.49935E-05	0.000774197
TC04000485.hg.1	HERC5	51191	HECT and RLD domain containing E3 ubiquitin protein ligase 5	-3.260048	0.00816304	0.02836978
TC21000288.hg.1	NRIP1	8204	nuclear receptor interacting protein 1	-3.263994	9.15395E-09	8.20169E-07
TC10000211.hg.1	MAP3K8	1326	mitogen-activated protein kinase kinase kinase 8	-3.273215	0.000237699	0.00163331
TC08001501.hg.1	AZIN1	51582	antizyme inhibitor 1	-3.281215	6.67795E-07	1.65198E-05
TC18000114.hg.1	TAF4B	6875	TAF4b RNA polymerase II, TATA box binding protein (TBP)-associated factor, 105kDa	-3.284169	1.07365E-05	0.000138388
TC05001946.hg.1	TNIP1	10318	TNFAIP3 interacting protein 1	-3.28508	1.0285E-06	2.27974E-05
TC12001207.hg.1	CD69	969	CD69 molecule	-3.287471	3.73979E-06	6.14441E-05
TC01002056.hg.1	LOC100996554	100996554	uncharacterized LOC100996554	-3.303278	5.60015E-07	1.45518E-05
TC0X000875.hg.1	PIGA	5277	phosphatidylinositol glycan anchor biosynthesis, class A	-3.309795	9.15539E-05	0.000750551
TC14001029.hg.1	CFL2	1073	cofilin 2 (muscle)	-3.323016	1.59467E-07	5.98823E-06
TC11000628.hg.1	MIR612	693197	microRNA 612	-3.333436	2.92179E-06	5.06656E-05
TC11001915.hg.1	EHD1	10938	EH-domain containing 1	-3.35244	7.66743E-10	1.66382E-07
TC19000588.hg.1	CD79A	973	CD79a molecule, immunoglobulin-associated alpha	-3.357072	0.000514883	0.00307471
TC12001027.hg.1	GLT1D1	144423	glycosyltransferase 1 domain containing 1	-3.383688	0.000357655	0.002277638
TC11000175.hg.1	SWAP70	23075	SWAP switching B-cell complex 70kDa subunit	-3.402378	9.55704E-06	0.000126608
TC07003314.hg.1	PILRA	29992	paired immunoglobin-like type 2 receptor alpha	-3.422119	2.8429E-07	8.9846E-06
TC17000977.hg.1	SLC43A2	124935	solute carrier family 43, member 2	-3.422447	3.56522E-07	1.05873E-05
TC10001007.hg.1	IL2RA	3559	interleukin 2 receptor, alpha	-3.428407	4.25548E-07	1.20099E-05
TC11001847.hg.1	SLC15A3	51296	solute carrier family 15, member 3	-3.42851	3.16614E-08	1.86286E-06
TC12000130.hg.1	CLEC4D	338339	C-type lectin domain family 4, member D	-3.430273	0.000287518	0.001903952
TC04000809.hg.1	RAPGEF2	9693	Rap guanine nucleotide exchange factor (GEF) 2	-3.438939	8.58299E-07	1.99765E-05
TC02000620.hg.1	IL1R1	3554	interleukin 1 receptor, type I	-3.455653	4.35093E-08	2.3496E-06
TC15001257.hg.1	EHD4	30844	EH-domain containing 4	-3.484307	9.29647E-07	2.1131E-05
TC20000019.hg.1	SIRPA	140885	signal-regulatory protein alpha	-3.492459	1.80786E-08	1.26052E-06
TC01001624.hg.1	RGS2	5997	regulator of G-protein signaling 2, 24kDa	-3.52914	9.12034E-06	0.000122672
TC11000956.hg.1	BIRC3	330	baculoviral IAP repeat containing 3	-3.53082	2.52198E-08	1.57586E-06
TC08002590.hg.1	DOCK5	80005	dedicator of cytokinesis 5	-3.549281	5.04895E-06	7.76052E-05
TC17000349.hg.1	CPD	1362	carboxypeptidase D	-3.549974	7.25123E-06	0.000103042
TC03001554.hg.1	RYBP	23429	RING1 and YY1 binding protein	-3.55388	2.40903E-05	0.000260794
TC01000795.hg.1	IFI44	10561	interferon-induced protein 44	-3.556998	0.001548596	0.007539153
TC05002066.hg.1	DUSP1	1843	dual specificity phosphatase 1	-3.641022	5.10778E-06	7.81048E-05
TC01003985.hg.1	IRF2BP2	359948	interferon regulatory factor 2 binding protein 2	-3.688042	2.22444E-09	3.53427E-07
TC09000660.hg.1	ZBTB43	23099	zinc finger and BTB domain containing 43	-3.706378	8.20776E-06	0.000113319
TC06000608.hg.1	VEGFA	7422	vascular endothelial growth factor A	-3.710606	2.04083E-06	3.85568E-05
TC02000183.hg.1	FOSL2	2355	FOS-like antigen 2	-3.716707	2.2655E-06	4.1696E-05
TC15000691.hg.1	C15orf39	56905	chromosome 15 open reading frame 39	-3.726122	1.12757E-06	2.4519E-05
TC07003291.hg.1	IFRD1	3475	interferon-related developmental regulator 1	-3.802311	1.26847E-06	2.67253E-05
TC01000261.hg.1	CDA	978	cytidine deaminase	-3.802443	1.17681E-06	0.000025295
TC10000140.hg.1	ARL5B	221079	ADP-ribosylation factor-like 5B	-3.816469	0.000206227	0.001457302
TC01002053.hg.1	LOC729737	729737	uncharacterized LOC729737	-3.824276	1.31887E-06	2.74508E-05
TC11000182.hg.1	ADM	133	adrenomedullin	-3.827273	1.26906E-05	0.000157761
TC19000544.hg.1	ZFP36	7538	ZFP36 ring finger protein	-3.836884	3.09683E-08	1.82631E-06
TC02000398.hg.1	PLEK	5341	pleckstrin	-3.846026	7.55643E-05	0.000643687
TC12003253.hg.1	THAP2	83591	THAP domain containing, apoptosis associated protein 2	-3.853355	4.20259E-08	2.30783E-06
TC13000829.hg.1	GPR183	1880	G protein-coupled receptor 183	-3.863647	4.0316E-09	4.95585E-07
TC10000753.hg.1	NFKB2	4791	nuclear factor of kappa light polypeptide gene enhancer in B-cells 2 (p49/p100)	-3.88699	1.00591E-07	4.27818E-06
TC08000545.hg.1	RIPK2	8767	receptor-interacting serine-threonine kinase 2	-3.911007	0.000178055	0.001295725
TC01000314.hg.1	CLIC4	25932	chloride intracellular channel 4	-3.933209	7.93437E-07	1.87855E-05
TC09002906.hg.1	ORM2	5005	orosomucoid 2	-3.954572	2.0176E-06	0.000038222
TC21000129.hg.1	IFNGR2	3460	interferon gamma receptor 2 (interferon gamma transducer 1)	-4.015784	7.64617E-07	1.82356E-05
TC17000166.hg.1	MGC12916	84815	uncharacterized protein MGC12916	-4.016591	3.26208E-07	9.83311E-06

transcript cluster id	Gene Symbol	Entrez Gene ID	Description	FoldChange (AIM/control)	P.Value (AIM/control)	adj.P.Val (AIM/control)
TC14000914.hg.1	LINC00641	283624	long intergenic non-protein coding RNA 641	-4.029544	2.32702E-05	0.000253035
TC22001424.hg.1	APOBEC3A	200315	apolipoprotein B mRNA editing enzyme, catalytic polypeptide-like 3A	-4.071852	0.000222282	0.001544203
TC17001860.hg.1	CD300E	342510	CD300e molecule	-4.092091	1.41159E-08	1.07713E-06
TC0X000985.hg.1	MIR221	407006	microRNA 221	-4.106877	0.000170223	0.001249094
TC11000812.hg.1	DGAT2	84649	diacylglycerol O-acyltransferase 2	-4.197863	6.10086E-05	0.000544382
TC07001189.hg.1	LOC541472	541472	uncharacterized LOC541472	-4.202705	3.3378E-05	0.000337238
TC16000384.hg.1	ZNF267	10308	zinc finger protein 267	-4.214291	7.42812E-08	3.41521E-06
TC05001610.hg.1	ELL2	22936	elongation factor, RNA polymerase II, 2	-4.220864	2.87228E-05	0.000299202
TC02002481.hg.1	IFIH1	64135	interferon induced with helicase C domain 1	-4.228145	0.000146317	0.001105332
TC09000601.hg.1	TLR4	7099	toll-like receptor 4	-4.257653	6.50687E-06	9.4925E-05
TC01000474.hg.1	ZC3H12A	80149	zinc finger CCCH-type containing 12A	-4.261388	1.95906E-07	6.93873E-06
TC19000976.hg.1	FLJ45445	399844	uncharacterized LOC399844	-4.265047	8.40713E-07	1.95782E-05
TC06002120.hg.1	VNN3	55350	vanin 3	-4.286237	0.000915881	0.004945509
TC03000978.hg.1	B3GNT5	84002	UDP-GlcNAc:betaGal beta-1,3-N-acetylglucosaminyltransferase 5	-4.35126	1.48639E-06	3.01855E-05
TC12002089.hg.1	RILPL2	196383	Rab interacting lysosomal protein-like 2	-4.361623	8.18487E-07	1.92149E-05
TC03000917.hg.1	SKIL	6498	SKI-like oncogene	-4.387406	3.40502E-06	0.000056696
TC10001640.hg.1	MIR4482-1	100616323	microRNA 4482-1	-4.437452	2.7156E-08	1.66774E-06
TC02002445.hg.1	NR4A2	4929	nuclear receptor subfamily 4, group A, member 2	-4.437637	0.001172212	0.006020588
TC22000224.hg.1	LIMK2	3985	LIM domain kinase 2	-4.472971	5.60791E-05	0.000508716
TC06004142.hg.1	LOC100129518	100129518	uncharacterized LOC100129518	-4.487395	3.07991E-07	9.50675E-06
TC06000087.hg.1	EDN1	1906	endothelin 1	-4.497495	5.89734E-06	8.76421E-05
TC14001253.hg.1	ZFP36L1	677	ZFP36 ring finger protein-like 1	-4.508992	3.34295E-06	5.59496E-05
TC17001917.hg.1	SOCS3	9021	suppressor of cytokine signaling 3	-4.514006	8.90183E-10	1.85993E-07
TC05000934.hg.1	LOC257358	257358	uncharacterized LOC257358	-4.541608	4.04519E-09	4.95585E-07
TC15001837.hg.1	ANPEP	290	alanyl (membrane) aminopeptidase	-4.586711	7.88359E-05	0.000665411
TC13000644.hg.1	KIAA0226L	80183	KIAA0226-like	-4.587183	5.7069E-07	1.46816E-05
TC10000566.hg.1	PPIF	10105	peptidylprolyl isomerase F	-4.643631	4.94518E-06	7.62244E-05
TC07000459.hg.1	NCF1	653361	neutrophil cytosolic factor 1	-4.64553	1.71757E-06	3.39394E-05
TC06002126.hg.1	SGK1	6446	serum/glucocorticoid regulated kinase 1	-4.682725	3.91567E-07	1.12173E-05
TC12000413.hg.1	GRASP	160622	GRP1 (general receptor for phosphoinositides 1)-associated scaffold protein	-4.714844	4.72608E-10	1.32746E-07
TC01002708.hg.1	JUN	3725	jun proto-oncogene	-4.716593	4.09304E-06	6.56065E-05
TC04000411.hg.1	CXCL1	2919	chemokine (C-X-C motif) ligand 1 (melanoma growth stimulating activity, alpha)	-4.76509	3.97186E-09	4.94506E-07
TC03001302.hg.1	CSRNP1	64651	cysteine-serine-rich nuclear protein 1	-4.766516	4.31753E-07	1.2089E-05
TC11000333.hg.1	CD44	960	CD44 molecule (Indian blood group)	-4.776796	8.56844E-05	0.000709746
TC12000414.hg.1	NR4A1	3164	nuclear receptor subfamily 4, group A, member 1	-4.801777	3.98686E-08	2.19782E-06
TC06001762.hg.1	NFKBIE	4794	nuclear factor of kappa light polypeptide gene enhancer in B-cells inhibitor, epsilon	-4.811289	2.16045E-07	7.45609E-06
TC17000384.hg.1	CCL7	6354	chemokine (C-C motif) ligand 7	-4.813156	4.29785E-05	0.000410363
TC12001798.hg.1	ATP2B1	490	ATPase, Ca++ transporting, plasma membrane 1	-4.858323	1.71443E-07	6.32562E-06
TC07000899.hg.1	MGAM	8972	maltase-glucoamylase (alpha-glucosidase)	-4.943176	7.4845E-05	0.000638276
TC11002136.hg.1	GAB2	9846	GRB2-associated binding protein 2	-4.959432	2.45016E-06	4.41178E-05
TC21000408.hg.1	IL10RB-AS1	100288432	IL10RB antisense RNA 1 (head to head)	-5.051873	2.99983E-08	1.7789E-06
TC09002907.hg.1	ORM1	5004	orosomucoid 1	-5.070853	3.41722E-07	1.01924E-05
TC02001746.hg.1	CDC42EP3	10602	CDC42 effector protein (Rho GTPase binding) 3	-5.081925	3.24895E-08	1.88516E-06
TC01000272.hg.1	ALPL	249	alkaline phosphatase, liver/bone/kidney	-5.169585	0.000118039	0.00092714
TC01002322.hg.1	ECE1	1889	endothelin converting enzyme 1	-5.182949	5.00558E-09	5.55855E-07
TC19001241.hg.1	EMR3	84658	egf-like module containing, mucin-like, hormone receptor-like 3	-5.194254	2.91191E-06	5.05324E-05
TC04000775.hg.1	TLR2	7097	toll-like receptor 2	-5.222413	5.82638E-06	8.67285E-05
TC17001466.hg.1	CCR7	1236	chemokine (C-C motif) receptor 7	-5.233091	9.77145E-05	0.000791118
TC08000512.hg.1	ZBTB10	65986	zinc finger and BTB domain containing 10	-5.278672	9.09232E-08	3.99965E-06
TC14002197.hg.1	HIF1A	3091	hypoxia inducible factor 1, alpha subunit (basic helix-loop-helix transcription factor)	-5.306878	9.33928E-08	4.06501E-06
TC14000794.hg.1	TNFAIP2	7127	tumor necrosis factor, alpha-induced protein 2	-5.320456	7.19015E-08	3.34488E-06
TC13000871.hg.1	IRS2	8660	insulin receptor substrate 2	-5.324964	7.06208E-08	3.31088E-06
TC19001787.hg.1	FPR1	2357	formyl peptide receptor 1	-5.335845	4.4716E-06	7.06622E-05
TC07001516.hg.1	NCF1C	654817	neutrophil cytosolic factor 1C pseudogene	-5.343457	1.10535E-06	2.40398E-05
TC07000438.hg.1	NCF1B	654816	neutrophil cytosolic factor 1B pseudogene	-5.384777	1.74751E-06	3.43174E-05
TC04000414.hg.1	EREG	2069	epiregulin	-5.434127	6.29991E-06	9.23538E-05
TC04000526.hg.1	NFKB1	4790	nuclear factor of kappa light polypeptide gene enhancer in B-cells 1	-5.443395	5.47111E-08	2.75173E-06
TC10000640.hg.1	IFIT5	24138	interferon-induced protein with tetratricopeptide repeats 5	-5.459044	9.26764E-07	2.10367E-05
TC11001397.hg.1	DENND5A	23258	DENN/MADD domain containing 5A	-5.487104	9.99875E-10	2.01189E-07
TC02001364.hg.1	CCL20	6364	chemokine (C-C motif) ligand 20	-5.525104	6.60146E-06	9.59154E-05
TC02001292.hg.1	CXCR2	3579	chemokine (C-X-C motif) receptor 2	-5.534015	5.99906E-05	0.000537736
TC19000647.hg.1	FOSB	2354	FBJ murine osteosarcoma viral oncogene homolog B	-5.62829	5.12495E-06	7.81048E-05
TC15001719.hg.1	BCL2A1	597	BCL2-related protein A1	-5.671748	9.07199E-08	3.99551E-06
TC01003605.hg.1	GLUL	2752	glutamate-ammonia ligase	-5.687212	6.56975E-06	9.55102E-05
TC19000788.hg.1	FPR2	2358	formyl peptide receptor 2	-5.71805	8.42388E-05	0.000701056
TC01003629.hg.1	IVNS1ABP	10625	influenza virus NS1A binding protein	-5.782921	8.1091E-07	1.90765E-05
TC06002270.hg.1	TAGAP	117289	T-cell activation RhoGTPase activating protein	-5.853597	2.88999E-09	4.09334E-07
TC09001434.hg.1	ABCA1	19	ATP-binding cassette, sub-family A (ABC1), member 1	-5.904807	1.12458E-05	0.000143074
TC21000076.hg.1	MIR155	406947	microRNA 155	-5.905237	6.38452E-06	9.34294E-05
TC21001069.hg.1	SAMSN1	64092	SAM domain, SH3 domain and nuclear localization signals 1	-5.906267	0.000272348	0.001820365
TC03000257.hg.1	CCRL2	9034	chemokine (C-C motif) receptor-like 2	-5.982672	6.59219E-09	6.63579E-07
TC08000733.hg.1	TRIB1	10221	tribbles homolog 1 (Drosophila)	-6.065465	5.13667E-07	1.35899E-05

transcript cluster id	Gene Symbol	Entrez Gene ID	Description	FoldChange (AIM/control)	P.Value (AIM/control)	adj.P.Val (AIM/control)
TC12000698.hg.1	LINC00936	338758	long intergenic non-protein coding RNA 936	-6.301703	1.38056E-10	7.15239E-08
TC01000566.hg.1	PLK3	1263	polo-like kinase 3	-6.403756	1.36602E-08	1.05231E-06
TC16001335.hg.1	SLC7A5	8140	solute carrier family 7 (amino acid transporter light chain, L system), member 5	-6.426448	1.95801E-06	3.72671E-05
TC17001029.hg.1	CXCL16	58191	chemokine (C-X-C motif) ligand 16	-6.439565	1.10156E-06	2.39799E-05
TC15001643.hg.1	SEMA7A	8482	semaphorin 7A, GPI membrane anchor (John Milton Hagen blood group)	-6.459616	1.07726E-08	8.93452E-07
TC17000813.hg.1	KCNJ2	3759	potassium inwardly-rectifying channel, subfamily J, member 2	-6.466612	4.33676E-05	0.000413363
TC15000357.hg.1	MIR147B	100126311	microRNA 147b	-6.51673	1.3589E-08	1.04999E-06
TC10000629.hg.1	LIPN	643418	lipase, family member N	-6.521874	1.99669E-12	9.67172E-09
TC14001036.hg.1	NFKBIA	4792	nuclear factor of kappa light polypeptide gene enhancer in B-cells inhibitor, alpha	-6.63895	4.93217E-07	1.32268E-05
TC10001421.hg.1	C10orf55	414236	chromosome 10 open reading frame 55	-6.683992	6.31877E-09	6.43414E-07
TC09001516.hg.1	TNFSF8	944	tumor necrosis factor (ligand) superfamily, member 8	-6.703688	3.34925E-10	1.06469E-07
TC20000703.hg.1	CD93	22918	CD93 molecule	-6.881406	5.21748E-10	1.39356E-07
TC20000134.hg.1	RIN2	54453	Ras and Rab interactor 2	-7.013146	3.81782E-09	4.86008E-07
TC06001126.hg.1	SNX9	51429	sorting nexin 9	-7.253681	3.51675E-09	4.59631E-07
TC10001642.hg.1	CCDC147-AS1	100505869	CCDC147 antisense RNA 1 (head to head)	-7.272669	1.18719E-06	2.53599E-05
TC12000793.hg.1	DRAM1	55332	DNA-damage regulated autophagy modulator 1	-7.593439	3.19304E-07	9.74155E-06
TC03003320.hg.1	NFKBIZ	64332	nuclear factor of kappa light polypeptide gene enhancer in B-cells inhibitor, zeta	-7.632391	1.39749E-07	5.38469E-06
TC12002079.hg.1	HCAR3	8843	hydroxycarboxylic acid receptor 3	-7.684368	1.45364E-06	2.95396E-05
TC04001286.hg.1	CXCL2	2920	chemokine (C-X-C motif) ligand 2	-7.795708	2.46915E-09	3.71801E-07
TC02000720.hg.1	IL1RN	3557	interleukin 1 receptor antagonist	-7.882173	2.3425E-08	1.49244E-06
TC07001868.hg.1	LOC646329	646329	uncharacterized LOC646329	-7.987838	4.81644E-08	2.5129E-06
TC19000885.hg.1	FCAR	2204	Fc fragment of IgA, receptor for	-8.18753	3.80675E-07	1.0939E-05
TC21000169.hg.1	ETS2	2114	v-ets erythroblastosis virus E26 oncogene homolog 2 (avian)	-8.203815	2.29684E-09	3.56194E-07
TC19001457.hg.1	NFKBID	84807	nuclear factor of kappa light polypeptide gene enhancer in B-cells inhibitor, delta	-8.206868	2.90222E-08	1.72719E-06
TC0X000112.hg.1	SAT1	6303	spermidine/spermine N1-acetyltransferase 1	-8.265994	2.37822E-07	7.95229E-06
TC01002512.hg.1	CSF3R	1441	colony stimulating factor 3 receptor (granulocyte)	-8.347206	1.3917E-08	1.06635E-06
TC04001812.hg.1	MIR3945	100500818	microRNA 3945	-8.363963	8.38611E-07	1.95141E-05
TC16000494.hg.1	GPR97	222487	G protein-coupled receptor 97	-8.411657	9.93478E-06	0.000130035
TC19000676.hg.1	C5AR1	728	complement component 5a receptor 1	-8.418248	2.25114E-05	0.000246352
TC01002478.hg.1	RNF19B	127544	ring finger protein 19B	-8.451222	9.2964E-07	2.10415E-05
TC17001833.hg.1	KCNJ2-AS1	400617	KCNJ2 antisense RNA 1 (head to head)	-8.486571	7.6882E-05	0.000652046
TC07001738.hg.1	NAMPT	10135	nicotinamide phosphoribosyltransferase	-8.514027	2.49858E-07	8.19936E-06
TC04002922.hg.1	RASGEF1B	153020	RasGEF domain family, member 1B	-8.548631	6.213E-07	1.54918E-05
TC10000639.hg.1	IFIT1	3434	interferon-induced protein with tetratricopeptide repeats 1	-8.575684	0.000407362	0.002536393
TC02001911.hg.1	PELI1	57162	pellino E3 ubiquitin protein ligase 1	-8.884353	1.61433E-07	5.99352E-06
TC09001345.hg.1	NINJ1	4814	ninjurin 1	-8.962151	5.64974E-08	2.81213E-06
TC02001128.hg.1	NABP1	64859	nucleic acid binding protein 1	-9.20124	1.93028E-07	6.88326E-06
TC04001305.hg.1	CXCL10	3627	chemokine (C-X-C motif) ligand 10	-9.267756	0.009032343	0.03077394
TC20000341.hg.1	PI3	5266	peptidase inhibitor 3, skin-derived	-9.380501	3.17365E-10	1.05004E-07
TC17000117.hg.1	KDM6B	23135	lysine (K)-specific demethylase 6B	-9.45671	1.35984E-09	2.47545E-07
TC06000909.hg.1	MARCKS	4082	myristoylated alanine-rich protein kinase C substrate	-9.691567	2.63532E-07	8.48674E-06
TC06000120.hg.1	RNF144B	255488	ring finger protein 144B	-10.384849	1.18436E-07	4.78189E-06
TC04001811.hg.1	LOC731424	731424	uncharacterized LOC731424	-10.733956	1.33307E-07	5.20781E-06
TC19000711.hg.1	PPP1R15A	23645	protein phosphatase 1, regulatory subunit 15A	-10.755791	4.83254E-09	5.45311E-07
TC05002051.hg.1	LOC100128059	100128059	uncharacterized LOC100128059	-10.782115	3.13266E-12	1.08232E-08
TC10000053.hg.1	PFKFB3	5209	6-phosphofructo-2-kinase/fructose-2,6-biphosphatase 3	-10.88562	4.48725E-09	5.26348E-07
TC06000371.hg.1	TNF	7124	tumor necrosis factor	-11.189885	6.16413E-10	1.41553E-07
TC19000055.hg.1	GADD45B	4616	growth arrest and DNA-damage-inducible, beta	-11.324549	4.76157E-08	2.49155E-06
TC04001809.hg.1	ACSL1	2180	acyl-CoA synthetase long-chain family member 1	-11.453818	1.13218E-06	2.45053E-05
TC02001696.hg.1	BRE-AS1	100302650	BRE antisense RNA 1	-11.634495	1.57826E-07	5.88349E-06
TC17000408.hg.1	CCL4	6351	chemokine (C-C motif) ligand 4	-11.672041	3.47647E-05	0.000346722
TC12001178.hg.1	CLEC4E	26253	C-type lectin domain family 4, member E	-11.876532	8.67286E-07	1.99973E-05
TC10000637.hg.1	IFIT3	3437	interferon-induced protein with tetratricopeptide repeats 3	-11.933042	4.48739E-05	0.000424671
TC12001216.hg.1	OLR1	4973	oxidized low density lipoprotein (lectin-like) receptor 1	-12.062664	1.20261E-07	4.82818E-06
TC13000291.hg.1	IRG1	730249	immunoresponsive 1 homolog (mouse)	-12.100474	0.000577611	0.003385159
TC12001569.hg.1	GPR84	53831	G protein-coupled receptor 84	-12.141895	6.15208E-07	1.53443E-05
TC02004970.hg.1	MXD1	4084	MAX dimerization protein 1	-12.205547	8.04308E-08	3.59303E-06
TC02000034.hg.1	RSAD2	91543	radical S-adenosyl methionine domain containing 2	-12.353832	0.000412956	0.002564427
TC19000174.hg.1	ICAM1	3383	intercellular adhesion molecule 1	-12.442835	3.7963E-07	1.09096E-05
TC01002008.hg.1	NLRP3	114548	NLR family, pyrin domain containing 3	-12.757644	6.31495E-08	3.03685E-06
TC06000099.hg.1	CD83	9308	CD83 molecule	-12.830481	5.25339E-08	2.67541E-06
TC06004141.hg.1	SOD2	6648	superoxide dismutase 2, mitochondrial	-13.18798	1.24846E-08	9.92906E-07
TC02002218.hg.1	IL1A	3552	interleukin 1, alpha	-13.854458	2.54181E-07	8.29394E-06
TC22000648.hg.1	OSM	5008	oncostatin M	-14.393278	2.72376E-08	1.66302E-06
TC01001749.hg.1	G0S2	50486	G0/G1switch 2	-14.4093	4.92404E-08	2.55715E-06
TC04001810.hg.1	SLED1	643036	proteoglycan 3 pseudogene	-14.78964	6.85018E-07	1.67035E-05
TC17000423.hg.1	CCL4L1	9560	chemokine (C-C motif) ligand 4-like 1	-14.936446	1.37471E-07	5.33043E-06
TC09000508.hg.1	NR4A3	8013	nuclear receptor subfamily 4, group A, member 3	-15.525792	2.07518E-07	7.18008E-06
TC19001593.hg.1	PLAUR	5329	plasminogen activator, urokinase receptor	-16.637864	2.48858E-08	1.55545E-06
TC10000636.hg.1	IFIT2	3433	interferon-induced protein with tetratricopeptide repeats 2	-17.509367	3.3308E-05	0.000335995
TC03000056.hg.1	IRAK2	3656	interleukin-1 receptor-associated kinase 2	-17.638733	8.63008E-09	7.87551E-07
TC02000937.hg.1	TNFAIP6	7130	tumor necrosis factor, alpha-induced protein 6	-17.851705	2.19142E-05	0.000241467

transcript cluster id	Gene Symbol	Entrez Gene ID	Description	FoldChange (AIM/control)	P.Value (AIM/control)	adj.P.Val (AIM/control)
TC06001717.hg.1	TREM1	54210	triggering receptor expressed on myeloid cells 1	-18.38512	5.61906E-08	2.79816E-06
TC06001510.hg.1	IER3	8870	immediate early response 3	-18.559874	2.62917E-09	3.79989E-07
TC17000383.hg.1	CCL2	6347	chemokine (C-C motif) ligand 2	-18.866262	7.37571E-05	0.000630777
TC12002078.hg.1	HCAR2	338442	hydroxycarboxylic acid receptor 2	-19.109437	5.23537E-07	1.37736E-05
TC17000416.hg.1	CCL4L1	9560	chemokine (C-C motif) ligand 4-like 1	-20.362272	4.74191E-07	1.28084E-05
TC05001589.hg.1	LUCAT1	100505994	lung cancer associated transcript 1 (non-protein coding)	-20.631379	1.7361E-09	2.97728E-07
TC07000137.hg.1	IL6	3569	interleukin 6 (interferon, beta 2)	-24.025591	2.9416E-06	5.06656E-05
TC10000475.hg.1	PLAU	5328	plasminogen activator, urokinase	-27.827608	9.89751E-10	2.00071E-07
TC15000270.hg.1	THBS1	7057	thrombospondin 1	-29.348638	1.7535E-08	1.22449E-06
TC04000408.hg.1	IL8	3576	interleukin 8	-30.360529	5.69454E-07	1.46332E-05
TC15000441.hg.1	AQP9	366	aquaporin 9	-30.799144	6.61684E-09	6.63579E-07
TC02002219.hg.1	IL1B	3553	interleukin 1, beta	-34.572947	4.51721E-06	7.1004E-05
TC19000470.hg.1	FFAR2	2867	free fatty acid receptor 2	-37.999714	1.52297E-06	3.07026E-05
TC17001385.hg.1	CCL3	6348	chemokine (C-C motif) ligand 3	-50.121928	5.3019E-07	1.38889E-05
TC17001388.hg.1	CCL3L3	414062	chemokine (C-C motif) ligand 3-like 3	-68.239177	4.0501E-07	1.14707E-05
TC01003638.hg.1	PTGS2	5743	prostaglandin-endoperoxide synthase 2 (prostaglandin G/H synthase and cyclooxygenase	-68.338026	4.41819E-07	1.22508E-05
TC17001395.hg.1	CCL3L3	414062	chemokine (C-C motif) ligand 3-like 3	-69.846052	4.10683E-07	1.15877E-05

SupplementaryTable S3 -Differential genes CAEBV vs. CTRL

transcript cluster id	Gene Symbol	Entrez Gene ID	Description	FoldChange (CAEBV/control)	P.Value (CAEBV/control)	adj.P.Val (CAEBV/control)
TC16000473.hg.1	MT1F	4494	metallothionein 1F	6.824896	2.19E-03	0.04072506
TC01003057.hg.1	FCGR1B	2210	Fc fragment of IgG, high affinity Ib, receptor (CD64)	5.911559	1.07E-04	0.007435839
TC01001172.hg.1	FCGR1A	2209	Fc fragment of IgG, high affinity Ia, receptor (CD64)	5.403833	7.19E-05	0.006178474
TC01001161.hg.1	FCGR1C	100132417	Fc fragment of IgG, high affinity Ic, receptor (CD64), pseudogene	5.112693	2.32E-04	0.01182214
TC03001904.hg.1	P2RY14	9934	purinergic receptor P2Y, G-protein coupled, 14	4.981921	3.93E-04	0.01566411
TC05000227.hg.1	GZMK	3003	granzyme K (granzyme 3; tryptase II)	4.938196	1.34E-03	0.03172305
TC10001497.hg.1	ANKRD22	118932	ankyrin repeat domain 22	4.593239	5.73E-04	0.01879983
TC07001546.hg.1	FGL2	10875	fibrinogen-like 2	4.069256	2.48E-03	0.04439475
TC05000231.hg.1	GZMA	3001	granzyme A (granzyme 1, cytotoxic T-lymphocyte-associated serine esterase 3)	4.033046	0.000435799	0.01631952
TC01003796.hg.1	SLC30A1	7779	solute carrier family 30 (zinc transporter), member 1	3.746986	0.002726547	0.04635823
TC03001906.hg.1	P2RY13	53829	purinergic receptor P2Y, G-protein coupled, 13	3.555116	0.002996345	0.04856319
TC18000477.hg.1	PSTPIP2	9050	proline-serine-threonine phosphatase interacting protein 2	3.447714	0.000186226	0.01029691
TC09000035.hg.1	JAK2	3717	Janus kinase 2	3.156778	0.000046994	0.004865109
TC20000241.hg.1	MIR644A	693229	microRNA 644a	2.926487	1.73947E-05	0.002633372
TC14000981.hg.1	GZMH	2999	granzyme H (cathepsin G-like 2, protein h-CCPX)	2.656706	0.001597343	0.0350869
TC02002924.hg.1	MIR4441	100616493	microRNA 4441	2.624999	5.88686E-05	0.005483618
TC10001756.hg.1	MKI67	4288	antigen identified by monoclonal antibody Ki-67	2.557156	0.001762455	0.03645361
TC10001535.hg.1	MYOF	26509	myoferlin	2.503896	0.000544227	0.01819669
TC08001286.hg.1	MYBL1	4603	v-myb myeloblastosis viral oncogene homolog (avian)-like 1	2.475441	0.001236159	0.03011813
TC12000138.hg.1	KLRG1	10219	killer cell lectin-like receptor subfamily G, member 1	2.310367	8.97819E-05	0.006973136
TC11000572.hg.1	RARRES3	5920	retinoic acid receptor responder (tazarotene induced) 3	2.246411	7.48815E-07	0.000468492
TC15000364.hg.1	SQRDL	58472	sulfide quinone reductase-like (yeast)	2.237426	6.27947E-05	0.005673344
TC14000386.hg.1	SYNE2	23224	spectrin repeat containing, nuclear envelope 2	2.190961	0.000921208	0.02526854
TC09000463.hg.1	HIATL1	84641	hippocampus abundant transcript-like 1	2.175953	3.77964E-05	0.004285869
TC09000038.hg.1	PDCD1LG2	80380	programmed cell death 1 ligand 2	2.163824	0.000600581	0.01947535
TC03000715.hg.1	DNAJC13	23317	DnaJ (Hsp40) homolog, subfamily C, member 13	2.159274	0.000150651	0.009074596
TC04001171.hg.1	NFXL1	152518	nuclear transcription factor, X-box binding-like 1	2.114949	0.003045443	0.04890894
TC06000581.hg.1	UBR2	23304	ubiquitin protein ligase E3 component n-recognin 2	2.11379	4.97528E-05	0.004999366
TC06000523.hg.1	MAPK14	1432	mitogen-activated protein kinase 14	2.081303	0.002842938	0.0473339
TC01001840.hg.1	LOC100287497	100287497	uncharacterized LOC100287497	2.049332	0.000250721	0.01234591
TC01006390.hg.1	HNRNPU-AS1	284702	HNRNPU antisense RNA 1	2.028341	0.000333116	0.01447347
TC14000968.hg.1	PSME2	5721	proteasome (prosome, macropain) activator subunit 2 (PA28 beta)	2.013715	8.87416E-05	0.006973136
TC05001295.hg.1	FYB	2533	FYN binding protein	2.005313	5.21723E-05	0.005125277
TC05001331.hg.1	EMB	133418	embigin	2.001129	4.56571E-05	0.004862438
TC02002766.hg.1	CXCR2P1	3580	chemokine (C-X-C motif) receptor 2 pseudogene 1	2.000741	0.002396186	0.04330446
TC06001355.hg.1	HIST1H4H	8365	histone cluster 1, H4h	1.993958	0.000689898	0.02116644
TC03001537.hg.1	MIR3136	100422859	microRNA 3136	1.965299	0.000282835	0.0131547
TC11002175.hg.1	CTSC	1075	cathepsin C	1.963773	4.71246E-07	0.00036142
TC01002903.hg.1	DPYD	1806	dihydropyrimidine dehydrogenase	1.929423	0.00283787	0.04732997
TC03001851.hg.1	XRN1	54464	5'-3' exoribonuclease 1	1.924276	6.2851E-05	0.005684951
TC13000491.hg.1	PARP4	143	poly (ADP-ribose) polymerase family, member 4	1.913388	1.41793E-05	0.002346091
TC01001090.hg.1	TXNIP	10628	thioredoxin interacting protein	1.906297	0.000221832	0.01163974
TC18000506.hg.1	ACAA2	10449	acetyl-CoA acyltransferase 2	1.901792	0.000120029	0.007963656
TC04001718.hg.1	DDX60	55601	DEAD (Asp-Glu-Ala-Asp) box polypeptide 60	1.885602	0.00018509	0.01029691
TC01001347.hg.1	PYHIN1	149628	pyrin and HIN domain family, member 1	1.877669	0.001230643	0.03011813
TC01000286.hg.1	C1QC	714	complement component 1, q subcomponent, C chain	1.872626	0.001436579	0.0331514
TC01002954.hg.1	SORT1	6272	sortilin 1	1.867513	0.000525357	0.01819669
TC02004995.hg.1	IL18R1	8809	interleukin 18 receptor 1	1.867204	0.002521793	0.04473924
TC10002930.hg.1	ERLIN1	10613	ER lipid raft associated 1	1.831121	0.003100914	0.04946367
TC06000186.hg.1	BTN3A1	11119	butyrophilin, subfamily 3, member A1	1.829124	0.000423991	0.01622381
TC12000467.hg.1	NCKAP1L	3071	NCK-associated protein 1-like	1.826943	1.00704E-05	0.001978921
TC05000209.hg.1	PARP8	79668	poly (ADP-ribose) polymerase family, member 8	1.825988	0.000288706	0.01332029
TC04000151.hg.1	LAP3	51056	leucine aminopeptidase 3	1.792258	0.002624798	0.04552769
TC07000545.hg.1	AKAP9	10142	A kinase (PRKA) anchor protein (yotiao) 9	1.791893	5.05361E-05	0.00507231
TC02001083.hg.1	ITGA4	3676	integrin, alpha 4 (antigen CD49D, alpha 4 subunit of VLA-4 receptor)	1.78677	0.002408927	0.04350371
TC07000969.hg.1	CUL1	8454	cullin 1	1.780738	4.40929E-05	0.004796921
TC01001053.hg.1	EMBP1	647121	embigin pseudogene 1	1.767436	0.00046896	0.01700571
TC03000873.hg.1	SMC4	10051	structural maintenance of chromosomes 4	1.761147	0.000539354	0.01819669
TC14001085.hg.1	MIS18BP1	55320	MIS18 binding protein 1	1.757606	6.01798E-06	0.001567471
TC01003405.hg.1	CD84	8832	CD84 molecule	1.754194	1.4474E-05	0.002377532
TC11002009.hg.1	CPT1A	1374	carnitine palmitoyltransferase 1A (liver)	1.73676	0.001541412	0.03465332
TC14000326.hg.1	KTN1	3895	kinectin 1 (kinesin receptor)	1.725706	8.35768E-05	0.006810759
TC14001348.hg.1	SPTLC2	9517	serine palmitoyltransferase, long chain base subunit 2	1.718219	7.39E-06	0.001782452
TC05000639.hg.1	RAD50	10111	RAD50 homolog (S. cerevisiae)	1.710894	1.60E-03	0.03520809
TC12001296.hg.1	RECQL	5965	RecQ protein-like (DNA helicase Q1-like)	1.710462	3.04E-04	0.01387732
TC02002923.hg.1	MIR4440	100616397	microRNA 4440	1.705535	9.27E-04	0.02546052
TC08001288.hg.1	VCPIP1	80124	valosin containing protein (p97)/p47 complex interacting protein 1	1.70388	1.12E-04	0.007686689
TC18000180.hg.1	ME2	4200	malic enzyme 2, NAD(+)-dependent, mitochondrial	1.703705	0.000384399	0.01566411
TC04001476.hg.1	TIFA	92610	TRAF-interacting protein with forkhead-associated domain	1.701827	0.000251963	0.01246771
TC10001101.hg.1	PIP4K2A	5305	phosphatidylinositol-5-phosphate 4-kinase, type II, alpha	1.69919	0.002046448	0.03933833
TC13000552.hg.1	N4BP2L2-IT2	116828	N4BPL2 intronic transcript 2 (non-protein coding)	1.695738	0.001786063	0.03672707
TC01001830.hg.1	BROX	148362	BRO1 domain and CAAX motif containing	1.695478	4.19384E-05	0.004629679
TC09000335.hg.1	ANXA1	301	annexin A1	1.693675	3.64967E-05	0.004240823
TC09000607.hg.1	CNTRL	11064	centriolin	1.689081	0.000261876	0.01264519

transcript cluster id	Gene Symbol	Entrez Gene ID	Description	FoldChange (CAEBV/control)	P.Value (CAEBV/control)	adj.P.Val (CAEBV/control)
TC02002467.hg.1	LY75-CD302	100526664	LY75-CD302 readthrough	1.678691	0.000535272	0.01819669
TC01001640.hg.1	PTPRC	5788	protein tyrosine phosphatase, receptor type, C	1.674923	4.55531E-05	0.004865109
TC06004098.hg.1	PHACTR2	9749	phosphatase and actin regulator 2	1.673786	5.4895E-06	0.001474856
TC15001264.hg.1	ZNF106	64397	zinc finger protein 106	1.673658	0.000986741	0.02658113
TC06000188.hg.1	BTN3A3	10384	butyrophilin, subfamily 3, member A3	1.668212	0.002109766	0.04009659
TC15001685.hg.1	ETFA	2108	electron-transfer-flavoprotein, alpha polypeptide	1.667132	2.43229E-06	0.000986821
TC12001938.hg.1	SELPLG	6404	selectin P ligand	1.665101	0.000403203	0.01610578
TC09001673.hg.1	SETX	23064	senataxin	1.663891	0.000325456	0.01439629
TC09001547.hg.1	STOM	2040	stomatin	1.659255	0.000260077	0.01260337
TC10001097.hg.1	DNAJC1	64215	DnaJ (Hsp40) homolog, subfamily C, member 1	1.652119	7.61179E-05	0.006373769
TC17001372.hg.1	SLFN12L	100506736	schlafen family member 12-like	1.65102	0.000374235	0.01554216
TC14000572.hg.1	GOLGA5	9950	golgin A5	1.650552	1.59964E-05	0.002552257
TC03000417.hg.1	ARL6IP5	10550	ADP-ribosylation-like factor 6 interacting protein 5	1.643783	2.19791E-05	0.003137494
TC12000012.hg.1	WNK1	65125	WNK lysine deficient protein kinase 1	1.640683	0.001104082	0.02827508
TC01000287.hg.1	C1QB	713	complement component 1, q subcomponent, B chain	1.637483	0.000185092	0.01033293
TC10001674.hg.1	MIR2110	100302224	microRNA 2110	1.626849	0.001071223	0.02778701
TC06002103.hg.1	SAMD3	154075	sterile alpha motif domain containing 3	1.623865	0.001273172	0.03071227
TC12001766.hg.1	PPP1R12A	4659	protein phosphatase 1, regulatory subunit 12A	1.623792	3.34688E-05	0.004060383
TC12000325.hg.1	IRAK4	51135	interleukin-1 receptor-associated kinase 4	1.617385	0.000189445	0.01047847
TC15001296.hg.1	SPG11	80208	spastic paraplegia 11 (autosomal recessive)	1.616107	0.001914826	0.03820404
TC04001805.hg.1	IRF2	3660	interferon regulatory factor 2	1.615519	0.000730713	0.02210348
TC06004137.hg.1	ARHGAP18	93663	Rho GTPase activating protein 18	1.615234	1.15724E-05	0.002158557
TC03000761.hg.1	RASA2	5922	RAS p21 protein activator 2	1.613151	0.000556795	0.01863459
TC11000133.hg.1	ILK	3611	integrin-linked kinase	1.612896	1.34352E-05	0.002325355
TC01003887.hg.1	LBR	3930	lamin B receptor	1.609683	2.60278E-05	0.003401214
TC04002955.hg.1	ARAP2	116984	ArfGAP with RhoGAP domain, ankyrin repeat and PH domain 2	1.60758	0.002651051	0.04580096
TC04001177.hg.1	FRYL	285527	FRY-like	1.606924	0.000955225	0.02615537
TC11001839.hg.1	MS4A6A	64231	membrane-spanning 4-domains, subfamily A, member 6A	1.601074	0.000790705	0.02300787
TC12003283.hg.1	TMBIM4	51643	transmembrane BAX inhibitor motif containing 4	1.597969	0.001957749	0.03870911
TC17001378.hg.1	CCL5	6352	chemokine (C-C motif) ligand 5	1.595332	0.002035217	0.03929476
TC06001895.hg.1	PHIP	55023	pleckstrin homology domain interacting protein	1.587722	5.5917E-05	0.005415672
TC17000362.hg.1	NF1	4763	neurofibromin 1	1.581166	8.53463E-05	0.006933737
TC14001372.hg.1	SEL1L	6400	sel-1 suppressor of lin-12-like (C. elegans)	1.581162	1.72377E-05	0.002656818
TC17001408.hg.1	SYNRG	11276	synergin, gamma	1.580366	0.001812156	0.0369733
TC01002649.hg.1	EPS15	2060	epidermal growth factor receptor pathway substrate 15	1.578862	0.000397338	0.01599221
TC03001549.hg.1	EIF4E3	317649	eukaryotic translation initiation factor 4E family member 3	1.575442	2.23335E-05	0.003157523
TC09000925.hg.1	PSIP1	11168	PC4 and SFRS1 interacting protein 1	1.571656	0.001942806	0.03865349
TC08000767.hg.1	EFR3A	23167	EFR3 homolog A (S. cerevisiae)	1.57165	2.67218E-05	0.003472604
TC12001184.hg.1	M6PR	4074	mannose-6-phosphate receptor (cation dependent)	1.566171	0.001766528	0.03666019
TC06004076.hg.1	C2	717	complement component 2	1.565055	2.1604E-06	0.000923417
TC02000666.hg.1	GCC2	9648	GRIP and coiled-coil domain containing 2	1.564103	0.000270045	0.01286928
TC14001097.hg.1	NEMF	9147	nuclear export mediator factor	1.560915	9.2264E-06	0.001947592
TC01000835.hg.1	GBP6	163351	guanylate binding protein family, member 6	1.5603	0.000886483	0.02483424
TC03000221.hg.1	NKTR	4820	natural killer-tumor recognition sequence	1.55898	0.002893724	0.04776735
TC21000039.hg.1	USP25	29761	ubiquitin specific peptidase 25	1.558237	4.71218E-05	0.004951229
TC02000213.hg.1	BIRC6	57448	baculoviral IAP repeat containing 6	1.558146	8.13002E-05	0.006771434
TC18000476.hg.1	EPG5	57724	ectopic P-granules autophagy protein 5 homolog (C. elegans)	1.557751	0.001038426	0.02744322
TC01003636.hg.1	TPR	7175	translocated promoter region, nuclear basket protein	1.557415	0.001508223	0.03448431
TC14000568.hg.1	CPSF2	53981	cleavage and polyadenylation specific factor 2, 100kDa	1.553574	0.000603846	0.01963457
TC16001136.hg.1	FAM192A	80011	family with sequence similarity 192, member A	1.553239	1.59945E-06	0.000755692
TC01000934.hg.1	STXBP3	6814	syntaxin binding protein 3	1.55225	0.002874499	0.04768332
TC02005060.hg.1	CALM2	805	calmodulin 2 (phosphorylase kinase, delta)	1.550063	1.13566E-06	0.000652633
TC05000277.hg.1	PPWD1	23398	peptidylprolyl isomerase domain and WD repeat containing 1	1.548258	0.000501448	0.01782493
TC10001069.hg.1	RSU1	6251	Ras suppressor protein 1	1.544462	0.000890489	0.02486745
TC11001124.hg.1	GRAMD1B	57476	GRAM domain containing 1B	1.542441	0.001783213	0.03678576
TC15000335.hg.1	PDIA3	2923	protein disulfide isomerase family A, member 3	1.541876	0.000436928	0.0164587
TC09001287.hg.1	AGTPBP1	23287	ATP/GTP binding protein 1	1.541424	0.001267866	0.03070956
TC11001724.hg.1	CKAP5	9793	cytoskeleton associated protein 5	1.539507	0.002842336	0.0475798
TC12001208.hg.1	CLEC2B	9976	C-type lectin domain family 2, member B	1.538426	0.000979399	0.02657062
TC06001302.hg.1	DEK	7913	DEK oncogene	1.537221	0.000353235	0.01504367
TC11002246.hg.1	CASP4	837	caspase 4, apoptosis-related cysteine peptidase	1.537167	0.002894939	0.04776735
TC09001163.hg.1	LOC642236	642236	FSHD region gene 1 pseudogene	1.536544	0.000129462	0.008411908
TC11000908.hg.1	KIAA1731	85459	KIAA1731	1.533824	3.76244E-05	0.004339459
TC02000192.hg.1	CLIP4	79745	CAP-GLY domain containing linker protein family, member 4	1.53075	3.77529E-05	0.004339459
TC02000513.hg.1	VAMP5	10791	vesicle-associated membrane protein 5	1.530423	0.001805087	0.0369733
TC02000146.hg.1	RAB10	10890	RAB10, member RAS oncogene family	1.526911	0.000288391	0.01338402
TC01001214.hg.1	SNX27	81609	sorting nexin family member 27	1.526665	0.001346908	0.03190439
TC12000139.hg.1	A2M-AS1	144571	A2M antisense RNA 1	1.526605	9.55683E-06	0.001966924
TC20000361.hg.1	CTSA	5476	cathepsin A	1.523141	0.000691693	0.02132066
TC09000092.hg.1	DENND4C	55667	DENN/MADD domain containing 4C	1.51846	0.002022495	0.03929476
TC12000329.hg.1	ANO6	196527	anoctamin 6	1.518297	0.00051632	0.01812963
TC03001650.hg.1	ATG3	64422	autophagy related 3	1.516621	8.68948E-05	0.006973136
TC20000178.hg.1	FRG1B	284802	FSHD region gene 1 family, member B	1.515363	0.000221364	0.01168965
TC15001465.hg.1	RAB27A	5873	RAB27A, member RAS oncogene family	1.514876	8.9979E-05	0.006998998
TC0X000609.hg.1	SH2D1A	4068	SH2 domain containing 1A	1.512715	0.000558313	0.0187245
TC02001271.hg.1	XRCC5	7520	X-ray repair complementing defective repair in Chinese hamster cells 5 (double-strand-br	1.508532	0.000254483	0.01255066

transcript cluster id	Gene Symbol	Entrez Gene ID	Description	FoldChange (CAEBV/control)	P.Value (CAEBV/control)	adj.P.Val (CAEBV/control)
TC01003623.hg.1	EDEM3	80267	ER degradation enhancer, mannosidase alpha-like 3	1.508131	0.000435566	0.0164587
TC15000731.hg.1	PSMA4	5685	proteasome (prosome, macropain) subunit, alpha type, 4	1.505655	0.000635178	0.02016456
TC16000597.hg.1	ATXN1L	342371	ataxin 1-like	-1.501427	0.000363422	0.01533423
TC06001146.hg.1	WTAP	9589	Wilms tumor 1 associated protein	-1.50145	0.002012028	0.03925519
TC19000260.hg.1	CLEC17A	388512	C-type lectin domain family 17, member A	-1.50151	0.000387664	0.01577173
TC05001315.hg.1	LOC100132356	100132356	uncharacterized LOC100132356	-1.501663	0.000102213	0.007435839
TC03001684.hg.1	CD80	941	CD80 molecule	-1.50322	0.00032734	0.01447347
TC20000395.hg.1	SNAI1	6615	snail family zinc finger 1	-1.504877	0.001262457	0.03070956
TC02001885.hg.1	BCL11A	53335	B-cell CLL/lymphoma 11A (zinc finger protein)	-1.505574	0.000128122	0.00837537
TC17001933.hg.1	EIF4A3	9775	eukaryotic translation initiation factor 4A3	-1.510103	0.002515718	0.04477791
TC07000878.hg.1	LOC100134229	100134229	uncharacterized LOC100134229	-1.513518	4.40661E-05	0.004844662
TC09001411.hg.1	ALG2	85365	ALG2, alpha-1,3/1,6-mannosyltransferase	-1.514859	0.001146942	0.02907494
TC03000207.hg.1	EIF1B	10289	eukaryotic translation initiation factor 1B	-1.516761	0.000265079	0.01283623
TC11000484.hg.1	DTX4	23220	deltex homolog 4 (Drosophila)	-1.518735	0.00167095	0.03601172
TC19000812.hg.1	ZNF331	55422	zinc finger protein 331	-1.520834	0.000575578	0.0191095
TC19000454.hg.1	GRAMD1A	57655	GRAM domain containing 1A	-1.525045	0.0009	0.02502562
TC11000332.hg.1	CD44	960	CD44 molecule (Indian blood group)	-1.530187	0.002512251	0.04473924
TC14001564.hg.1	GPR132	29933	G protein-coupled receptor 132	-1.530846	0.000313811	0.01415585
TC10001555.hg.1	BLNK	29760	B-cell linker	-1.533071	0.000542347	0.01831688
TC09001691.hg.1	SLC2A6	11182	solute carrier family 2 (facilitated glucose transporter), member 6	-1.533477	4.76037E-05	0.004955053
TC19001586.hg.1	LYPD3	27076	LY6/PLAUR domain containing 3	-1.533928	8.65462E-05	0.006973136
TC02000874.hg.1	MGAT5	4249	mannosyl (alpha-1,6-)-glycoprotein beta-1,6-N-acetyl-glucosaminyltransferase	-1.535022	0.001966614	0.03875313
TC05001066.hg.1	LINC00847	729678	long intergenic non-protein coding RNA 847	-1.535478	2.26182E-05	0.003193533
TC11000985.hg.1	ZC3H12C	85463	zinc finger CCCH-type containing 12C	-1.536483	5.57294E-05	0.005415672
TC14000417.hg.1	KIAA0247	9766	KIAA0247	-1.543099	0.002400369	0.04353036
TC15001690.hg.1	TSPAN3	10099	tetraspanin 3	-1.543663	0.000603502	0.01963457
TC14000915.hg.1	HNRNPC	3183	heterogeneous nuclear ribonucleoprotein C (C1/C2)	-1.544112	0.000455213	0.01679811
TC13000828.hg.1	GPR18	2841	G protein-coupled receptor 18	-1.544661	0.001624924	0.03559822
TC17000897.hg.1	SYNGR2	9144	synaptogyrin 2	-1.544995	0.002617676	0.04556724
TC07001740.hg.1	CCDC71L	168455	coiled-coil domain containing 71-like	-1.551638	0.000505962	0.01784964
TC04000447.hg.1	PRDM8	56978	PR domain containing 8	-1.556844	5.304E-06	0.001464182
TC19000729.hg.1	CD37	951	CD37 molecule	-1.557736	0.00108423	0.02798867
TC07000250.hg.1	RALA	5898	v-ral simian leukemia viral oncogene homolog A (ras related)	-1.557944	0.001138056	0.02887909
TC01001138.hg.1	LOC100132999	100132999	uncharacterized LOC100132999	-1.559553	0.000355601	0.01509265
TC0X001020.hg.1	PIM2	11040	pim-2 oncogene	-1.560578	0.000992839	0.02676979
TC10001297.hg.1	CSTF2T	23283	cleavage stimulation factor, 3' pre-RNA, subunit 2, 64kDa, tau variant	-1.560698	0.002712297	0.04649022
TC07001349.hg.1	SNORA9	677798	small nucleolar RNA, H/ACA box 9	-1.561859	0.00036708	0.01539309
TC15000870.hg.1	FURIN	5045	furin (paired basic amino acid cleaving enzyme)	-1.56195	0.003078377	0.04941618
TC08001602.hg.1	MTSS1	9788	metastasis suppressor 1	-1.562136	0.001513033	0.03448431
TC04000678.hg.1	CCRN4L	25819	CCR4 carbon catabolite repression 4-like (S. cerevisiae)	-1.563802	0.00046169	0.01692269
TC17001808.hg.1	GNA13	10672	guanine nucleotide binding protein (G protein), alpha 13	-1.56571	0.000763813	0.02266125
TC20000980.hg.1	PMEPA1	56937	prostate transmembrane protein, androgen induced 1	-1.567875	1.5018E-06	0.000727875
TC19000339.hg.1	FAM129C	199786	family with sequence similarity 129, member C	-1.568145	7.91642E-06	0.001846628
TC03001089.hg.1	LOC100128262	100128262	uncharacterized LOC100128262	-1.568888	0.000610494	0.01972183
TC06001226.hg.1	TUBB2A	7280	tubulin, beta 2A class IIa	-1.569141	5.18173E-05	0.005125277
TC11003482.hg.1	SLC43A3	29015	solute carrier family 43, member 3	-1.570184	0.00134288	0.03185805
TC19000937.hg.1	ZNF460	10794	zinc finger protein 460	-1.573616	7.6988E-05	0.006433841
TC06001777.hg.1	TNFRSF21	27242	tumor necrosis factor receptor superfamily, member 21	-1.576003	1.48932E-06	0.000727875
TC17001032.hg.1	CHRNE	1145	cholinergic receptor, nicotinic, epsilon (muscle)	-1.577065	3.42782E-05	0.004152561
TC11002116.hg.1	LRRC32	2615	leucine rich repeat containing 32	-1.577928	2.42282E-05	0.003271702
TC20000051.hg.1	PRNP	5621	prion protein	-1.5783	0.000237421	0.01215157
TC20000403.hg.1	PTPN1	5770	protein tyrosine phosphatase, non-receptor type 1	-1.579099	0.000437672	0.0164587
TC20000019.hg.1	SIRPA	140885	signal-regulatory protein alpha	-1.579359	0.002772069	0.04682418
TC12000868.hg.1	SH2B3	10019	SH2B adaptor protein 3	-1.579702	0.000125924	0.008266659
TC12001656.hg.1	C12orf61	283416	chromosome 12 open reading frame 61	-1.584812	1.69885E-07	0.000251226
TC03000862.hg.1	PTX3	5806	pentraxin 3, long	-1.584955	0.002056532	0.0395265
TC10000093.hg.1	CAMK1D	57118	calcium/calmodulin-dependent protein kinase ID	-1.589521	0.000196976	0.01079855
TC08001099.hg.1	DUSP4	1846	dual specificity phosphatase 4	-1.589642	0.000846661	0.02392155
TC13001719.hg.1	FOXO1	2308	forkhead box O1	-1.589652	0.00031176	0.0141277
TC02000668.hg.1	RANBP2	5903	RAN binding protein 2	-1.592482	0.001879105	0.03780539
TC05000658.hg.1	MIR3661	100500905	microRNA 3661	-1.593688	0.000217751	0.01157454
TC01001569.hg.1	IER5	51278	immediate early response 5	-1.593814	0.001121581	0.02859343
TC12000512.hg.1	IL23A	51561	interleukin 23, alpha subunit p19	-1.596619	2.71731E-07	0.000300376
TC08001684.hg.1	AGO2	27161	argonaute RISC catalytic component 2	-1.597648	0.002849307	0.04758088
TC06001502.hg.1	PPP1R10	5514	protein phosphatase 1, regulatory subunit 10	-1.598749	0.003150087	0.04988971
TC10000565.hg.1	ZMIZ1	57178	zinc finger, MIZ-type containing 1	-1.60017	0.000373598	0.01554216
TC01003720.hg.1	LOC730227	730227	uncharacterized LOC730227	-1.600857	6.50346E-07	0.000468492
TC01000116.hg.1	SLC25A33	84275	solute carrier family 25 (pyrimidine nucleotide carrier), member 33	-1.601764	0.000842495	0.0238563
TC06000273.hg.1	ZNF165	7718	zinc finger protein 165	-1.604916	3.2002E-06	0.001173735
TC0X000220.hg.1	CHST7	56548	carbohydrate (N-acetylglucosamine 6-O) sulfotransferase 7	-1.609546	7.08128E-07	0.000468492
TC01003874.hg.1	TP53BP2	7159	tumor protein p53 binding protein, 2	-1.61272	0.001999782	0.03907724
TC09000560.hg.1	UGCG	7357	UDP-glucose ceramide glucosyltransferase	-1.614045	0.00062735	0.02007106
TC01000955.hg.1	CSF1	1435	colony stimulating factor 1 (macrophage)	-1.615364	0.002615299	0.04552769
TC14001451.hg.1	LGMN	5641	legumain	-1.616358	1.83331E-05	0.002739919
TC01000194.hg.1	PLEKHM2	23207	pleckstrin homology domain containing, family M (with RUN domain) member 2	-1.617454	0.000457874	0.01679811
TC01003608.hg.1	RGS16	6004	regulator of G-protein signaling 16	-1.617614	1.4953E-07	0.000251226

transcript cluster id	Gene Symbol	Entrez Gene ID	Description	FoldChange (CAEBV/control)	P.Value (CAEBV/control)	adj.P.Val (CAEBV/control)
TC19000466.hg.1	FFAR1	2864	free fatty acid receptor 1	-1.618523	2.54093E-05	0.003384087
TC12001807.hg.1	BTG1	694	B-cell translocation gene 1, anti-proliferative	-1.621829	0.000145015	0.008937197
TC08000022.hg.1	AGPAT5	55326	1-acylglycerol-3-phosphate O-acyltransferase 5	-1.622264	0.000141131	0.008800028
TC16000098.hg.1	MMP25	64386	matrix metallopeptidase 25	-1.622371	0.002160869	0.04069996
TC16000312.hg.1	CD19	930	CD19 molecule	-1.622427	0.000163068	0.009583616
TC16002106.hg.1	SLC7A5P1	81893	solute carrier family 7 (amino acid transporter light chain, L system), member 5 pseudoge	-1.624894	0.001789756	0.03678928
TC06000795.hg.1	PNRC1	10957	proline-rich nuclear receptor coactivator 1	-1.627894	3.94204E-05	0.004462194
TC03000951.hg.1	PIK3CA	5290	phosphatidylinositol-4,5-bisphosphate 3-kinase, catalytic subunit alpha	-1.628183	0.002364653	0.04304503
TC02000617.hg.1	MAP4K4	9448	mitogen-activated protein kinase kinase kinase kinase 4	-1.628416	4.51244E-06	0.001291964
TC03001546.hg.1	FOXP1	27086	forkhead box P1	-1.630252	0.001196415	0.02980846
TC19000521.hg.1	SIPA1L3	23094	signal-induced proliferation-associated 1 like 3	-1.633353	0.00033013	0.01447347
TC17000965.hg.1	FAM101B	359845	family with sequence similarity 101, member B	-1.634267	0.001904183	0.03812042
TC08001120.hg.1	RNF122	79845	ring finger protein 122	-1.63462	0.002580517	0.04536813
TC18000527.hg.1	TCF4	6925	transcription factor 4	-1.635805	4.50798E-06	0.001291964
TC20000387.hg.1	SNORD12C	26765	small nucleolar RNA, C/D box 12C	-1.636515	0.001522322	0.03454147
TC03001767.hg.1	H1FX	8971	H1 histone family, member X	-1.63946	8.95499E-05	0.006998998
TC0X001412.hg.1	MMGT1	93380	membrane magnesium transporter 1	-1.642465	6.42556E-05	0.005760167
TC17000875.hg.1	SNORD1C	677850	small nucleolar RNA, C/D box 1C	-1.647564	0.000430896	0.01631952
TC04001813.hg.1	LOC100506229	100506229	uncharacterized LOC100506229	-1.650053	9.89615E-05	0.007308599
TC06000370.hg.1	LTA	4049	lymphotoxin alpha (TNF superfamily, member 1)	-1.650803	0.000538465	0.01819669
TC20000244.hg.1	TP53INP2	58476	tumor protein p53 inducible nuclear protein 2	-1.652203	1.16089E-05	0.002158557
TC20000871.hg.1	TOMM34	10953	translocase of outer mitochondrial membrane 34	-1.656213	0.000426456	0.01628837
TC06002209.hg.1	ZC3H12D	340152	zinc finger CCCH-type containing 12D	-1.65665	5.22384E-05	0.005125277
TC01001077.hg.1	LOC101060698	101060698	uncharacterized LOC101060698	-1.657874	0.001258194	0.0305317
TC01003359.hg.1	ETV3	2117	ets variant 3	-1.659541	7.19165E-05	0.006231629
TC11000229.hg.1	NCR3LG1	374383	natural killer cell cytotoxicity receptor 3 ligand 1	-1.668352	5.68463E-05	0.005440434
TC0X000788.hg.1	MIR1184-2	100422985	microRNA 1184-2	-1.671076	0.000410782	0.01610578
TC0X001548.hg.1	MIR1184-1	100302111	microRNA 1184-1	-1.671076	0.000410782	0.01610578
TC0X001553.hg.1	MIR1184-2	100422985	microRNA 1184-2	-1.671076	0.000410782	0.01610578
TC19000350.hg.1	ARRDC2	27106	arrestin domain containing 2	-1.671505	0.000452907	0.01676963
TC07000125.hg.1	ITGB8	3696	integrin, beta 8	-1.674364	1.47004E-07	0.000251226
TC0Y000008.hg.1	IL3RA	3563	interleukin 3 receptor, alpha (low affinity)	-1.675118	1.09375E-05	0.00211401
TC22000304.hg.1	GTPBP1	9567	GTP binding protein 1	-1.682206	0.000681875	0.02109413
TC21000288.hg.1	NRIP1	8204	nuclear receptor interacting protein 1	-1.683064	0.000387649	0.01566411
TC05000184.hg.1	PTGER4	5734	prostaglandin E receptor 4 (subtype EP4)	-1.683582	0.001499462	0.03427994
TC10000946.hg.1	INPP5A	3632	inositol polyphosphate-5-phosphatase, 40kDa	-1.693737	0.000348374	0.01493103
TC07000111.hg.1	TSPAN13	27075	tetraspanin 13	-1.694669	0.000234085	0.01199989
TC01002155.hg.1	KLHL21	9903	kelch-like family member 21	-1.699775	1.42266E-05	0.002362603
TC0X000952.hg.1	DYNLT3	6990	dynein, light chain, Tctex-type 3	-1.706358	0.000129443	0.00837195
TC22000416.hg.1	PIM3	415116	pim-3 oncogene	-1.710672	0.000106314	0.007477657
TC05000226.hg.1	SNX18	112574	sorting nexin 18	-1.710939	0.002059364	0.0395265
TC22000682.hg.1	RTCB	51493	RNA 2',3'-cyclic phosphate and 5'-OH ligase	-1.711133	0.000119738	0.007963656
TC02000365.hg.1	LOC100507006	100507006	uncharacterized LOC100507006	-1.713748	1.24083E-05	0.002197629
TC01001113.hg.1	BCL9	607	B-cell CLL/lymphoma 9	-1.714732	0.002254173	0.04177081
TC0X000008.hg.1	IL3RA	3563	interleukin 3 receptor, alpha (low affinity)	-1.714805	8.20661E-06	0.001859808
TC21000198.hg.1	ABCG1	9619	ATP-binding cassette, sub-family G (WHITE), member 1	-1.717551	0.001028184	0.02727055
TC02001600.hg.1	FAM49A	81553	family with sequence similarity 49, member A	-1.717797	0.001735598	0.03631168
TC01000267.hg.1	LOC100506801	100506801	uncharacterized LOC100506801	-1.71926	1.09457E-05	0.00211401
TC17001922.hg.1	USP36	57602	ubiquitin specific peptidase 36	-1.719754	0.002548768	0.04501327
TC08001044.hg.1	EGR3	1960	early growth response 3	-1.721984	0.001551122	0.03475678
TC17000055.hg.1	C17orf107	100130311	chromosome 17 open reading frame 107	-1.722162	5.38817E-06	0.001464182
TC11001397.hg.1	DENND5A	23258	DENN/MADD domain containing 5A	-1.723627	0.001687497	0.03608453
TC16000381.hg.1	CLUHP3	100132341	clustered mitochondria (cluA/CLU1) homolog pseudogene 3	-1.72835	1.11891E-05	0.002116017
TC22001457.hg.1	ADORA2A	135	adenosine A2a receptor	-1.730585	0.000414364	0.01611723
TC05002050.hg.1	LCP2	3937	lymphocyte cytosolic protein 2 (SH2 domain containing leukocyte protein of 76kDa)	-1.735778	4.46122E-05	0.004839226
TC10000008.hg.1	GTPBP4	23560	GTP binding protein 4	-1.73633	0.001755568	0.03645511
TC12001255.hg.1	DUSP16	80824	dual specificity phosphatase 16	-1.736729	7.31716E-05	0.006235402
TC06001219.hg.1	MGC39372	221756	serpin peptidase inhibitor, clade B (ovalbumin), member 9 pseudogene	-1.738405	2.46177E-05	0.003271702
TC20000235.hg.1	CHMP4B	128866	charged multivesicular body protein 4B	-1.739737	0.000424209	0.01622381
TC12001361.hg.1	FAM60A	58516	family with sequence similarity 60, member A	-1.739967	0.000260008	0.01259157
TC17000868.hg.1	UBALD2	283991	UBA-like domain containing 2	-1.740509	0.000282133	0.0131547
TC16000862.hg.1	SOCS1	8651	suppressor of cytokine signaling 1	-1.743566	0.000696088	0.02131392
TC09000760.hg.1	GTF3C4	9329	general transcription factor IIIC, polypeptide 4, 90kDa	-1.744777	0.000269586	0.01283818
TC18000500.hg.1	SMAD7	4092	SMAD family member 7	-1.746048	0.000618124	0.01981864
TC07003328.hg.1	RABGEF1	27342	RAB guanine nucleotide exchange factor (GEF) 1	-1.747702	0.001386789	0.03238467
TC06002270.hg.1	TAGAP	117289	T-cell activation RhoGTPase activating protein	-1.756904	0.002992799	0.04868998
TC03003367.hg.1	LRRC33	375387	leucine rich repeat containing 33	-1.759796	5.57419E-08	0.000182845
TC01000909.hg.1	S1PR1	1901	sphingosine-1-phosphate receptor 1	-1.762763	0.001701223	0.03623268
TC15000840.hg.1	AEN	64782	apoptosis enhancing nuclease	-1.766287	7.1385E-05	0.006178474
TC11001847.hg.1	SLC15A3	51296	solute carrier family 15, member 3	-1.775718	0.000507878	0.01782493
TC16000384.hg.1	ZNF267	10308	zinc finger protein 267	-1.778496	0.002963441	0.04833427
TC05001946.hg.1	TNIP1	10318	TNFAIP3 interacting protein 1	-1.783319	0.002968158	0.04837069
TC17000486.hg.1	CSF3	1440	colony stimulating factor 3 (granulocyte)	-1.792463	0.00104714	0.02744322
TC01003784.hg.1	LAMB3	3914	laminin, beta 3	-1.79442	3.80827E-06	0.001212956
TC01002163.hg.1	TNFRSF9	3604	tumor necrosis factor receptor superfamily, member 9	-1.800228	0.000233344	0.01197912
TC22000835.hg.1	TNFRSF13C	115650	tumor necrosis factor receptor superfamily, member 13C	-1.810183	2.41556E-05	0.003257922

transcript cluster id	Gene Symbol	Entrez Gene ID	Description	FoldChange (CAEBV/control)	P.Value (CAEBV/control)	adj.P.Val (CAEBV/control)
TC11001401.hg.1	LOC644656	644656	uncharacterized LOC644656	-1.822041	1.80973E-05	0.002685941
TC19000117.hg.1	TNFSF9	8744	tumor necrosis factor (ligand) superfamily, member 9	-1.82234	1.0595E-05	0.002063271
TC03001071.hg.1	HES1	3280	hairy and enhancer of split 1, (Drosophila)	-1.824932	1.25076E-05	0.002197629
TC14000474.hg.1	BATF	10538	basic leucine zipper transcription factor, ATF-like	-1.825328	0.00099121	0.02658113
TC10000753.hg.1	NFKB2	4791	nuclear factor of kappa light polypeptide gene enhancer in B-cells 2 (p49/p100)	-1.827846	0.001560812	0.03484315
TC07001347.hg.1	PURB	5814	purine-rich element binding protein B	-1.839818	3.58322E-05	0.004202995
TC0X002305.hg.1	CRLF2	64109	cytokine receptor-like factor 2	-1.841535	0.000346623	0.01491655
TC0Y000347.hg.1	CRLF2	64109	cytokine receptor-like factor 2	-1.841535	0.000346623	0.01491655
TC14001029.hg.1	CFL2	1073	cofilin 2 (muscle)	-1.845044	0.000640532	0.02016456
TC22000796.hg.1	JOSD1	9929	Josephin domain containing 1	-1.853936	9.60098E-05	0.007123582
TC16000293.hg.1	IL4R	3566	interleukin 4 receptor	-1.855575	8.49637E-06	0.001859808
TC01000474.hg.1	ZC3H12A	80149	zinc finger CCCH-type containing 12A	-1.856923	0.003094408	0.04941618
TC16000230.hg.1	SNX29P1	100652781	sorting nexin 29 pseudogene 1	-1.859289	9.34127E-05	0.007056533
TC16000666.hg.1	IRF8	3394	interferon regulatory factor 8	-1.860284	4.62752E-06	0.001291964
TC07001039.hg.1	INSIG1	3638	insulin induced gene 1	-1.865981	0.000267591	0.01280196
TC11001073.hg.1	CXCR5	643	chemokine (C-X-C motif) receptor 5	-1.866329	3.86744E-06	0.001212956
TC10000323.hg.1	WDFY4	57705	WDFY family member 4	-1.873397	0.000162994	0.009520337
TC08000087.hg.1	BLK	640	B lymphoid tyrosine kinase	-1.874253	0.000142926	0.008802496
TC10001641.hg.1	ITPRIP	85450	inositol 1,4,5-trisphosphate receptor interacting protein	-1.876218	0.000347879	0.01493103
TC19001019.hg.1	MKNK2	2872	MAP kinase interacting serine/threonine kinase 2	-1.886768	0.000103416	0.007434151
TC19002665.hg.1	ZNF134	7693	zinc finger protein 134	-1.890575	0.000413199	0.01610578
TC16000165.hg.1	SNN	8303	stannin	-1.901385	0.001866114	0.03758527
TC12000627.hg.1	RAB21	23011	RAB21, member RAS oncogene family	-1.924217	0.002573801	0.04523774
TC04001592.hg.1	TBC1D9	23158	TBC1 domain family, member 9 (with GRAM domain)	-1.932836	0.000677717	0.02095927
TC19000758.hg.1	SPIB	6689	Spi-B transcription factor (Spi-1/PU.1 related)	-1.933324	1.45771E-06	0.000727875
TC03000407.hg.1	KBTBD8	84541	kelch repeat and BTB (POZ) domain containing 8	-1.934108	3.78872E-05	0.004295004
TC02000695.hg.1	BCL2L11	10018	BCL2-like 11 (apoptosis facilitator)	-1.934231	1.26989E-05	0.002197629
TC02002136.hg.1	AFF3	3899	AF4/FMR2 family, member 3	-1.940429	0.000351254	0.01493103
TC07001869.hg.1	MIR29A	407021	microRNA 29a	-1.941439	0.001363743	0.03198298
TC01000314.hg.1	CLIC4	25932	chloride intracellular channel 4	-1.944018	0.002592917	0.04536813
TC17000977.hg.1	SLC43A2	124935	solute carrier family 43, member 2	-1.947506	0.000635416	0.02008483
TC20000878.hg.1	SDC4	6385	syndecan 4	-1.949049	0.000127924	0.008266659
TC01001373.hg.1	LY9	4063	lymphocyte antigen 9	-1.959463	0.000121719	0.00800254
TC01003406.hg.1	SLAMF1	6504	signaling lymphocytic activation molecule family member 1	-1.963739	6.41832E-05	0.005751613
TC06001946.hg.1	BACH2	60468	BTB and CNC homology 1, basic leucine zipper transcription factor 2	-1.964872	0.001441888	0.03320698
TC21000516.hg.1	ICOSLG	23308	inducible T-cell co-stimulator ligand	-1.976551	0.000165501	0.00959535
TC12000558.hg.1	MIRLET7I	406891	microRNA let-7i	-1.97679	8.66357E-07	0.000525204
TC0X000219.hg.1	ZNF674-AS1	401588	ZNF674 antisense RNA 1 (head to head)	-1.985872	9.86329E-08	0.000226565
TC15000695.hg.1	NEIL1	79661	nei endonuclease VIII-like 1 (E. coli)	-1.992831	0.000135527	0.008522111
TC17000118.hg.1	TMEM88	92162	transmembrane protein 88	-2.010119	0.00032936	0.01439986
TC07000793.hg.1	TSPAN33	340348	tetraspanin 33	-2.017699	0.000364275	0.01517583
TC18000268.hg.1	ADNP2	22850	ADNP homeobox 2	-2.020061	0.000353926	0.01495211
TC19002660.hg.1	KIR2DS4	3809	killer cell immunoglobulin-like receptor, two domains, short cytoplasmic tail, 4	-2.028377	0.001786059	0.03670375
TC19000544.hg.1	ZFP36	7538	ZFP36 ring finger protein	-2.041753	0.000142495	0.008800028
TC0X000925.hg.1	KLHL15	80311	kelch-like family member 15	-2.047136	9.15419E-05	0.006998998
TC0X000963.hg.1	BCOR	54880	BCL6 corepressor	-2.048406	5.39997E-05	0.005194614
TC16000480.hg.1	HERPUD1	9709	homocysteine-inducible, endoplasmic reticulum stress-inducible, ubiquitin-like domain m	-2.054915	0.001438286	0.0331514
TC16000294.hg.1	IL21R	50615	interleukin 21 receptor	-2.057987	0.000271979	0.01283818
TC02002074.hg.1	EIF2AK3	9451	eukaryotic translation initiation factor 2-alpha kinase 3	-2.087124	0.000452593	0.01670219
TC06002265.hg.1	EZR	7430	ezrin	-2.093966	4.71836E-05	0.004876453
TC01000057.hg.1	SKI	6497	v-ski sarcoma viral oncogene homolog (avian)	-2.097554	0.000817658	0.02331008
TC05001110.hg.1	LPCAT1	79888	lysophosphatidylcholine acyltransferase 1	-2.102815	2.66753E-05	0.003401214
TC12003284.hg.1	RHOF	54509	ras homolog family member F (in filopodia)	-2.102865	4.24147E-05	0.004629679
TC03001468.hg.1	DCP1A	55802	decapping mRNA 1A	-2.102939	1.54807E-05	0.002480214
TC06002126.hg.1	SGK1	6446	serum/glucocorticoid regulated kinase 1	-2.104234	0.001812454	0.03687654
TC19002659.hg.1	KIR2DL1	3802	killer cell immunoglobulin-like receptor, two domains, long cytoplasmic tail, 1	-2.11459	0.000381658	0.01561189
TC10000413.hg.1	DDX21	9188	DEAD (Asp-Glu-Ala-Asp) box helicase 21	-2.116424	8.33584E-05	0.006777192
TC02000583.hg.1	ARID5A	10865	AT rich interactive domain 5A (MRF1-like)	-2.125814	0.002341268	0.04265508
TC01003985.hg.1	IRF2BP2	359948	interferon regulatory factor 2 binding protein 2	-2.14772	6.27419E-06	0.001589936
TC07001318.hg.1	INHBA	3624	inhibin, beta A	-2.16602	0.002166037	0.04063394
TC17000165.hg.1	HS3ST3B1	9953	heparan sulfate (glucosamine) 3-O-sulfotransferase 3B1	-2.168988	1.27315E-05	0.002197629
TC21000169.hg.1	ETS2	2114	v-ets erythroblastosis virus E26 oncogene homolog 2 (avian)	-2.169485	0.000832332	0.02345715
TC19001285.hg.1	JUND	3727	jun D proto-oncogene	-2.173362	0.000112243	0.007664407
TC05001995.hg.1	EBF1	1879	early B-cell factor 1	-2.177696	6.42936E-05	0.005751613
TC08000512.hg.1	ZBTB10	65986	zinc finger and BTB domain containing 10	-2.180745	0.000940574	0.02563221
TC19000545.hg.1	PLEKHG2	64857	pleckstrin homology domain containing, family G (with RhoGef domain) member 2	-2.18163	2.91952E-07	0.000300376
TC22000620.hg.1	MN1	4330	meningioma (disrupted in balanced translocation) 1	-2.182454	3.52126E-09	2.49406E-05
TC15000691.hg.1	C15orf39	56905	chromosome 15 open reading frame 39	-2.207656	0.000539738	0.01819669
TC13000829.hg.1	GPR183	1880	G protein-coupled receptor 183	-2.220284	9.29949E-06	0.001941909
TC01002322.hg.1	ECE1	1889	endothelin converting enzyme 1	-2.223851	9.68364E-05	0.007123582
TC19002661.hg.1	KIR2DL3	3804	killer cell immunoglobulin-like receptor, two domains, long cytoplasmic tail, 3	-2.228948	0.00116153	0.02907494
TC19001116.hg.1	FCER2	2208	Fc fragment of IgE, low affinity II, receptor for (CD23)	-2.230144	1.17697E-06	0.000652633
TC03001649.hg.1	BTLA	151888	B and T lymphocyte associated	-2.235758	0.000154791	0.009161342
TC06001279.hg.1	TBC1D7	51256	TBC1 domain family, member 7	-2.244246	1.9481E-06	0.000854781
TC01000566.hg.1	PLK3	1263	polo-like kinase 3	-2.251091	0.000534358	0.01819669
TC04000632.hg.1	SPRY1	10252	sprouty homolog 1, antagonist of FGF signaling (Drosophila)	-2.253558	0.001803825	0.03680085

transcript cluster id	Gene Symbol	Entrez Gene ID	Description	FoldChange (CAEBV/control)	P.Value (CAEBV/control)	adj.P.Val (CAEBV/control)
TC17000297.hg.1	MAP2K3	5606	mitogen-activated protein kinase kinase 3	-2.258183	7.19791E-08	0.000200275
TC17001860.hg.1	CD300E	342510	CD300e molecule	-2.258998	3.17484E-05	0.003835236
TC06000135.hg.1	SOX4	6659	SRY (sex determining region Y)-box 4	-2.269161	7.13854E-06	0.001725995
TC04000298.hg.1	MIR4449	100616436	microRNA 4449	-2.276674	0.003022603	0.04881692
TC17000330.hg.1	TRAF4	9618	TNF receptor-associated factor 4	-2.282898	4.27345E-06	0.001265106
TC02000183.hg.1	FOSL2	2355	FOS-like antigen 2	-2.296031	0.00056113	0.01861254
TC19001102.hg.1	TNFSF14	8740	tumor necrosis factor (ligand) superfamily, member 14	-2.296387	0.000105004	0.007435839
TC09001516.hg.1	TNFSF8	944	tumor necrosis factor (ligand) superfamily, member 8	-2.331354	3.54186E-05	0.004157772
TC03001302.hg.1	CSRNP1	64651	cysteine-serine-rich nuclear protein 1	-2.331925	0.000716487	0.02167637
TC14001253.hg.1	ZFP36L1	677	ZFP36 ring finger protein-like 1	-2.332661	0.001985382	0.03875313
TC17001661.hg.1	PHOSPHO1	162466	phosphatase, orphan 1	-2.332816	5.65769E-07	0.000413473
TC11001260.hg.1	DUSP8	1850	dual specificity phosphatase 8	-2.431903	4.49953E-06	0.001291964
TC17001917.hg.1	SOCS3	9021	suppressor of cytokine signaling 3	-2.449777	2.44479E-06	0.000974588
TC09001541.hg.1	TRAF1	7185	TNF receptor-associated factor 1	-2.459406	3.36262E-07	0.000300376
TC01003718.hg.1	CHI3L1	1116	chitinase 3-like 1 (cartilage glycoprotein-39)	-2.46194	1.65048E-07	0.000251226
TC06000608.hg.1	VEGFA	7422	vascular endothelial growth factor A	-2.463103	0.000239397	0.01210824
TC15001257.hg.1	EHD4	30844	EH-domain containing 4	-2.481111	7.14919E-05	0.006178474
TC12003253.hg.1	THAP2	83591	THAP domain containing, apoptosis associated protein 2	-2.509239	1.15043E-05	0.002116017
TC12001751.hg.1	PHLDA1	22822	pleckstrin homology-like domain, family A, member 1	-2.513785	3.56623E-06	0.001173735
TC02001746.hg.1	CDC42EP3	10602	CDC42 effector protein (Rho GTPase binding) 3	-2.523057	7.1333E-05	0.006178474
TC14000056.hg.1	PNP	4860	purine nucleoside phosphorylase	-2.542161	3.99507E-06	0.001212956
TC11000175.hg.1	SWAP70	23075	SWAP switching B-cell complex 70kDa subunit	-2.54791	0.000291398	0.01332029
TC09001072.hg.1	PAX5	5079	paired box 5	-2.60423	0.00030517	0.01385176
TC10001640.hg.1	MIR4482-1	100616323	microRNA 4482-1	-2.636714	0.000013917	0.002325355
TC02000620.hg.1	IL1R1	3554	interleukin 1 receptor, type I	-2.647266	2.11866E-06	0.000889419
TC11002153.hg.1	RAB30	27314	RAB30, member RAS oncogene family	-2.648874	1.60349E-06	0.000727875
TC12000698.hg.1	LINC00936	338758	long intergenic non-protein coding RNA 936	-2.668834	2.04216E-06	0.00087315
TC07001189.hg.1	LOC541472	541472	uncharacterized LOC541472	-2.671148	0.001962866	0.03865349
TC08001320.hg.1	MSC	9242	musculin	-2.730533	3.08362E-06	0.001124588
TC18000114.hg.1	TAF4B	6875	TAF4b RNA polymerase II, TATA box binding protein (TBP)-associated factor, 105kDa	-2.732961	0.000116936	0.007854507
TC11001915.hg.1	EHD1	10938	EH-domain containing 1	-2.747188	2.1638E-08	9.08767E-05
TC10001007.hg.1	IL2RA	3559	interleukin 2 receptor, alpha	-2.750956	9.37301E-06	0.001941909
TC21000408.hg.1	IL10RB-AS1	100288432	IL10RB antisense RNA 1 (head to head)	-2.763098	2.29318E-05	0.003157063
TC04000411.hg.1	CXCL1	2919	chemokine (C-X-C motif) ligand 1 (melanoma growth stimulating activity, alpha)	-2.80742	2.27744E-06	0.000923417
TC02001696.hg.1	BRE-AS1	100302650	BRE antisense RNA 1	-2.859772	0.002690753	0.04596106
TC13000644.hg.1	KIAA0226L	80183	KIAA0226-like	-2.951059	6.23469E-05	0.005630754
TC12000793.hg.1	DRAM1	55332	DNA-damage regulated autophagy modulator 1	-2.966569	0.00064537	0.02016456
TC01002008.hg.1	NLRP3	114548	NLR family, pyrin domain containing 3	-2.971904	0.001616121	0.03527875
TC08000749.hg.1	MYC	4609	v-myc myelocytomatosis viral oncogene homolog (avian)	-2.996621	1.81643E-05	0.002667247
TC06004142.hg.1	LOC100129518	100129518	uncharacterized LOC100129518	-3.003459	2.67056E-05	0.003401214
TC04000526.hg.1	NFKB1	4790	nuclear factor of kappa light polypeptide gene enhancer in B-cells 1	-3.006292	2.50835E-05	0.003271702
TC02000720.hg.1	IL1RN	3557	interleukin 1 receptor antagonist	-3.014228	0.000108813	0.007513062
TC10000566.hg.1	PPIF	10105	peptidylprolyl isomerase F	-3.015824	0.000308669	0.01387773
TC06001220.hg.1	SERPINB9	5272	serpin peptidase inhibitor, clade B (ovalbumin), member 9	-3.041428	1.58042E-06	0.000727875
TC02001364.hg.1	CCL20	6364	chemokine (C-C motif) ligand 20	-3.05968	0.000901445	0.02486026
TC12000413.hg.1	GRASP	160622	GRP1 (general receptor for phosphoinositides 1)-associated scaffold protein	-3.11412	8.76851E-08	0.000214952
TC10000053.hg.1	PFKFB3	5209	6-phosphofructo-2-kinase/fructose-2,6-biphosphatase 3	-3.168519	9.49444E-05	0.007068524
TC03000257.hg.1	CCRL2	9034	chemokine (C-C motif) receptor-like 2	-3.180826	4.59977E-06	0.001291964
TC19000711.hg.1	PPP1R15A	23645	protein phosphatase 1, regulatory subunit 15A	-3.192283	8.87497E-05	0.006973136
TC17000166.hg.1	MGC12916	84815	uncharacterized protein MGC12916	-3.19527	6.01048E-06	0.001541284
TC06001126.hg.1	SNX9	51429	sorting nexin 9	-3.210849	8.56791E-06	0.001859808
TC15000357.hg.1	MIR147B	100126311	microRNA 147b	-3.221716	1.28566E-05	0.002197629
TC06000099.hg.1	CD83	9308	CD83 molecule	-3.227899	0.000798053	0.02297995
TC21000076.hg.1	MIR155	406947	microRNA 155	-3.234534	0.000804848	0.02307445
TC06000005.hg.1	IRF4	3662	interferon regulatory factor 4	-3.237321	0.000307506	0.01387732
TC05000934.hg.1	LOC257358	257358	uncharacterized LOC257358	-3.363021	1.8438E-07	0.000251226
TC17000117.hg.1	KDM6B	23135	lysine (K)-specific demethylase 6B	-3.394352	1.02422E-05	0.001978921
TC19001457.hg.1	NFKBID	84807	nuclear factor of kappa light polypeptide gene enhancer in B-cells inhibitor, delta	-3.410911	5.07674E-05	0.005038209
TC19001593.hg.1	PLAUR	5329	plasminogen activator, urokinase receptor	-3.439682	0.000739792	0.02210348
TC19002611.hg.1	CD22	933	CD22 molecule	-3.526405	8.44136E-05	0.006789482
TC20000134.hg.1	RIN2	54453	Ras and Rab interactor 2	-3.549743	2.70508E-06	0.001032977
TC09002906.hg.1	ORM2	5005	orosomucoid 2	-3.556606	9.72006E-06	0.001947592
TC19000055.hg.1	GADD45B	4616	growth arrest and DNA-damage-inducible, beta	-3.624548	0.000194344	0.01052681
TC02001202.hg.1	ICOS	29851	inducible T-cell co-stimulator	-3.703925	1.98184E-05	0.002845518
TC04001811.hg.1	LOC731424	731424	uncharacterized LOC731424	-3.834814	0.000212081	0.01117056
TC11000510.hg.1	MS4A1	931	membrane-spanning 4-domains, subfamily A, member 1	-3.862001	0.000436427	0.01631952
TC04001286.hg.1	CXCL2	2920	chemokine (C-X-C motif) ligand 2	-3.889597	1.50362E-06	0.000727875
TC03000056.hg.1	IRAK2	3656	interleukin-1 receptor-associated kinase 2	-3.92782	0.000172031	0.009811233
TC06001510.hg.1	IER3	8870	immediate early response 3	-4.023382	7.46636E-05	0.006235402
TC17000384.hg.1	CCL7	6354	chemokine (C-C motif) ligand 7	-4.05946	0.000232276	0.01182214
TC12000414.hg.1	NR4A1	3164	nuclear receptor subfamily 4, group A, member 1	-4.169702	2.83061E-07	0.000300376
TC05001589.hg.1	LUCAT1	100505994	lung cancer associated transcript 1 (non-protein coding)	-4.194254	5.91084E-05	0.005483618
TC09002907.hg.1	ORM1	5004	orosomucoid 1	-4.197428	3.14995E-06	0.001124588
TC16001335.hg.1	SLC7A5	8140	solute carrier family 7 (amino acid transporter light chain, L system), member 5	-4.367308	5.3584E-05	0.005125277
TC09000508.hg.1	NR4A3	8013	nuclear receptor subfamily 4, group A, member 3	-4.385807	0.000462069	0.01679811
TC10000629.hg.1	LIPN	643418	lipase, family member N	-4.473433	1.38253E-10	1.84742E-06

transcript cluster id	Gene Symbol	Entrez Gene ID	Description	FoldChange (CAEBV/control)	P.Value (CAEBV/control)	adj.P.Val (CAEBV/control)
TC17001466.hg.1	CCR7	1236	chemokine (C-C motif) receptor 7	-4.593782	0.000354316	0.01493103
TC10001421.hg.1	C10orf55	414236	chromosome 10 open reading frame 55	-4.671577	2.35745E-07	0.000297585
TC19000588.hg.1	CD79A	973	CD79a molecule, immunoglobulin-associated alpha	-4.937414	5.21812E-05	0.005099378
TC06000371.hg.1	TNF	7124	tumor necrosis factor	-5.01977	3.91124E-07	0.000300376
TC20000703.hg.1	CD93	22918	CD93 molecule	-5.117226	1.24171E-08	6.19089E-05
TC22000648.hg.1	OSM	5008	oncostatin M	-5.827242	1.16089E-05	0.002116017
TC07000137.hg.1	IL6	3569	interleukin 6 (interferon, beta 2)	-6.027266	0.001763973	0.03644795
TC15001643.hg.1	SEMA7A	8482	semaphorin 7A, GPI membrane anchor (John Milton Hagen blood group)	-6.112475	3.27952E-08	0.000115022
TC15000270.hg.1	THBS1	7057	thrombospondin 1	-6.592058	5.55112E-05	0.005255952
TC12001216.hg.1	OLR1	4973	oxidized low density lipoprotein (lectin-like) receptor 1	-6.64516	7.70916E-06	0.001782452
TC20000341.hg.1	PI3	5266	peptidase inhibitor 3, skin-derived	-7.266821	3.95758E-09	2.49406E-05
TC05002051.hg.1	LOC100128059	100128059	uncharacterized LOC100128059	-7.578386	8.02759E-11	1.84742E-06
TC17000423.hg.1	CCL4L1	9560	chemokine (C-C motif) ligand 4-like 1	-7.653981	9.79897E-06	0.001947592
TC02002218.hg.1	IL1A	3552	interleukin 1, alpha	-8.024831	9.11377E-06	0.001926686
TC17000383.hg.1	CCL2	6347	chemokine (C-C motif) ligand 2	-8.03793	0.002605076	0.04536813
TC17000408.hg.1	CCL4	6351	chemokine (C-C motif) ligand 4	-8.869909	0.000199596	0.01076081
TC17000416.hg.1	CCL4L1	9560	chemokine (C-C motif) ligand 4-like 1	-8.975222	4.09389E-05	0.004476463
TC17001395.hg.1	CCL3L3	414062	chemokine (C-C motif) ligand 3-like 3	-9.657444	0.000791784	0.02287813
TC17001388.hg.1	CCL3L3	414062	chemokine (C-C motif) ligand 3-like 3	-10.100844	0.000628413	0.01988463
TC10000475.hg.1	PLAU	5328	plasminogen activator, urokinase	-11.641376	1.55806E-07	0.000251226
TC17001385.hg.1	CCL3	6348	chemokine (C-C motif) ligand 3	-12.13508	0.000180303	0.01011048

Supplementary Table S4 -303 Immune mediators

Gene Symbol	Entrez Gene ID	Gene Category	Affymetrix HTA2.0 Transcript ID	Fold-Change (AIM/control)	P.Value (AIM/control)	adj.P.Val (AIM/control)	Fold-Change (CAEBV/control)	P.Value (CAEBV/control)	adj.P.Val (CAEBV/control)	Illumina Human Ref8 Probe ID	Fold Change (AIM/Baseline)	P.Value (AIM/Baseline)	adj.P.Value (AIM/Baseline)
CCL1	6346	chemokine (C-C motif)	TC17001360.hg.1	-1.11456	0.014247	0.0468064	1.01954	0.647254	0.833865	ILMN_2086965	1.06922	0.143753	0.219024
CCL2	6347	chemokine (C-C motif)	TC17000383.hg.1	-18.8663	7.38E-05	0.000630777	-8.03793	0.00260508	0.0453681	ILMN_1720048	1.11976	0.595856	0.474281
CCL3	6348	chemokine (C-C motif)	TC17001385.hg.1	-50.1219	5.30E-07	1.39E-05	-12.1351	0.000180303	0.0101105	ILMN_1671509	-1.5738	0.187215	0.253464
CCL3L3	414062	chemokine (C-C motif)	TC17001395.hg.1	-69.8461	4.11E-07	1.16E-05	-9.65744	0.000791784	0.0228781	ILMN_2105573	-1.95413	0.00776452	0.0500219
CCL4	6351	chemokine (C-C motif)	TC17000408.hg.1	-11.672	3.48E-05	0.000346722	-8.86991	0.000199596	0.0107608	no probe			
CCL4L1	9560	chemokine (C-C motif)	TC17000416.hg.1	-20.3623	4.74E-07	1.28E-05	-8.97522	4.09E-05	0.00447646	ILMN_2100209	-1.48177	0.166692	0.2381
CCL5	6352	chemokine (C-C motif)	TC17001378.hg.1	3.11801	5.36E-08	2.74E-06	1.59533	0.00203522	0.0392948	ILMN_2098126	1.43998	0.0504985	0.121607
CCL7	6354	chemokine (C-C motif)	TC17000384.hg.1	-4.81316	4.30E-05	0.000410363	-4.05946	0.000232276	0.0118221	ILMN_1683456	1.01297	0.452597	0.413063
CCL8	6355	chemokine (C-C motif)	TC17000386.hg.1	-1.89652	0.00527548	0.0200589	-1.7283	0.0183289	0.130555	ILMN_1772964	1.12612	0.372303	0.371753
CCL11	6356	chemokine (C-C motif)	TC17000385.hg.1	1.01402	0.734634	0.819658	-1.01175	0.786216	0.905192	ILMN_1725519	1.01808	0.527699	0.446605
CCL13	6357	chemokine (C-C motif)	TC17000387.hg.1	1.02366	0.493372	0.62508	1.03599	0.327021	0.604404	ILMN_1783593	-1.02115	0.420109	0.396827
CCL14	6358	chemokine (C-C motif)	TC17001383.hg.1	-1.04081	0.124299	X	-1.02313	0.390087	X	no probe			
CCL16	6360	chemokine (C-C motif)	TC17001382.hg.1	-1.05587	0.0511082	0.127995	-1.0016	0.953708	0.980178	ILMN_2045324	1.04787	0.413512	0.393859
CCL17	6361	chemokine (C-C motif)	TC16000489.hg.1	-1.04281	0.351265	0.491358	1.00401	0.931482	0.972316	ILMN_1710186	-1.0719	0.147566	0.222524
CCL18	6362	chemokine (C-C motif)	TC17000406.hg.1	-1.18297	0.00095879	0.00552312	-1.1528	0.00502528	0.0684617	ILMN_1654411	1.13786	0.0198805	0.0752172
CCL19	6363	chemokine (C-C motif)	TC09001044.hg.1	-1.05482	0.332984	0.470829	1.03245	0.57692	0.790099	ILMN_1769129	1.00526	0.893548	0.570739
CCL20	6364	chemokine (C-C motif)	TC02001364.hg.1	-5.5251	6.60E-06	9.59E-05	-3.05968	0.000901445	0.0248603	ILMN_1657234	-1.45788	0.068248	0.142772
CCL21	6366	chemokine (C-C motif)	TC09001045.hg.1	1.07769	0.14688	0.262008	1.11392	0.0521396	0.231176	ILMN_1677505	-1.02182	0.433075	0.403525
CCL22	6367	chemokine (C-C motif)	TC16000487.hg.1	-1.09991	0.0753734	X	-1.06813	0.227971	X	ILMN_2160476	1.03562	0.0893103	0.165707
CCL23	6368	chemokine (C-C motif)	TC17001384.hg.1	-1.10921	0.00513327	0.0217515	-1.03184	0.366002	0.638715	ILMN_1764030	1.09237	0.021233	0.0776342
CCL24	6369	chemokine (C-C motif)	TC07001536.hg.1	-1.01392	0.741919	X	1.00907	0.837443	X	ILMN_1653766	-1.07383	0.0506384	0.121845
CCL25	6370	chemokine (C-C motif)	TC19000143.hg.1	-1.04093	0.210719	0.345998	-1.03102	0.358044	0.633053	ILMN_1737817	1.60319	0.00048415	0.0316958
CCL26	10344	chemokine (C-C motif)	TC07001535.hg.1	-1.06458	0.123697	X	-1.02779	0.507462	X	ILMN_1659601	-1.01221	0.793268	0.54209
CCL27	10850	chemokine (C-C motif)	TC09002914.hg.1	1.03273	0.474061	0.606223	1.0039	0.93369	0.973352	ILMN_2113606	-1.08002	0.0551199	0.127227
CCL28	56477	chemokine (C-C motif)	TC05001317.hg.1	1.00004	0.999235	0.999362	1.10053	0.0477631	0.222775	ILMN_2228845	1.06049	0.0113403	0.0581158
CX3CL1	6376	chemokine (C-X3-C motif)	TC16000488.hg.1	-1.10727	0.0134696	0.0451314	-1.06915	0.10217	0.336524	ILMN_1654072	1.05128	0.275509	0.314892
CXCL1	2919	chemokine (C-X-C motif)	TC04000411.hg.1	-4.76509	3.97E-09	4.95E-07	-2.80742	2.28E-06	0.000923417	ILMN_1787897	-1.33503	0.034	0.0986013
CXCL2	2920	chemokine (C-X-C motif)	TC04001286.hg.1	-7.79571	2.47E-09	3.72E-07	-3.8896	1.50E-06	0.000727875	ILMN_1682636	-3.19571	0.00519802	0.0432062
CXCL3	2921	chemokine (C-X-C motif)	TC04001284.hg.1	-1.45576	1.88E-05	0.000223548	-1.37217	0.000197056	0.0109714	ILMN_1709350	1.00965	0.845599	0.557755
CXCL5	6374	chemokine (C-X-C motif)	TC04001283.hg.1	-1.21975	0.000773287	0.00456033	-1.08029	0.146724	0.400756	ILMN_2171384	-1.63907	0.0999851	0.17744
CXCL6	6372	chemokine (C-X-C motif)	TC04000409.hg.1	-1.07829	0.0167077	X	-1.07683	0.0236335	X	ILMN_2161577	-1.0189	0.585068	0.470464
CXCL9	4283	chemokine (C-X-C motif)	TC04001304.hg.1	1.03455	0.859591	0.908104	1.56278	0.0382305	0.19572	ILMN_1745356	2.11086	0.00259655	0.0369974
CXCL10	3627	chemokine (C-X-C motif)	TC04001305.hg.1	-9.26776	0.00903234	0.0307739	-1.24366	0.785889	0.904024	ILMN_1791759	4.09232	0.0048017	0.0423815
CXCL11	6373	chemokine (C-X-C motif)	TC04001306.hg.1	-2.85688	0.0779584	0.161369	-1.39455	0.577886	0.788999	ILMN_2067895	1.01923	0.660216	0.498225
CXCL12	6387	chemokine (C-X-C motif)	TC10001212.hg.1	-1.01883	0.406663	0.557082	-1.02116	0.375304	0.654223	ILMN_1791447	1.04106	0.270483	0.311717
CXCL13	10563	chemokine (C-X-C motif)	TC04000432.hg.1	-1.04521	0.183741	X	-1.03856	0.273821	X	ILMN_1718552	1.07655	0.0724995	0.147488
CXCL14	9547	chemokine (C-X-C motif)	TC05001804.hg.1	-1.023	0.438285	0.578883	-1.02121	0.494192	0.739981	ILMN_1748323	-1.03716	0.540204	0.452533
CXCL16	58191	chemokine (C-X-C motif)	TC17001029.hg.1	-6.43957	1.10E-06	2.40E-05	-1.98783	0.0180144	0.129524	ILMN_1728478	-2.37737	0.0043495	0.0413059
CXCL17	284340	chemokine (C-X-C motif)	TC19001574.hg.1	-1.05661	0.30671	0.444286	-1.00644	0.908022	0.960021	ILMN_1796337	1.01186	0.847726	0.55838
XCL1	6375	chemokine (C motif)	TC01001469.hg.1	-1.67334	0.00934991	0.0316963	-1.56679	0.0260033	0.158562	ILMN_2224955	-1.10699	0.00378422	0.0404966
XCL2	6846	chemokine (C motif)	TC01003490.hg.1	-1.65491	0.0189435	0.0554391	-1.84546	0.00796594	0.0845416	no probe			
CCR1	1230	chemokine (C-C motif) receptor	TC03001355.hg.1	-2.89502	0.00187507	0.00876932	-1.39289	0.287921	0.559776	ILMN_1678833	-1.25907	0.283944	0.320772
CCR2	729230	chemokine (C-C motif) receptor	TC03000255.hg.1	1.26245	0.0212113	0.061019	1.17565	0.110712	0.34494	no probe			
CCR3	1232	chemokine (C-C motif) receptor	TC03000254.hg.1	-1.32482	0.00194458	0.00918349	-1.19508	0.0406045	0.203462	ILMN_1763322	-1.09226	0.0782153	0.153462
CCR4	1233	chemokine (C-C motif) receptor	TC03000164.hg.1	-2.26173	8.13E-05	0.000685794	-1.60494	0.0109293	0.100254	ILMN_2086143	-1.22034	0.00514028	0.043159
CCR5	1234	chemokine (C-C motif) receptor	TC03000256.hg.1	1.52364	2.27E-06	4.39E-05	1.08518	0.20963	0.480307	ILMN_2145033	1.41426	0.00730226	0.0487774
CCR6	1235	chemokine (C-C motif) receptor	TC06004106.hg.1	-1.41459	8.00E-08	4.13E-06	-1.36608	6.42E-07	0.000468492	ILMN_2387696	-1.06861	0.0205847	0.0764902
CCR7	1236	chemokine (C-C motif) receptor	TC17001466.hg.1	-5.23309	9.77E-05	0.000791118	-4.59378	0.000354316	0.014931	ILMN_1715131	-2.33353	0.00803014	0.0506991
CCR8	1237	chemokine (C-C motif) receptor	TC03000200.hg.1	-1.03948	0.202534	X	-1.01858	0.555523	X	ILMN_1739421	-1.07585	0.0752848	0.150529
CCR9	10803	chemokine (C-C motif) receptor	TC03000251.hg.1	-1.24536	0.0016346	0.00815449	-1.238	0.00292612	0.0489089	ILMN_2337386	-1.14767	0.0587421	0.131756

Gene Symbol	Entrez Gene ID	Gene Category	Affymetrix HTA2.0 Transcript ID	Fold-Change (AIM/control)	P.Value (AIM/control)	adj.P.Val (AIM/control)	Fold-Change (CAEBV/control)	P.Value (CAEBV/control)	adj.P.Val (CAEBV/control)	Illumina Human Ref8 Probe ID	Fold Change (AIM/Baseline)	P.Value (AIM/Baseline)	adj.P.Value (AIM/Baseline)
CCR10	2826	chemokine (C-C motif) receptor	TC17001539.hg.1	-1.03525	0.278165	0.419513	-1.01653	0.61958	0.819886	ILMN_1666493	-1.22534	0.062913	0.136805
CCRL1	51554	chemokine (C-C motif) receptor	TC03000716.hg.1	-1.0263	0.33746	X	-1.00957	0.734008	X	ILMN_1740436	1.0208	0.563326	0.461701
CCRL2	9034	chemokine (C-C motif) receptor	TC03000257.hg.1	-5.98267	6.59E-09	6.64E-07	-3.18083	4.60E-06	0.00129196	no probe			
CX3CR1	1524	chemokine (C-X3-C motif) receptor	TC03001304.hg.1	5.66496	1.99E-05	0.000223728	1.74286	0.0902206	0.305614	ILMN_2088437	1.57984	0.0440266	0.112986
CXCR1	3577	chemokine (C-X-C motif) receptor	TC02002767.hg.1	-2.65493	0.0164599	0.0496062	1.39159	0.401384	0.661132	ILMN_1662524	-1.0208	0.692575	0.50836
CXCR2	3579	chemokine (C-X-C motif) receptor	TC02001292.hg.1	-5.53402	6.00E-05	0.000537736	-1.42898	0.303741	0.575995	ILMN_1680397	-1.38686	0.00653962	0.0470596
CXCR3	2833	chemokine (C-X-C motif) receptor	TC0X001128.hg.1	1.19292	0.0400399	0.0988963	-1.14887	0.113413	0.34952	ILMN_1797975	1.91297	0.0214742	0.0778566
CXCR4	7852	chemokine (C-X-C motif) receptor	TC02002378.hg.1	-2.78213	0.000620946	0.00359358	-1.45606	0.159152	0.41422	ILMN_1801584	-2.68737	0.00657566	0.047171
CXCR5	643	chemokine (C-X-C motif) receptor	TC11001073.hg.1	-1.89813	1.52E-06	3.13E-05	-1.86633	3.87E-06	0.00121296	ILMN_2337928	-2.6026	0.000895186	0.0335386
CXCR6	10663	chemokine (C-X-C motif) receptor	TC03000253.hg.1	1.25941	0.00316869	0.0136154	-1.05678	0.442169	0.69534	ILMN_1674640	1.82379	0.0477591	0.117832
CXCR7	57007	chemokine (C-X-C motif) receptor	TC02001437.hg.1	-1.10726	0.00192236	0.0104273	-1.09422	0.00663503	0.0834277	ILMN_1798360	-2.08187	0.000145378	0.0255956
XCR1	2829	chemokine (C motif) receptor	TC03001354.hg.1	-1.10139	0.00374691	0.0173675	-1.01367	0.657315	0.840395	ILMN_1773238	1.05613	0.133165	0.209885
IL1A	3552	Interleukin	TC02002218.hg.1	-13.8545	2.54E-07	8.29E-06	-8.02483	9.11E-06	0.00192669	ILMN_1658483	-1.29336	0.107821	0.185462
IL1B	3553	Interleukin	TC02002219.hg.1	-34.5729	4.52E-06	7.10E-05	-5.1811	0.00906921	0.09169	ILMN_1775501	-5.06514	0.00637091	0.0464817
IL2	3558	Interleukin	TC04001520.hg.1	-1.04502	0.149814	X	-1.03086	0.334479	X	ILMN_1704321	-1.03186	0.209697	0.270367
IL4	3565	Interleukin	TC05000641.hg.1	-1.10453	0.00376682	0.017307	-1.07776	0.0274048	0.173013	ILMN_1669174	-1.03823	0.244455	0.294583
IL5	3567	Interleukin	TC05001769.hg.1	1.00985	0.703106	0.799714	-1.019	0.487399	0.737292	ILMN_2207190	1.06359	0.0862875	0.162563
IL6	3569	Interleukin	TC07000137.hg.1	-24.0256	2.94E-06	5.07E-05	-6.02727	0.00176397	0.036448	ILMN_1699651	-1.51288	0.0238212	0.081886
IL7	3574	Interleukin	TC08001350.hg.1	-1.11404	0.000110371	0.00123572	-1.06069	0.0182194	0.146248	ILMN_2059744	-1.06106	0.301932	0.331516
IL8	3576	Interleukin	TC04000408.hg.1	-30.3605	5.69E-07	1.46E-05	-2.77269	0.0394181	0.198849	ILMN_2184373	-5.78942	0.00420488	0.0413059
IL9	3578	Interleukin	TC05001807.hg.1	-1.04211	0.249611	0.386845	-1.05336	0.170233	0.436307	ILMN_2151676	-1.02088	0.587149	0.4711
IL10	3586	Interleukin	TC01003766.hg.1	-1.26327	0.006356	0.0237517	-1.30214	0.00380475	0.0565632	ILMN_2073307	-1.39462	0.0115749	0.0585989
IL11	3589	Interleukin	TC19001870.hg.1	-1.0935	0.101032	0.199356	-1.08058	0.169758	0.430798	ILMN_1788107	1.05137	0.127864	0.205186
IL12A	3592	Interleukin	TC03000870.hg.1	-1.04508	0.159835	0.285641	-1.1083	0.00461994	0.0679861	ILMN_1671353	1.37915	0.028866	0.0903333
IL12B	3593	Interleukin	TC05001997.hg.1	-1.15069	0.00887642	0.0316634	-1.10317	0.0648764	0.260774	ILMN_1681132	1.06337	0.218957	0.277214
IL13	3596	Interleukin	TC05000640.hg.1	-1.03852	0.424796	0.560957	-1.07211	0.169221	0.431034	ILMN_2052511	-1.01338	0.862891	0.562913
IL15	3600	Interleukin	TC04000693.hg.1	-1.07639	0.0831496	0.174789	1.12536	0.0120106	0.109341	ILMN_2369221	1.19785	0.111805	0.189723
IL16	3603	Interleukin	TC15000756.hg.1	1.13278	0.0712794	0.152494	-1.05731	0.42304	0.679549	ILMN_1813572	-1.28248	0.00433632	0.0413059
IL17A	3605	Interleukin	TC06000652.hg.1	-1.0347	0.44486	0.579356	-1.07659	0.125284	0.371285	ILMN_1774983	-1.04288	0.180618	0.248696
IL17B	27190	Interleukin	TC05001922.hg.1	-1.06092	0.107782	0.212695	1.01874	0.617568	0.817069	ILMN_1766707	-1.04488	0.287201	0.322629
IL17C	27189	Interleukin	TC16000681.hg.1	-1.20663	0.00375643	0.0157553	-1.20082	0.00625772	0.0755625	ILMN_1788109	-1.10085	0.170263	0.240981
IL17D	53342	Interleukin	TC13000027.hg.1	-1.08003	0.0641599	X	-1.12816	0.0089327	X	ILMN_1753823	-1.06311	0.0246537	0.0830656
IL17F	112744	Interleukin	TC06001798.hg.1	-1.10096	0.0300299	0.0825668	-1.10218	0.0355221	0.193777	ILMN_2188247	1.04623	0.13919	0.215262
IL18	3606	Interleukin	TC11002293.hg.1	-1.59944	0.0425889	0.102597	1.25747	0.321556	0.592648	ILMN_1778457	-1.39317	0.000602713	0.0335386
IL19	29949	Interleukin	TC01001728.hg.1	-1.06997	0.0201384	X	-1.01362	0.63031	X	ILMN_1682592	1.02929	0.561038	0.460758
IL20	50604	Interleukin	TC01001729.hg.1	-1.01745	0.635349	0.743403	1.02535	0.514242	0.750283	no probe			
IL21	59067	Interleukin	TC04001521.hg.1	-1.03008	0.52471	X	-1.01611	0.742426	X	ILMN_1772674	-1.00023	0.994629	0.596983
IL22	50616	Interleukin	TC12001698.hg.1	-1.02061	0.421886	0.567353	-1.01099	0.679317	0.853015	ILMN_2100046	-1.01929	0.465294	0.418749
IL23A	51561	Interleukin	TC12000512.hg.1	-1.85805	2.57E-09	4.02E-07	-1.59662	2.72E-07	0.000300376	ILMN_1715603	-1.7428	0.000261761	0.027705
IL24	11009	Interleukin	TC01001730.hg.1	-1.02776	0.455034	X	-1.01792	0.64223	X	ILMN_2407799	-1.03186	0.189509	0.255462
IL25	64806	Interleukin	TC14000145.hg.1	-1.04211	0.296119	X	-1.00011	0.997844	X	ILMN_2401883	1.04681	0.375151	0.373178
IL26	55801	Interleukin	TC12001697.hg.1	-1.02497	0.401174	X	-1.02921	0.351357	X	ILMN_2123182	-1.03687	0.113888	0.191663
IL27	246778	Interleukin	TC16000988.hg.1	-1.0548	0.449184	0.580907	1.00278	0.969741	0.987539	ILMN_1753758	1.32982	0.0254712	0.0845193
IL3	3562	Interleukin	TC05000628.hg.1	-1.08231	0.0319453	0.0879237	-1.0593	0.122691	0.370921	ILMN_1766320	-1.03065	0.595236	0.474019
IL31	386653	Interleukin	TC12002070.hg.1	-1.00157	0.955607	X	-1.09697	0.00553101	X	ILMN_2201866	1.01224	0.696745	0.509687
IL32	9235	Interleukin	TC16000099.hg.1	3.27491	1.05E-07	4.42E-06	1.49078	0.0111787	0.101595	ILMN_1778010	2.38187	0.00462773	0.0420583
IL33	90865	Interleukin	TC09000045.hg.1	1.01098	0.676744	X	-1.00386	0.88811	X	ILMN_2052924	-1.03304	0.379772	0.375956
IL34	146433	Interleukin	TC16000582.hg.1	-1.05367	0.110282	0.217743	-1.03053	0.368	0.640781	ILMN_1713686	1.02921	0.389317	0.381028
IL36A	27179	Interleukin	TC02000716.hg.1	1.00671	0.856828	0.907866	-1.03583	0.370632	0.640781	ILMN_1704000	1.0312	0.454637	0.41346
IL36B	27177	Interleukin	TC02002220.hg.1	-1.00239	0.932021	X	-1.01577	0.595972	X	ILMN_1754002	-1.0202	0.377205	0.37437
IL36G	56300	Interleukin	TC02000715.hg.1	-1.10421	0.035859	0.0935505	-1.06599	0.17874	0.444065	ILMN_2158713	-1.01518	0.644032	0.492853
IL37	27178	Interleukin	TC02000714.hg.1	-1.02967	0.352503	X	-1.03252	0.33246	X	ILMN_1697710	1.71369	0.00495007	0.0427064

Gene Symbol	Entrez Gene ID	Gene Category	Affymetrix HTA2.0 Transcript ID	Fold-Change (AIM/control)	P.Value (AIM/control)	adj.P.Val (AIM/control)	Fold-Change (CAEBV/control)	P.Value (CAEBV/control)	adj.P.Val (CAEBV/control)	Illumina Human Ref8 Probe ID	Fold Change (AIM/Baseline)	P.Value (AIM/Baseline)	adj.P.Value (AIM/Baseline)
IL1R1	3554	Interleukine receptor	TC02000620.hg.1	-3.45565	4.35E-08	2.35E-06	-2.64727	2.12E-06	0.000889419	ILMN_1810584	-1.05149	0.153783	0.227483
IL1R2	7850	Interleukine receptor	TC02000619.hg.1	-2.79776	7.47E-06	0.000105745	-1.64499	0.00908269	0.0919899	ILMN_1772131	-1.52307	0.00216445	0.035711
IL1RN	3557	Interleukine receptor	TC02000720.hg.1	-7.88217	2.34E-08	1.49E-06	-3.01423	0.000108813	0.00751306	ILMN_1774874	-1.53489	0.0867518	0.163083
IL2RA	3559	Interleukine receptor	TC10001007.hg.1	-3.42841	4.26E-07	1.20E-05	-2.75096	9.37E-06	0.00194191	ILMN_2108699	-1.08258	0.117505	0.195384
IL2RB	3560	Interleukine receptor	TC22000722.hg.1	1.38601	0.0404019	0.0988433	-1.33558	0.0778888	0.282712	ILMN_1684349	1.15906	0.409996	0.391934
IL2RG	3561	Interleukine receptor	TC0X001121.hg.1	1.89233	0.000210598	0.00149183	1.17743	0.265101	0.536501	ILMN_1794386	1.30378	0.00747401	0.0494004
IL3RA	3563	Interleukine receptor	TC0X000008.hg.1	-2.0703	9.03E-08	4.07E-06	-1.7148	8.21E-06	0.00185981	ILMN_1747344	-1.1004	0.349632	0.359731
IL4R	3566	Interleukine receptor	TC16000293.hg.1	-2.71117	7.40E-09	7.34E-07	-1.85558	8.50E-06	0.00185981	ILMN_1652185	-1.84469	0.00472119	0.0421516
IL5RA	3568	Interleukine receptor	TC03001122.hg.1	-1.08185	0.0846464	0.176468	-1.00048	0.991695	0.996151	ILMN_1756455	-1.07822	0.105088	0.182784
IL6R	3570	Interleukine receptor	TC01001276.hg.1	-1.60335	0.000312123	0.00206487	-1.07925	0.4944	0.733299	ILMN_1754753	-1.76287	0.0146698	0.0655564
IL7R	3575	Interleukine receptor	TC05000159.hg.1	-1.35877	0.162548	0.278642	1.05049	0.825547	0.924582	ILMN_2342579	-2.13759	0.0100415	0.0555017
IL9R	3581	Interleukine receptor	TC0X002325.hg.1	-1.07226	0.039553	0.103327	-1.03974	0.250765	0.531012	ILMN_1794686	-1.09379	0.0418737	0.109926
IL10RA	3587	Interleukine receptor	TC11001058.hg.1	-1.34349	0.00192247	0.0090895	-1.23389	0.023315	0.149985	ILMN_1652825	-1.11154	0.469993	0.420701
IL10RB	3588	Interleukine receptor	TC21001059.hg.1	1.02355	0.822834	0.882001	1.14393	0.227705	0.497269	ILMN_2230892	-1.22764	0.207431	0.268621
IL11RA	3590	Interleukine receptor	TC09002897.hg.1	-1.05764	0.18887	0.315793	1.0062	0.887113	0.952232	ILMN_1653459	-1.11438	0.000356712	0.0297928
IL12RB1	3594	Interleukine receptor	TC19001281.hg.1	1.80077	3.24E-08	1.97E-06	1.1483	0.04504	0.21516	ILMN_1815890	1.71252	0.00248163	0.0365661
IL12RB2	3595	Interleukine receptor	TC01000741.hg.1	1.50605	0.104141	0.200577	2.31445	0.00383352	0.0561595	ILMN_1761921	1.47225	0.0351898	0.100347
IL13RA1	3597	Interleukine receptor	TC0X000575.hg.1	-2.60571	0.000763445	0.00426665	1.34245	0.244704	0.513795	ILMN_1768505	-2.50191	0.0131108	0.062326
IL13RA2	3598	Interleukine receptor	TC0X001290.hg.1	1.00164	0.951	X	-1.01967	0.489909	X	ILMN_1688722	-1.02818	0.455535	0.413913
IL15RA	3601	Interleukine receptor	TC10001006.hg.1	-1.16026	0.0446606	0.10763	-1.06546	0.389509	0.652593	ILMN_1665682	1.46625	0.00130373	0.0335386
IL17RA	23765	Interleukine receptor	TC22000019.hg.1	1.06255	0.737142	0.819376	1.45019	0.0629125	0.252248	ILMN_1728724	-1.39356	0.142612	0.217858
IL17RB	55540	Interleukine receptor	TC03000352.hg.1	-1.01372	0.585131	X	-1.04137	0.133228	X	ILMN_1767523	-1.3167	0.00695842	0.0482348
IL17RC	84818	Interleukine receptor	TC03000047.hg.1	-1.05749	0.0337268	0.0963556	-1.01762	0.499804	0.745828	ILMN_1666226	-1.03054	0.361617	0.366182
IL17RD	54756	Interleukine receptor	TC03001489.hg.1	-1.01029	0.654545	X	1.00037	0.987692	X	ILMN_2407851	-1.32188	0.0016923	0.0340315
IL17RE	132014	Interleukine receptor	TC03000046.hg.1	-1.06217	0.0481522	X	-1.02854	0.356068	X	ILMN_1752089	-1.08559	0.0226348	0.079607
IL18R1	8809	Interleukine receptor	TC02004995.hg.1	1.13042	0.474317	0.602925	1.8672	0.00252179	0.0447392	ILMN_1781700	1.06401	0.58804	0.47139
IL20RA	53832	Interleukine receptor	TC06002150.hg.1	-1.05517	0.0618814	X	-1.02683	0.359957	X	ILMN_1695107	1.018	0.646536	0.49373
IL20RB	53833	Interleukine receptor	TC03003324.hg.1	-1.0738	0.0385344	0.101337	1.07773	0.0381467	0.204092	ILMN_1765668	1.03871	0.463744	0.418144
IL21R	50615	Interleukine receptor	TC16000294.hg.1	-1.65114	0.00388916	0.0157026	-2.05799	0.000271979	0.0128382	ILMN_1798204	1.29075	0.0811385	0.156499
IL22RA1	58985	Interleukine receptor	TC01002358.hg.1	-1.06505	0.173648	X	-1.10836	0.0412685	X	ILMN_1666175	1.00914	0.798953	0.543923
IL22RA2	116379	Interleukine receptor	TC06002151.hg.1	-1.02614	0.359075	X	-1.01283	0.662422	X	ILMN_2307721	1.03662	0.442319	0.408386
IL23R	149233	Interleukine receptor	TC01000739.hg.1	-1.0355	0.139546	X	1.02116	0.387137	X	ILMN_2223663	-1.19474	0.0514253	0.122747
IL27RA	9466	Interleukine receptor	TC19000250.hg.1	1.41504	0.00227596	0.010325	-1.10834	0.323298	0.594635	ILMN_1688152	-1.09225	0.152225	0.226318
IL31RA	133396	Interleukine receptor	TC05000238.hg.1	1.00024	0.99236	0.995142	1.10942	0.00104513	0.0317956	ILMN_1667575	-1.02624	0.532644	0.448927
IL36RN	26525	Interleukine receptor	TC02000717.hg.1	-1.01783	0.593684	X	-1.04284	0.235624	X	ILMN_1759141	1.06956	0.00371519	0.0404195
IFNA1	3439	interferon	TC09000103.hg.1	-1.04759	0.348728	0.487727	-1.12526	0.0324926	0.183494	ILMN_1688663	-1.01672	0.672814	0.501646
IFNA2	3440	interferon	TC09000960.hg.1	-1.00198	0.958735	X	1.04429	0.287024	X	ILMN_1698186	1.02602	0.227675	0.282866
IFNA4	3441	interferon	TC09000950.hg.1	1.01032	0.776787	X	-1.00714	0.851181	X	ILMN_2202096	1.05296	0.163387	0.235449
IFNA5	3442	interferon	TC09000956.hg.1	-1.00043	0.98799	0.992449	-1.00671	0.825303	0.925435	ILMN_1660195	1.05319	0.326563	0.346022
IFNA6	3443	interferon	TC09000958.hg.1	1.01332	0.657816	X	1.00242	0.938211	X	ILMN_1684538	-1.02917	0.406543	0.390157
IFNA7	3444	interferon	TC09002913.hg.1	-1.00621	0.902565	0.937207	1.00793	0.881549	0.950105	ILMN_2186745	1.06693	0.166952	0.238295
IFNA8	3445	interferon	TC09000102.hg.1	1.05237	0.0768352	0.171041	-1.01324	0.648847	0.836453	ILMN_2110561	-1.03998	0.56418	0.462046
IFNA10	3446	interferon	TC09000952.hg.1	1.01407	0.664241	0.767036	1.02791	0.418148	0.681209	ILMN_1769988	1.16035	0.131142	0.208148
IFNA13	3447	interferon	TC09000959.hg.1	-1.09158	0.0292536	X	-1.05563	0.178241	X	ILMN_2193942	1.01461	0.659014	0.497867
IFNA14	3448	interferon	TC09002912.hg.1	-1.04669	0.196295	X	-1.08077	0.0435557	X	ILMN_2129202	-1.00996	0.766826	0.533622
IFNA16	3449	interferon	TC09000953.hg.1	-1.04079	0.395679	0.533404	-1.04317	0.392038	0.656188	ILMN_1733136	-1.0235	0.698476	0.51008
IFNA17	3451	interferon	TC09000954.hg.1	-1.00396	0.901602	0.937292	-1.03084	0.370533	0.642748	ILMN_1652376	-1.00786	0.714438	0.51586
IFNA21	3452	interferon	TC09000949.hg.1	1.01027	0.763511	X	1.02583	0.476855	X	ILMN_1675192	1.00006	0.998632	0.598035
IFNB1	3456	interferon	TC09000946.hg.1	-1.25855	0.040752	0.0998167	-1.14013	0.244476	0.514494	ILMN_1682245	1.13476	0.00662001	0.047257
IFNE	338376	interferon	TC09000962.hg.1	-1.01409	0.753644	X	-1.05603	0.252765	X	ILMN_2097997	1.01102	0.710296	0.514412
IFNG	3458	interferon	TC12001696.hg.1	-1.75544	0.233129	0.360787	-1.98262	0.169857	0.427382	ILMN_2207291	5.38507	0.00124284	0.0335386
IFNK	56832	interferon	TC09000125.hg.1	-1.02028	0.668298	0.767756	-1.01164	0.813508	0.919439	ILMN_1676704	1.07699	0.125844	0.203144

Gene Symbol	Entrez Gene ID	Gene Category	Affymetrix HTA2.0 Transcript ID	Fold-Change (AIM/control)	P.Value (AIM/control)	adj.P.Val (AIM/control)	Fold-Change (CAEBV/control)	P.Value (CAEBV/control)	adj.P.Val (CAEBV/control)	Illumina Human Ref8 Probe ID	Fold Change (AIM/Baseline)	P.Value (AIM/Baseline)	adj.P.Value (AIM/Baseline)
IFNL1	282618	interferon	TC19000540.hg.1	-1.02701	0.622673	0.731733	-1.00931	0.869981	0.945454	ILMN_2149624	-1.1995	0.0437175	0.112552
IFNL2	282616	interferon	TC19000539.hg.1	-1.08305	0.116058	0.22036	-1.08174	0.138297	0.388437	ILMN_1662302	1.00542	0.907827	0.574659
IFNL3	282617	interferon	TC19001514.hg.1	-1.13732	0.00973351	0.0342422	-1.14467	0.00966399	0.0971788	ILMN_1768900	1.00207	0.936182	0.581936
IFNW1	3467	interferon	TC09000947.hg.1	-1.01157	0.753862	X	-1.02054	0.598317	X	ILMN_2176225	1.0581	0.187985	0.254156
IFNAR1	3454	interferon receptor	TC21000128.hg.1	-1.15815	0.221092	0.348613	1.19934	0.152193	0.404749	ILMN_1752923	-1.30561	0.0354371	0.100774
IFNAR2	3455	interferon receptor	TC21001060.hg.1	-1.14148	0.0625094	0.138374	1.09427	0.212581	0.48276	ILMN_1791057	-1.49498	0.0318823	0.0948585
IFNGR1	3459	interferon receptor	TC06002152.hg.1	-2.8264	6.93E-05	0.000600904	-1.00982	0.962907	0.983723	ILMN_1675939	-1.79294	0.00275397	0.0376125
IFNGR2	3460	interferon receptor	TC21000129.hg.1	-4.01578	7.65E-07	1.82E-05	-1.33943	0.142816	0.390715	ILMN_1764964	-1.61475	0.0427254	0.111326
IFNLR1	163702	interferon receptor	TC01002359.hg.1	1.1121	0.0109486	0.0383044	-1.08652	0.0481812	0.225344	ILMN_2405324	2.01504	0.0178869	0.0717625
IRF1	3659	interferon regulatory factor	TC05001767.hg.1	-1.38484	0.081411	0.166987	1.12807	0.521541	0.751328	ILMN_1708375	1.55264	0.00292337	0.03842
IRF2	3660	interferon regulatory factor	TC04001805.hg.1	1.77609	8.01E-05	0.000682497	1.61552	0.000730713	0.0221035	ILMN_2090607	1.1539	0.0296857	0.0915847
IRF3	3661	interferon regulatory factor	TC19001722.hg.1	1.31736	1.25E-06	3.02E-05	1.0516	0.21596	0.491152	ILMN_1765649	-1.03699	0.488466	0.42892
IRF4	3662	interferon regulatory factor	TC06000005.hg.1	-2.14882	0.00663407	0.0241223	-3.23732	0.000307506	0.0138773	ILMN_1754507	1.09006	0.462709	0.417541
IRF5	3663	interferon regulatory factor	TC07000789.hg.1	-1.09007	0.0641631	0.143618	-1.03465	0.465552	0.716341	ILMN_2312606	-1.75523	0.0479417	0.118105
IRF6	3664	interferon regulatory factor	TC01003787.hg.1	-1.02142	0.435687	X	-1.00604	0.831303	X	ILMN_2072357	1.03302	0.476306	0.423542
IRF7	3665	interferon regulatory factor	TC11001241.hg.1	-1.34327	0.0398417	0.0979943	-1.07476	0.610133	0.809535	ILMN_2349061	1.31581	0.148097	0.223003
IRF8	3394	interferon regulatory factor	TC16000666.hg.1	-2.61037	6.65E-09	6.76E-07	-1.86028	4.63E-06	0.00129196	ILMN_1666594	-1.62769	0.0473784	0.117409
IRF9	10379	interferon regulatory factor	TC14000161.hg.1	1.00259	0.967927	0.978712	-1.00842	0.901024	0.957314	ILMN_1745471	1.23925	0.00662029	0.047257
FCAMR	83953	Fc receptor	TC01003769.hg.1	-1.0046	0.859968	0.911438	1.03578	0.208094	0.489823	ILMN_2206009	1.05883	0.0598516	0.132903
FCAR	2204	Fc receptor	TC19000885.hg.1	-8.18753	3.81E-07	1.09E-05	-1.92542	0.0282567	0.16497	ILMN_2279367	-1.43326	0.00313114	0.0391567
FCER1A	2205	Fc receptor	TC01001351.hg.1	-1.49416	0.0082848	0.0288646	-1.58576	0.00453331	0.0615112	no probe			
FCER1G	2207	Fc receptor	TC01001382.hg.1	-1.89454	0.000803028	0.00444868	1.24293	0.202429	0.468748	ILMN_2123743	-1.67161	0.0655516	0.139889
FCER2	2208	Fc receptor	TC19001116.hg.1	-1.98831	4.52E-06	7.22E-05	-2.23014	1.18E-06	0.000652633	ILMN_1662451	-1.98297	0.00439646	0.0414531
FCGR1A	2209	Fc receptor	TC01001172.hg.1	1.48547	0.214577	0.340508	5.40383	7.19E-05	0.00617847	ILMN_2176063	2.18352	0.018093	0.0720577
FCGR1B	2210	Fc receptor	TC01003057.hg.1	1.60894	0.174631	0.293353	5.91156	0.000106639	0.00743584	ILMN_2261600	2.63013	0.0185349	0.0729705
FCGR2A	2212	Fc receptor	TC01001399.hg.1	-2.25007	0.000561428	0.00331636	1.20593	0.361971	0.628573	ILMN_2289849	-1.72484	0.0236736	0.0816754
FCGR2B	2213	Fc receptor	TC01006338.hg.1	-1.48586	0.000903515	0.00495559	-1.21388	0.0774013	0.2827	ILMN_2382403	-1.3057	0.039985	0.107309
FCGR3A	2214	Fc receptor	TC01006307.hg.1	-1.13047	0.495397	0.621069	1.16945	0.408323	0.666991	ILMN_2112580	1.05075	0.548404	0.455658
FCGR3B	2215	Fc receptor	TC01006308.hg.1	-2.31779	0.015127	0.0464433	1.09816	0.777057	0.899657	ILMN_2134453	-1.00486	0.922399	0.57819
ITGA1	3672	integrin	TC05003439.hg.1	1.37005	0.0600608	0.13289	1.47594	0.0296685	0.170482	ILMN_1802411	1.3382	0.042218	0.110505
ITGA2	3673	integrin	TC05000218.hg.1	-1.0114	0.595235	X	-1.00821	0.714345	X	ILMN_1665792	1.02117	0.661131	0.498557
ITGA2B	3674	integrin	TC17001581.hg.1	-1.0785	0.466661	0.595993	1.25812	0.0458898	0.215745	ILMN_1721888	-1.53333	0.169611	0.240499
ITGA3	3675	integrin	TC17000654.hg.1	-1.04971	0.0706596	0.162407	-1.04364	0.123744	0.379891	ILMN_1685397	-1.16503	0.113292	0.19113
ITGA4	3676	integrin	TC02001083.hg.1	2.20229	9.62E-05	0.000786174	1.78677	0.00240893	0.0435037	ILMN_1747052	1.28578	0.0889149	0.165224
ITGA5	3678	integrin	TC12001571.hg.1	-1.84017	5.86E-05	0.000533728	-1.19434	0.156427	0.411173	ILMN_1792679	-1.47415	0.0194088	0.0742548
ITGA6	3655	integrin	TC02001030.hg.1	-1.02849	0.902886	0.937075	1.39348	0.181326	0.442902	ILMN_2359287	-1.55128	0.00857883	0.0523212
ITGA7	3679	integrin	TC12001583.hg.1	-1.0347	0.347798	0.490216	-1.00138	0.970797	0.988058	ILMN_1791409	1.00774	0.855271	0.560481
ITGA8	8516	integrin	TC10001064.hg.1	-1.0079	0.740871	X	-1.0222	0.383479	X	ILMN_1660306	-1.01631	0.60648	0.478405
ITGA9	3680	integrin	TC03000181.hg.1	-1.01709	0.532697	0.663589	-1.01454	0.611709	0.815942	no probe			
ITGA10	8515	integrin	TC01001095.hg.1	-1.02518	0.326986	X	-1.00278	0.915838	X	ILMN_1700144	-1.17447	0.0498306	0.120489
ITGA11	22801	integrin	TC15001593.hg.1	-1.04186	0.147697	X	-1.01307	0.652537	X	ILMN_2406084	1.01604	0.650336	0.494916
ITGAD	3681	integrin	TC16000376.hg.1	-1.16964	0.000219919	0.00175909	-1.09515	0.0189295	0.14014	ILMN_1681945	-1.03869	0.387722	0.380091
ITGAE	3682	integrin	TC17001011.hg.1	-1.02027	0.449764	0.591881	-1.06153	0.0426416	0.219274	ILMN_1683927	1.03445	0.677722	0.503403
ITGAL	3683	integrin	TC16000349.hg.1	2.68525	3.14E-06	5.35E-05	1.36459	0.0552033	0.235014	ILMN_1749591	1.23036	0.0028512	0.0379471
ITGAM	3684	integrin	TC16000374.hg.1	-1.3315	0.102104	0.197974	1.09922	0.592856	0.7992	ILMN_1685009	-1.6647	0.00289819	0.0382259
ITGAV	3685	integrin	TC02001100.hg.1	-1.1209	0.0304046	0.0822796	-1.06403	0.236831	0.509274	ILMN_2169439	-1.08255	0.18766	0.253809
ITGAX	3687	integrin	TC16000375.hg.1	-2.69446	6.45E-06	9.48E-05	-1.58855	0.01048	0.0981768	ILMN_2254635	-1.62218	0.0214717	0.0778566
ITGB1	3688	integrin	TC10001163.hg.1	1.36537	0.0326216	0.0845319	1.23084	0.156894	0.411343	ILMN_1723467	-1.2328	0.0171556	0.0702807
ITGB2	3689	integrin	TC21000538.hg.1	1.37473	0.00118821	0.00620554	1.0752	0.408184	0.667557	ILMN_2175912	1.13752	0.00154671	0.0335386
ITGB3	3690	integrin	TC17002878.hg.1	-1.29135	0.277521	0.408425	1.86737	0.0183993	0.130869	ILMN_1733324	-1.49347	0.154978	0.228492
ITGB4	3691	integrin	TC17000861.hg.1	-1.0601	0.0531608	0.130178	-1.03971	0.203257	0.482136	ILMN_2253707	1.05632	0.101027	0.178496
ITGB5	3693	integrin	TC03001717.hg.1	-1.0614	0.259054	0.392677	1.09793	0.0991393	0.327526	ILMN_1668374	-2.30212	0.0209767	0.0771387

Gene Symbol	Entrez Gene ID	Gene Category	Affymetrix HTA2.0 Transcript ID	Fold-Change (AIM/control)	P.Value (AIM/control)	adj.P.Val (AIM/control)	Fold-Change (CAEBV/control)	P.Value (CAEBV/control)	adj.P.Val (CAEBV/control)	Illumina Human Ref8 Probe ID	Fold Change (AIM/Baseline)	P.Value (AIM/Baseline)	adj.P.Value (AIM/Baseline)
ITGB6	3694	integrin	TC02002469.hg.1	-1.01153	0.567107	X	-1.00079	0.969958	X	ILMN_1789846	1.01445	0.678442	0.503597
ITGB7	3695	integrin	TC12001544.hg.1	2.09303	0.000114116	0.000905648	1.3406	0.0750869	0.277624	ILMN_1777519	1.20205	0.290928	0.325025
ITGB8	3696	integrin	TC07000125.hg.1	-1.763	1.98E-08	1.41E-06	-1.67436	1.47E-07	0.000251226	ILMN_1732921	-1.06956	0.0952422	0.172505
TLR1	7096	toll-like receptor	TC04002916.hg.1	-1.51751	0.0615805	0.135184	1.32006	0.220625	0.489762	ILMN_1731048	-1.1177	0.567216	0.463342
TLR2	7097	toll-like receptor	TC04000775.hg.1	-5.22241	5.83E-06	8.67E-05	1.13905	0.629898	0.821507	ILMN_1772387	-1.36161	0.0475941	0.117645
TLR3	7098	toll-like receptor	TC04000912.hg.1	1.00773	0.953693	0.96963	1.27592	0.093731	0.312902	ILMN_2155708	1.05575	0.22768	0.282866
TLR4	7099	toll-like receptor	TC09000601.hg.1	-4.25765	6.51E-06	9.49E-05	-1.22011	0.408434	0.666991	ILMN_1706217	-1.61129	0.0209684	0.0771275
TLR5	7100	toll-like receptor	TC01003871.hg.1	1.01357	0.804804	0.869137	1.14286	0.0300761	0.174708	ILMN_1722981	-1.86113	0.00833306	0.0515722
TLR6	10333	toll-like receptor	TC04002917.hg.1	-1.48872	0.0373181	0.0932832	1.22887	0.279467	0.551199	ILMN_1749287	-1.21306	0.0337711	0.0983847
TLR7	51284	toll-like receptor	TC0X000051.hg.1	-1.91769	0.00199095	0.00919864	-1.53691	0.0342789	0.18548	ILMN_1677827	-1.27371	0.361975	0.366364
TLR8	51311	toll-like receptor	TC0X000052.hg.1	-1.58299	0.112278	0.211648	2.14159	0.017068	0.125923	ILMN_1705047	-1.72478	0.0557992	0.128009
TLR9	54106	toll-like receptor	TC03001448.hg.1	1.71168	8.69E-08	4.06E-06	1.14558	0.0445152	0.213844	ILMN_1679798	-1.37081	0.0299311	0.0917533
TLR10	81793	toll-like receptor	TC04001123.hg.1	-1.24892	0.00641267	0.0239462	-1.22999	0.0133739	0.112776	ILMN_2298366	-1.17875	0.000449905	0.0314472
CIITA	4261	NOD-like receptor	TC16000158.hg.1	-1.05862	0.694807	0.787029	-1.24945	0.1555	0.409262	ILMN_1773363	-1.0539	0.704662	0.512268
NAIP	4671	NOD-like receptor	TC05001455.hg.1	-1.00496	0.972055	0.981741	1.63304	0.00383258	0.05629	ILMN_2345812	-1.04837	0.0865363	0.162887
NOD1	10392	NOD-like receptor	TC07001249.hg.1	1.23399	0.000365725	0.00248457	1.09518	0.083901	0.297993	ILMN_2114422	1.16468	0.0355061	0.100896
NOD2	64127	NOD-like receptor	TC16000442.hg.1	-1.65423	0.0275981	0.0743184	1.31292	0.230172	0.498928	ILMN_1762594	-1.58607	0.0891737	0.1656
NLRC3	197358	NOD-like receptor	TC16000820.hg.1	1.26896	0.000504218	0.00317192	1.07236	0.245138	0.517285	ILMN_1740576	-1.09253	0.0580738	0.130876
NLRC4	58484	NOD-like receptor	TC02001723.hg.1	-1.02609	0.536952	0.661801	1.04454	0.323758	0.598754	ILMN_1796976	-1.31341	0.0646413	0.138571
NLRC5	84166	NOD-like receptor	TC16000482.hg.1	1.50805	0.000281765	0.00190504	1.4018	0.00232298	0.0427842	ILMN_1716704	1.53837	0.0123377	0.0607165
NLRP1	22861	NOD-like receptor	TC17001059.hg.1	-1.30491	0.0161408	0.0492812	-1.17391	0.142783	0.391471	ILMN_2313079	-1.16413	0.488119	0.428809
NLRP2	55655	NOD-like receptor	TC19000887.hg.1	1.16144	0.00535628	0.0213082	-1.04107	0.4231	0.681314	ILMN_1664878	1.53195	0.0401293	0.107436
NLRP3	114548	NOD-like receptor	TC01002008.hg.1	-12.7576	6.31E-08	3.04E-06	-2.9719	0.00161612	0.0352787	ILMN_2310896	-2.08093	0.0159045	0.0676159
NLRP4	147945	NOD-like receptor	TC19000913.hg.1	1.00183	0.943643	X	-1.00449	0.869093	X	ILMN_1746820	1.07693	0.147154	0.222177
NLRP5	126206	NOD-like receptor	TC19000915.hg.1	1.01133	0.716271	0.807597	-1.01609	0.624104	0.821736	ILMN_1814652	-1.04713	0.35253	0.36114
NLRP6	171389	NOD-like receptor	TC11000007.hg.1	-1.25205	0.000471242	0.00301668	-1.13337	0.0344052	0.188733	ILMN_1702970	1.05511	0.174657	0.244215
NLRP7	199713	NOD-like receptor	TC19001853.hg.1	-1.00615	0.782983	X	-1.02544	0.289575	X	ILMN_1652366	-1.13712	0.16561	0.237093
NLRP8	126205	NOD-like receptor	TC19000914.hg.1	-1.00429	0.884135	X	1.00149	0.961496	X	ILMN_2075794	-1.43622	0.0143874	0.0650277
NLRP9	338321	NOD-like receptor	TC19001880.hg.1	-1.00144	0.950314	X	1.01407	0.566533	X	ILMN_1698897	1.01182	0.805544	0.545502
NLRP10	338322	NOD-like receptor	TC11001381.hg.1	1.00668	0.857446	X	1.01366	0.727366	X	ILMN_1656992	-1.0206	0.549073	0.455956
NLRP11	204801	NOD-like receptor	TC19001881.hg.1	-1.06325	0.00658564	X	-1.02561	0.239099	X	ILMN_1790843	1.0297	0.244601	0.294625
NLRP12	91662	NOD-like receptor	TC19001826.hg.1	-1.17831	0.00043312	0.00296072	-1.02063	0.610076	0.812342	ILMN_1716105	-2.54465	0.0012151	0.0335386
NLRP13	126204	NOD-like receptor	TC19001882.hg.1	-1.0127	0.699407	X	-1.01582	0.647264	X	ILMN_1739386	1.0185	0.630776	0.487731
NLRP14	338323	NOD-like receptor	TC11000143.hg.1	-1.01701	0.571301	X	-1.01945	0.537981	X	ILMN_1782651	-1.04959	0.188878	0.254971
NLRX1	79671	NOD-like receptor	TC11001087.hg.1	1.09327	0.0100069	0.0368063	1.02812	0.400845	0.66814	ILMN_1698752	1.06224	0.275727	0.31497
TNF	7124	TNF	TC06000371.hg.1	-11.1899	6.16E-10	1.42E-07	-5.01977	3.91E-07	0.000300376	ILMN_1728106	-1.58796	0.05533	0.127371
TNFSF4	7292	TNF	TC01003525.hg.1	-1.08884	0.00818517	0.0320338	-1.03311	0.288787	0.57169	ILMN_2089875	-2.02737	0.0157946	0.0674107
TNFSF8	944	TNF	TC09001516.hg.1	-6.70369	3.35E-10	1.06E-07	-2.33135	3.54E-05	0.00415777	ILMN_1761778	-1.38633	0.0684733	0.143012
TNFSF9	8744	TNF	TC19000117.hg.1	-1.42114	0.0014308	0.00718347	-1.82234	1.06E-05	0.00206327	ILMN_1751464	-1.07854	0.249705	0.298071
TNFSF10	8743	TNF	TC03002006.hg.1	-1.20117	0.491616	0.617622	1.67177	0.0778263	0.282614	ILMN_1801307	1.34405	0.169259	0.240152
TNFSF11	8600	TNF	TC13000154.hg.1	-1.03744	0.143962	X	-1.02524	0.335559	X	ILMN_1658713	-1.06288	0.0579993	0.130768
TNFSF12	8742	TNF	TC17002864.hg.1	-1.05886	0.148133	0.266665	-1.03998	0.335731	0.610465	ILMN_1683700	-1.45407	0.0155063	0.0669968
TNFSF13	8741	TNF	TC17002865.hg.1	-1.0378	0.698946	0.790343	1.18669	0.102074	0.329629	ILMN_1784264	-1.14391	0.427837	0.400716
TNFSF13B	10673	TNF	TC13000383.hg.1	-1.5418	0.0750204	0.156867	1.46078	0.131748	0.3755	ILMN_2066858	-1.41912	0.131547	0.20847
TNFSF14	8740	TNF	TC19001102.hg.1	-3.0146	2.31E-06	4.24E-05	-2.29639	0.000105004	0.00743584	ILMN_2363392	-1.32376	0.00332184	0.0398558
TNFSF15	9966	TNF	TC09001515.hg.1	-1.05644	0.177845	0.303111	-1.06226	0.158721	0.4206	ILMN_2106380	-1.20176	0.0176258	0.0712502
TNFSF18	8995	TNF	TC01003523.hg.1	-1.00683	0.858577	X	-1.05366	0.203166	X	ILMN_1738335	1.03959	0.309065	0.336014
TNFRSF4	7293	TNF receptor	TC01002086.hg.1	-1.06041	0.125306	0.236152	-1.03301	0.406099	0.669832	ILMN_2112256	-2.17186	0.0038498	0.0405708
TNFRSF8	943	TNF receptor	TC01000154.hg.1	-1.09309	0.0493062	0.118216	-1.11023	0.0298678	0.176269	ILMN_1659257	-1.40414	0.00402722	0.0410481
TNFRSF9	3604	TNF receptor	TC01002163.hg.1	-1.73087	0.000287429	0.00192113	-1.80023	0.000233344	0.0119791	ILMN_1813379	-1.02932	0.681906	0.505033
TNFRSF10A	8797	TNF receptor	TC08001052.hg.1	-1.33899	0.00177981	0.00858019	1.04714	0.583346	0.793626	ILMN_1721316	-1.7851	0.0380373	0.104171
TNFRSF10B	8795	TNF receptor	TC08001049.hg.1	-1.79167	2.68E-09	4.11E-07	-1.32476	6.67E-05	0.00617847	ILMN_1699265	-2.14256	0.00501983	0.042769

Gene Symbol	Entrez Gene ID	Gene Category	Affymetrix HTA2.0 Transcript ID	Fold-Change (AIM/control)	P.Value (AIM/control)	adj.P.Val (AIM/control)	Fold-Change (CAEBV/control)	P.Value (CAEBV/control)	adj.P.Val (CAEBV/control)	Illumina Human Ref8 Probe ID	Fold Change (AIM/Baseline)	P.Value (AIM/Baseline)	adj.P.Value (AIM/Baseline)
TNFRSF10D	8793	TNF receptor	TC08001051.hg.1	-1.73896	2.92E-05	0.000306941	-1.24405	0.0472345	0.218532	ILMN_1666022	-1.21781	0.0571571	0.129566
TNFRSF11A	8792	TNF receptor	TC18000221.hg.1	-1.09847	0.0263793	0.0753082	-1.06662	0.129356	0.378966	ILMN_1698952	1.01691	0.692655	0.50836
TNFRSF11B	4982	TNF receptor	TC08001552.hg.1	-1.02179	0.523655	X	-1.03309	0.361596	X	ILMN_1676663	-1.02735	0.321095	0.342885
TNFRSF12A	51330	TNF receptor	TC16000095.hg.1	-1.01841	0.791018	0.859445	-1.04262	0.56484	0.781253	ILMN_1689004	1.07382	0.234123	0.287098
TNFRSF13B	23495	TNF receptor	TC17001181.hg.1	-1.1102	0.004593	0.019933	-1.09653	0.0138334	0.11956	ILMN_1759075	-1.8291	0.0417943	0.109796
TNFRSF13C	115650	TNF receptor	TC22000835.hg.1	-1.77233	2.12E-05	0.000239991	-1.81018	2.42E-05	0.00325792	ILMN_1731742	-1.41363	0.000227824	0.0273185
TNFRSF14	8764	TNF receptor	TC01000061.hg.1	-1.29838	0.00901436	0.03108	-1.17102	0.106893	0.338284	ILMN_1697409	1.08138	0.239007	0.290569
TNFRSF17	608	TNF receptor	TC16000166.hg.1	-1.03086	0.519312	0.644828	1.00287	0.953492	0.980074	ILMN_1768016	-1.9012	0.165302	0.236826
TNFRSF18	8784	TNF receptor	TC01002085.hg.1	-1.23775	0.000316936	0.00220405	-1.1919	0.00253171	0.0461305	ILMN_2349633	-1.30395	0.0391383	0.106055
TNFRSF19	55504	TNF receptor	TC13000049.hg.1	-1.04632	0.0807986	X	-1.00093	0.971248	X	ILMN_1783244	-1.045	0.0203669	0.0761619
TNFRSF1A	7132	TNF receptor	TC12001135.hg.1	-1.40383	0.109522	0.207872	-1.05428	0.804662	0.913747	ILMN_1685005	-1.17352	0.124148	0.20158
TNFRSF1B	7133	TNF receptor	TC01000155.hg.1	-1.59711	0.0595444	0.131876	-1.60094	0.0697918	0.266221	ILMN_1764788	-1.17315	0.144536	0.219647
TNFRSF21	27242	TNF receptor	TC06001777.hg.1	-1.90812	6.12E-09	6.64E-07	-1.576	1.49E-06	0.000727875	ILMN_1699695	-2.81597	0.00677528	0.0476574
TGFA	7039	transforming growth factor	TC02001963.hg.1	-1.23675	0.00012565	0.0010659	-1.10294	0.0434655	0.213732	ILMN_2083946	-1.42453	0.00531204	0.0436766
TGFB1	7040	transforming growth factor	TC19001553.hg.1	-1.17839	0.0990501	0.1943	-1.07087	0.496653	0.73503	ILMN_2129668	1.03267	0.28246	0.319823
TGFB2	7042	transforming growth factor	TC01001805.hg.1	-1.06749	0.0211845	X	-1.04708	0.105936	X	ILMN_1812526	1.01628	0.577713	0.46747
TGFB3	7043	transforming growth factor	TC14001326.hg.1	-1.00363	0.919618	X	1.00698	0.853417	X	ILMN_1687652	-1.17762	0.0169778	0.0698043
TGFBR1	7046	transforming growth factor receptor	TC09000500.hg.1	-1.00482	0.942485	0.962075	-1.03818	0.593275	0.800375	ILMN_1745927	-1.00625	0.95124	0.585381
TGFBR2	7048	transforming growth factor receptor	TC03000149.hg.1	-1.38346	0.0292418	0.0777856	-1.52207	0.00925421	0.0928229	ILMN_2384241	-1.19742	0.0801453	0.155262
TGFBR3	7049	transforming growth factor receptor	TC01002864.hg.1	3.07556	1.00E-05	0.000131614	1.56966	0.029032	0.167912	ILMN_1784287	1.87498	0.020317	0.0761435
C1QA	712	complement component	TC01000285.hg.1	1.08003	0.281276	0.414651	1.21602	0.0155069	0.121295	ILMN_1737918	1.40642	0.0047205	0.0421516
C1QB	713	complement component	TC01000287.hg.1	1.33262	0.00970143	0.0328724	1.63748	0.000185092	0.0103329	ILMN_1796409	2.95467	0.000316923	0.028897
C1QC	714	complement component	TC01000286.hg.1	1.35	0.0728515	0.153514	1.87263	0.00143658	0.0331514	ILMN_1785902	2.60314	0.000551885	0.0333402
C1R	715	complement component	TC12001156.hg.1	1.06888	0.0192474	0.063095	1.02154	0.440194	0.702631	ILMN_1677198	1.22833	0.0197416	0.0748466
C1S	716	complement component	TC12003224.hg.1	-1.01868	0.38758	X	1.01412	0.530248	X	ILMN_1781626	-1.01107	0.49367	0.431369
C2	717	complement component	TC06004076.hg.1	1.14969	0.0338873	0.0882427	1.56506	2.16E-06	0.000923417	ILMN_1710740	1.50169	0.00516535	0.0432062
C3	718	complement component	TC19001103.hg.1	-1.41446	2.34E-05	0.000266614	-1.31932	0.00039337	0.0161058	ILMN_1662523	-1.02827	0.417905	0.395848
C4B	721	complement component	TC06004079.hg.1	-1.09324	0.014165	0.0478434	-1.01432	0.682066	0.85258	no probe			
C5	727	complement component	TC09001542.hg.1	1.01164	0.685592	0.785114	1.05509	0.0868543	0.312934	ILMN_1746819	-1.14982	0.276547	0.315428
C6	729	complement component	TC05001304.hg.1	-1.02725	0.243748	X	1.00477	0.840883	X	ILMN_1688242	1.02576	0.429908	0.40167
C7	730	complement component	TC05000187.hg.1	-1.0102	0.66662	X	1.02255	0.371173	X	ILMN_1687848	1.07686	0.00636155	0.0464817
C8A	731	complement component	TC01000686.hg.1	-1.04173	0.121466	0.238499	-1.01802	0.505904	0.74894	ILMN_1710606	1.0169	0.624709	0.485429
C8B	732	complement component	TC01002704.hg.1	-1.02909	0.277916	X	-1.02102	0.448644	X	ILMN_1731293	-1.02577	0.0939506	0.171052
C8G	733	complement component	TC09000828.hg.1	-1.03936	0.304846	0.445958	-1.0127	0.745463	0.886351	ILMN_1737242	1.08513	0.250403	0.298528
C9	735	complement component	TC05003424.hg.1	-1.02903	0.293128	0.440067	-1.02117	0.459337	0.717216	ILMN_1767831	1.07848	0.0066308	0.047257
CFB	629	complement factor	TC06004077.hg.1	-1.27805	0.0010758	0.00581372	-1.12386	0.0911031	0.309852	ILMN_1774287	1.44931	0.00734104	0.0489181
CFD	1675	complement factor	TC19000020.hg.1	-1.26365	0.00638839	0.0238439	-1.15909	0.0782474	0.284618	ILMN_1777190	-2.33956	0.00900729	0.0531424
CFH	3075	complement factor	TC01006291.hg.1	1.00172	0.967309	0.978654	1.12177	0.0169957	0.129524	ILMN_2412192	-1.21773	0.00504885	0.0427961
CFI	3426	complement factor	TC04001466.hg.1	-1.03771	0.136434	X	-1.02024	0.430739	X	ILMN_1727815	-1.03092	0.450653	0.411804
CFP	5199	complement factor	TC0X000998.hg.1	-1.5453	0.000521517	0.00315002	-1.12122	0.297312	0.569871	ILMN_1658121	-1.92898	0.0205078	0.0763518
C3AR1	719	complement receptor	TC12001171.hg.1	-1.36963	0.100701	0.195934	1.42039	0.0823634	0.291453	ILMN_1787529	-1.33254	0.049586	0.120159
C5AR1	728	complement receptor	TC19000676.hg.1	-8.41825	2.25E-05	0.000246352	-2.10129	0.0700041	0.266655	ILMN_1689836	-3.18839	0.00269322	0.0372634
C5AR2	27202	complement receptor	TC19000677.hg.1	-1.43573	0.0124723	0.0399844	-1.07887	0.582349	0.792498	ILMN_1669317	-1.10628	0.123118	0.200922
CR1	1378	complement receptor	TC01001738.hg.1	-2.97614	4.85E-05	0.000452001	-1.34105	0.181146	0.442754	ILMN_2388112	-1.2716	0.0433982	0.112111
CR2	1380	complement receptor	TC01001737.hg.1	-1.01887	0.849646	0.901852	-1.13508	0.23078	0.50084	ILMN_2369666	-1.6476	0.00603141	0.0455589

Note: X -transcriopt was excluded in multiple test correction

Supplementary Table S5 -Primers used in qPCR assays

Gene Symbol	Forward	Reverse	Amplicon Size (bp)
CCL2	GGCTGAGACTAACCCAGAAAC	GAATGAAGGTGGCTGCTATGA	116
CCNA2	TCCTCCTTGGAAAGCAAACA	GGGCATCTTCACGCTCTATT	116
CCR5	CCCAGTGGGACTTTGGAAATA	CGATTGTCAGGAGGATGATGAA	99
CD3D	GGAACTTGAGGACAGAGTGTTT	CCCAGGTCCAGTCTTGTAATG	102
CD3E	GCATGGAGATGGATGTGATGT	TGGCCTTGGCCTTTCTATTC	114
CD3G	GTCCTCATCCTGGCTATCATTC	GTCAGAAGTACCGAACCATCTT	110
CD8A	TCCACACCATTCAGGTCTTTC	GCACAGTATCCCAGGTATCAAG	106
CD8B	GGTCCTGATGAGTGGATCTTTAC	GTTGATGGTGGCAGAGAAGT	99
CXCR1	CTGGTGATGCTGGTCATCTTAT	CAAGGTCAGGGCAAAGAGTAG	102
FCGR1A	TAAGTCACAATGGCACCTACC	CAGCACTGGAGCTGGAAATA	107
FCGR1B	AAGTCACAATGGCACCTACC	AAGGACATGAAACCAGACAGG	121
GZMA	GAGACTCGTGCAATGGAGATT	CGAGGGTCTCCGCATTTATT	100
GZMK	CTTGTCCCGCCTCATACAAA	CCCATATGTGCTCTGCTTTACT	117
IFNGR1	ACGAGCAGGAAGTCGATTATG	TCTTCCTTCTGCGTGAGTATTT	115
IL1B	TTCTTCGACACATGGGATAACG	TGGAGAACACCACTTGTTGCT	169
IL1R1	GCTTCAGATGGAAAGACCTATGA	CAAGACCTCAGGCAAGACTTTA	111
IL2RA	CTGGGCAACAGAGCAAGAT	GCCTCCCGGTGATTCTAATATG	109
IL4R	GCTATGACTAAAGCAGGGACAA	CAGGGTGGCTGAGCATATTTA	110
IL6	CCAGGAGAAGATTCCAAAGATGTA	CGTCGAGGATGTACCGAATTT	94
IL6R	CCACTCCTGGAACTCATCTTTC	GTCCTTGACCATCCATGTTGT	94
IL8	CTTGGCAGCCTTCCTGATTT	GGGTGGAAAGGTTTGGAGTATG	111
KLRD1	GTACCGGTGCAACTGTTACT	CTGAAGCAGGCTGGATTTCT	94
MKI67	GACCTCAAACTGGCTCCTAATC	GCTGCCAGATAGAGTCAGAAAG	103
SLAMF6	CCTAAGAGAAACCTTCCCAGAC	CCTCCCAAAGTACAGGGATTAC	93
TLR2	GAAGAGTGAGTGGTGCAAGTAT	AATGGGCTCCAGAAGAATGAG	97
TLR4	GATGAGGACTGGGTAAGGAATG	GGCCACACCGGGAATAAA	102
TNF	ATTGCTTCAGCTCCACGGA	CCCGGGTTATGCTGGTTGTAC	196
TNFSF9	TCCACCTCCTGTGTGATACT	TTGCACCGCTGCATTCTA	99
β-actin	CATGTACGTTGCTATCCAGGC	CTCCTTAATGTCACGCACGAT	250
